# Computational and *In Vitro* Analysis of Plumbagin’s Molecular Mechanism for the Treatment of Hepatocellular Carcinoma

**DOI:** 10.3389/fphar.2021.594833

**Published:** 2021-04-12

**Authors:** Yanfei Wei, Yuning Lin, Wanjun Chen, Shasha Liu, Lijie Jin, Delun Huang

**Affiliations:** Department of Physiology, Guangxi University of Chinese Medicine, Nanning, China

**Keywords:** plumbagin, hepatocellular carcinoma, network pharmacology, ROS, PI3K/Akt pathway, MAPK pathway

## Abstract

Hepatocellular carcinoma (HCC) is the fifth most common malignant tumor and the second leading cause of cancer-related death in the world. Plumbagin (PL) is a small molecule naphthoquinone compound isolated from *Plumbago zeylanica* L*.* that has important anticancer properties, but its mechanism requires further investigation. In this study, we used a comprehensive network pharmacology approach to study the mechanism of action of PL for the treatment of HCC. The method includes the construction of multiple networks; moreover, Gene Ontology (GO) and Kyoto Encyclopedia of Genes and Genomes (KEGG) analyses were performed to identify biological processes and signaling pathways. Subsequently, *in vitro* experiments were performed to verify the predicted molecular mechanisms obtained from the network pharmacology-based analysis. Network pharmacological analysis showed that PL may exert anti-HCC effects by enhancing reactive oxygen species (ROS) production to generate oxidative stress and by regulating the PI3K/Akt and MAPK signaling pathways. *In vitro* experiments confirmed that PL mainly mediates the production of ROS, regulates the PI3K/Akt and MAPK signaling pathways to promote apoptosis and autophagy, and shows significant therapeutic effects on HCC. In conclusion, our work proposes a comprehensive systems pharmacology approach to explore the potential mechanism of PL for the treatment of HCC.

## Introduction

Hepatocellular carcinoma (HCC) comprises a group of malignant tumors that seriously threaten human life. Surgical treatment is currently the accepted treatment of choice, but the high recurrence and high metastatic characteristics of HCC severely restrict the survival prognosis of HCC patients (Farazi and Depinho, 2006). In recent years, studies have found that traditional Chinese medicine can inhibit the growth and proliferation of HCC cells in various ways and has a prominent role in HCC treatment (Franco and Usatoff, 2001; Belghiti and Fuks, 2012). Therefore, it is a feasible research direction to search for potential liver cancer treatment drugs by screening and selecting biologically active ingredients that can effectively reduce liver cancer mortality (Su et al., 2019).

East Asian (China, Japan, and South Korea) and traditional Chinese medicine (TCM) have been widely used for disease prevention and treatment for more than two thousand years. Chinese herbal medicine is an important part of Chinese medicine, including plant medicine, animal medicine and mineral medicine. In recent years, Chinese medicine has received increasing attention (Lam et al., 2015). Plumbagin (PL) is a natural naphthoquinone compound isolated and purified from *Plumbago zeylanica* L., a traditional Chinese medicinal plant from China (Tilak et al., 2004; Sakamoto et al., 2011). A large number of studies have shown that PL has antitumor, anti-liver fibrosis, anti-hepatitis B virus, antiplatelet activity, anti-atherosclerosis and other effects (Panda and Kamble, 2016). Studies have shown that PL has significant inhibitory effects on leukemia, lung cancer, prostate cancer, melanoma, breast cancer and other malignant tumors (Subramaniya et al., 2011; Tian et al., 2011; Liu et al., 2017). Previous experimental studies have shown that PL can induce HCC SMMC-7721 cell apoptosis by inhibiting the epithelial-mesenchymal transition (EMT) and inhibit HCC cell angiogenesis and proliferation through the SDF-1-CXCR4/CXCR7 axis (Wei et al., 2019; Zhong et al., 2019).

**FIGURE 1:**
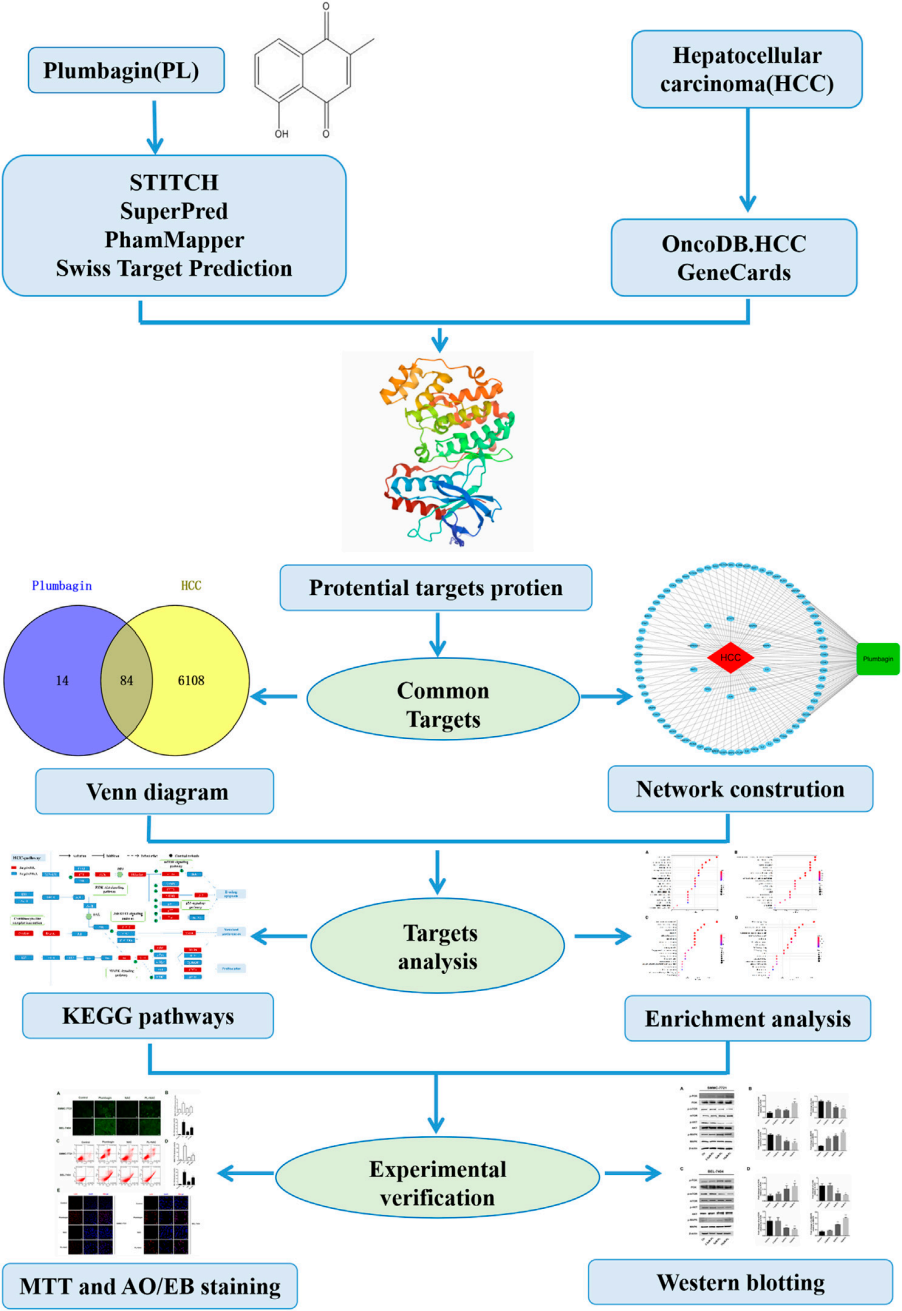
Systems pharmacology approach workflow.

However, more detailed therapeutic targets and signaling mechanisms of PL acting on HCC have not been revealed. Therefore, to further explore the relevant mechanisms of PL acting on HCC, this study aimed to identify therapeutic targets and signaling mechanisms through network pharmacology based on bioinformatics and verify them through relevant experiments *in vitro*. Network pharmacology is an emerging new method to explore the systemic mechanism of therapeutic compounds in diseases (Kohl et al., 2011; Szklarczyk et al., 2016a). The workflow is shown in Figure 1. Using network pharmacology to determine herbal targets and potential mechanisms is becoming increasingly important to save money, effort and time required for drug discovery and design (Győrffy et al., 2013; Zhang et al., 2013). Indeed, network pharmacology has successfully led to the construction and visualization of drug-disease-target networks, which are helpful for evaluating drug mechanisms from multiple perspectives (Gao et al., 2016; Fang et al., 2017). Therefore, network pharmacology can be used to determine the pharmacological target and mechanism of action of PL in HCC and provide guidance and evidence for future research on the use of PL to treat HCC.

## Materials and Methods

### Network Pharmacology-Based Analysis

#### Identifying PL Targets

The predicted genes were obtained by searching the keyword “plumbagin” in the STITCH database (http://stitch.embl.de) (Szklarczyk et al., 2016b). The chemical structural formula of PL was entered into the SuperPred database (http://prediction.charite.de) (Mathias et al., 2008) and the PhamMapper database (http://lilab.ecust.edu.cn/pharmmapper/help.php) (Li et al., 2017). Additionally, the UniProt database (https://www.UniProt.org/) was combined with the literature to collect PL-predictive genes (Rolf et al., 2004), and the Swiss Target Prediction database (http://www.swisstargetprediction.ch/) (Antoine et al., 2019) was used to supplement the gene prediction information. We predicted the target genes of the compound by selecting the compound with the highest “Tanimoto score” and set the “minimum required interaction score” to “high confidence (0.700)” in STITCH. The threshold of “gene probability” was set to >0.6, and the related genes of the predicted compounds in the “Swiss Target Forecast” were selected.

#### Identification of HCC Prediction Genes

The significant prediction genes of HCC were retrieved from OncoDB.HCC (http://oncodb.hcc.ibms.sinica.edu.tw/) (Su et al., 2007) and the GeneCards database (https://www.genecards.org/) (Marilyn et al., 2010). OncoDB.HCC can effectively integrate data sets in public references, provide a multidimensional view of current HCC research, and is the first comprehensive HCC genome database. Potential genes associated with HCC were collected from GeneCards with the keyword “Hepatocellular carcinoma.”

#### Construction of the Components–Targets–Disease Network

Venny2.1.0 (http://bioinfogp.cnb.csic.es/tools/venny/) (Shade and Handelsman, 2012) was used to screen common targets related to PL and HCC. The Cytoscape v3.7.1 (Shannon et al., 2003) software was used to establish target disease and component-target network models, and the merge function was used to build component-target-disease network models. The results were analyzed to determine the relationships in the network model. The proteins were sorted according to their degree of binding.

#### Gene Ontology and KEGG Pathway Analysis

Gene Ontology and KEGG pathway analyses can clarify the role of potential targets by gene function and signaling pathways. The Bioconductor package “org.Hs.eg.db” was installed and run in the R software (Stoll et al., 2005). The drug-disease common targets were converted into Entrez IDs, and then the “clusterProfiler” package was installed in the R software. According to the converted Entrez IDs, enrichment analysis of key target gene GO functions and analysis of KEGG signaling pathways were performed with *p* < 0.05. The results were output in the form of bar and bubble charts.

## Experimental Validation

### Chemicals and Reagents

Plumbagin (PL) was purchased from Sigma-Aldrich (St. Louis, MO, United States) with purity ≥98%; the Acridine Orange (AO)/Ethidium bromide (EB) Double Stain Kit was from Solable Technology (Beijing, China); N-acetyl-l-cysteine, SB203580, and SB202190 were from Sigma-Aldrich (St. Louis, MO, United States); SC-79, MEK2206, 3-MA, and Z-VAD-FMK were from Selleck (Texas, United States); the BCA Protein Assay Kit, ROS Assay Kit, Annexin V-FITC Apoptosis Detection Kit and Cell lysis buffer for Western were all obtained from Beyotime Biotechnology (Shanghai, China); antibodies against Akt, phospho-Akt, mTOR, phospho-mTOR, p38 MAPK, phospho-p38 MAPK, PI3K, phospho-PI3K, LC3B, cleave-RP, and cleave-caspase 3 were from Cell Signaling Technology, Inc. (Boston, MA, United States); and β-p38 MAPK was purchased from Boster Technology (Wuhan, China).

### Cell Lines and Culture

The human HCC cell lines SMMC-7721 and BEL-7404 were purchased from the Shanghai Institute of Biological Sciences (Shanghai, China). The DMEM high-glucose medium required for human HCC SMMC-7721 cell culture and the RPMI-1640 medium required for human HCC BEL-7404 cells were from Thermo Fisher Scientific (MA, United States). The complete culture medium contained 10% fetal bovine serum (FBS), 100 μg/ml penicillin and 100 μg/ml streptomycin, which were obtained from Invitrogen (CA, United States). The cells were cultured in an incubator at 37°C with a humidified atmosphere of 5% CO_2_.

### Cell Viability Assay

Cell operations were performed on an ultraclean workbench. After the cells were recovered, SMMC-7721 and BEL-7404 cells at 80% confluence were digested into seed plates and seeded in 96-well plates at a density of 5 × 10^3^ cells/well. After 24 h, the cells were completely attached to the wall and then synchronized. Each group was equipped with four auxiliary holes. A negative control well was set up without seeding cells. After treatment with various concentrations of PL (0.5, 3, 6, and 10 μM) for 6, 12 or 24 h in a 5% CO_2_ incubator at 37°C, 20 μL of 5 mg/ml MTT solution was added to each well, the plates were wrapped in foil and protected from light, and then placed in an incubator to continue incubation for an additional 3–4 h. The absorbance value of each well was measured using a continuous spectrum scanning microplate reader (Thermo Fisher, MA, United States) at a wavelength of 560 nm.

### AO/EB Staining

SMMC-7721 and BEL-7404 cells in logarithmic growth phase were digested and plated. Three multi-wells were set up and inoculated into a 24-well culture plate according to the standard of 500 μL per well, after which a moderate concentration of PL was added to stimulate the cells for 24 h. The supernatant was discarded after the intervention, a final concentration of 1 μg/ml AO/EB was added, and the plate was incubated for 10 min in the dark. Observation by fluorescence microscopy (Olympus, Tokyo, Japan) revealed that AO produces green fluorescence in the cytosol and the nuclear compartment (emission peak between 530 and 550 nm). Fluorescence microscopy showed four cell morphologies. The nuclear chromatin of living cells was green and showed a normal structure; the nuclear chromatin of early apoptotic cells was green, though the shape was shrunken or rounded; the nuclear spot material of late apoptotic cells was orange-red, and the shape was solid or as round beads; and the nuclear chromatin of nonapoptotic dead cells was orange-red, with normal cell morphology.

### Detection of ROS

ROS production was determined using a 2′,7′-dichlorodihydrofluorescein diacetate (DCFH-DA) assay (Beyotime, Shanghai, China). After treatment, SMMC-7721 and BEL-7404 cells were incubated with DCFH-DA for 30 min at 37°C in the dark. The cells were then harvested and suspended in PBS. The fluorescence intensity in each well was detected by an LSM 700 confocal microscope (Zeiss, Hamburg, Germany).

### Flow Cytometry for Apoptosis Analysis

The instructions of the Annexin V-FITC Apoptosis Detection Kit were strictly followed to process each group of cells. The cells were digested with trypsin without EDTA and washed twice with prechilled PBS, and 10–50 × 10^4^ cells were collected. The cells were then resuspended in 100 μL of 1×Binding Buffer, and then 5 μL each of Annexin V-FITC and PI staining solution were added. The lysates were then mixed gently and incubated at room temperature in the dark for 10 min. The cells were detected by flow cytometry within 1 h. Apoptosis rate = (number of apoptotic cells/total number of cells observed) × 100%.

### Immunofluorescence Staining

The cells were fixed with 4% paraformaldehyde for 15 min and permeabilized with 0.1% Triton X-100 for 10 min. The cells were then blocked with 2% bovine serum albumin (BSA) at 37°C for 30 min, after which the cells were incubated with anti-LC3B (1:100, Cell Signaling Technology, Boston, United States) overnight at 4°C. The cells were then incubated with the corresponding secondary antibodies for 1 h at room temperature. The nuclei were stained with DAPI for 10 min and washed twice with PBS, and then images were captured using an LSM 700 confocal microscope (Zeiss, Hamburg, Germany).

### Western Blotting

SMMC-7721 and BEL-7404 cells treated with the preset intervention conditions were collected and proteins were extracted. After using BSA to measure the absorbance, a standard curve was prepared versus the standard, and the protein concentration of the sample was determined according to the standard curve. The samples were prepared according to the instructions for the BCA protein quantitation kit. Depending on the relative molecular weights of the proteins, 7.5–12.5% PAGE-SDS gel electrophoresis was performed, and the proteins were then transferred to PVDF membranes. The PVDF membranes were blocked with 5% skimmed milk powder for 1 h at room temperature. Then, the following appropriately diluted primary antibodies were added: anti-β-actin (1:200), PI3K (1:1000), p-PI3K (1:500), Akt (1:2000), p-Akt (1:500), mTOR (1:1000), p-mTOR (1:1000), p38 MAPK (1:1000), p-p38 MAPK (1:500), cleave-caspase 3 (1:1000), cleave-PARP (1:1000), and LC3B (1:1000). Next, the blots were incubated at 4°C overnight. After washing three times with PBS, the corresponding secondary antibody was added and incubated for 2 h at room temperature. After incubating with the luminescent solution from the ECL kit, the film was exposed in the cassette, the protein bands were developed, and Band Scan software was used to scan grayscale images.

### Statistical Analysis

Data are expressed as the means ± standard deviation (SD). Statistical analysis was performed using SPSS 22.0. One-way ANOVA was used for comparisons between groups, and the difference was statistically significant at *p* < 0.05.

## Results

### Screening Results of PL Targets and HCC Disease Targets

The 3D chemical structure of PL was imported into the chemical substance interaction search tool STITCH and the SuperPred and PhamMapper databases for retrieval; the Swiss Target Prediction database was used to supplement the target information. Then, the corresponding targets were input into the UniProt database, and after removing the duplicates, 98 targets of PL were obtained; the list of targeted genes is shown in Supplementary Table S1. Potential genes related to HCC were collected from GeneCards with the keyword “hepatocellular carcinoma.” In addition, the potential targets of HCC were retrieved from the OncoDB.HCC database, and after removing the duplicates, 6192 targets related to HCC were obtained; the list of targeted genes is shown in Supplementary Table S2.

### Network Construction

**FIGURE 2:**
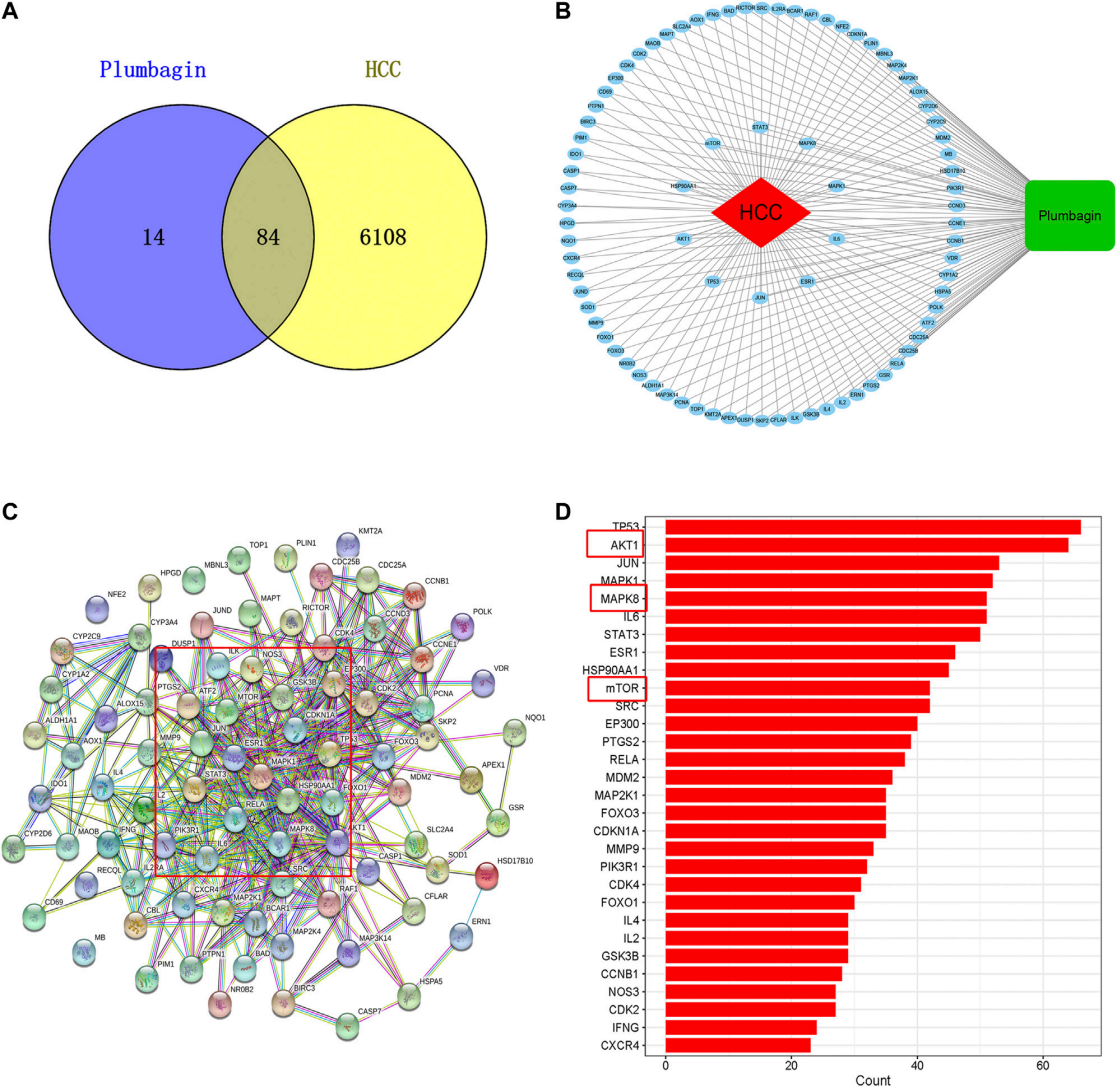
**(A)** Venn diagram of common targets of plumbagin (PL) in HCC. **(B)** Network of PL targets and HCC targets. The rectangle represents the compound PL, the ellipse represents 84 common targets, and the diamond represents HCC. The protein-protein interaction (PPI) network based on targets of PL in HCC. **(C)** Nodes represent different proteins. Edges represent protein-protein associations, and the line thickness indicates the strength of data support. **(D)** Bar plot of the protein-protein interaction (PPI) network. The X-axis represents the number of neighboring proteins of the target protein. The Y-axis represents the target proteins.

Among the 6192 HCC-related targets discovered, 84 targets were common targets corresponding to PL. Through Venny 2.1.0, AKT1, ALDH1A1, MTOR, NR0B2, FOXO3, FOXO1, MMP9, SOD1, JUND, RECQL, CXCR4, NQO1 and other common targets were obtained (Figure 2A and Table 1). The 84 drug-disease common targets of PL and HCC were input into the Cytoscape 3.7.1 software, and a network diagram of the “component-target-disease” interaction was drawn (Figure 2B). The above 84 common targets were entered into the STRING database, and the PPI network of protein interactions was analyzed (Figure 2C). The ranking of the top 30 targets is shown in Figure 2D.

**TABLE 1:** Common targets between PL and HCC.

Gene name	Gene symbol	Uniprot ID
Histone acetyltransferase p300	EP300	Q09472
Indoleamine 2,3-dioxygenase	IDO1	P14902
Dual specificity phosphatase Cdc25B	CDC25B	P30305
Serine/threonine-protein kinase PIM1	PIM1	P11309
Dual specificity protein phosphatase 1	DUSP1	P28562
Monoamine oxidase B	MAOB	P27338
Glutathione reductase	GSR	P00390
Serine/threonine-protein kinase/endoribonuclease IRE1	ERN1	O75460
Myoglobin	MB	P02144
C-X-C chemokine receptor type 4	CXCR4	P61073
RAC-alpha serine/threonine-protein	AKT1	P31749
Mitogen-activated protein kinase 8	MAPK8	P45983
Superoxide dismutase [Cu-Zn]	SOD1	P00441
Transcription factor NF-E2	NFE2	Q16621
Solute carrier family 2	SLC2A4	P14672
Proto-oncogene tyrosine-protein kinase	SRC	P12931
Cyclin-dependent kinase inhibitor 1	CDKN1A	P38936
DNA topoisomerase I	TOP1	P11387
RAF proto-oncogene serine/threonine-protein kinase	RAF1	P04049
Tumor suppressor p53/oncoprotein Mdm2	TP53	P04637
Vitamin D receptor	VDR	P11473
15-Hydroxyprostaglandin dehydrogenase [NAD+]	HPGD	P15428
78 kDa glucose-regulated protein	HSPA5	P11021
Aldehyde dehydrogenase 1A1	ALDH1A1	P00352
Arachidonate 15-lipoxygenase	ALOX15	P16050
ATP-dependent DNA helicase Q1	RECQL	P46063
Caspase-1	CASP1	P29466
Caspase-7	CASP7	P55210
DNA polymerase kappa	POLK	Q9UBT6
Dual specificity mitogen-activated protein kinase kinase	MAP2K1	Q02750
Endoplasmic reticulum-associated amyloid beta-peptide-binding protein	HSD17B10	Q99714
Microtubule-associated protein tau	MAPT	P10636
Mitogen-activated protein kinase kinase kinase 14	MAP3K14	Q99558
Mitogen-activated protein kinase; ERK1/ERK2	MAPK1	P28482
Nuclear factor NF-kappa-B p65 subunit	RELA	Q04206
Transcription factor AP-1	JUN	P05412
Transcription factor jun-D	JUND	P17535
Estrogen receptor, ER	ESR1	P03372
Dual specificity mitogen-activated protein kinase kinase 4	MAP2K4	P45985
Signal transducer and activator of transcription 3	STAT3	P40763
Tyrosine-protein phosphatase non-receptor type 1	PTPN1	P18031
E3 ubiquitin-protein ligase CBL	CBL	P22681
Nuclear receptor subfamily 0 group B member 2	NR0B2	Q15466
E3 ubiquitin-protein ligase Mdm2	MDM2	Q00987
Serine/threonine-protein kinase mTOR	MTOR	P42345
Heat shock protein HSP 90-alpha	HSP90AA1	P07900
Cyclin-dependent kinase 2	CDK2	P24941
S-phase kinase-associated protein 2	SKP2	Q13309
E3 ubiquitin-protein ligase CCNB1IP1	CCNB1	Q9NPC3
G1/S-specific cyclin-E1	CCNE1	P24864
G1/S-specific cyclin-D3	CCND3	P30281
Cyclin-dependent kinase 4	CDK4	P11802
Glycogen synthase kinase-3 beta	GSK3B	P49841
Cyclic AMP-dependent transcription factor ATF-2	ATF2	P15336
Integrin-linked protein kinase	ILK	Q13418
Rapamycin-insensitive companion of mTOR	RICTOR	Q6R327
Nitric oxide synthase, endothelial	NOS3	P29474
Zinc finger protein Gfi-1	CDKNIA	Q99684
PCNA-associated factor	PCNA	Q15004
Forkhead box protein O3	FOXO3	O43524
Forkhead box protein O1	FOXO1	Q12778
Breast cancer anti-estrogen resistance protein 1	BCAR1	P56945
Baculoviral IAP repeat-containing protein 3	BIRC3	Q13489
Interleukin-6	IL6	P05231
Phosphatidylinositol 3-kinase regulatory subunit alpha	PIK3R1	P27986
Bcl2-associated agonist of cell death	BAD	Q92934
Histone-lysine N-methyltransferase 2A	KMT2A	Q03164
Interleukin-4	IL4	P05112
Early activation antigen CD69	CD69	Q07108
M-phase inducer phosphatase 1	CDC25A	P30304
CASP8 and FADD-like apoptosis regulator	CFLAR	O15519
Matrix metalloproteinase-9	MMP9	P14780
Prostaglandin G/H synthase 2	PTGS2	P35354
DNA-(apurinic or apyrimidinic site) lyase	APEX1	P27695
Perilipin-1	PLIN1	O60240
Muscleblind-like protein 3	MBNL3	Q9NUK0
Interleukin-2	IL2	P60568
Interleukin-2 receptor subunit alpha	IL2RA	P01589
Cytochrome P450 1A2	CYP1A2	P05177
Ubiquinol oxidase 1a	AOX1	Q39219
Cytochrome P450 3A4	CYP3A4	P08684
Cytochrome P450 2C9	CYP2C9	P11712
Cytochrome P450 2D6	CYP2D6	P10635
Interferon gamma	IFNG	P01579
NAD(P)H dehydrogenase [quinone] 1	NQO1	P15559

### GO and KEGG Pathway Analyses

**FIGURE 3:**
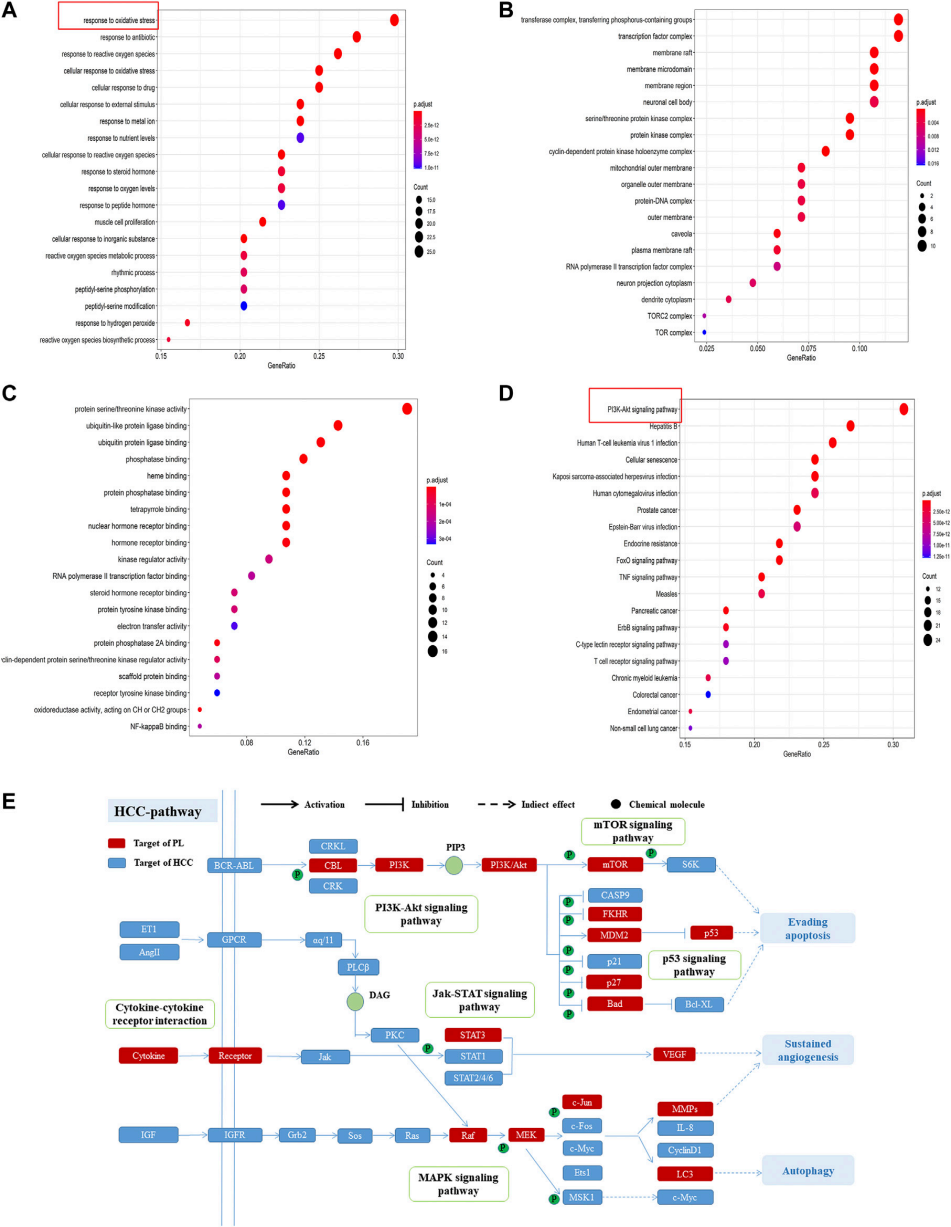
**(A)** GO enrichment analysis of biological processes of common targets of PL and HCC. The Y-axis represents significant GO biological processes, and the X-axis represents the counts of enriched targets. The gradient of color represents the different adjusted *p* values. **(B)** GO enrichment analysis of cell components of common targets of PL and HCC. The Y-axis represents significant cell components, and the X-axis represents the counts of enriched targets. The gradient of color represents the different adjusted *p* values. **(C)** GO enrichment analysis of the molecular functions of common targets of PL and HCC. The Y-axis represents significant GO molecular functions, and the X-axis represents the counts of enriched targets. The gradient of color represents the different adjusted *p* values. **(D)** KEGG analysis for common targets of PL and HCC. The Y-axis represents significant KEGG pathways, and the X-axis represents the ratio of enriched targets in a pathway to all common targets. The size of the nodes shows the count of targets, and the gradient of color represents the adjusted *p* value. **(E)** Distribution of target proteins of PL on the compressed “HCC pathway.” Five pathways form the tubular HCC pathway. Arrows show activation activity, T-arrows show inhibition activity, and segments represent indirect activation effects or inhibition effects.

GO enrichment analysis was used to further study the biological processes, cellular components and molecular functions of the 84 common targets. As shown in Figure 3A, the intersection genes were enriched in 1,560 biological processes, and there were 164 biological processes with a number of enriched genes >10. The results indicated that the top 20 GO items with significant abundance (*p* < 0.001) are mainly related to biological processes such as oxidative stress, reactive oxygen synthesis, cell proliferation, and peptidyl-serine phosphorylation. Intersect gene enrichment analysis of the expression processes of the 27 cell components revealed that they are mainly related to transferase complex, transferring phosphorus-containing groups, transcription factor complex, and membrane raft (Figure 3B). The intersection genes were enriched in 100 processes related to molecular function, mainly related to protein serine/threonine kinase activity and phosphatase binding (Figure 3C). The above results indicate that PL correlates highly with anticancer activity, and PL may exert anti-HCC effects by participating in oxidative stress and reactive oxygen synthesis, regulating protein kinase phosphorylation.

Furthermore, of the 84 common targets, 78 genes participate in 154 KEGG pathways with adjusted *p*-values <0.01; after sorting the adjusted *p*-values, the top 20 pathways were analyzed (Figure 3D). The results show that the common targets of PL and HCC are mainly enriched in the PI3K-Akt, mTOR and MAPK signaling pathways. Then, considering the complex mechanism of PL to treat HCC, a complete “HCC pathway” was constructed by integrating the key pathways obtained through KEGG network analysis, as shown in Figure 3E. The results indicate that these integration pathways involve multiple pathophysiological modules, such as cell survival, proliferation, apoptosis, and angiogenesis. The PI3K/AKT pathway is responsible for regulating cell proliferation and apoptosis, and the mTOR/p38 MAPK pathway is responsible for regulating cell autophagy. Therefore, we performed *in vitro* experiments to validate that PL promotes apoptosis and autophagy through the PI3K-Akt/p38 MAPK signaling pathway, thereby explaining the potential therapeutic mechanism of PL in HCC treatment.

### Experimental Validation

#### PL Inhibits HCC Cell Proliferation

**FIGURE 4:**
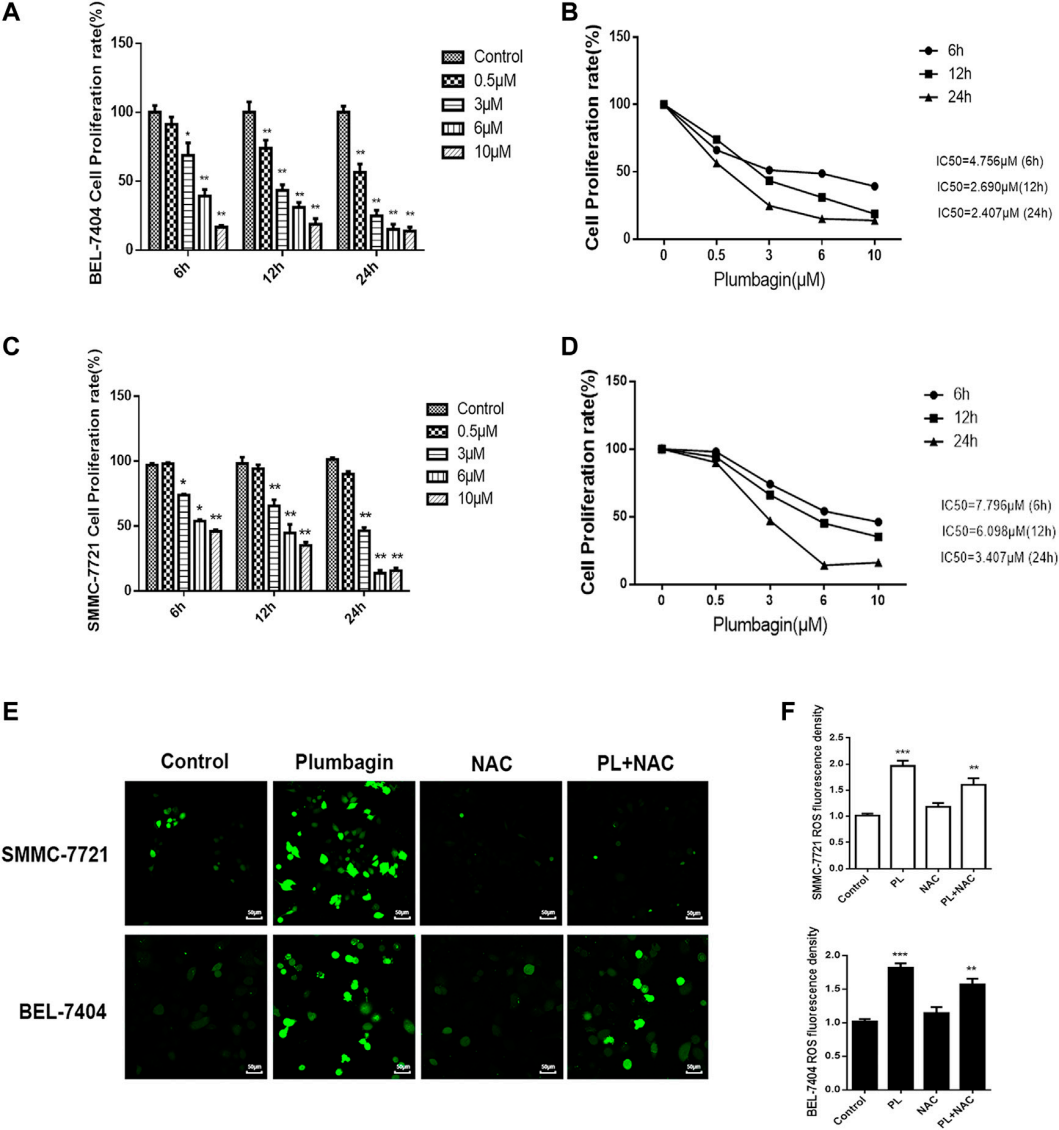
**(A)** Cell viability of BEL-7404 cells treated with various concentrations of PL (0.5, 3, 6, and 10 μM) for 6, 12 or 24 h *; *p* < 0.05, **; *p* < 0.01 ***; *p* < 0.001, compared with the 0 μM group. **(B)** The 50% inhibitory concentrations (IC50) of BEL-7404 cells were 4.756 μM at 6 h, 2.690 μM at 12 h, and 2.407 μM at 24 h. **(C)** Cell viability of SMMC-7721 cells treated with various concentrations of PL (0.5, 3, 6, and 10 μM) for 6, 12 or 24 h; *p* < 0.05, **; *p* < 0.01 ***; *p* < 0.001, compared with the 0 μM group. **(D)** The 50% inhibitory concentrations (IC50) of SMMC-7721 cells were 7.796 μM at 6 h, 6.098 μM at 12 h, and 3.407 μM at 24 h. **(E)** The production of ROS was measured by DCFH-DA to assess the level of oxidative stress. SMMC-7721 and BEL-7404 cells were pretreated with 10 mM NAC for 30 min and were then treated with 5 μM PL for 24 h, and fluorescence was detected by confocal fluorescence microscopy. Scale bar, 50 μm. **(F)** For statistical analysis, the mean DCFH-DA fluorescence intensity (representing cellular ROS level) was measured from 9 random fields for each culture. All values represent the mean ± SEM of three independent experiments. *p < 0.05, **p < 0.01 vs. normal control group, respectively.

The MTT assay was found that PL inhibited BEL-7404 and SMMC-7721 cell proliferation (Lin et al., 2018). And the inhibitory effect of PL on BEL-7404 and SMMC-7721 cell proliferation was concentration- and time-dependent. After treatment with different concentrations of PL (0–10 μM) at three time points (6, 12 and 24 h), the cell proliferation rate decreased significantly (*p* < 0.05). Within the dose range of 3–10 μM, the cell growth inhibition rate reached greater than 50% within 24 h (Figures 4A,C). The half-inhibitory concentrations (IC50) of PL on BEL-7404 hepatocellular carcinoma cells were 4.689 μM at 6 h, 2.770 μM at 12 h, and 2.457 μM at 24 h (Figure 4B). The half-inhibitory concentrations (IC50) of PL on SMMC-7721 HCC cells were 36.05 μM at 6 h, 5.41 μM at 12 h, and 2.43 μM at 24 h (Figure 4D) (Lin et al., 2018).

#### PL Mediated Reactive Oxygen Generation in HCC Cells

Oxidative stress mediates the pathogenesis of liver cancer. The network pharmacology GO analysis results indicate that PL may mediate oxidative stress by activating the production of ROS and may have an anti-liver cancer effect. In our *in vitro* validation experiments, we treated BEL-7404 and SMMC-7721 cells with PL, the antioxidant NAC and PL+NAC and then detected levels of ROS in the cells using fluorescent probe technology. The results are shown in Figure 4E. After PL treatment, ROS expression levels in the two cell groups were significantly increased. Statistical analysis showed that after treatment with PL+NAC antioxidants, ROS expression levels were lower than in the PL group but higher than in the NAC group (Figure 4F). These findings indicate that PL may mediate oxidative stress through the production of ROS and may promote an anti-liver cancer effect.

#### PL Promotes Apoptosis by Mediating the Generation of ROS in HCC Cells

**FIGURE 5:**
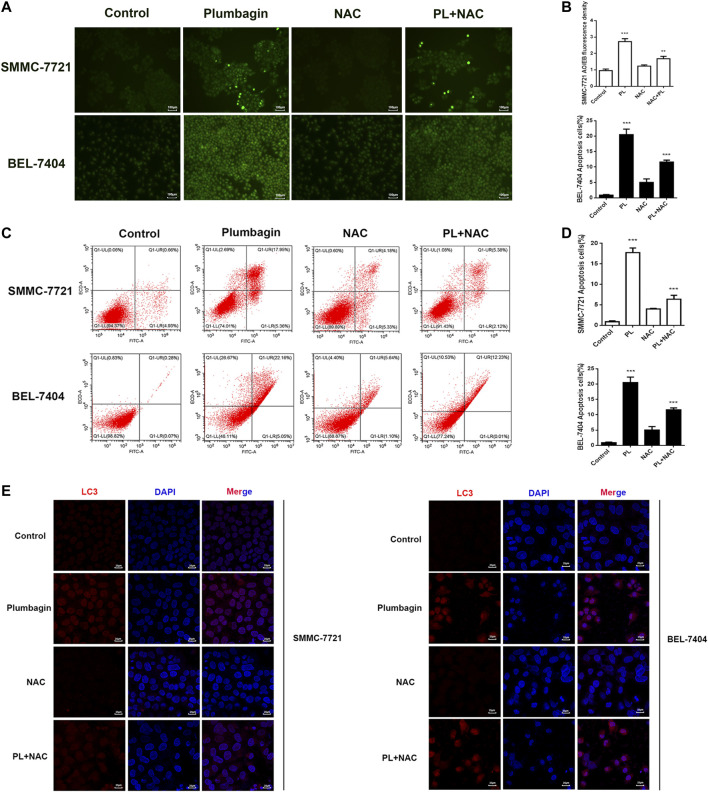
**(A)** AO/EB staining to detect the formation of PL-induced BEL-7404 cells and SMMC-7721 apoptotic bodies. Scale bar, 100 μm. All data are representative of at least three independent experiments. **(B)** Data were shown as the mean ± SD. *p < 0.05, **p < 0.01 vs. the normal control group. **(C)** SMMC-7721 and BEL-7404 cells were pretreated with 10 mM NAC for 30 min and were then treated with 5 μM PL for 24 h. Then, the ratio of apoptosis was measured and analyzed by Annexin V-FITC/PI staining and flow cytometry. **(D)** Data are shown as the mean ± SD. *p < 0.05, **p < 0.01 vs. the normal control group. **(E)** SMMC-7721 and BEL-7404 cells were pretreated with 10 mM NAC for 30 min and were then treated with 5 μM PL for 24 h. Immunofluorescence detection of LC3 expression. The expression of LC3 (red) was significantly higher. The morphology of SMMC-7721 and BEL-7404 cells had a fusiform shape rather than a rounded shape. Cell nuclei were defined by DAPI (blue). Scale bar, 25 μm.

SMMC-7721 and BEL-7404 cells were subjected to AO/EB staining (Lin et al., 2018). Observation under a fluorescence microscope showed that cells in the PL group emitted green fluorescence after AO staining. The nuclei presented one to several bead-shaped bodies of varying sizes, called apoptotic bodies, which are characterized by smooth and clear edges, and the fluorescence under the excitation of a fluorescence microscope is enhanced and uniform. These findings are consistent with the nuclear morphological features of apoptosis. In addition, the cell membrane may display a “budding” phenomenon. Late apoptotic cells with damaged cell membranes were simultaneously stained by EB and showed the same nuclear morphological characteristics, but the fluorescence showed orange-red, the green fluorescence of cells in the PL+NAC group decreased after AO staining, and the formation of apoptotic bodies was reduced, while the green fluorescence intensity was stronger than that of the NAC group alone (Figures 5A,B). These results indicate that PL may mediate apoptosis through ROS.

Moreover, we used flow cytometry to further verify that PL may mediate apoptosis through ROS. As shown in Figures 5C,D, after PL treatment, the percentage of apoptosis increased significantly compared with the control group, mainly with regard to the percentage of late apoptotic cells. The level of apoptosis in the PL+NAC group was significantly reduced. Therefore, these findings further indicate that PL may mediate apoptosis through ROS.

#### PL Induces Autophagy by Mediating the Production of ROS in HCC Cells

The expression level of the autophagy marker LC3 in BEL-7404 and SMMC-7721 cells after PL treatment was assessed by immunofluorescence detection. As depicted in Figure 5E, the red fluorescence of the autophagy marker LC3 in the PL group showed a concentrated state, the fluorescence dot density increased, and the fluorescence intensity increased, indicating that the expression level of LC3 protein increased. In contrast, the fluorescence intensity decreased in the PL+NAC antioxidant group. These findings indicate that PL may induce autophagy by mediating the generation of ROS in HCC cells, thereby playing a role in the treatment of liver cancer.

#### PI3K/Akt/ and mTOR/p38 MAPK Pathway Verification

**FIGURE 6:**
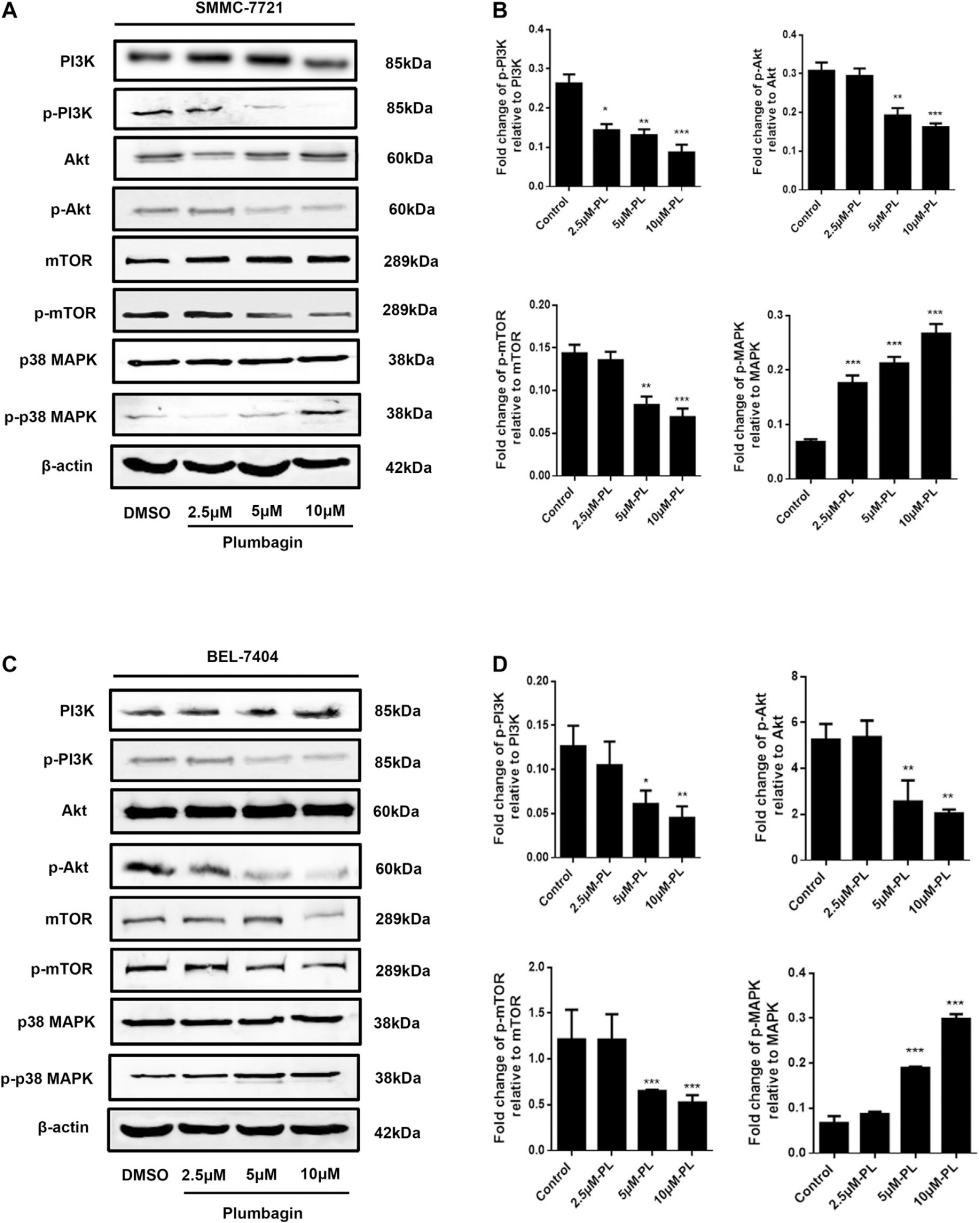
**(A)** Protein expression levels of PI3K, p-PI3K, Akt, p-Akt, mTOR, p-mTOR, MAPK, and p-MAPK in SMMC-7721 cells measured by Western blot after treatment with various concentrations of PL (0.5, 2.5, 5, and 10 μM). **(B)** PI3K, MAPK, Akt, and mTOR were used as internal reference total proteins for gray value analysis. Each bar represents the mean value ± standard deviation (SD) of the intensity of PL-induced cells across triplicate experiments. *p < 0.05, **p < 0.01 ***p < 0.001, compared with the control group. **(C)** Protein expression levels of PI3K, p-PI3K, AKT, p-AKT, mTOR, p-mTOR, MAPK, and p-MAPK in BEL-7404 cells measured by Western blot after treatment with various concentrations of PL (0.5, 2.5, 5, and 10 μM). **(D)** PI3K, MAPK, Akt, and mTOR were used as internal reference total proteins for gray value analysis. Each bar represents the mean value ± standard deviation (SD) of the intensity of PL-induced cells across triplicate experiments. *p < 0.05, **p < 0.01 ***p < 0.001, compared with the control group.

Western blotting revealed no significant difference in expression of Akt and mTOR phosphorylated protein in the PL low concentration group compared with the control group in SMMC-7721 cells (*p* > 0.05). After intervention, the medium- and high-concentration groups of PL showed significantly reduced expression of PI3K Akt and mTOR phosphorylated proteins and upregulated expression of p38 MAPK phosphorylated proteins (Figure 6A). As shown in Figure 6B, when using PI3K, MAPK, Akt, and mTOR as internal reference total proteins for gray value analysis, the difference was statistically significant (*p* < 0.05). For BEL-7404 cells, there was no significant difference in expression levels of PI3K, MAPK, Akt and mTOR phosphorylated proteins in the PL low-concentration group compared with the control group (*p* > 0.05). Additionally, PI3K, Akt, and mTOR phosphorylated protein expression was significantly downregulated in the PL medium- and high-concentration groups, with increased expression of p38 MAPK phosphorylated protein (Figure 6C). As shown in Figure 6D, the difference was statistically significant when using PI3K, MAPK, Akt, and mTOR as internal reference total proteins for gray value analysis (*p* < 0.05).

#### PL Promotes Apoptosis Through the PI3K/Akt Signaling Pathway

**FIGURE 7:**
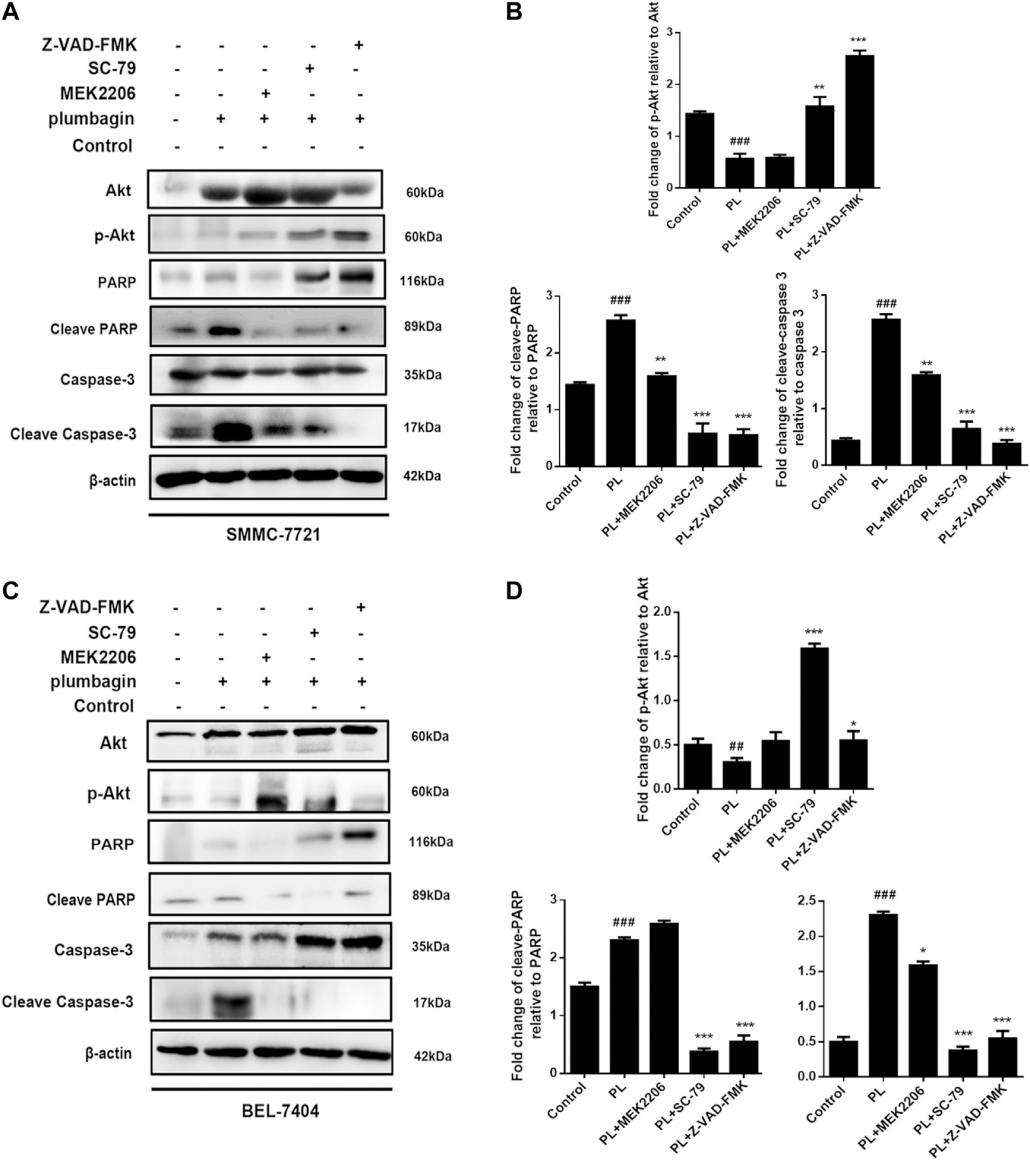
**(A)** Protein expression levels of p-Akt, Akt, PARP, cleaved PARP, caspase 3, and cleaved caspase 3 in SMMC-7721 cells measured by Western blot after treatment with 5 μM PL, 10 μM SC79 Akt agonist, 10 μM MEK2206 Akt inhibitor and 10 μM Z-VAD-FMK caspase inhibitor. **(B)** p-Akt, Akt, PARP, cleaved PARP, caspase 3, and cleaved caspase 3 were used as internal reference total proteins for gray value analysis. *p < 0.05, **p < 0.01 ***p < 0.001, compared with the PL group. #*p* < 0.05, ##*p* < 0.01 vs. the control group, respectively. **(C)** Protein expression levels of p-Akt, Akt, PARP, cleaved PARP, caspase 3, and cleaved caspase 3 in BEL-7404 cells measured by Western blot after treatment with 5 μM PL, 10 μM SC79 Akt agonist, 10 μM MEK2206 Akt inhibitor and 10 μM Z-VAD-FMK caspase inhibitor. **(D)** p-Akt, Akt, PARP, cleaved PARP, caspase 3, and cleaved caspase 3 were used as internal reference total proteins for gray value analysis. *p < 0.05, **p < 0.01 ***p < 0.001, compared with the PL group. #*p* < 0.05, ##*p* < 0.01 ###*p* < 0.001 vs. the control group.

We employed Western blotting to verify that PL mediates apoptosis through regulation of the PI3K/Akt signaling pathway. The results showed that for the SMMC-7721 liver cancer cell line, expression of phosphorylated Akt was significantly downregulated in the PL group compared with the control group. At the same time, protein expression levels of cleave-caspase 3 and cleave-PARP were upregulated, but caspase 3 and PARP total proteins were decreased, as shown in Figures 7A,B. After pretreatment with the Akt agonist SC-79 and apoptosis-targeting Caspase inhibitor Z-VAD-FMK for 1 h followed by PL treatment for 24 h, p-Akt levels increased, and the levels of cleave-caspase 3 and cleave-PARP were reduced; however, caspase 3 and PARP total protein levels remained unchanged. Interestingly, after pretreatment with the Akt inhibitor MEK2206 for 1 h followed by PL incubation for 24 h, there was no significant difference in protein expression levels of p-Akt, cleave-caspase 3 and cleave-PARP compared with the PL treatment group alone.

In the BEL-7404 liver cancer cell line, pretreatment with the Akt agonist SC-79 and the apoptosis-targeting Caspase inhibitor Z-VAD-FMK for 1 h followed by PL treatment for 24 h upregulated p-Akt expression. Protein expression levels of cleave-caspase 3 and cleave-PARP were downregulated, which was consistent with the results for SMMC-7721 cells (Figures 7C,D). The above results indicate that PL mediates apoptosis by regulating the PI3K/Akt signaling pathway.

#### PL Induces Autophagy Through the mTOR/p38 MAPK Signaling Pathway

**FIGURE 8:**
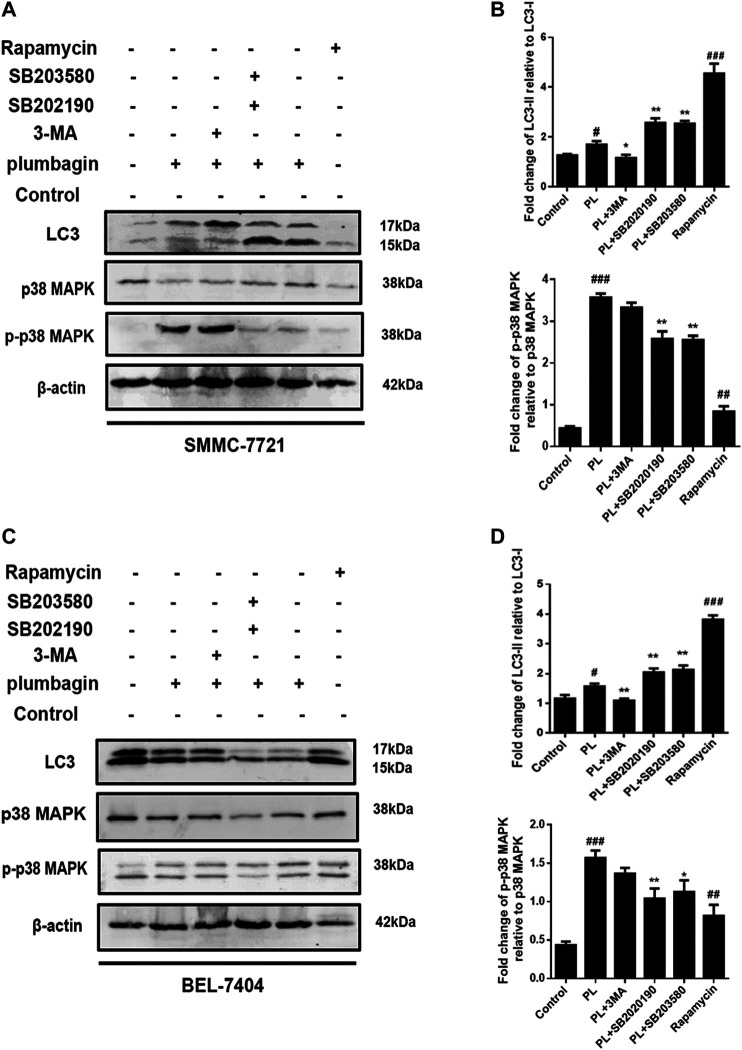
**(A)** Protein expression levels of LC3-I, LC3-II, p38 MAPK, and p-p38 MAPK in SMMC-7721 cells measured by Western blot after treatment with 5 μM PL, 10 μM 3-MA autophagy inhibitor, 10 μM rapamycin autophagy agonist, 10 μM SB202190, and 10 μM SB203580 p38 MAPK inhibitor. **(B)** LC3-I, LC3-II, p38 MAPK, and p-p38 MAPK were used as internal reference total proteins for gray value analysis. *p < 0.05, **p < 0.01 ***p < 0.001, compared with the PL group. #*p* < 0.05, ##*p* < 0.01 ###*p* < 0.001 vs. the control group. **(C)** Protein expression levels of LC3-I, LC3-II, p38 MAPK, and p-p38 MAPK in BEL-7404 cells measured by Western blot after treatment with 5 μM PL, 10 μM 3-MA autophagy inhibitor, 10 μM rapamycin autophagy agonist, 10 μM SB202190, and 10 μM SB203580 p38 MAPK inhibitor. **(D)** LC3-I, LC3-II, p38 MAPK, and p-p38 MAPK were used as internal reference total proteins for gray value analysis. *p < 0.05, **p < 0.01 ***p < 0.001, compared with the PL group. #*p* < 0.05, ##*p* < 0.01 ###*p* < 0.001 vs. the control group.

The experimental results showed that in SMMC-7721 cells, both PL and the autophagy agonist rapamycin upregulated the expression level of the autophagy marker protein LC3 and the phosphorylation level of p-p38 compared with the control group, and the difference was significant. In addition, the autophagy inhibitor 3-MA and the p38 MAPK inhibitors SB202190 and SB203580 jointly interfered in SMMC-7721 cells treated with PL. Compared with the PL treatment alone group, levels of LC3 were significantly downregulated in the p-p383-MA+PL group. These expression levels indicated suppression of PL-induced autophagy. In the SB202190+PL and SB203580+PL groups, LC3 expression was increased, whereas that of p-p38 was significantly decreased (Figures 8A,B).

In BEL-7404 cells, expression levels of the autophagy marker protein LC3 in the PL and autophagy agonist rapamycin groups were significantly increased compared with the blank control group. In addition, the autophagy inhibitor 3-MA and the p38 MAPK inhibitors SB202190 and SB203580 acted in BEL-7404 cells treated with PL. Compared with the PL treatment group, the 3-MA+PL group showed significantly decreased levels of LC3 and p-p38. LC3 expression levels were increased in the SB202190+PL and SB203580+PL groups, while p38 phosphorylation expression levels were significantly reduced in the SB203580+PL group (Figures 8C,D). The above results indicate that PL may simultaneously upregulate the phosphorylation of the p38 MAPK protein and induce autophagy in HCC cells.

## Discussion

HCC is a malignant tumor with high morbidity and mortality in China and worldwide (Orcutt and Anaya, 2018 Jan-Mar). Due to its rapid proliferation and high metastasis and recurrence rates, it is not sensitive to the characteristics of conventional chemotherapy drugs and is prone to drug resistance; thus, treatment of HCC is facing a severe test. Although the current treatments for HCC include surgical resection, arterial intubation chemotherapy, radiotherapy and local treatment, the effects are not satisfactory. Moreover, substantial progress in the diagnosis and treatment of HCC has failed to greatly improve its efficacy. Therefore, international interest in finding new ways to treat HCC is also increasing. The development and application of new drugs is important for tumor treatment. The search for safe and effective anti-cancer drugs is a major research topic in the treatment of HCC. By exploring the regulation of multichannel signaling pathways, network pharmacology can improve the efficacy of drugs and the success rate of clinical trials, reducing the cost of drug discovery (Zhang et al., 2013). In this study, network pharmacology results from the network databases revealed core gene targets, and we explored the biological functions, pathways, and mechanisms of PL in HCC. The potential targets and signaling pathways of PL acting on HCC were further explored and verified through *in vitro* experiments. These findings indicate that PL is a promising resource with specific therapeutic effects on HCC.

In various human cancer cell lines, ROS is involved in autophagy, apoptosis and cell cycle arrest (Zou et al., 2017). Studies have shown that c-Jun induced by ROS is activated and regulated in HeLa cells, leading to apoptosis (Lo and Wang, 2013). In breast cancer MDA-MB-231 and MCF-7 cells, ROS-induced JNK activation induces apoptosis through mitochondrial membrane depolarization (Lebelo et al., 2020). In addition, antioxidant N-acetylcysteine (NAC) restored the depleted GSH contents of the new polyphenol conjugate DPP-23 in pancreatic cancer MIAPaCa-2 cells, further confirming that apoptosis and oxidation induced by DPP-23 stress are closely related. These findings also show that oxidative stress is an upstream event that induces apoptosis (Shin et al., 2014). Studies have shown that the anticancer effects of PL are mainly related to ROS generation, mitochondrial function, apoptosis and autophagy and related signaling pathways (Zhou et al., 2015). In our study, GO analysis results showed that the biological functions affected by the common targets of PL and HCC are mainly related to oxidative stress and ROS generation, and KEGG pathway analysis showed that the targets are mainly concentrated in the PI3K/Akt/and mTOR/MAPK signaling pathways. Our *in vitro* cell experiments confirmed that PL can mediate the generation of ROS and promote apoptosis. In addition, the phosphorylation levels of PI3K, Akt and mTOR proteins in the PI3K/Akt signaling pathway were downregulated. Our study also found that PL mainly mediates apoptosis through the mitochondrial apoptotic pathway. These results indicate that PL-induced apoptosis of human liver cancer cells is closely related to the increase in intracellular ROS, and ROS may be an upstream factor that regulates the PI3K/Akt/mTOR pathway. The interaction between PL-induced autophagy and apoptosis varies with different cell types, external stimuli and environments. Some studies have suggested that autophagy is a potential partner, antagonist or promoter of apoptosis. Our study found that the autophagy inhibitor 3-MA can attenuate PL-induced apoptosis of HCC cells. Therefore, we speculate that PL-induced autophagy can enhance apoptosis to a certain extent, and when autophagy is weakened by autophagy inhibitors, apoptosis will also be affected.

The p38 MAPK signaling pathway is thought to regulate autophagy. p38 mitogen-activated protein kinase (p38 MAPK) is a well-known kinase that is phosphorylated and participates in amino acid signal transduction in bacterial lipopolysaccharide, heat shock, osmotic stress and other environmental stress responses (Webber, 2010; Gupta et al., 2014). Recent studies have shown interaction between the p38α type of p38 MAPK and a new ATG9 binding partner, p38IP, to control the level of starvation-induced autophagy (Webber and Tooze, 2010). p38 MAPK has a dual role in autophagy: it is both a positive and a negative regulator. Importantly, phosphorylation of p38α leads to increased expression of the autophagy protein marker LC3 (Moruno-Manchón et al., 2013). Our study found that consistency between PL-induced autophagy in HCC cells and the positive effect of p38α in autophagy control. Overall, PL acts on SMMC-7721 and BEL-7404 cells to increase the phosphorylation level of p38α, thereby inducing autophagy.

Studies have also shown that the pyridinimidazole compound SB203580 is an inhibitor of p38 MAPK. It can inhibit the expression of p38 and can induce autophagy when acting on colorectal cancer cells. Prolonged inhibition can lead to cell death with autophagy characteristics (Lim et al., 2006). Our research also found that after co-intervention of the p38 MAPK inhibitors SB203580 and SB202190 with PL, the expression levels of LC3 increased, indicating that the level of autophagy increased. In addition, expression of p-p38/p38 was significantly reduced. These results indicate that using p38 MAPK inhibitors to reduce the phosphorylation levels of p38 can also induce autophagy, suggesting that this may be a potential mechanism that limits the degree of autophagy that cells can withstand and that p38α activity may determine the balance between cell survival and cell death during cell stress. As autophagy is becoming an important factor affecting many neurodegenerative diseases and cancers, p38 in the p38 MAPK signaling pathway may become a clinical target for autophagy control.

## Conclusion

In conclusion, this study identified key targets of effects of PL against HCC through network pharmacology. *In vitro* experiments were conducted to verify the effective molecular mechanism by which PL targets HCC. This study shows that PL can promote apoptosis and induce autophagy through ROS-mediated PI3K/Akt and mTOR/MAPK signaling pathways and suggests that PL may exert anti-HCC effects through multiple targets and signaling pathways. This study also demonstrates that network pharmacology is of great significance for target screening and pathway prediction of drugs and diseases.

## Data Availability

The raw data supporting the conclusions of this article will be made available by the authors, without undue reservation.
