# Supplementary material for: Computational and In Vitro Analysis of Plumbagin’s Molecular Mechanism for the Treatment of Hepatocellular Carcinoma
Source: Front Pharmacol. 2021 Apr 12;12:594833. doi: 10.3389/fphar.2021.594833 (PMC8072012; doi:10.3389/fphar.2021.594833)
Supplement: Supplementary file 1 [file datasheet1.doc]

**Supplemental Table 1：**

**Table 1. Drug target of compound PL**

| Gene name | Gene symbol | Uniprot ID |
| --- | --- | --- |
| Histone acetyltransferase p300 | EP300 | Q09472 |
| Indoleamine 2,3-dioxygenase | IDO1 | P14902 |
| Dual specificity phosphatase Cdc25B | CDC25B | P30305 |
| Serine/threonine-protein kinase PIM1 | PIM1 | P11309 |
| Dual specificity protein phosphatase 1 | DUSP1 | P28562 |
| Monoamine oxidase B | MAOB | P27338 |
| Glutathione reductase | GSR | P00390 |
| Serine/threonine-protein kinase/endoribonuclease IRE1 | ERN1 | O75460 |
| Myoglobin | MB | P02144 |
| C-X-C chemokine receptor type 4 | CXCR4 | P61073 |
| RAC-alpha serine/threonine-protein | AKT1 | P31749 |
| Mitogen-activated protein kinase 8 | MAPK8 | P45983 |
| Superoxide dismutase [Cu-Zn] | SOD1 | P00441 |
| Transcription factor NF-E2 | NFE2 | Q16621 |
| Solute carrier family 2 | SLC2A4 | P14672 |
| Dihydroorotate dehydrogenase (quinone) | DHODH | Q02127 |
| Proto-oncogene tyrosine-protein kinase | SRC | P12931 |
| Cyclin-dependent kinase inhibitor 1 | CDKN1A | P38936 |
| DNA topoisomerase I | TOP1 | P11387 |
| RAF proto-oncogene serine/threonine-protein kinase | RAF1 | P04049 |
| Tumour suppressor p53/oncoprotein Mdm2 | TP53 | P04637 |
| Vitamin D receptor | VDR | P11473 |
| 15-hydroxyprostaglandin dehydrogenase [NAD+] | HPGD | P15428 |
| 78 kDa glucose-regulated protein | HSPA5 | P11021 |
| Aldehyde dehydrogenase 1A1 | ALDH1A1 | P00352 |
| Arachidonate 15-lipoxygenase | ALOX15 | P16050 |
| ATP-dependent DNA helicase Q1 | RECQL | P46063 |
| Bloom syndrome protein | BLM | P54132 |
| Caspase-1 | CASP1 | P29466 |
| Caspase-7 | CASP7 | P55210 |
| DNA polymerase kappa | POLK | Q9UBT6 |
| Dual specificity mitogen-activated protein kinase kinase | MAP2K1 | Q02750 |
| Endoplasmic reticulum-associated amyloid beta-peptide-binding protein | HSD17B10 | Q99714 |
| Histone acetyltransferase GCN5 | KAT2A | Q92830 |
| Lysine-specific demethylase 4D-like | KDM4E | B2RXH2 |
| Microtubule-associated protein tau | MAPT | P10636 |
| Mitogen-activated protein kinase kinase kinase 14 | MAP3K14 | Q99558 |
| Mitogen-activated protein kinase; ERK1/ERK2 | MAPK1 | P28482 |
| Nuclear factor NF-kappa-B p65 subunit | RELA | Q04206 |
| Survival motor neuron protein | SMN1 | Q16637 |
| Tyrosyl-DNA phosphodiesterase 1 | TDP1 | Q9NUW8 |
| Ubiquitin carboxyl-terminal hydrolase 1 | USP1 | O94782 |
| Transcription factor AP-1 | JUN | P05412 |
| Transcription factor jun-D | JUND | P17535 |
| Estrogen receptor, ER | ESR1 | P03372 |
| Dual specificity mitogen-activated protein kinase kinase 4 | MAP2K4 | P45985 |
| C-Jun-amino-terminal kinase-interacting protein 1 | MAPK8IP1 | Q9UQF2 |
| Mitogen-activated protein kinase 14A | MAPK14A | Q9DGE2 |
| Signal transducer and activator of transcription 3 | STAT3 | P40763 |
| Tyrosine-protein phosphatase non-receptor type 1 | PTPN1 | P18031 |
| E3 ubiquitin-protein ligase CBL | CBL | P22681 |
| Nuclear receptor subfamily 0 group B member 2 | NR0B2 | Q15466 |
| E3 ubiquitin-protein ligase Mdm2 | MDM2 | Q00987 |
| Serine/threonine-protein kinase mTOR | MTOR | P42345 |
| Heat shock protein HSP 90-alpha | HSP90AA1 | P07900 |
| Cyclin-dependent kinase 2 | CDK2 | P24941 |
| S-phase kinase-associated protein 2 | SKP2 | Q13309 |
| E3 ubiquitin-protein ligase CCNB1IP1 | CCNB1 | Q9NPC3 |
| G1/S-specific cyclin-E1 | CCNE1 | P24864 |
| G1/S-specific cyclin-D3 | CCND3 | P30281 |
| Cyclin-dependent kinase 4 | CDK4 | P11802 |
| Glycogen synthase kinase-3 beta | GSK3B | P49841 |
| Cyclic AMP-dependent transcription factor ATF-2 | ATF2 | P15336 |
| Integrin-linked protein kinase | ILK | Q13418 |
| Rapamycin-insensitive companion of mTOR | RICTOR | Q6R327 |
| Nitric oxide synthase, endothelial | NOS3 | P29474 |
| Zinc finger protein Gfi-1 | CDKNIA | Q99684 |
| PCNA-associated factor | PCNA | Q15004 |
| Forkhead box protein O3 | FOXO3 | O43524 |
| Forkhead box protein O1 | FOXO1 | Q12778 |
| Breast cancer anti-estrogen resistance protein 1 | BCAR1 | P56945 |
| Beta-secretase 1 | BACE1 | P56817 |
| TNF receptor-associated factor 2 | TRAF | Q12933 |
| Baculoviral IAP repeat-containing protein 3 | BIRC3 | Q13489 |
| Interleukin-6 | IL6 | P05231 |
| Phosphatidylinositol 3-kinase regulatory subunit alpha | PIK3R1 | P27986 |
| Bcl2-associated agonist of cell death | BAD | Q92934 |
| Histone-lysine N-methyltransferase 2A | KMT2A | Q03164 |
| Interleukin-4 | IL4 | P05112 |
| Early activation antigen CD69 | CD69 | Q07108 |
| M-phase inducer phosphatase 1 | CDC25A | P30304 |
| CASP8 and FADD-like apoptosis regulator | CFLAR | O15519 |
| Matrix metalloproteinase-9 | MMP9 | P14780 |
| Prostaglandin G/H synthase 2 | PTGS2 | P35354 |
| DNA-(apurinic or apyrimidinic site) lyase | APEX1 | P27695 |
| Perilipin-1 | PLIN1 | O60240 |
| Muscleblind-like protein 1 | MBNL1 | Q9NR56 |
| Muscleblind-like protein 3 | MBNL3 | Q9NUK0 |
| Interleukin-2 | IL2 | P60568 |
| Interleukin-2 receptor subunit alpha | IL2RA | P01589 |
| Cytochrome P450 1A2 | CYP1A2 | P05177 |
| Ubiquinol oxidase 1a | AOX1 | Q39219 |
| Cytochrome P450 3A4 | CYP3A4 | P08684 |
| Cytochrome P450 2C9 | CYP2C9 | P11712 |
| Cytochrome P450 2D6 | CYP2D6 | P10635 |
| Histone acetyltransferase KAT2A | KAT2A | Q92830 |
| Cytochrome P450 2C19 | CYR2C19 | P33261 |
| Interferon gamma | IFNG | P01579 |
| NAD(P)H dehydrogenase [quinone] 1 | NQO1 | P15559 |

**Supplemental Table 2：**

**Table 2. Potential targets for HCC**

| Gene Symbol | Gene name | GC Id |
| --- | --- | --- |
| TP53 | Tumor Protein P53 | GC17M007661 |
| MET | MET Proto-Oncogene, Receptor Tyrosine Kinase | GC07P116672 |
| CTNNB1 | Catenin Beta 1 | GC03P041236 |
| PIK3CA | Phosphatidylinositol-4,5-Bisphosphate 3-Kinase Catalytic Subunit Alpha | GC03P179148 |
| CDKN2A | Cyclin Dependent Kinase Inhibitor 2A | GC09M021967 |
| APC | APC Regulator Of WNT Signaling Pathway | GC05P112707 |
| EGFR | Epidermal Growth Factor Receptor | GC07P055019 |
| PTEN | Phosphatase And Tensin Homolog | GC10P087863 |
| CDH1 | Cadherin 1 | GC16P068737 |
| HRAS | HRas Proto-Oncogene, GTPase | GC11M000522 |
| AKT1 | AKT Serine/Threonine Kinase 1 | GC14M104769 |
| ERBB2 | Erb-B2 Receptor Tyrosine Kinase 2 | GC17P039687 |
| KRAS | KRAS Proto-Oncogene, GTPase | GC12M025204 |
| CCND1 | Cyclin D1 | GC11P069641 |
| BRAF | B-Raf Proto-Oncogene, Serine/Threonine Kinase | GC07M140719 |
| TERT | Telomerase Reverse Transcriptase | GC05M001253 |
| CDKN1A | Cyclin Dependent Kinase Inhibitor 1A | GC06P046057 |
| TGFBR2 | Transforming Growth Factor Beta Receptor 2 | GC03P030623 |
| MSH2 | MutS Homolog 2 | GC02P047402 |
| SMAD4 | SMAD Family Member 4 | GC18P051028 |
| MTOR | Mechanistic Target Of Rapamycin Kinase | GC01M011106 |
| VEGFA | Vascular Endothelial Growth Factor A | GC06P043770 |
| FAS | Fas Cell Surface Death Receptor | GC10P088969 |
| BAX | BCL2 Associated X, Apoptosis Regulator | GC19P048954 |
| CASP8 | Caspase 8 | GC02P201233 |
| NRAS | NRAS Proto-Oncogene, GTPase | GC01M114704 |
| CDKN1B | Cyclin Dependent Kinase Inhibitor 1B | GC12P012716 |
| EGF | Epidermal Growth Factor | GC04P109912 |
| RB1 | RB Transcriptional Corepressor 1 | GC13P048303 |
| ESR1 | Estrogen Receptor 1 | GC06P151656 |
| MLH1 | MutL Homolog 1 | GC03P036993 |
| EPCAM | Epithelial Cell Adhesion Molecule | GC02P047345 |
| NFE2L2 | Nuclear Factor, Erythroid 2 Like 2 | GC02M177227 |
| RET | Ret Proto-Oncogene | GC10P043081 |
| FASLG | Fas Ligand | GC01P172628 |
| HNF1A | HNF1 Homeobox A | GC12P120978 |
| FHIT | Fragile Histidine Triad Diadenosine Triphosphatase | GC03M059747 |
| KIT | KIT Proto-Oncogene, Receptor Tyrosine Kinase | GC04P054657 |
| MDM2 | MDM2 Proto-Oncogene | GC12P068808 |
| H19 | H19 Imprinted Maternally Expressed Transcript | GC11M001995 |
| MIR21 | MicroRNA 21 | GC17P059841 |
| IGF2 | Insulin Like Growth Factor 2 | GC11M002130 |
| AXIN1 | Axin 1 | GC16M000287 |
| FGFR1 | Fibroblast Growth Factor Receptor 1 | GC08M038400 |
| PTCH1 | Patched 1 | GC09M095442 |
| CDKN3 | Cyclin Dependent Kinase Inhibitor 3 | GC14P054398 |
| STK11 | Serine/Threonine Kinase 11 | GC19P001177 |
| MYC | MYC Proto-Oncogene, BHLH Transcription Factor | GC08P127735 |
| BRCA1 | BRCA1 DNA Repair Associated | GC17M043044 |
| MMP1 | Matrix Metallopeptidase 1 | GC11M102810 |
| STAT3 | Signal Transducer And Activator Of Transcription 3 | GC17M042313 |
| HIF1A | Hypoxia Inducible Factor 1 Subunit Alpha | GC14P061695 |
| SRC | SRC Proto-Oncogene, Non-Receptor Tyrosine Kinase | GC20P037344 |
| AFP | Alpha Fetoprotein | GC04P073431 |
| PTGS2 | Prostaglandin-Endoperoxide Synthase 2 | GC01M186640 |
| BCL2 | BCL2 Apoptosis Regulator | GC18M063123 |
| KRT7 | Keratin 7 | GC12P052232 |
| RAF1 | Raf-1 Proto-Oncogene, Serine/Threonine Kinase | GC03M012583 |
| BIRC5 | Baculoviral IAP Repeat Containing 5 | GC17P078214 |
| TGFB1 | Transforming Growth Factor Beta 1 | GC19M041301 |
| FGFR3 | Fibroblast Growth Factor Receptor 3 | GC04P001795 |
| IGF2R | Insulin Like Growth Factor 2 Receptor | GC06P159969 |
| MMP9 | Matrix Metallopeptidase 9 | GC20P046008 |
| BRCA2 | BRCA2 DNA Repair Associated | GC13P032315 |
| MIR145 | MicroRNA 145 | GC05P149430 |
| TNFRSF10B | TNF Receptor Superfamily Member 10b | GC08M023006 |
| AR | Androgen Receptor | GC0XP067544 |
| KRT19 | Keratin 19 | GC17M041523 |
| TWIST1 | Twist Family BHLH Transcription Factor 1 | GC07M019020 |
| MIR34A | MicroRNA 34a | GC01M009151 |
| MIR17 | MicroRNA 17 | GC13P091350 |
| TP73 | Tumor Protein P73 | GC01P003652 |
| CDKN2B | Cyclin Dependent Kinase Inhibitor 2B | GC09M022002 |
| MIR200A | MicroRNA 200a | GC01P001296 |
| MMP2 | Matrix Metallopeptidase 2 | GC16P055390 |
| MIR221 | MicroRNA 221 | GC0XM045746 |
| MSH6 | MutS Homolog 6 | GC02P047695 |
| FGFR2 | Fibroblast Growth Factor Receptor 2 | GC10M121478 |
| IL6 | Interleukin 6 | GC07P022765 |
| MUC1 | Mucin 1, Cell Surface Associated | GC01M155185 |
| AURKA | Aurora Kinase A | GC20M056370 |
| CASP3 | Caspase 3 | GC04M184627 |
| IDH1 | Isocitrate Dehydrogenase (NADP(+)) 1 | GC02M208236 |
| CD44 | CD44 Molecule (Indian Blood Group) | GC11P035139 |
| FGFR4 | Fibroblast Growth Factor Receptor 4 | GC05P177086 |
| TGFBR1 | Transforming Growth Factor Beta Receptor 1 | GC09P099104 |
| MIR146A | MicroRNA 146a | GC05P160485 |
| GNAS | GNAS Complex Locus | GC20P058839 |
| MAP2K1 | Mitogen-Activated Protein Kinase Kinase 1 | GC15P066386 |
| MIR195 | MicroRNA 195 | GC17M007018 |
| MIR155 | MicroRNA 155 | GC21P025573 |
| MIR27A | MicroRNA 27a | GC19M013987 |
| MIR143 | MicroRNA 143 | GC05P149410 |
| MKI67 | Marker Of Proliferation Ki-67 | GC10M128096 |
| MIR141 | MicroRNA 141 | GC12P007879 |
| SMARCA4 | SWI/SNF Related, Matrix Associated, Actin Dependent Regulator Of Chromatin, Subfamily A, Member 4 | GC19P010932 |
| SMARCB1 | SWI/SNF Related, Matrix Associated, Actin Dependent Regulator Of Chromatin, Subfamily B, Member 1 | GC22P023786 |
| MIR31 | MicroRNA 31 | GC09M021513 |
| HFE | Homeostatic Iron Regulator | GC06P026087 |
| MIR122 | MicroRNA 122 | GC18P058451 |
| MIR200C | MicroRNA 200c | GC12P007878 |
| MAPK1 | Mitogen-Activated Protein Kinase 1 | GC22M021754 |
| TERC | Telomerase RNA Component | GC03M169765 |
| OGG1 | 8-Oxoguanine DNA Glycosylase | GC03P009751 |
| JAG1 | Jagged Canonical Notch Ligand 1 | GC20M010637 |
| KEAP1 | Kelch Like ECH Associated Protein 1 | GC19M010486 |
| GSTP1 | Glutathione S-Transferase Pi 1 | GC11P067583 |
| MIR125A | MicroRNA 125a | GC19P051723 |
| KLF6 | Kruppel Like Factor 6 | GC10M003779 |
| MIR126 | MicroRNA 126 | GC09P136670 |
| CHEK2 | Checkpoint Kinase 2 | GC22M028687 |
| MIR214 | MicroRNA 214 | GC01M172229 |
| MTUS1 | Microtubule Associated Scaffold Protein 1 | GC08M017643 |
| MIR150 | MicroRNA 150 | GC19M049500 |
| MIR205 | MicroRNA 205 | GC01P209432 |
| MIR203A | MicroRNA 203a | GC14P104331 |
| MIR200B | MicroRNA 200b | GC01P001167 |
| TNF | Tumor Necrosis Factor | GC06P033397 |
| JUN | Jun Proto-Oncogene, AP-1 Transcription Factor Subunit | GC01M058780 |
| BCL2L1 | BCL2 Like 1 | GC20M031664 |
| CXCL8 | C-X-C Motif Chemokine Ligand 8 | GC04P073740 |
| MIR106B | MicroRNA 106b | GC07M100247 |
| IDH2 | Isocitrate Dehydrogenase (NADP(+)) 2 | GC15M090083 |
| AXIN2 | Axin 2 | GC17M065528 |
| ATM | ATM Serine/Threonine Kinase | GC11P108222 |
| FN1 | Fibronectin 1 | GC02M215360 |
| KDR | Kinase Insert Domain Receptor | GC04M055078 |
| HGF | Hepatocyte Growth Factor | GC07M081699 |
| CD274 | CD274 Molecule | GC09P005450 |
| HNF4A | Hepatocyte Nuclear Factor 4 Alpha | GC20P044355 |
| MIR148A | MicroRNA 148a | GC07M025993 |
| NOTCH1 | Notch Receptor 1 | GC09M136582 |
| MIR22 | MicroRNA 22 | GC17M001713 |
| PPARG | Peroxisome Proliferator Activated Receptor Gamma | GC03P012287 |
| PTPN11 | Protein Tyrosine Phosphatase Non-Receptor Type 11 | GC12P112418 |
| MIR222 | MicroRNA 222 | GC0XM045747 |
| TGFA | Transforming Growth Factor Alpha | GC02M070447 |
| VIM | Vimentin | GC10P017227 |
| MIR26A1 | MicroRNA 26a-1 | GC03P037969 |
| MIR223 | MicroRNA 223 | GC0XP066018 |
| MIR193A | MicroRNA 193a | GC17P031559 |
| EP300 | E1A Binding Protein P300 | GC22P041091 |
| MIR30E | MicroRNA 30e | GC01P040754 |
| GPC3 | Glypican 3 | GC0XM133535 |
| MIR10B | MicroRNA 10b | GC02P176150 |
| DLC1 | DLC1 Rho GTPase Activating Protein | GC08M013083 |
| MIR204 | MicroRNA 204 | GC09M070809 |
| MIR183 | MicroRNA 183 | GC07M129788 |
| MIRLET7A1 | MicroRNA Let-7a-1 | GC09P094175 |
| RASSF1 | Ras Association Domain Family Member 1 | GC03M050329 |
| MIR483 | MicroRNA 483 | GC11M002178 |
| IFNG | Interferon Gamma | GC12M068064 |
| NFKBIA | NFKB Inhibitor Alpha | GC14M035401 |
| MIR182 | MicroRNA 182 | GC07M129770 |
| ABCB1 | ATP Binding Cassette Subfamily B Member 1 | GC07M087504 |
| FLCN | Folliculin | GC17M017212 |
| MIR18A | MicroRNA 18a | GC13P091422 |
| TSC2 | TSC Complex Subunit 2 | GC16P002385 |
| MIR29A | MicroRNA 29a | GC07M130876 |
| SERPINA1 | Serpin Family A Member 1 | GC14M094376 |
| GSTM1 | Glutathione S-Transferase Mu 1 | GC01P109687 |
| CDK4 | Cyclin Dependent Kinase 4 | GC12M057743 |
| CASP9 | Caspase 9 | GC01M015491 |
| PMS2 | PMS1 Homolog 2, Mismatch Repair System Component | GC07M005973 |
| NFKB1 | Nuclear Factor Kappa B Subunit 1 | GC04P102501 |
| KRT8 | Keratin 8 | GC12M052897 |
| MIR185 | MicroRNA 185 | GC22P020034 |
| MIR23A | MicroRNA 23a | GC19M013988 |
| CEACAM5 | CEA Cell Adhesion Molecule 5 | GC19P041709 |
| KRT18 | Keratin 18 | GC12P052948 |
| IFNA1 | Interferon Alpha 1 | GC09P021478 |
| KCNQ1OT1 | KCNQ1 Opposite Strand/Antisense Transcript 1 | GC11M002661 |
| NME1 | NME/NM23 Nucleoside Diphosphate Kinase 1 | GC17P051154 |
| SPRTN | SprT-Like N-Terminal Domain | GC01P231337 |
| MIR181A1 | MicroRNA 181a-1 | GC01M198860 |
| IL2 | Interleukin 2 | GC04M122451 |
| VHL | Von Hippel-Lindau Tumor Suppressor | GC03P010205 |
| TCF4 | Transcription Factor 4 | GC18M055222 |
| MIR140 | MicroRNA 140 | GC16P069934 |
| IGF1R | Insulin Like Growth Factor 1 Receptor | GC15P098648 |
| TSC1 | TSC Complex Subunit 1 | GC09M132891 |
| MAPK8 | Mitogen-Activated Protein Kinase 8 | GC10P048306 |
| MIR20A | MicroRNA 20a | GC13P091427 |
| MIR93 | MicroRNA 93 | GC07M100246 |
| SPP1 | Secreted Phosphoprotein 1 | GC04P087975 |
| FGF2 | Fibroblast Growth Factor 2 | GC04P122826 |
| MIR192 | MicroRNA 192 | GC11M064891 |
| TNFSF10 | TNF Superfamily Member 10 | GC03M172505 |
| MIR16-1 | MicroRNA 16-1 | GC13M050048 |
| MAPK3 | Mitogen-Activated Protein Kinase 3 | GC16M030117 |
| MIR23B | MicroRNA 23b | GC09P095085 |
| MIR191 | MicroRNA 191 | GC03M049202 |
| FAH | Fumarylacetoacetate Hydrolase | GC15P080152 |
| MIR34C | MicroRNA 34c | GC11P111579 |
| MIR15B | MicroRNA 15b | GC03P160404 |
| MIR224 | MicroRNA 224 | GC0XM151958 |
| HNF1B | HNF1 Homeobox B | GC17M037686 |
| MIR96 | MicroRNA 96 | GC07M129774 |
| VEGFC | Vascular Endothelial Growth Factor C | GC04M176683 |
| MIRLET7G | MicroRNA Let-7g | GC03M052268 |
| ALB | Albumin | GC04P073397 |
| EZH2 | Enhancer Of Zeste 2 Polycomb Repressive Complex 2 Subunit | GC07M148807 |
| MIR15A | MicroRNA 15a | GC13M050049 |
| SLC2A1 | Solute Carrier Family 2 Member 1 | GC01M042925 |
| CXCR4 | C-X-C Motif Chemokine Receptor 4 | GC02M136114 |
| MIR107 | MicroRNA 107 | GC10M089601 |
| CDKN1C | Cyclin Dependent Kinase Inhibitor 1C | GC11M002886 |
| MIR19A | MicroRNA 19a | GC13P091426 |
| MIR25 | MicroRNA 25 | GC07M100093 |
| CYP1A1 | Cytochrome P450 Family 1 Subfamily A Member 1 | GC15M074719 |
| PTK2 | Protein Tyrosine Kinase 2 | GC08M140657 |
| SYP | Synaptophysin | GC0XM049187 |
| TYMP | Thymidine Phosphorylase | GC22M050525 |
| NKX2-1 | NK2 Homeobox 1 | GC14M036516 |
| PDGFRL | Platelet Derived Growth Factor Receptor Like | GC08P017576 |
| MIR127 | MicroRNA 127 | GC14P104252 |
| ERBB3 | Erb-B2 Receptor Tyrosine Kinase 3 | GC12P056086 |
| PLAU | Plasminogen Activator, Urokinase | GC10P073909 |
| G6PC | Glucose-6-Phosphatase Catalytic Subunit | GC17P042900 |
| RARB | Retinoic Acid Receptor Beta | GC03P025194 |
| NR1H4 | Nuclear Receptor Subfamily 1 Group H Member 4 | GC12P100473 |
| IL10 | Interleukin 10 | GC01M206767 |
| MIR199B | MicroRNA 199b | GC09M128244 |
| CREBBP | CREB Binding Protein | GC16M003726 |
| MIRLET7D | MicroRNA Let-7d | GC09P094178 |
| MMP7 | Matrix Metallopeptidase 7 | GC11M102425 |
| PARP1 | Poly(ADP-Ribose) Polymerase 1 | GC01M226360 |
| TIMP2 | TIMP Metallopeptidase Inhibitor 2 | GC17M078852 |
| CCNA2 | Cyclin A2 | GC04M121816 |
| FLT1 | Fms Related Receptor Tyrosine Kinase 1 | GC13M028300 |
| ABCB4 | ATP Binding Cassette Subfamily B Member 4 | GC07M087401 |
| MIRLET7B | MicroRNA Let-7b | GC22P046120 |
| CDK2 | Cyclin Dependent Kinase 2 | GC12P055966 |
| CHGA | Chromogranin A | GC14P092923 |
| BSG | Basigin (Ok Blood Group) | GC19P000571 |
| INS | Insulin | GC11M002159 |
| RAC1 | Rac Family Small GTPase 1 | GC07P006380 |
| PCNA | Proliferating Cell Nuclear Antigen | GC20M005114 |
| MAPK14 | Mitogen-Activated Protein Kinase 14 | GC06P046047 |
| PEG10 | Paternally Expressed 10 | GC07P094656 |
| MIR196A2 | MicroRNA 196a-2 | GC12P054167 |
| JAK2 | Janus Kinase 2 | GC09P004985 |
| MGMT | O-6-Methylguanine-DNA Methyltransferase | GC10P129467 |
| SMAD2 | SMAD Family Member 2 | GC18M047809 |
| CAV1 | Caveolin 1 | GC07P116524 |
| MAGEA1 | MAGE Family Member A1 | GC0XP153179 |
| PRKAR1A | Protein Kinase CAMP-Dependent Type I Regulatory Subunit Alpha | GC17P068414 |
| MUTYH | MutY DNA Glycosylase | GC01M045329 |
| HULC | Hepatocellular Carcinoma Up-Regulated Long Non-Coding RNA | GC06P008438 |
| MIRLET7C | MicroRNA Let-7c | GC21P016553 |
| MIR324 | MicroRNA 324 | GC17M007223 |
| MIR137 | MicroRNA 137 | GC01M098046 |
| SMAD3 | SMAD Family Member 3 | GC15P067063 |
| MMP14 | Matrix Metallopeptidase 14 | GC14P025005 |
| CXCL12 | C-X-C Motif Chemokine Ligand 12 | GC10M044370 |
| MEN1 | Menin 1 | GC11M064803 |
| DNMT1 | DNA Methyltransferase 1 | GC19M010133 |
| SDHB | Succinate Dehydrogenase Complex Iron Sulfur Subunit B | GC01M017020 |
| MIRLET7E | MicroRNA Let-7e | GC19P051722 |
| IGF1 | Insulin Like Growth Factor 1 | GC12M102395 |
| CYCS | Cytochrome C, Somatic | GC07M025118 |
| SERPINB3 | Serpin Family B Member 3 | GC18M063655 |
| YAP1 | Yes Associated Protein 1 | GC11P102110 |
| MAGEA3 | MAGE Family Member A3 | GC0XP152698 |
| CCNB1 | Cyclin B1 | GC05P069167 |
| PLAUR | Plasminogen Activator, Urokinase Receptor | GC19M043646 |
| XRCC1 | X-Ray Repair Cross Complementing 1 | GC19M043543 |
| SNAI1 | Snail Family Transcriptional Repressor 1 | GC20P049982 |
| MIR128-2 | MicroRNA 128-2 | GC03P035751 |
| TLR2 | Toll Like Receptor 2 | GC04P153684 |
| IFI27 | Interferon Alpha Inducible Protein 27 | GC14P094104 |
| ABCC1 | ATP Binding Cassette Subfamily C Member 1 | GC16P015949 |
| CDH2 | Cadherin 2 | GC18M027950 |
| TIMP1 | TIMP Metallopeptidase Inhibitor 1 | GC0XP047583 |
| E2F1 | E2F Transcription Factor 1 | GC20M033675 |
| MIR29C | MicroRNA 29c | GC01M207802 |
| HOTAIR | HOX Transcript Antisense RNA | GC12M053962 |
| HSPB1 | Heat Shock Protein Family B (Small) Member 1 | GC07P076302 |
| XRCC3 | X-Ray Repair Cross Complementing 3 | GC14M103697 |
| MIR24-2 | MicroRNA 24-2 | GC19M013986 |
| RHOA | Ras Homolog Family Member A | GC03M049359 |
| IL1B | Interleukin 1 Beta | GC02M112829 |
| MALAT1 | Metastasis Associated Lung Adenocarcinoma Transcript 1 | GC11P065621 |
| XIAP | X-Linked Inhibitor Of Apoptosis | GC0XP123859 |
| HTATIP2 | HIV-1 Tat Interactive Protein 2 | GC11P020363 |
| IL12RB1 | Interleukin 12 Receptor Subunit Beta 1 | GC19M018030 |
| MIR152 | MicroRNA 152 | GC17M048037 |
| MIR130A | MicroRNA 130a | GC11P057641 |
| IFNA2 | Interferon Alpha 2 | GC09M021374 |
| MME | Membrane Metalloendopeptidase | GC03P155024 |
| MIR181B1 | MicroRNA 181b-1 | GC01M198858 |
| SNAI2 | Snail Family Transcriptional Repressor 2 | GC08M048854 |
| BMP2 | Bone Morphogenetic Protein 2 | GC20P006696 |
| MCL1 | MCL1 Apoptosis Regulator, BCL2 Family Member | GC01M150673 |
| TIMP3 | TIMP Metallopeptidase Inhibitor 3 | GC22P032800 |
| MIRLET7A3 | MicroRNA Let-7a-3 | GC22P046112 |
| IFNL3 | Interferon Lambda 3 | GC19M039243 |
| NOS2 | Nitric Oxide Synthase 2 | GC17M027756 |
| POU5F1 | POU Class 5 Homeobox 1 | GC06M031177 |
| MIR181A2 | MicroRNA 181a-2 | GC09P124692 |
| CTTN | Cortactin | GC11P070398 |
| NQO1 | NAD(P)H Quinone Dehydrogenase 1 | GC16M069706 |
| CDK6 | Cyclin Dependent Kinase 6 | GC07M092604 |
| ITGB1 | Integrin Subunit Beta 1 | GC10M032900 |
| CALCA | Calcitonin Related Polypeptide Alpha | GC11M014945 |
| F2 | Coagulation Factor II, Thrombin | GC11P046720 |
| PRKACA | Protein Kinase CAMP-Activated Catalytic Subunit Alpha | GC19M014092 |
| CDK1 | Cyclin Dependent Kinase 1 | GC10P060772 |
| SP1 | Sp1 Transcription Factor | GC12P053380 |
| ZEB1 | Zinc Finger E-Box Binding Homeobox 1 | GC10P031318 |
| MIR199A1 | MicroRNA 199a-1 | GC19M010792 |
| CFLAR | CASP8 And FADD Like Apoptosis Regulator | GC02P201117 |
| BMPR1A | Bone Morphogenetic Protein Receptor Type 1A | GC10P086756 |
| ABCB11 | ATP Binding Cassette Subfamily B Member 11 | GC02M168922 |
| ANXA2 | Annexin A2 | GC15M060347 |
| PGR | Progesterone Receptor | GC11M100943 |
| TP63 | Tumor Protein P63 | GC03P189566 |
| GSK3B | Glycogen Synthase Kinase 3 Beta | GC03M119821 |
| ANXA5 | Annexin A5 | GC04M121667 |
| ABCG2 | ATP Binding Cassette Subfamily G Member 2 (Junior Blood Group) | GC04M088090 |
| PROM1 | Prominin 1 | GC04M015965 |
| FOXM1 | Forkhead Box M1 | GC12M002857 |
| SKP2 | S-Phase Kinase Associated Protein 2 | GC05P036103 |
| WRAP53 | WD Repeat Containing Antisense To TP53 | GC17P007950 |
| DNMT3B | DNA Methyltransferase 3 Beta | GC20P032762 |
| DPYD | Dihydropyrimidine Dehydrogenase | GC01M097015 |
| SOD2 | Superoxide Dismutase 2 | GC06M159669 |
| AREG | Amphiregulin | GC04P074445 |
| KRT20 | Keratin 20 | GC17M040875 |
| HSPA5 | Heat Shock Protein Family A (Hsp70) Member 5 | GC09M125234 |
| MIR215 | MicroRNA 215 | GC01M220117 |
| ARID1A | AT-Rich Interaction Domain 1A | GC01P026706 |
| SPARC | Secreted Protein Acidic And Cysteine Rich | GC05M151639 |
| HEPN1 | Hepatocellular Carcinoma, Down-Regulated 1 | GC11P124919 |
| JAK1 | Janus Kinase 1 | GC01M064833 |
| RXRA | Retinoid X Receptor Alpha | GC09P134317 |
| UCA1 | Urothelial Cancer Associated 1 | GC19P015828 |
| MLH3 | MutL Homolog 3 | GC14M075013 |
| TNFRSF10A | TNF Receptor Superfamily Member 10a | GC08M023190 |
| EPAS1 | Endothelial PAS Domain Protein 1 | GC02P046293 |
| ENG | Endoglin | GC09M127815 |
| FOS | Fos Proto-Oncogene, AP-1 Transcription Factor Subunit | GC14P075278 |
| PIK3CG | Phosphatidylinositol-4,5-Bisphosphate 3-Kinase Catalytic Subunit Gamma | GC07P106865 |
| HPSE | Heparanase | GC04M083292 |
| RUNX3 | RUNX Family Transcription Factor 3 | GC01M024899 |
| PIK3R1 | Phosphoinositide-3-Kinase Regulatory Subunit 1 | GC05P068215 |
| BAK1 | BCL2 Antagonist/Killer 1 | GC06M033572 |
| PKM | Pyruvate Kinase M1/2 | GC15M072199 |
| MIR139 | MicroRNA 139 | GC11M072615 |
| GGT1 | Gamma-Glutamyltransferase 1 | GC22P024583 |
| CCNE1 | Cyclin E1 | GC19P029811 |
| SIRT1 | Sirtuin 1 | GC10P067884 |
| EPO | Erythropoietin | GC07P100720 |
| PMS1 | PMS1 Homolog 1, Mismatch Repair System Component | GC02P189784 |
| CTAG1B | Cancer/Testis Antigen 1B | GC0XM154617 |
| MIR30A | MicroRNA 30a | GC06M071403 |
| GPT | Glutamic--Pyruvic Transaminase | GC08P144502 |
| ABCC2 | ATP Binding Cassette Subfamily C Member 2 | GC10P099782 |
| AKT2 | AKT Serine/Threonine Kinase 2 | GC19M040230 |
| BMP6 | Bone Morphogenetic Protein 6 | GC06P007726 |
| MIR181C | MicroRNA 181c | GC19P013876 |
| ICAM1 | Intercellular Adhesion Molecule 1 | GC19P010270 |
| CYP2E1 | Cytochrome P450 Family 2 Subfamily E Member 1 | GC10P133520 |
| SERPINA3 | Serpin Family A Member 3 | GC14P094612 |
| PTPRC | Protein Tyrosine Phosphatase Receptor Type C | GC01P198607 |
| TLR4 | Toll Like Receptor 4 | GC09P117704 |
| CASP7 | Caspase 7 | GC10P113679 |
| IL6R | Interleukin 6 Receptor | GC01P154405 |
| GRB2 | Growth Factor Receptor Bound Protein 2 | GC17M075318 |
| PVT1 | Pvt1 Oncogene | GC08P127805 |
| MAGEC2 | MAGE Family Member C2 | GC0XM142202 |
| PDCD1 | Programmed Cell Death 1 | GC02M241849 |
| SLC25A13 | Solute Carrier Family 25 Member 13 | GC07M096120 |
| MEG3 | Maternally Expressed 3 | GC14P104431 |
| BAD | BCL2 Associated Agonist Of Cell Death | GC11M064273 |
| STAT1 | Signal Transducer And Activator Of Transcription 1 | GC02M190964 |
| NTRK1 | Neurotrophic Receptor Tyrosine Kinase 1 | GC01P156786 |
| PRKCA | Protein Kinase C Alpha | GC17P066302 |
| HSPA4 | Heat Shock Protein Family A (Hsp70) Member 4 | GC05P133051 |
| BECN1 | Beclin 1 | GC17M042810 |
| CD82 | CD82 Molecule | GC11P044586 |
| NBN | Nibrin | GC08M089933 |
| FASN | Fatty Acid Synthase | GC17M082078 |
| WNT5A | Wnt Family Member 5A | GC03M055465 |
| ZEB2 | Zinc Finger E-Box Binding Homeobox 2 | GC02M144384 |
| TCF7L2 | Transcription Factor 7 Like 2 | GC10P112950 |
| FOXP3 | Forkhead Box P3 | GC0XM049250 |
| NF1 | Neurofibromin 1 | GC17P031007 |
| STARD13 | StAR Related Lipid Transfer Domain Containing 13 | GC13M033103 |
| BIRC3 | Baculoviral IAP Repeat Containing 3 | GC11P102317 |
| SETD2 | SET Domain Containing 2, Histone Lysine Methyltransferase | GC03M047033 |
| RPS6KB1 | Ribosomal Protein S6 Kinase B1 | GC17P059893 |
| TUG1 | Taurine Up-Regulated 1 | GC22P030969 |
| LEF1 | Lymphoid Enhancer Binding Factor 1 | GC04M108047 |
| MIR149 | MicroRNA 149 | GC02P240456 |
| WNT1 | Wnt Family Member 1 | GC12P049041 |
| KRT5 | Keratin 5 | GC12M052514 |
| AKR1B10 | Aldo-Keto Reductase Family 1 Member B10 | GC07P134527 |
| GAPDH | Glyceraldehyde-3-Phosphate Dehydrogenase | GC12P006630 |
| GAS5 | Growth Arrest Specific 5 | GC01M174119 |
| CDKN2B-AS1 | CDKN2B Antisense RNA 1 | GC09P021994 |
| KRT14 | Keratin 14 | GC17M041582 |
| HDAC1 | Histone Deacetylase 1 | GC01P032292 |
| SOCS1 | Suppressor Of Cytokine Signaling 1 | GC16M011255 |
| MIR9-1 | MicroRNA 9-1 | GC01M156420 |
| CTLA4 | Cytotoxic T-Lymphocyte Associated Protein 4 | GC02P203867 |
| EGR1 | Early Growth Response 1 | GC05P138465 |
| MIR142 | MicroRNA 142 | GC17M058331 |
| URGCP | Upregulator Of Cell Proliferation | GC07M043876 |
| CASP10 | Caspase 10 | GC02P201182 |
| SFRP1 | Secreted Frizzled Related Protein 1 | GC08M041238 |
| MSH3 | MutS Homolog 3 | GC05P080654 |
| HMGA2 | High Mobility Group AT-Hook 2 | GC12P065824 |
| BUB1 | BUB1 Mitotic Checkpoint Serine/Threonine Kinase | GC02M110637 |
| FGL1 | Fibrinogen Like 1 | GC08M017864 |
| MIR451A | MicroRNA 451a | GC17M028861 |
| CDX2 | Caudal Type Homeobox 2 | GC13M027962 |
| MVP | Major Vault Protein | GC16P030048 |
| ETS1 | ETS Proto-Oncogene 1, Transcription Factor | GC11M128458 |
| MIR24-1 | MicroRNA 24-1 | GC09P095086 |
| CEACAM3 | CEA Cell Adhesion Molecule 3 | GC19P041796 |
| SOX2 | SRY-Box Transcription Factor 2 | GC03P181711 |
| CCAT1 | Colon Cancer Associated Transcript 1 | GC08M127207 |
| CCR6 | C-C Motif Chemokine Receptor 6 | GC06P167111 |
| SDHC | Succinate Dehydrogenase Complex Subunit C | GC01P161314 |
| ALDH2 | Aldehyde Dehydrogenase 2 Family Member | GC12P111766 |
| CYP17A1 | Cytochrome P450 Family 17 Subfamily A Member 1 | GC10M102830 |
| PALB2 | Partner And Localizer Of BRCA2 | GC16M023603 |
| MTA1 | Metastasis Associated 1 | GC14P105419 |
| ANGPT2 | Angiopoietin 2 | GC08M006499 |
| NANOG | Nanog Homeobox | GC12P007787 |
| PRKCD | Protein Kinase C Delta | GC03P053156 |
| CD34 | CD34 Molecule | GC01M207880 |
| TG | Thyroglobulin | GC08P132866 |
| MIR144 | MicroRNA 144 | GC17M029703 |
| E2F3 | E2F Transcription Factor 3 | GC06P020402 |
| IRS1 | Insulin Receptor Substrate 1 | GC02M226731 |
| E2F2 | E2F Transcription Factor 2 | GC01M023520 |
| STMN1 | Stathmin 1 | GC01M025884 |
| SPRY4-IT1 | SPRY4 Intronic Transcript 1 | GC05U901574 |
| AKT3 | AKT Serine/Threonine Kinase 3 | GC01M243488 |
| RECK | Reversion Inducing Cysteine Rich Protein With Kazal Motifs | GC09P036036 |
| PDGFRA | Platelet Derived Growth Factor Receptor Alpha | GC04P054229 |
| TGFB2 | Transforming Growth Factor Beta 2 | GC01P218345 |
| MIR181B2 | MicroRNA 181b-2 | GC09P124693 |
| MIR101-1 | MicroRNA 101-1 | GC01M065058 |
| LRRC56 | Leucine Rich Repeat Containing 56 | GC11P000520 |
| NEAT1 | Nuclear Paraspeckle Assembly Transcript 1 | GC11P065418 |
| BUB1B | BUB1 Mitotic Checkpoint Serine/Threonine Kinase B | GC15P040161 |
| ERCC2 | ERCC Excision Repair 2, TFIIH Core Complex Helicase Subunit | GC19M045349 |
| DCC | DCC Netrin 1 Receptor | GC18P052340 |
| RSF1 | Remodeling And Spacing Factor 1 | GC11M077659 |
| SHC1 | SHC Adaptor Protein 1 | GC01M154962 |
| ENO1 | Enolase 1 | GC01M008861 |
| MAGEA4 | MAGE Family Member A4 | GC0XP151912 |
| TJP2 | Tight Junction Protein 2 | GC09P069121 |
| SMARCA2 | SWI/SNF Related, Matrix Associated, Actin Dependent Regulator Of Chromatin, Subfamily A, Member 2 | GC09P001980 |
| ABCC3 | ATP Binding Cassette Subfamily C Member 3 | GC17P050634 |
| CASC2 | Cancer Susceptibility 2 | GC10P118046 |
| PIK3CB | Phosphatidylinositol-4,5-Bisphosphate 3-Kinase Catalytic Subunit Beta | GC03M138652 |
| LINC-ROR | Long Intergenic Non-Protein Coding RNA, Regulator Of Reprogramming | GC18M057054 |
| SSTR2 | Somatostatin Receptor 2 | GC17P073165 |
| SMAD7 | SMAD Family Member 7 | GC18M048919 |
| SQSTM1 | Sequestosome 1 | GC05P179806 |
| ZC4H2 | Zinc Finger C4H2-Type Containing | GC0XM064915 |
| PXN | Paxillin | GC12M120210 |
| SOS1 | SOS Ras/Rac Guanine Nucleotide Exchange Factor 1 | GC02M038981 |
| ZFAS1 | ZNFX1 Antisense RNA 1 | GC20P049276 |
| DKK1 | Dickkopf WNT Signaling Pathway Inhibitor 1 | GC10P052314 |
| GAST | Gastrin | GC17P041712 |
| PTK2B | Protein Tyrosine Kinase 2 Beta | GC08P027311 |
| ENPP2 | Ectonucleotide Pyrophosphatase/Phosphodiesterase 2 | GC08M119556 |
| UCHL1 | Ubiquitin C-Terminal Hydrolase L1 | GC04P041256 |
| CCN2 | Cellular Communication Network Factor 2 | GC06M131948 |
| XIST | X Inactive Specific Transcript | GC0XM073820 |
| IGFBP3 | Insulin Like Growth Factor Binding Protein 3 | GC07M045912 |
| TGFB3 | Transforming Growth Factor Beta 3 | GC14M075958 |
| PNPLA3 | Patatin Like Phospholipase Domain Containing 3 | GC22P043923 |
| PAX8 | Paired Box 8 | GC02M113215 |
| SOCS3 | Suppressor Of Cytokine Signaling 3 | GC17M078356 |
| MIR196A1 | MicroRNA 196a-1 | GC17M048632 |
| SST | Somatostatin | GC03M187668 |
| AHCY | Adenosylhomocysteinase | GC20M034257 |
| CYTOR | Cytoskeleton Regulator RNA | GC02P087457 |
| CYP1A2 | Cytochrome P450 Family 1 Subfamily A Member 2 | GC15P074748 |
| HMOX1 | Heme Oxygenase 1 | GC22P035380 |
| EPHX1 | Epoxide Hydrolase 1 | GC01P225810 |
| DNMT3A | DNA Methyltransferase 3 Alpha | GC02M025228 |
| MIR99A | MicroRNA 99a | GC21P016539 |
| DIABLO | Diablo IAP-Binding Mitochondrial Protein | GC12M122208 |
| HOTTIP | HOXA Distal Transcript Antisense RNA | GC07P027198 |
| CD80 | CD80 Molecule | GC03M119524 |
| CEACAM1 | CEA Cell Adhesion Molecule 1 | GC19M042507 |
| MTHFR | Methylenetetrahydrofolate Reductase | GC01M011785 |
| HEIH | Hepatocellular Carcinoma Up-Regulated EZH2-Associated Long Non-Coding RNA | GC05M180826 |
| FGF3 | Fibroblast Growth Factor 3 | GC11M069811 |
| CDC25A | Cell Division Cycle 25A | GC03M048173 |
| ATP7B | ATPase Copper Transporting Beta | GC13M051905 |
| MIR375 | MicroRNA 375 | GC02M219001 |
| CA9 | Carbonic Anhydrase 9 | GC09P035673 |
| ERCC1 | ERCC Excision Repair 1, Endonuclease Non-Catalytic Subunit | GC19M045409 |
| SULF1 | Sulfatase 1 | GC08P069466 |
| TFE3 | Transcription Factor Binding To IGHM Enhancer 3 | GC0XM049028 |
| PLCG1 | Phospholipase C Gamma 1 | GC20P041136 |
| GOLM1 | Golgi Membrane Protein 1 | GC09M086026 |
| UROD | Uroporphyrinogen Decarboxylase | GC01P045012 |
| TYR | Tyrosinase | GC11P089177 |
| ESR2 | Estrogen Receptor 2 | GC14M064084 |
| DNAJB1 | DnaJ Heat Shock Protein Family (Hsp40) Member B1 | GC19M014514 |
| RHOC | Ras Homolog Family Member C | GC01M112701 |
| MIR124-1 | MicroRNA 124-1 | GC08M009903 |
| MIR423 | MicroRNA 423 | GC17P030117 |
| PHF20 | PHD Finger Protein 20 | GC20P035771 |
| RRM2 | Ribonucleotide Reductase Regulatory Subunit M2 | GC02P010123 |
| EPHA2 | EPH Receptor A2 | GC01M016124 |
| MSR1 | Macrophage Scavenger Receptor 1 | GC08M016107 |
| CCAT2 | Colon Cancer Associated Transcript 2 | GC08P127400 |
| FZD7 | Frizzled Class Receptor 7 | GC02P202034 |
| SERPINE1 | Serpin Family E Member 1 | GC07P101127 |
| CRP | C-Reactive Protein | GC01M159715 |
| MIR133B | MicroRNA 133b | GC06P052148 |
| HK2 | Hexokinase 2 | GC02P074833 |
| FBXW7 | F-Box And WD Repeat Domain Containing 7 | GC04M152321 |
| ERCC6 | ERCC Excision Repair 6, Chromatin Remodeling Factor | GC10M049454 |
| PTHLH | Parathyroid Hormone Like Hormone | GC12M027959 |
| MIR429 | MicroRNA 429 | GC01P001297 |
| WNT3A | Wnt Family Member 3A | GC01P228072 |
| WNT3 | Wnt Family Member 3 | GC17M046762 |
| HEPACAM | Hepatic And Glial Cell Adhesion Molecule | GC11M124919 |
| AURKB | Aurora Kinase B | GC17M008770 |
| FADD | Fas Associated Via Death Domain | GC11P070203 |
| ENO2 | Enolase 2 | GC12P006913 |
| TPX2 | TPX2 Microtubule Nucleation Factor | GC20P031739 |
| ITGAV | Integrin Subunit Alpha V | GC02P186589 |
| GJB1 | Gap Junction Protein Beta 1 | GC0XP071215 |
| ARID2 | AT-Rich Interaction Domain 2 | GC12P045729 |
| IL4 | Interleukin 4 | GC05P132673 |
| TNFRSF6B | TNF Receptor Superfamily Member 6b | GC20P063696 |
| HMGB1 | High Mobility Group Box 1 | GC13M030456 |
| ALK | ALK Receptor Tyrosine Kinase | GC02M029156 |
| MIR125B1 | MicroRNA 125b-1 | GC11M122100 |
| PRKCB | Protein Kinase C Beta | GC16P023872 |
| ITGA5 | Integrin Subunit Alpha 5 | GC12M054396 |
| MIR338 | MicroRNA 338 | GC17M081126 |
| CHP2 | Calcineurin Like EF-Hand Protein 2 | GC16P023871 |
| UBD | Ubiquitin D | GC06M029556 |
| POLE | DNA Polymerase Epsilon, Catalytic Subunit | GC12M132624 |
| ITGB4 | Integrin Subunit Beta 4 | GC17P075721 |
| CRNDE | Colorectal Neoplasia Differentially Expressed | GC16M054845 |
| PIK3R2 | Phosphoinositide-3-Kinase Regulatory Subunit 2 | GC19P018153 |
| H2AC18 | H2A Clustered Histone 18 | GC01M149914 |
| TYMS | Thymidylate Synthetase | GC18P000657 |
| IGF2BP2 | Insulin Like Growth Factor 2 MRNA Binding Protein 2 | GC03M185643 |
| COPS5 | COP9 Signalosome Subunit 5 | GC08M067043 |
| PBRM1 | Polybromo 1 | GC03M052545 |
| ANGPT1 | Angiopoietin 1 | GC08M107246 |
| PSMD10 | Proteasome 26S Subunit, Non-ATPase 10 | GC0XM108084 |
| DANCR | Differentiation Antagonizing Non-Protein Coding RNA | GC04P052712 |
| PCAT1 | Prostate Cancer Associated Transcript 1 | GC08P126553 |
| PIK3R3 | Phosphoinositide-3-Kinase Regulatory Subunit 3 | GC01M046041 |
| IFNB1 | Interferon Beta 1 | GC09M021077 |
| HSP90B1 | Heat Shock Protein 90 Beta Family Member 1 | GC12P103930 |
| PRDM2 | PR/SET Domain 2 | GC01P013700 |
| CXCR1 | C-X-C Motif Chemokine Receptor 1 | GC02M218162 |
| GSTM3 | Glutathione S-Transferase Mu 3 | GC01M109733 |
| PODXL | Podocalyxin Like | GC07M131500 |
| LOX | Lysyl Oxidase | GC05M122063 |
| HLA-A | Major Histocompatibility Complex, Class I, A | GC06P033211 |
| SF3B2 | Splicing Factor 3b Subunit 2 | GC11P066050 |
| PANDAR | Promoter Of CDKN1A Antisense DNA Damage Activated RNA | GC06M036673 |
| MST1R | Macrophage Stimulating 1 Receptor | GC03M049894 |
| IFNAR2 | Interferon Alpha And Beta Receptor Subunit 2 | GC21P033229 |
| MDK | Midkine | GC11P046402 |
| SNHG1 | Small Nucleolar RNA Host Gene 1 | GC11M063085 |
| MIRLET7I | MicroRNA Let-7i | GC12P062606 |
| DDIT3 | DNA Damage Inducible Transcript 3 | GC12M057516 |
| NOTCH4 | Notch Receptor 4 | GC06M032407 |
| AFAP1-AS1 | AFAP1 Antisense RNA 1 | GC04P007756 |
| RNASEL | Ribonuclease L | GC01M182542 |
| GSTT1 | Glutathione S-Transferase Theta 1 | GC22Mi00270 |
| PTPRH | Protein Tyrosine Phosphatase Receptor Type H | GC19M055181 |
| FLT4 | Fms Related Receptor Tyrosine Kinase 4 | GC05M180607 |
| ARG1 | Arginase 1 | GC06P131473 |
| CCL5 | C-C Motif Chemokine Ligand 5 | GC17M035871 |
| SDHD | Succinate Dehydrogenase Complex Subunit D | GC11P112087 |
| MIR26B | MicroRNA 26b | GC02P218402 |
| LINC00261 | Long Intergenic Non-Protein Coding RNA 261 | GC20M022547 |
| LARP1 | La Ribonucleoprotein 1, Translational Regulator | GC05P154682 |
| VDR | Vitamin D Receptor | GC12M047841 |
| MRE11 | MRE11 Homolog, Double Strand Break Repair Nuclease | GC11M094416 |
| NDUFA13 | NADH:Ubiquinone Oxidoreductase Subunit A13 | GC19P019515 |
| LRP6 | LDL Receptor Related Protein 6 | GC12M013402 |
| GHET1 | Gastric Carcinoma Proliferation Enhancing Transcript 1 | GC07P149023 |
| PTENP1 | Phosphatase And Tensin Homolog Pseudogene 1 | GC09M033673 |
| CYP3A4 | Cytochrome P450 Family 3 Subfamily A Member 4 | GC07M099759 |
| TLR3 | Toll Like Receptor 3 | GC04P186059 |
| CTNNA1 | Catenin Alpha 1 | GC05P138613 |
| SREBF1 | Sterol Regulatory Element Binding Transcription Factor 1 | GC17M017810 |
| ELK1 | ETS Transcription Factor ELK1 | GC0XM047635 |
| HNF1A-AS1 | HNF1A Antisense RNA 1 | GC12M121014 |
| WNT2 | Wnt Family Member 2 | GC07M117276 |
| HAMP | Hepcidin Antimicrobial Peptide | GC19P037707 |
| CCKBR | Cholecystokinin B Receptor | GC11P006259 |
| SET | SET Nuclear Proto-Oncogene | GC09P128684 |
| PIGR | Polymeric Immunoglobulin Receptor | GC01M206928 |
| GLUL | Glutamate-Ammonia Ligase | GC01M182350 |
| MIR197 | MicroRNA 197 | GC01P109549 |
| MIR133A1 | MicroRNA 133a-1 | GC18M021826 |
| MIR30D | MicroRNA 30d | GC08M134804 |
| GAB1 | GRB2 Associated Binding Protein 1 | GC04P143336 |
| HMBS | Hydroxymethylbilane Synthase | GC11P119084 |
| MIR296 | MicroRNA 296 | GC20M058817 |
| CCL2 | C-C Motif Chemokine Ligand 2 | GC17P034255 |
| HDGF | Heparin Binding Growth Factor | GC01M156749 |
| CPQ | Carboxypeptidase Q | GC08P096645 |
| RBM39 | RNA Binding Motif Protein 39 | GC20M035703 |
| MIR491 | MicroRNA 491 | GC09P020716 |
| ACTG1 | Actin Gamma 1 | GC17M081509 |
| HDAC9 | Histone Deacetylase 9 | GC07P018086 |
| PLPP5 | Phospholipid Phosphatase 5 | GC08M038261 |
| SMYD3 | SET And MYND Domain Containing 3 | GC01M245749 |
| INSR | Insulin Receptor | GC19M007112 |
| PINX1 | PIN2 (TERF1) Interacting Telomerase Inhibitor 1 | GC08M010726 |
| GADD45B | Growth Arrest And DNA Damage Inducible Beta | GC19P002476 |
| LGALS3 | Galectin 3 | GC14P055124 |
| MIR132 | MicroRNA 132 | GC17M002049 |
| BAP1 | BRCA1 Associated Protein 1 | GC03M052401 |
| MIR148B | MicroRNA 148b | GC12P054337 |
| URI1 | URI1 Prefoldin Like Chaperone | GC19P029923 |
| ACTB | Actin Beta | GC07M005527 |
| FABP1 | Fatty Acid Binding Protein 1 | GC02M088122 |
| NUS1 | NUS1 Dehydrodolichyl Diphosphate Synthase Subunit | GC06P117675 |
| GADD45G | Growth Arrest And DNA Damage Inducible Gamma | GC09P089605 |
| LAPTM4B | Lysosomal Protein Transmembrane 4 Beta | GC08P097775 |
| CAT | Catalase | GC11P034460 |
| NPM1 | Nucleophosmin 1 | GC05P171387 |
| WWOX | WW Domain Containing Oxidoreductase | GC16P078099 |
| FRZB | Frizzled Related Protein | GC02M182833 |
| BANCR | BRAF-Activated Non-Protein Coding RNA | GC09M069296 |
| SLCO1B3 | Solute Carrier Organic Anion Transporter Family Member 1B3 | GC12P020810 |
| CNDP2 | Carnosine Dipeptidase 2 | GC18P074495 |
| MGAT5 | Alpha-1,6-Mannosylglycoprotein 6-Beta-N-Acetylglucosaminyltransferase | GC02P134119 |
| B2M | Beta-2-Microglobulin | GC15P044711 |
| VPS37A | VPS37A Subunit Of ESCRT-I | GC08P017246 |
| MIR363 | MicroRNA 363 | GC0XM134214 |
| CASC15 | Cancer Susceptibility 15 | GC06P021669 |
| ASS1 | Argininosuccinate Synthase 1 | GC09P130444 |
| LIN28B | Lin-28 Homolog B | GC06P104939 |
| RAD51C | RAD51 Paralog C | GC17P058692 |
| SOX2-OT | SOX2 Overlapping Transcript | GC03P180989 |
| KLRK1 | Killer Cell Lectin Like Receptor K1 | GC12M013372 |
| CYP1B1 | Cytochrome P450 Family 1 Subfamily B Member 1 | GC02M038034 |
| CLDN7 | Claudin 7 | GC17M007259 |
| MIR186 | MicroRNA 186 | GC01M071067 |
| MAT1A | Methionine Adenosyltransferase 1A | GC10M080271 |
| RCHY1 | Ring Finger And CHY Zinc Finger Domain Containing 1 | GC04M075479 |
| CDH17 | Cadherin 17 | GC08M094127 |
| TCF7 | Transcription Factor 7 | GC05P134114 |
| NTS | Neurotensin | GC12P085876 |
| RAD51 | RAD51 Recombinase | GC15P040694 |
| DYNLRB1 | Dynein Light Chain Roadblock-Type 1 | GC20P034516 |
| YY1AP1 | YY1 Associated Protein 1 | GC01M155659 |
| CLDN10 | Claudin 10 | GC13P095433 |
| LNCRNA-ATB | Long Noncoding RNA Activated By TGF-Beta | GC14U902188 |
| GPX3 | Glutathione Peroxidase 3 | GC05P150997 |
| ZEB2-AS1 | ZEB2 Antisense RNA 1 | GC02P144519 |
| GSTA1 | Glutathione S-Transferase Alpha 1 | GC06M052791 |
| BATF2 | Basic Leucine Zipper ATF-Like Transcription Factor 2 | GC11M064987 |
| ATP8B1 | ATPase Phospholipid Transporting 8B1 | GC18M057646 |
| SEMA4A | Semaphorin 4A | GC01P156119 |
| CREB3L3 | CAMP Responsive Element Binding Protein 3 Like 3 | GC19P004153 |
| HOXA11-AS | HOXA11 Antisense RNA | GC07P027184 |
| SLC37A4 | Solute Carrier Family 37 Member 4 | GC11M119024 |
| TFR2 | Transferrin Receptor 2 | GC07M100620 |
| PLAG1 | PLAG1 Zinc Finger | GC08M056161 |
| NPTN-IT1 | NPTN Intronic Transcript 1 | GC15M073566 |
| CBR3-AS1 | CBR3 Antisense RNA 1 | GC21M036131 |
| DEPDC5 | DEP Domain Containing 5, GATOR1 Subcomplex Subunit | GC22P031753 |
| SDHA | Succinate Dehydrogenase Complex Flavoprotein Subunit A | GC05P000208 |
| GNMT | Glycine N-Methyltransferase | GC06P046115 |
| MIR490 | MicroRNA 490 | GC07P136903 |
| ARID1B | AT-Rich Interaction Domain 1B | GC06P156777 |
| KLK3 | Kallikrein Related Peptidase 3 | GC19P050854 |
| ZNRD1ASP | Zinc Ribbon Domain Containing 1 Antisense, Pseudogene | GC06M030650 |
| NAA40 | N-Alpha-Acetyltransferase 40, NatD Catalytic Subunit | GC11P063938 |
| ING1 | Inhibitor Of Growth Family Member 1 | GC13P110712 |
| FTX | FTX Transcript, XIST Regulator | GC0XM073946 |
| CTSD | Cathepsin D | GC11M001752 |
| FZD2 | Frizzled Class Receptor 2 | GC17P044557 |
| STEAP3 | STEAP3 Metalloreductase | GC02P119222 |
| MIR212 | MicroRNA 212 | GC17M002050 |
| MIR210 | MicroRNA 210 | GC11M000622 |
| GSTO2 | Glutathione S-Transferase Omega 2 | GC10P104268 |
| BMI1 | BMI1 Proto-Oncogene, Polycomb Ring Finger | GC10P022326 |
| WNT11 | Wnt Family Member 11 | GC11M076186 |
| RBP5 | Retinol Binding Protein 5 | GC12M007123 |
| TCP10L | T-Complex 10 Like | GC21M032574 |
| BCL2L11 | BCL2 Like 11 | GC02P111119 |
| TUSC7 | Tumor Suppressor Candidate 7 | GC03P116709 |
| RNF43 | Ring Finger Protein 43 | GC17M058352 |
| FUCA1 | Alpha-L-Fucosidase 1 | GC01M023845 |
| LEP | Leptin | GC07P128241 |
| CREB1 | CAMP Responsive Element Binding Protein 1 | GC02P207529 |
| NORAD | Non-Coding RNA Activated By DNA Damage | GC20M036048 |
| WNT4 | Wnt Family Member 4 | GC01M022190 |
| TXNRD1 | Thioredoxin Reductase 1 | GC12P104215 |
| SLC4A2 | Solute Carrier Family 4 Member 2 | GC07P151057 |
| RAD51D | RAD51 Paralog D | GC17M035092 |
| TP53COR1 | Tumor Protein P53 Pathway Corepressor 1 | GC06U903133 |
| DVL1 | Dishevelled Segment Polarity Protein 1 | GC01M001335 |
| INTS3 | Integrator Complex Subunit 3 | GC01P153728 |
| GLI1 | GLI Family Zinc Finger 1 | GC12P057460 |
| NOTUM | Notum, Palmitoleoyl-Protein Carboxylesterase | GC17M081952 |
| MMP3 | Matrix Metallopeptidase 3 | GC11M102835 |
| MIR30C1 | MicroRNA 30c-1 | GC01P040757 |
| GLS2 | Glutaminase 2 | GC12M056470 |
| BRD7 | Bromodomain Containing 7 | GC16M050313 |
| SMO | Smoothened, Frizzled Class Receptor | GC07P129303 |
| WNT5B | Wnt Family Member 5B | GC12P001529 |
| DICER1 | Dicer 1, Ribonuclease III | GC14M095086 |
| WNT7B | Wnt Family Member 7B | GC22M045920 |
| CHUK | Component Of Inhibitor Of Nuclear Factor Kappa B Kinase Complex | GC10M100188 |
| MIR374A | MicroRNA 374a | GC0XM074297 |
| SNHG15 | Small Nucleolar RNA Host Gene 15 | GC07M044983 |
| SNHG12 | Small Nucleolar RNA Host Gene 12 | GC01M028578 |
| DACT1 | Dishevelled Binding Antagonist Of Beta Catenin 1 | GC14P058633 |
| CD81 | CD81 Molecule | GC11P002377 |
| IL2RA | Interleukin 2 Receptor Subunit Alpha | GC10M006010 |
| ODC1 | Ornithine Decarboxylase 1 | GC02M010432 |
| XPC | XPC Complex Subunit, DNA Damage Recognition And Repair Factor | GC03M015413 |
| UGT1A7 | UDP Glucuronosyltransferase Family 1 Member A7 | GC02P233681 |
| RELA | RELA Proto-Oncogene, NF-KB Subunit | GC11M065653 |
| FBP1 | Fructose-Bisphosphatase 1 | GC09M094603 |
| ALDOB | Aldolase, Fructose-Bisphosphate B | GC09M101420 |
| GSTO1 | Glutathione S-Transferase Omega 1 | GC10P104235 |
| IL17A | Interleukin 17A | GC06P052186 |
| EGFR-AS1 | EGFR Antisense RNA 1 | GC07M055179 |
| WT1 | WT1 Transcription Factor | GC11M032365 |
| PDGFRB | Platelet Derived Growth Factor Receptor Beta | GC05M150113 |
| MIR125B2 | MicroRNA 125b-2 | GC21P016590 |
| MIR219A1 | MicroRNA 219a-1 | GC06P033207 |
| RIOX2 | Ribosomal Oxygenase 2 | GC03M097942 |
| IL12A | Interleukin 12A | GC03P159988 |
| CSF3 | Colony Stimulating Factor 3 | GC17P040015 |
| PIK3CD | Phosphatidylinositol-4,5-Bisphosphate 3-Kinase Catalytic Subunit Delta | GC01P009629 |
| ASPH | Aspartate Beta-Hydroxylase | GC08M061500 |
| CHEK1 | Checkpoint Kinase 1 | GC11P125625 |
| MIR100 | MicroRNA 100 | GC11M122152 |
| SERPINB5 | Serpin Family B Member 5 | GC18P063476 |
| ACTC1 | Actin Alpha Cardiac Muscle 1 | GC15M034788 |
| NR1H2 | Nuclear Receptor Subfamily 1 Group H Member 2 | GC19P050329 |
| GDF15 | Growth Differentiation Factor 15 | GC19P022252 |
| MAP2K2 | Mitogen-Activated Protein Kinase Kinase 2 | GC19M004090 |
| HJV | Hemojuvelin BMP Co-Receptor | GC01M146018 |
| LAMC2 | Laminin Subunit Gamma 2 | GC01P183155 |
| EZR | Ezrin | GC06M158765 |
| HOXA13 | Homeobox A13 | GC07M027232 |
| SNHG20 | Small Nucleolar RNA Host Gene 20 | GC17P077087 |
| SARNP | SAP Domain Containing Ribonucleoprotein | GC12M055752 |
| HSP90AA1 | Heat Shock Protein 90 Alpha Family Class A Member 1 | GC14M102080 |
| NTRK3 | Neurotrophic Receptor Tyrosine Kinase 3 | GC15M087859 |
| HLA-DRB1 | Major Histocompatibility Complex, Class II, DR Beta 1 | GC06M032578 |
| CYP19A1 | Cytochrome P450 Family 19 Subfamily A Member 1 | GC15M051208 |
| RASA1 | RAS P21 Protein Activator 1 | GC05P087267 |
| NOTCH3 | Notch Receptor 3 | GC19M015159 |
| THBS1 | Thrombospondin 1 | GC15P039581 |
| MECOM | MDS1 And EVI1 Complex Locus | GC03M169083 |
| GSTM2 | Glutathione S-Transferase Mu 2 | GC01P109668 |
| TIFA | TRAF Interacting Protein With Forkhead Associated Domain | GC04M112274 |
| DVL3 | Dishevelled Segment Polarity Protein 3 | GC03P184155 |
| MIR151A | MicroRNA 151a | GC08M140733 |
| HINT2 | Histidine Triad Nucleotide Binding Protein 2 | GC09M035812 |
| KDM4C | Lysine Demethylase 4C | GC09P006720 |
| ECI2 | Enoyl-CoA Delta Isomerase 2 | GC06M004115 |
| NUTM1 | NUT Midline Carcinoma Family Member 1 | GC15P034343 |
| POLD1 | DNA Polymerase Delta 1, Catalytic Subunit | GC19P050385 |
| MUC2 | Mucin 2, Oligomeric Mucus/Gel-Forming | GC11P001074 |
| TOP2A | DNA Topoisomerase II Alpha | GC17M040388 |
| PTPN3 | Protein Tyrosine Phosphatase Non-Receptor Type 3 | GC09M109375 |
| CXCR3 | C-X-C Motif Chemokine Receptor 3 | GC0XM071615 |
| MIR301A | MicroRNA 301a | GC17M059151 |
| FOXO1 | Forkhead Box O1 | GC13M040555 |
| ANGPTL8 | Angiopoietin Like 8 | GC19P011238 |
| GRP | Gastrin Releasing Peptide | GC18P059220 |
| MT-CO1 | Mitochondrially Encoded Cytochrome C Oxidase I | GCMTP005906 |
| MIR485 | MicroRNA 485 | GC14P104467 |
| ZEB1-AS1 | ZEB1 Antisense RNA 1 | GC10M031166 |
| ADIPOQ | Adiponectin, C1Q And Collagen Domain Containing | GC03P186842 |
| DVL2 | Dishevelled Segment Polarity Protein 2 | GC17M007225 |
| IGF2BP3 | Insulin Like Growth Factor 2 MRNA Binding Protein 3 | GC07M023316 |
| CCEPR | Cervical Carcinoma Expressed PCNA Regulatory LncRNA | GC10U902486 |
| FER1L4 | Fer-1 Like Family Member 4 (Pseudogene) | GC20M035558 |
| HOXA-AS2 | HOXA Cluster Antisense RNA 2 | GC07P027107 |
| PIM2 | Pim-2 Proto-Oncogene, Serine/Threonine Kinase | GC0XM048913 |
| DGCR5 | DiGeorge Syndrome Critical Region Gene 5 | GC22P019451 |
| IGFBP2 | Insulin Like Growth Factor Binding Protein 2 | GC02P216632 |
| LECT2 | Leukocyte Cell Derived Chemotaxin 2 | GC05M135922 |
| DNAJA3 | DnaJ Heat Shock Protein Family (Hsp40) Member A3 | GC16P004425 |
| F5 | Coagulation Factor V | GC01M169511 |
| CPE | Carboxypeptidase E | GC04P165361 |
| MMP13 | Matrix Metallopeptidase 13 | GC11M102942 |
| NCAM1 | Neural Cell Adhesion Molecule 1 | GC11P112961 |
| SOD1 | Superoxide Dismutase 1 | GC21P031659 |
| RASGRP1 | RAS Guanyl Releasing Protein 1 | GC15M038488 |
| PCBP2-OT1 | PCBP2 Overlapping Transcript 1 | GC12P053464 |
| PDPN | Podoplanin | GC01P013583 |
| GSTA4 | Glutathione S-Transferase Alpha 4 | GC06M052977 |
| HAGLR | HOXD Antisense Growth-Associated Long Non-Coding RNA | GC02M176173 |
| ATF6 | Activating Transcription Factor 6 | GC01P161766 |
| MGST1 | Microsomal Glutathione S-Transferase 1 | GC12P016347 |
| EHD4 | EH Domain Containing 4 | GC15M041895 |
| TFDP1 | Transcription Factor Dp-1 | GC13P113584 |
| SNHG6 | Small Nucleolar RNA Host Gene 6 | GC08M066921 |
| IL1A | Interleukin 1 Alpha | GC02M112773 |
| ALDH3A1 | Aldehyde Dehydrogenase 3 Family Member A1 | GC17M019737 |
| CDH3 | Cadherin 3 | GC16P068637 |
| PCAT29 | Prostate Cancer Associated Transcript 29 | GC15P072009 |
| ARHGEF39 | Rho Guanine Nucleotide Exchange Factor 39 | GC09M035658 |
| SRA1 | Steroid Receptor RNA Activator 1 | GC05M140537 |
| OTUD7A | OTU Deubiquitinase 7A | GC15M031475 |
| FGF19 | Fibroblast Growth Factor 19 | GC11M069699 |
| MMP11 | Matrix Metallopeptidase 11 | GC22P023768 |
| MIR9-2 | MicroRNA 9-2 | GC05M088666 |
| SAPCD2 | Suppressor APC Domain Containing 2 | GC09M137062 |
| TFDP3 | Transcription Factor Dp Family Member 3 | GC0XM133216 |
| GLYAT | Glycine-N-Acyltransferase | GC11M059800 |
| LGALS1 | Galectin 1 | GC22P037675 |
| AZIN1 | Antizyme Inhibitor 1 | GC08M102826 |
| RAB11FIP4 | RAB11 Family Interacting Protein 4 | GC17P031391 |
| MMP12 | Matrix Metallopeptidase 12 | GC11M102862 |
| TF | Transferrin | GC03P133666 |
| NKILA | NF-KappaB Interacting LncRNA | GC20P057711 |
| DYNLRB2 | Dynein Light Chain Roadblock-Type 2 | GC16P080540 |
| IKBKB | Inhibitor Of Nuclear Factor Kappa B Kinase Subunit Beta | GC08P042271 |
| TP73-AS1 | TP73 Antisense RNA 1 | GC01M003735 |
| SLC25A47 | Solute Carrier Family 25 Member 47 | GC14P100323 |
| SAMD9L | Sterile Alpha Motif Domain Containing 9 Like | GC07M093130 |
| TTC36 | Tetratricopeptide Repeat Domain 36 | GC11P118527 |
| PSD3 | Pleckstrin And Sec7 Domain Containing 3 | GC08M018527 |
| MUC5AC | Mucin 5AC, Oligomeric Mucus/Gel-Forming | GC11P001151 |
| AXL | AXL Receptor Tyrosine Kinase | GC19P041219 |
| SLC40A1 | Solute Carrier Family 40 Member 1 | GC02M189560 |
| NOTCH2 | Notch Receptor 2 | GC01M119911 |
| PDX1 | Pancreatic And Duodenal Homeobox 1 | GC13P027921 |
| CLDN1 | Claudin 1 | GC03M190305 |
| HLA-G | Major Histocompatibility Complex, Class I, G | GC06P033174 |
| SEC23B | SEC23 Homolog B, COPII Coat Complex Component | GC20P018507 |
| DRAIC | Downregulated RNA In Cancer, Inhibitor Of Cell Invasion And Migration | GC15P071841 |
| WT1-AS | WT1 Antisense RNA | GC11P032434 |
| HMGCR | 3-Hydroxy-3-Methylglutaryl-CoA Reductase | GC05P075336 |
| TMEM176A | Transmembrane Protein 176A | GC07P150800 |
| MUC16 | Mucin 16, Cell Surface Associated | GC19M008848 |
| MIR216A | MicroRNA 216a | GC02M055988 |
| IRF5 | Interferon Regulatory Factor 5 | GC07P128937 |
| CASC11 | Cancer Susceptibility 11 | GC08M127709 |
| DAPK1 | Death Associated Protein Kinase 1 | GC09P087497 |
| BHMT | Betaine--Homocysteine S-Methyltransferase | GC05P079111 |
| JUP | Junction Plakoglobin | GC17M041754 |
| MIR22HG | MIR22 Host Gene | GC17M001711 |
| SLC17A5 | Solute Carrier Family 17 Member 5 | GC06M073593 |
| TFRC | Transferrin Receptor | GC03M196027 |
| HLA-B | Major Histocompatibility Complex, Class I, B | GC06M031289 |
| CERNA2 | Competing Endogenous LncRNA 2 For MicroRNA Let-7b | GC10M084169 |
| ZDHHC2 | Zinc Finger DHHC-Type Palmitoyltransferase 2 | GC08P017156 |
| MIRLET7A2 | MicroRNA Let-7a-2 | GC11M122146 |
| S100A1 | S100 Calcium Binding Protein A1 | GC01P153627 |
| ACTL6A | Actin Like 6A | GC03P179562 |
| AMACR | Alpha-Methylacyl-CoA Racemase | GC05M033986 |
| CD40 | CD40 Molecule | GC20P046118 |
| CLU | Clusterin | GC08M027596 |
| MIR10A | MicroRNA 10a | GC17M048579 |
| MIR124-2 | MicroRNA 124-2 | GC08P064379 |
| FRAT1 | FRAT Regulator Of WNT Signaling Pathway 1 | GC10P097319 |
| TFF1 | Trefoil Factor 1 | GC21M042362 |
| LINC00473 | Long Intergenic Non-Protein Coding RNA 473 | GC06M165328 |
| TLR9 | Toll Like Receptor 9 | GC03M052222 |
| SOX9 | SRY-Box Transcription Factor 9 | GC17P072121 |
| CD36 | CD36 Molecule | GC07P080369 |
| MIR499A | MicroRNA 499a | GC20P034990 |
| VEGFD | Vascular Endothelial Growth Factor D | GC0XM015345 |
| ACSL4 | Acyl-CoA Synthetase Long Chain Family Member 4 | GC0XM109624 |
| FALEC | Focally Amplified Long Non-Coding RNA In Epithelial Cancer | GC01P150515 |
| MIR106A | MicroRNA 106a | GC0XM134249 |
| S100A4 | S100 Calcium Binding Protein A4 | GC01M153543 |
| XRCC2 | X-Ray Repair Cross Complementing 2 | GC07M152644 |
| PRAP1 | Proline Rich Acidic Protein 1 | GC10P133347 |
| HLA-DQB1 | Major Histocompatibility Complex, Class II, DQ Beta 1 | GC06M032660 |
| IRF1 | Interferon Regulatory Factor 1 | GC05M132481 |
| DDR2 | Discoidin Domain Receptor Tyrosine Kinase 2 | GC01P162631 |
| CYLD | CYLD Lysine 63 Deubiquitinase | GC16P050742 |
| TGS1 | Trimethylguanosine Synthase 1 | GC08P055773 |
| HSPA1A | Heat Shock Protein Family A (Hsp70) Member 1A | GC06P033429 |
| CFAP52 | Cilia And Flagella Associated Protein 52 | GC17P009576 |
| NNT-AS1 | NNT Antisense RNA 1 | GC05M043572 |
| TNFRSF1A | TNF Receptor Superfamily Member 1A | GC12M006328 |
| CD4 | CD4 Molecule | GC12P006786 |
| LEPR | Leptin Receptor | GC01P065421 |
| HP | Haptoglobin | GC16P072089 |
| MIR500A | MicroRNA 500a | GC0XP050008 |
| NR0B2 | Nuclear Receptor Subfamily 0 Group B Member 2 | GC01M026922 |
| PLK1 | Polo Like Kinase 1 | GC16P023869 |
| CHKA | Choline Kinase Alpha | GC11M068052 |
| C9orf78 | Chromosome 9 Open Reading Frame 78 | GC09M129827 |
| TOP1 | DNA Topoisomerase I | GC20P041028 |
| CYP2D6 | Cytochrome P450 Family 2 Subfamily D Member 6 | GC22M044576 |
| DPP4 | Dipeptidyl Peptidase 4 | GC02M161992 |
| KLF4 | Kruppel Like Factor 4 | GC09M107484 |
| CTSB | Cathepsin B | GC08M011842 |
| ERBB4 | Erb-B2 Receptor Tyrosine Kinase 4 | GC02M211375 |
| KLHDC2 | Kelch Domain Containing 2 | GC14P049767 |
| EIF2D | Eukaryotic Translation Initiation Factor 2D | GC01M206571 |
| ALPP | Alkaline Phosphatase, Placental | GC02P232378 |
| COX5A | Cytochrome C Oxidase Subunit 5A | GC15M074919 |
| IGF2-AS | IGF2 Antisense RNA | GC11P002140 |
| SF3B1 | Splicing Factor 3b Subunit 1 | GC02M197389 |
| MIR1-2 | MicroRNA 1-2 | GC18M021828 |
| CPS1-IT1 | CPS1 Intronic Transcript 1 | GC02P210617 |
| MIR26A2 | MicroRNA 26a-2 | GC12M057824 |
| ALDOA | Aldolase, Fructose-Bisphosphate A | GC16P030082 |
| PRAL | P53 Regulation Associated LncRNA | GC17M006773 |
| IGFBP1 | Insulin Like Growth Factor Binding Protein 1 | GC07P046473 |
| GAS5-AS1 | GAS5 Antisense RNA 1 | GC01P173863 |
| MTDH | Metadherin | GC08P097644 |
| RAD50 | RAD50 Double Strand Break Repair Protein | GC05P132556 |
| NAT2 | N-Acetyltransferase 2 | GC08P018391 |
| MITF | Melanocyte Inducing Transcription Factor | GC03P069788 |
| FSCN1 | Fascin Actin-Bundling Protein 1 | GC07P005592 |
| MIR103A2 | MicroRNA 103a-2 | GC20P003917 |
| HAVCR2 | Hepatitis A Virus Cellular Receptor 2 | GC05M157063 |
| EIF4E | Eukaryotic Translation Initiation Factor 4E | GC04M098871 |
| MIR136 | MicroRNA 136 | GC14P104255 |
| MST1 | Macrophage Stimulating 1 | GC03M049683 |
| FOXO3 | Forkhead Box O3 | GC06P108559 |
| ERCC5 | ERCC Excision Repair 5, Endonuclease | GC13P102808 |
| CALB2 | Calbindin 2 | GC16P071392 |
| FOXA1 | Forkhead Box A1 | GC14M037589 |
| SHH | Sonic Hedgehog Signaling Molecule | GC07M155799 |
| CD24 | CD24 Molecule | GC06M106969 |
| CCR5 | C-C Motif Chemokine Receptor 5 (Gene/Pseudogene) | GC03P046384 |
| PIP | Prolactin Induced Protein | GC07P143132 |
| MSLN | Mesothelin | GC16P001255 |
| CALR | Calreticulin | GC19P012938 |
| CTNND1 | Catenin Delta 1 | GC11P057777 |
| RARA | Retinoic Acid Receptor Alpha | GC17P040309 |
| LINC01138 | Long Intergenic Non-Protein Coding RNA 1138 | GC01M148307 |
| IL2RB | Interleukin 2 Receptor Subunit Beta | GC22M037125 |
| CSF2 | Colony Stimulating Factor 2 | GC05P132073 |
| ATP7A | ATPase Copper Transporting Alpha | GC0XP077924 |
| FOXE1 | Forkhead Box E1 | GC09P097853 |
| DLEC1 | DLEC1 Cilia And Flagella Associated Protein | GC03P038038 |
| C20orf204 | Chromosome 20 Open Reading Frame 204 | GC20P064254 |
| PRL | Prolactin | GC06M022230 |
| IL11 | Interleukin 11 | GC19M055364 |
| LINC00210 | Long Intergenic Non-Protein Coding RNA 210 | GC01P217892 |
| SNHG3 | Small Nucleolar RNA Host Gene 3 | GC01P028506 |
| MIR346 | MicroRNA 346 | GC10M086264 |
| BRD4 | Bromodomain Containing 4 | GC19M015236 |
| RPS20 | Ribosomal Protein S20 | GC08M056067 |
| APOA1 | Apolipoprotein A1 | GC11M116835 |
| EPHB2 | EPH Receptor B2 | GC01P022710 |
| ITGA1 | Integrin Subunit Alpha 1 | GC05P052788 |
| MIR373 | MicroRNA 373 | GC19P054121 |
| MIR146B | MicroRNA 146b | GC10P102436 |
| CXCL9 | C-X-C Motif Chemokine Ligand 9 | GC04M076001 |
| FAN1 | FANCD2 And FANCI Associated Nuclease 1 | GC15P031231 |
| ITGA6 | Integrin Subunit Alpha 6 | GC02P172427 |
| LZTS1 | Leucine Zipper Tumor Suppressor 1 | GC08M020246 |
| EIF2AK2 | Eukaryotic Translation Initiation Factor 2 Alpha Kinase 2 | GC02M037099 |
| CD86 | CD86 Molecule | GC03P122055 |
| PDCD4 | Programmed Cell Death 4 | GC10P110871 |
| MT-CYB | Mitochondrially Encoded Cytochrome B | GCMTP014749 |
| MIR34B | MicroRNA 34b | GC11P111578 |
| TFEB | Transcription Factor EB | GC06M041835 |
| CD8A | CD8a Molecule | GC02M086784 |
| CTCF | CCCTC-Binding Factor | GC16P067563 |
| CIB1 | Calcium And Integrin Binding 1 | GC15M090229 |
| GIHCG | GIHCG Inhibitor Of MiR-200b/200a/429 Expression | GC12M057957 |
| DBH-AS1 | DBH Antisense RNA 1 | GC09M133654 |
| AOC4P | Amine Oxidase Copper Containing 4, Pseudogene | GC17P042865 |
| CDH13 | Cadherin 13 | GC16P082626 |
| MIR494 | MicroRNA 494 | GC14P104471 |
| ARAF | A-Raf Proto-Oncogene, Serine/Threonine Kinase | GC0XP047562 |
| TNFSF11 | TNF Superfamily Member 11 | GC13P042562 |
| XPA | XPA, DNA Damage Recognition And Repair Factor | GC09M097635 |
| PHB | Prohibitin | GC17M049404 |
| MIR486-1 | MicroRNA 486-1 | GC08M041660 |
| IL7 | Interleukin 7 | GC08M078689 |
| MAP3K20 | Mitogen-Activated Protein Kinase Kinase Kinase 20 | GC02P173076 |
| SRGAP1 | SLIT-ROBO Rho GTPase Activating Protein 1 | GC12P063844 |
| MIR33A | MicroRNA 33a | GC22P041900 |
| CDH4 | Cadherin 4 | GC20P061252 |
| GZMB | Granzyme B | GC14M024630 |
| ELAVL1 | ELAV Like RNA Binding Protein 1 | GC19M007958 |
| ST14 | Suppression Of Tumorigenicity 14 | GC11P130159 |
| HBEGF | Heparin Binding EGF Like Growth Factor | GC05M140332 |
| TKT | Transketolase | GC03M053224 |
| MICA | MHC Class I Polypeptide-Related Sequence A | GC06P031399 |
| MIR335 | MicroRNA 335 | GC07P130496 |
| CXCL10 | C-X-C Motif Chemokine Ligand 10 | GC04M076021 |
| NOD2 | Nucleotide Binding Oligomerization Domain Containing 2 | GC16P050693 |
| AHR | Aryl Hydrocarbon Receptor | GC07P016916 |
| PCNA-AS1 | PCNA Antisense RNA 1 | GC20P005119 |
| NCOA4 | Nuclear Receptor Coactivator 4 | GC10M046005 |
| MIR7-3HG | MIR7-3 Host Gene | GC19P004788 |
| ALDH1A1 | Aldehyde Dehydrogenase 1 Family Member A1 | GC09M072900 |
| MIR30B | MicroRNA 30b | GC08M134800 |
| GJA1 | Gap Junction Protein Alpha 1 | GC06P121436 |
| MT-CO2 | Mitochondrially Encoded Cytochrome C Oxidase II | GCMTP007587 |
| SALL4 | Spalt Like Transcription Factor 4 | GC20M051784 |
| CCR7 | C-C Motif Chemokine Receptor 7 | GC17M040556 |
| HSPA6 | Heat Shock Protein Family A (Hsp70) Member 6 | GC01P161524 |
| BRIP1 | BRCA1 Interacting Protein C-Terminal Helicase 1 | GC17M061679 |
| MPO | Myeloperoxidase | GC17M058269 |
| ZNF350-AS1 | ZNF350 Antisense RNA 1 | GC19P051951 |
| UGT1A1 | UDP Glucuronosyltransferase Family 1 Member A1 | GC02P233760 |
| IL18 | Interleukin 18 | GC11M112143 |
| NCOA1 | Nuclear Receptor Coactivator 1 | GC02P024492 |
| LINC00974 | Long Intergenic Non-Protein Coding RNA 974 | GC17M041549 |
| SLC10A1 | Solute Carrier Family 10 Member 1 | GC14M069775 |
| ROCK1 | Rho Associated Coiled-Coil Containing Protein Kinase 1 | GC18M020946 |
| MIRLET7F2 | MicroRNA Let-7f-2 | GC0XM053574 |
| GADD45A | Growth Arrest And DNA Damage Inducible Alpha | GC01P067685 |
| PPM1D | Protein Phosphatase, Mg2+/Mn2+ Dependent 1D | GC17P060600 |
| JPX | JPX Transcript, XIST Activator | GC0XP073963 |
| NR2F1-AS1 | NR2F1 Antisense RNA 1 | GC05M093409 |
| LINC01018 | Long Intergenic Non-Protein Coding RNA 1018 | GC05P006582 |
| ACTA2-AS1 | ACTA2 Antisense RNA 1 | GC10P088932 |
| LINC00665 | Long Intergenic Non-Protein Coding RNA 665 | GC19M041417 |
| ZNF674-AS1 | ZNF674 Antisense RNA 1 (Head To Head) | GC0XP046545 |
| PDIA3P1 | Protein Disulfide Isomerase Family A Member 3 Pseudogene 1 | GC01P147188 |
| GPC3-AS1 | GPC3 Antisense RNA 1 | GC0XP133897 |
| LDC1P | Leucine Decarboxylase 1, Pseudogene | GC01P031501 |
| MYCN | MYCN Proto-Oncogene, BHLH Transcription Factor | GC02P015950 |
| APOB | Apolipoprotein B | GC02M020956 |
| NSD1 | Nuclear Receptor Binding SET Domain Protein 1 | GC05P177134 |
| NRP1 | Neuropilin 1 | GC10M033177 |
| CCND3 | Cyclin D3 | GC06M041934 |
| SUFU | SUFU Negative Regulator Of Hedgehog Signaling | GC10P102503 |
| RACK1 | Receptor For Activated C Kinase 1 | GC05M181286 |
| XBP1 | X-Box Binding Protein 1 | GC22M028794 |
| C14orf132 | Chromosome 14 Open Reading Frame 132 | GC14P096040 |
| MIR27B | MicroRNA 27b | GC09P095098 |
| POSTN | Periostin | GC13M037562 |
| RAB4B-EGLN2 | RAB4B-EGLN2 Readthrough (NMD Candidate) | GC19P040778 |
| COMT | Catechol-O-Methyltransferase | GC22P019941 |
| IL6ST | Interleukin 6 Signal Transducer | GC05M055935 |
| SLC10A2 | Solute Carrier Family 10 Member 2 | GC13M103043 |
| MIR98 | MicroRNA 98 | GC0XM053576 |
| F3 | Coagulation Factor III, Tissue Factor | GC01M094530 |
| LINC00346 | Long Intergenic Non-Protein Coding RNA 346 | GC13M110863 |
| BMP4 | Bone Morphogenetic Protein 4 | GC14M053949 |
| SLPI | Secretory Leukocyte Peptidase Inhibitor | GC20M045252 |
| CXCR2 | C-X-C Motif Chemokine Receptor 2 | GC02P218125 |
| CLDN4 | Claudin 4 | GC07P073799 |
| PML | Promyelocytic Leukemia | GC15P073994 |
| PRC1-AS1 | PRC1 Antisense RNA 1 | GC15P090966 |
| ICOSLG | Inducible T Cell Costimulator Ligand | GC21M044222 |
| PPARA | Peroxisome Proliferator Activated Receptor Alpha | GC22P046150 |
| CD40LG | CD40 Ligand | GC0XP136649 |
| ANXA1 | Annexin A1 | GC09P073151 |
| LMNA | Lamin A/C | GC01P156082 |
| TAP1 | Transporter 1, ATP Binding Cassette Subfamily B Member | GC06M032866 |
| S100B | S100 Calcium Binding Protein B | GC21M047166 |
| AGER | Advanced Glycosylation End-Product Specific Receptor | GC06M032180 |
| HSPD1 | Heat Shock Protein Family D (Hsp60) Member 1 | GC02M197486 |
| ELN | Elastin | GC07P074027 |
| HLA-DQA1 | Major Histocompatibility Complex, Class II, DQ Alpha 1 | GC06P033442 |
| LINC00941 | Long Intergenic Non-Protein Coding RNA 941 | GC12P030757 |
| AIFM1 | Apoptosis Inducing Factor Mitochondria Associated 1 | GC0XM130129 |
| GATA3 | GATA Binding Protein 3 | GC10P008045 |
| SLC7A6 | Solute Carrier Family 7 Member 6 | GC16P068263 |
| DLK1 | Delta Like Non-Canonical Notch Ligand 1 | GC14P104336 |
| CP | Ceruloplasmin | GC03M149162 |
| POMC | Proopiomelanocortin | GC02M025160 |
| ILK | Integrin Linked Kinase | GC11P006581 |
| NARF-AS1 | NARF Antisense RNA 1 | GC17M082477 |
| ENSG00000278769 |  | GC15P042724 |
| CDC42 | Cell Division Cycle 42 | GC01P022043 |
| VLDLR-AS1 | VLDLR Antisense RNA 1 | GC09M002411 |
| KIF1B | Kinesin Family Member 1B | GC01P010210 |
| EIF4EBP1 | Eukaryotic Translation Initiation Factor 4E Binding Protein 1 | GC08P038007 |
| IATPR | ITGB1 Adjacent Tumor Promoting LncRNA | GC10M033073 |
| SLC7A5 | Solute Carrier Family 7 Member 5 | GC16M087830 |
| NRG1 | Neuregulin 1 | GC08P031639 |
| MLANA | Melan-A | GC09P005846 |
| UFC1 | Ubiquitin-Fold Modifier Conjugating Enzyme 1 | GC01P161152 |
| DES | Desmin | GC02P219418 |
| BARD1 | BRCA1 Associated RING Domain 1 | GC02M214725 |
| IRS2 | Insulin Receptor Substrate 2 | GC13M109752 |
| YBX1 | Y-Box Binding Protein 1 | GC01P042682 |
| RNF6 | Ring Finger Protein 6 | GC13M026132 |
| GREM1 | Gremlin 1, DAN Family BMP Antagonist | GC15P032717 |
| ENSG00000250899 |  | GC12P003041 |
| GRB7 | Growth Factor Receptor Bound Protein 7 | GC17P039744 |
| PTPRG | Protein Tyrosine Phosphatase Receptor Type G | GC03P061522 |
| STEAP4 | STEAP4 Metalloreductase | GC07M088278 |
| MIR331 | MicroRNA 331 | GC12P095308 |
| MIF | Macrophage Migration Inhibitory Factor | GC22P023894 |
| TNFRSF11B | TNF Receptor Superfamily Member 11b | GC08M118923 |
| LINC00589 | Long Intergenic Non-Protein Coding RNA 589 | GC08M029673 |
| KITLG | KIT Ligand | GC12M088492 |
| PTGS1 | Prostaglandin-Endoperoxide Synthase 1 | GC09P122370 |
| PECAM1 | Platelet And Endothelial Cell Adhesion Molecule 1 | GC17M064319 |
| SDHAF2 | Succinate Dehydrogenase Complex Assembly Factor 2 | GC11P061430 |
| MCU | Mitochondrial Calcium Uniporter | GC10P072692 |
| HES1 | Hes Family BHLH Transcription Factor 1 | GC03P194136 |
| CCN1 | Cellular Communication Network Factor 1 | GC01P085581 |
| DDR1 | Discoidin Domain Receptor Tyrosine Kinase 1 | GC06P033371 |
| CCND2 | Cyclin D2 | GC12P006576 |
| APEX1 | Apurinic/Apyrimidinic Endodeoxyribonuclease 1 | GC14P020455 |
| NDRG1 | N-Myc Downstream Regulated 1 | GC08M133237 |
| PRKN | Parkin RBR E3 Ubiquitin Protein Ligase | GC06M161348 |
| PIN1 | Peptidylprolyl Cis/Trans Isomerase, NIMA-Interacting 1 | GC19P009835 |
| APOE | Apolipoprotein E | GC19P044906 |
| EPHA3 | EPH Receptor A3 | GC03P089077 |
| TRIM24 | Tripartite Motif Containing 24 | GC07P138460 |
| TNFSF15 | TNF Superfamily Member 15 | GC09M114784 |
| TMEM51-AS1 | TMEM51 Antisense RNA 1 | GC01M015111 |
| ATG5 | Autophagy Related 5 | GC06M106045 |
| MMP10 | Matrix Metallopeptidase 10 | GC11M102770 |
| SIRT1-AS | SIRT1 Antisense RNA | GC10U902480 |
| CSF1R | Colony Stimulating Factor 1 Receptor | GC05M150053 |
| MIR342 | MicroRNA 342 | GC14P100109 |
| TFAP2A | Transcription Factor AP-2 Alpha | GC06M010393 |
| ABCC4 | ATP Binding Cassette Subfamily C Member 4 | GC13M095019 |
| MUC6 | Mucin 6, Oligomeric Mucus/Gel-Forming | GC11M001002 |
| CEL | Carboxyl Ester Lipase | GC09P133061 |
| MT1DP | Metallothionein 1D, Pseudogene | GC16P056643 |
| PSG2 | Pregnancy Specific Beta-1-Glycoprotein 2 | GC19M043064 |
| CSF1 | Colony Stimulating Factor 1 | GC01P109911 |
| CNC2 | Carney Complex Type 2, Multiple Neoplasia And Lentiginosis | GC02U990267 |
| DDB2 | Damage Specific DNA Binding Protein 2 | GC11P047237 |
| PSMB8 | Proteasome 20S Subunit Beta 8 | GC06M032840 |
| LINC00554 | Long Intergenic Non-Protein Coding RNA 554 | GC13M099994 |
| NONO | Non-POU Domain Containing Octamer Binding | GC0XP071283 |
| ENSG00000266990 |  | GC19M000999 |
| TNPO3 | Transportin 3 | GC07M128954 |
| MIR372 | MicroRNA 372 | GC19P054120 |
| BID | BH3 Interacting Domain Death Agonist | GC22M017734 |
| PPP2R1A | Protein Phosphatase 2 Scaffold Subunit Aalpha | GC19P052189 |
| VTN | Vitronectin | GC17M029696 |
| HDAC2 | Histone Deacetylase 2 | GC06M113933 |
| IL1RN | Interleukin 1 Receptor Antagonist | GC02P114906 |
| MYB | MYB Proto-Oncogene, Transcription Factor | GC06P135180 |
| ECHS1 | Enoyl-CoA Hydratase, Short Chain 1 | GC10M133362 |
| BCL10 | BCL10 Immune Signaling Adaptor | GC01M085265 |
| GPR35 | G Protein-Coupled Receptor 35 | GC02P240605 |
| MCM2 | Minichromosome Maintenance Complex Component 2 | GC03P127598 |
| TCN1 | Transcobalamin 1 | GC11M060026 |
| PEBP1 | Phosphatidylethanolamine Binding Protein 1 | GC12P118135 |
| KCTD13 | Potassium Channel Tetramerization Domain Containing 13 | GC16M029905 |
| SLC25A27 | Solute Carrier Family 25 Member 27 | GC06P046652 |
| KRT13 | Keratin 13 | GC17M041500 |
| GJB2 | Gap Junction Protein Beta 2 | GC13M020187 |
| SLCO1B1 | Solute Carrier Organic Anion Transporter Family Member 1B1 | GC12P021132 |
| MAD1L1 | Mitotic Arrest Deficient 1 Like 1 | GC07M001815 |
| CDR1 | Cerebellar Degeneration Related Protein 1 | GC0XM140782 |
| CADM1 | Cell Adhesion Molecule 1 | GC11M115169 |
| SERPINB4 | Serpin Family B Member 4 | GC18M063637 |
| ARC | Activity Regulated Cytoskeleton Associated Protein | GC08M142611 |
| NCOA3 | Nuclear Receptor Coactivator 3 | GC20P047501 |
| ERCC4 | ERCC Excision Repair 4, Endonuclease Catalytic Subunit | GC16P013920 |
| MIR100HG | Mir-100-Let-7a-2-Mir-125b-1 Cluster Host Gene | GC11M122029 |
| HNRNPK | Heterogeneous Nuclear Ribonucleoprotein K | GC09M083992 |
| CAMTA1 | Calmodulin Binding Transcription Activator 1 | GC01P006845 |
| SERHL | Serine Hydrolase Like (Pseudogene) | GC22P042500 |
| BAIAP2-DT | BAIAP2 Divergent Transcript | GC17M081042 |
| MIR320A | MicroRNA 320a | GC08M022245 |
| SFPQ | Splicing Factor Proline And Glutamine Rich | GC01M035176 |
| LAMB3 | Laminin Subunit Beta 3 | GC01M209614 |
| HOXB13 | Homeobox B13 | GC17M048725 |
| KRT17 | Keratin 17 | GC17M041619 |
| BPIFA1 | BPI Fold Containing Family A Member 1 | GC20P033235 |
| DDX3X | DEAD-Box Helicase 3 X-Linked | GC0XP041333 |
| ETV6 | ETS Variant Transcription Factor 6 | GC12P011649 |
| POU2AF1 | POU Class 2 Homeobox Associating Factor 1 | GC11M111352 |
| TSHR | Thyroid Stimulating Hormone Receptor | GC14P080954 |
| EDN1 | Endothelin 1 | GC06P012290 |
| PRPSAP1 | Phosphoribosyl Pyrophosphate Synthetase Associated Protein 1 | GC17M076309 |
| KRTAP5-AS1 | KRTAP5-1/KRTAP5-2 Antisense RNA 1 | GC11P001571 |
| FAM83A-AS1 | FAM83A Antisense RNA 1 | GC08M123201 |
| LINC00383 | Long Intergenic Non-Protein Coding RNA 383 | GC13P069222 |
| CCND3P1 | Cyclin D3 Pseudogene 1 | GC10M030403 |
| ENSG00000258798 |  | GC14P091418 |
| CECR7 | Cat Eye Syndrome Chromosome Region, Candidate 7 | GC22P017068 |
| MIR378A | MicroRNA 378a | GC05P149732 |
| MAPK10 | Mitogen-Activated Protein Kinase 10 | GC04M085990 |
| CYP2A6 | Cytochrome P450 Family 2 Subfamily A Member 6 | GC19M040843 |
| KRT10 | Keratin 10 | GC17M040818 |
| MIR206 | MicroRNA 206 | GC06P052144 |
| KCNQ1 | Potassium Voltage-Gated Channel Subfamily Q Member 1 | GC11P002444 |
| FANCC | FA Complementation Group C | GC09M095099 |
| HLA-C | Major Histocompatibility Complex, Class I, C | GC06M031272 |
| IDO1 | Indoleamine 2,3-Dioxygenase 1 | GC08P039891 |
| LDHA | Lactate Dehydrogenase A | GC11P018394 |
| NPC1 | NPC Intracellular Cholesterol Transporter 1 | GC18M023506 |
| TXN | Thioredoxin | GC09M110243 |
| MICB | MHC Class I Polypeptide-Related Sequence B | GC06P033390 |
| INHBA | Inhibin Subunit Beta A | GC07M041668 |
| PAK1 | P21 (RAC1) Activated Kinase 1 | GC11M077321 |
| RARS1 | Arginyl-TRNA Synthetase 1 | GC05P168487 |
| IVL | Involucrin | GC01P152881 |
| NAPSA | Napsin A Aspartic Peptidase | GC19M050383 |
| NR1I2 | Nuclear Receptor Subfamily 1 Group I Member 2 | GC03P119780 |
| PFKFB1 | 6-Phosphofructo-2-Kinase/Fructose-2,6-Biphosphatase 1 | GC0XM054932 |
| GLT1D1 | Glycosyltransferase 1 Domain Containing 1 | GC12P128853 |
| URB2 | URB2 Ribosome Biogenesis Homolog | GC01P229626 |
| PRR26 | Proline Rich 26 | GC10P000649 |
| FABP5P3 | Fatty Acid Binding Protein 5 Pseudogene 3 | GC07P152436 |
| LINC00173 | Long Intergenic Non-Protein Coding RNA 173 | GC12P116533 |
| MAPKAPK5-AS1 | MAPKAPK5 Antisense RNA 1 | GC12M111839 |
| LINC00926 | Long Intergenic Non-Protein Coding RNA 926 | GC15P057300 |
| HTR2A-AS1 | HTR2A Antisense RNA 1 | GC13P046852 |
| OVCH1-AS1 | OVCH1 Antisense RNA 1 | GC12P029389 |
| SEMA6A-AS1 | SEMA6A Antisense RNA 1 | GC05P116447 |
| CA3-AS1 | CA3 Antisense RNA 1 | GC08M085442 |
| LINC00601 | Long Intergenic Non-Protein Coding RNA 601 | GC10M126413 |
| ULK4P2 | ULK4 Pseudogene 2 | GC15P030572 |
| LINC01419 | Long Intergenic Non-Protein Coding RNA 1419 | GC08P083403 |
| PERCC1 | Proline And Glutamate Rich With Coiled Coil 1 | GC16P001431 |
| ENSG00000224086 |  | GC22P021938 |
| ENSG00000228363 |  | GC02P086562 |
| ENSG00000259488 |  | GC15M048312 |
| ENSG00000258711 |  | GC14M050956 |
| ENSG00000272953 |  | GC07P005426 |
| ENSG00000265778 |  | GC18P076492 |
| ENSG00000145063 |  | GC02M011105 |
| CXCL1P1 | C-X-C Motif Chemokine Ligand 1 Pseudogene 1 | GC04M073944 |
| ENSG00000279159 |  | GC22M029978 |
| LOC105369388 | Uncharacterized LOC105369388 | GC11M075205 |
| SART1 | Spliceosome Associated Factor 1, Recruiter Of U4/U6.U5 Tri-SnRNP | GC11P065979 |
| XRCC6 | X-Ray Repair Cross Complementing 6 | GC22P041622 |
| CEACAM6 | CEA Cell Adhesion Molecule 6 | GC19P041750 |
| BGLAP | Bone Gamma-Carboxyglutamate Protein | GC01P156242 |
| SPIB | Spi-B Transcription Factor | GC19P050418 |
| HPX | Hemopexin | GC11M006435 |
| HMGA1 | High Mobility Group AT-Hook 1 | GC06P046013 |
| GH1 | Growth Hormone 1 | GC17M063917 |
| BAG1 | BAG Cochaperone 1 | GC09M033245 |
| KISS1 | KiSS-1 Metastasis Suppressor | GC01M204190 |
| GLI2 | GLI Family Zinc Finger 2 | GC02P120735 |
| PTTG1 | PTTG1 Regulator Of Sister Chromatid Separation, Securin | GC05P160422 |
| IQGAP1 | IQ Motif Containing GTPase Activating Protein 1 | GC15P090388 |
| FOSL1 | FOS Like 1, AP-1 Transcription Factor Subunit | GC11M065909 |
| TEK | TEK Receptor Tyrosine Kinase | GC09P027109 |
| ADAM10 | ADAM Metallopeptidase Domain 10 | GC15M058588 |
| ATR | ATR Serine/Threonine Kinase | GC03M142449 |
| XRCC5 | X-Ray Repair Cross Complementing 5 | GC02P216107 |
| FST | Follistatin | GC05P053480 |
| NTRK2 | Neurotrophic Receptor Tyrosine Kinase 2 | GC09P084668 |
| MXI1 | MAX Interactor 1, Dimerization Protein | GC10P110208 |
| S100A7 | S100 Calcium Binding Protein A7 | GC01M153457 |
| MIR187 | MicroRNA 187 | GC18M035904 |
| LOC110806263 | TERT 5' Regulatory Region | GC05U902905 |
| PRKDC | Protein Kinase, DNA-Activated, Catalytic Subunit | GC08M047773 |
| AGR2 | Anterior Gradient 2, Protein Disulphide Isomerase Family Member | GC07M016818 |
| SFN | Stratifin | GC01P026873 |
| ELANE | Elastase, Neutrophil Expressed | GC19P000854 |
| MMEL1 | Membrane Metalloendopeptidase Like 1 | GC01M002590 |
| DMBT1 | Deleted In Malignant Brain Tumors 1 | GC10P122560 |
| CCNG1 | Cyclin G1 | GC05P163457 |
| FGF1 | Fibroblast Growth Factor 1 | GC05M142555 |
| EXO1 | Exonuclease 1 | GC01P241847 |
| LCN2 | Lipocalin 2 | GC09P128149 |
| ID1 | Inhibitor Of DNA Binding 1, HLH Protein | GC20P031605 |
| LNCARSR | LncRNA Regulator Of Akt Signaling Associated With HCC And RCC | GC09M079507 |
| GSN | Gelsolin | GC09P121207 |
| INHA | Inhibin Subunit Alpha | GC02P219569 |
| CD59 | CD59 Molecule (CD59 Blood Group) | GC11M033704 |
| PARK7 | Parkinsonism Associated Deglycase | GC01P007968 |
| MACC1 | MET Transcriptional Regulator MACC1 | GC07M020140 |
| BIRC7 | Baculoviral IAP Repeat Containing 7 | GC20P063235 |
| NAMPT | Nicotinamide Phosphoribosyltransferase | GC07M106248 |
| LOC111832670 | Alpha Fetoprotein (AFP) 5' Regulatory Region | GC04U902770 |
| ADH1B | Alcohol Dehydrogenase 1B (Class I), Beta Polypeptide | GC04M099304 |
| TUBB | Tubulin Beta Class I | GC06P030720 |
| REG1A | Regenerating Family Member 1 Alpha | GC02P079120 |
| JAK3 | Janus Kinase 3 | GC19M017824 |
| FGF7 | Fibroblast Growth Factor 7 | GC15P049423 |
| ACCS | 1-Aminocyclopropane-1-Carboxylate Synthase Homolog (Inactive) | GC11P044045 |
| MIR95 | MicroRNA 95 | GC04M008007 |
| CDC25C | Cell Division Cycle 25C | GC05M138285 |
| ANPEP | Alanyl Aminopeptidase, Membrane | GC15M089784 |
| PPP2R1B | Protein Phosphatase 2 Scaffold Subunit Abeta | GC11M111695 |
| G6PD | Glucose-6-Phosphate Dehydrogenase | GC0XM154531 |
| CDKN2C | Cyclin Dependent Kinase Inhibitor 2C | GC01P050960 |
| CA2 | Carbonic Anhydrase 2 | GC08P085463 |
| TDGF1 | Teratocarcinoma-Derived Growth Factor 1 | GC03P046576 |
| PRDX1 | Peroxiredoxin 1 | GC01M045511 |
| MIR193B | MicroRNA 193b | GC16P014307 |
| PPIA | Peptidylprolyl Isomerase A | GC07P044808 |
| CEACAM7 | CEA Cell Adhesion Molecule 7 | GC19M041673 |
| TFF3 | Trefoil Factor 3 | GC21M042311 |
| LOXL2 | Lysyl Oxidase Like 2 | GC08M023296 |
| GRN | Granulin Precursor | GC17P044345 |
| H2AX | H2A.X Variant Histone | GC11M119097 |
| GHRH | Growth Hormone Releasing Hormone | GC20M037251 |
| MIR154 | MicroRNA 154 | GC14P104438 |
| DUSP1 | Dual Specificity Phosphatase 1 | GC05M172768 |
| GATA6 | GATA Binding Protein 6 | GC18P022169 |
| EPHB4 | EPH Receptor B4 | GC07M100803 |
| WIF1 | WNT Inhibitory Factor 1 | GC12M065050 |
| CYP7A1 | Cytochrome P450 Family 7 Subfamily A Member 1 | GC08M058476 |
| STAT4 | Signal Transducer And Activator Of Transcription 4 | GC02M191029 |
| MIR497 | MicroRNA 497 | GC17M007022 |
| CXCL1 | C-X-C Motif Chemokine Ligand 1 | GC04P073869 |
| NR3C1 | Nuclear Receptor Subfamily 3 Group C Member 1 | GC05M143277 |
| ACTA2 | Actin Alpha 2, Smooth Muscle | GC10M088935 |
| TNC | Tenascin C | GC09M115019 |
| SDC1 | Syndecan 1 | GC02M020200 |
| ROS1 | ROS Proto-Oncogene 1, Receptor Tyrosine Kinase | GC06M117287 |
| GCG | Glucagon | GC02M162142 |
| OSMR | Oncostatin M Receptor | GC05P038845 |
| SELE | Selectin E | GC01M169722 |
| SERPINB2 | Serpin Family B Member 2 | GC18P063871 |
| FAP | Fibroblast Activation Protein Alpha | GC02M162170 |
| HBB | Hemoglobin Subunit Beta | GC11M005334 |
| SOX4 | SRY-Box Transcription Factor 4 | GC06P021593 |
| ETV4 | ETS Variant Transcription Factor 4 | GC17M043527 |
| PRKAA1 | Protein Kinase AMP-Activated Catalytic Subunit Alpha 1 | GC05M040759 |
| SPINK1 | Serine Peptidase Inhibitor Kazal Type 1 | GC05M147825 |
| EBAG9 | Estrogen Receptor Binding Site Associated Antigen 9 | GC08P109536 |
| HPRT1 | Hypoxanthine Phosphoribosyltransferase 1 | GC0XP134460 |
| MAP3K5 | Mitogen-Activated Protein Kinase Kinase Kinase 5 | GC06M136557 |
| MBL2 | Mannose Binding Lectin 2 | GC10M052760 |
| VEGFB | Vascular Endothelial Growth Factor B | GC11P064234 |
| TPO | Thyroid Peroxidase | GC02P001374 |
| PGF | Placental Growth Factor | GC14M074941 |
| MIR138-1 | MicroRNA 138-1 | GC03P044115 |
| ING3 | Inhibitor Of Growth Family Member 3 | GC07P120950 |
| SERPINC1 | Serpin Family C Member 1 | GC01M174131 |
| MAD2L1 | Mitotic Arrest Deficient 2 Like 1 | GC04M120055 |
| EPOR | Erythropoietin Receptor | GC19M011377 |
| CKS1B | CDC28 Protein Kinase Regulatory Subunit 1B | GC01P154974 |
| LDLR | Low Density Lipoprotein Receptor | GC19P011061 |
| MIA2 | MIA SH3 Domain ER Export Factor 2 | GC14P039230 |
| TFPI2 | Tissue Factor Pathway Inhibitor 2 | GC07M093885 |
| RSPO1 | R-Spondin 1 | GC01M037612 |
| APAF1 | Apoptotic Peptidase Activating Factor 1 | GC12P098645 |
| PRSS1 | Serine Protease 1 | GC07P144706 |
| CPOX | Coproporphyrinogen Oxidase | GC03M098576 |
| DHFR | Dihydrofolate Reductase | GC05M080626 |
| ITGA3 | Integrin Subunit Alpha 3 | GC17P050055 |
| DKC1 | Dyskerin Pseudouridine Synthase 1 | GC0XP154762 |
| MIR424 | MicroRNA 424 | GC0XM134625 |
| MIR361 | MicroRNA 361 | GC0XM085903 |
| SYK | Spleen Associated Tyrosine Kinase | GC09P091113 |
| PLIN2 | Perilipin 2 | GC09M019112 |
| IREB2 | Iron Responsive Element Binding Protein 2 | GC15P078437 |
| TIAM1 | TIAM Rac1 Associated GEF 1 | GC21M031118 |
| PSMB9 | Proteasome 20S Subunit Beta 9 | GC06P033443 |
| CUL2 | Cullin 2 | GC10M035046 |
| ELOC | Elongin C | GC08M073939 |
| RPSA | Ribosomal Protein SA | GC03P039406 |
| PDGFB | Platelet Derived Growth Factor Subunit B | GC22M044780 |
| HLA-DPB1 | Major Histocompatibility Complex, Class II, DP Beta 1 | GC06P033447 |
| UMPS | Uridine Monophosphate Synthetase | GC03P124730 |
| IKBKG | Inhibitor Of Nuclear Factor Kappa B Kinase Regulatory Subunit Gamma | GC0XP154541 |
| CFTR | CF Transmembrane Conductance Regulator | GC07P117465 |
| LTA | Lymphotoxin Alpha | GC06P033395 |
| CEBPA | CCAAT Enhancer Binding Protein Alpha | GC19M033299 |
| MOK | MOK Protein Kinase | GC14M102224 |
| F2R | Coagulation Factor II Thrombin Receptor | GC05P076716 |
| LAMB1 | Laminin Subunit Beta 1 | GC07M107923 |
| HHEX | Hematopoietically Expressed Homeobox | GC10P092689 |
| DOCK8 | Dedicator Of Cytokinesis 8 | GC09P000214 |
| ROCK2 | Rho Associated Coiled-Coil Containing Protein Kinase 2 | GC02M011181 |
| CLTC | Clathrin Heavy Chain | GC17P059619 |
| ADAR | Adenosine Deaminase RNA Specific | GC01M154582 |
| ARMC5 | Armadillo Repeat Containing 5 | GC16P031477 |
| TSG101 | Tumor Susceptibility 101 | GC11M018468 |
| NES | Nestin | GC01M156668 |
| NGFR | Nerve Growth Factor Receptor | GC17P049495 |
| NEUROD1 | Neuronal Differentiation 1 | GC02M181673 |
| CHRNA3 | Cholinergic Receptor Nicotinic Alpha 3 Subunit | GC15M078594 |
| UHRF1 | Ubiquitin Like With PHD And Ring Finger Domains 1 | GC19P004910 |
| BIRC2 | Baculoviral IAP Repeat Containing 2 | GC11P102347 |
| MAPK9 | Mitogen-Activated Protein Kinase 9 | GC05M180234 |
| SPINT2 | Serine Peptidase Inhibitor, Kunitz Type 2 | GC19P038244 |
| DNAH8 | Dynein Axonemal Heavy Chain 8 | GC06P046078 |
| NF2 | Neurofibromin 2 | GC22P029603 |
| TGIF1 | TGFB Induced Factor Homeobox 1 | GC18P003411 |
| CD9 | CD9 Molecule | GC12P006594 |
| PPARGC1A | PPARG Coactivator 1 Alpha | GC04M023755 |
| RUNX2 | RUNX Family Transcription Factor 2 | GC06P045327 |
| MMP8 | Matrix Metallopeptidase 8 | GC11M102617 |
| PHKA2 | Phosphorylase Kinase Regulatory Subunit Alpha 2 | GC0XM018892 |
| EWSR1 | EWS RNA Binding Protein 1 | GC22P029269 |
| CTSL | Cathepsin L | GC09P087725 |
| BNIP3 | BCL2 Interacting Protein 3 | GC10M131966 |
| MIR377 | MicroRNA 377 | GC14P104454 |
| CD151 | CD151 Molecule (Raph Blood Group) | GC11P000883 |
| CYP2C9 | Cytochrome P450 Family 2 Subfamily C Member 9 | GC10P094938 |
| ERG | ETS Transcription Factor ERG | GC21M038367 |
| APOBEC3G | Apolipoprotein B MRNA Editing Enzyme Catalytic Subunit 3G | GC22P039078 |
| OTC | Ornithine Carbamoyltransferase | GC0XP038353 |
| RARRES2 | Retinoic Acid Receptor Responder 2 | GC07M150333 |
| DKK3 | Dickkopf WNT Signaling Pathway Inhibitor 3 | GC11M011962 |
| RBP1 | Retinol Binding Protein 1 | GC03M139517 |
| S100A6 | S100 Calcium Binding Protein A6 | GC01M153535 |
| MIR326 | MicroRNA 326 | GC11M075335 |
| CIP2A | Cellular Inhibitor Of PP2A | GC03M108545 |
| MAP3K1 | Mitogen-Activated Protein Kinase Kinase Kinase 1 | GC05P056815 |
| DLL4 | Delta Like Canonical Notch Ligand 4 | GC15P040929 |
| PRKACB | Protein Kinase CAMP-Activated Catalytic Subunit Beta | GC01P084078 |
| U2AF1 | U2 Small Nuclear RNA Auxiliary Factor 1 | GC21M043092 |
| ERCC3 | ERCC Excision Repair 3, TFIIH Core Complex Helicase Subunit | GC02M127257 |
| GOLGA6L2 | Golgin A6 Family Like 2 | GC15M023440 |
| CXCR5 | C-X-C Motif Chemokine Receptor 5 | GC11P118892 |
| CLDN3 | Claudin 3 | GC07M073768 |
| ACKR3 | Atypical Chemokine Receptor 3 | GC02P236537 |
| THBD | Thrombomodulin | GC20M023026 |
| ABL1 | ABL Proto-Oncogene 1, Non-Receptor Tyrosine Kinase | GC09P130713 |
| MIR7-1 | MicroRNA 7-1 | GC09M083993 |
| PLG | Plasminogen | GC06P160702 |
| SHBG | Sex Hormone Binding Globulin | GC17P007613 |
| ADH1C | Alcohol Dehydrogenase 1C (Class I), Gamma Polypeptide | GC04M099336 |
| IL24 | Interleukin 24 | GC01P206897 |
| ROBO1 | Roundabout Guidance Receptor 1 | GC03M078597 |
| MAX | MYC Associated Factor X | GC14M065009 |
| ALAD | Aminolevulinate Dehydratase | GC09M113386 |
| STAT5B | Signal Transducer And Activator Of Transcription 5B | GC17M042199 |
| MIR328 | MicroRNA 328 | GC16M067203 |
| KDM1A | Lysine Demethylase 1A | GC01P023019 |
| MAP2K4 | Mitogen-Activated Protein Kinase Kinase 4 | GC17P012020 |
| SLC22A18 | Solute Carrier Family 22 Member 18 | GC11P002899 |
| SCARB1 | Scavenger Receptor Class B Member 1 | GC12M124776 |
| PTGER4 | Prostaglandin E Receptor 4 | GC05P040679 |
| F2RL1 | F2R Like Trypsin Receptor 1 | GC05P076818 |
| PCLAF | PCNA Clamp Associated Factor | GC15M064549 |
| KCNJ11 | Potassium Inwardly Rectifying Channel Subfamily J Member 11 | GC11M017364 |
| SRD5A2 | Steroid 5 Alpha-Reductase 2 | GC02M031522 |
| TAT | Tyrosine Aminotransferase | GC16M071565 |
| CSNK2A1 | Casein Kinase 2 Alpha 1 | GC20M000472 |
| WWTR1 | WW Domain Containing Transcription Regulator 1 | GC03M149517 |
| SPHK1 | Sphingosine Kinase 1 | GC17P076376 |
| EGLN3 | Egl-9 Family Hypoxia Inducible Factor 3 | GC14M033924 |
| NAT1 | N-Acetyltransferase 1 | GC08P018180 |
| SLC5A5 | Solute Carrier Family 5 Member 5 | GC19P022240 |
| KRT1 | Keratin 1 | GC12M052674 |
| MIR370 | MicroRNA 370 | GC14P104253 |
| UTP6 | UTP6 Small Subunit Processome Component | GC17M031860 |
| LGALS3BP | Galectin 3 Binding Protein | GC17M078971 |
| GRPR | Gastrin Releasing Peptide Receptor | GC0XP016141 |
| KISS1R | KISS1 Receptor | GC19P000917 |
| VCAM1 | Vascular Cell Adhesion Molecule 1 | GC01P100719 |
| SSTR5 | Somatostatin Receptor 5 | GC16P001072 |
| COL4A2 | Collagen Type IV Alpha 2 Chain | GC13P110305 |
| GFER | Growth Factor, Augmenter Of Liver Regeneration | GC16P001984 |
| ELOB | Elongin B | GC16M002772 |
| CLPTM1L | CLPTM1 Like | GC05M001317 |
| MIR218-1 | MicroRNA 218-1 | GC04P020668 |
| AQP1 | Aquaporin 1 (Colton Blood Group) | GC07P030911 |
| MIR503 | MicroRNA 503 | GC0XM134624 |
| PLCE1 | Phospholipase C Epsilon 1 | GC10P093993 |
| TACSTD2 | Tumor Associated Calcium Signal Transducer 2 | GC01M058575 |
| CDC25B | Cell Division Cycle 25B | GC20P003787 |
| CCN4 | Cellular Communication Network Factor 4 | GC08P133192 |
| TTN | Titin | GC02M178525 |
| CEBPB | CCAAT Enhancer Binding Protein Beta | GC20P050190 |
| FGF4 | Fibroblast Growth Factor 4 | GC11M069762 |
| MIR532 | MicroRNA 532 | GC0XP050004 |
| MIR29B1 | MicroRNA 29b-1 | GC07M130877 |
| PDGFA | Platelet Derived Growth Factor Subunit A | GC07M000497 |
| SATB1 | SATB Homeobox 1 | GC03M018364 |
| MCM7 | Minichromosome Maintenance Complex Component 7 | GC07M100092 |
| TNFRSF10D | TNF Receptor Superfamily Member 10d | GC08M023135 |
| IL4R | Interleukin 4 Receptor | GC16P027325 |
| LASP1 | LIM And SH3 Protein 1 | GC17P038869 |
| S100A8 | S100 Calcium Binding Protein A8 | GC01M153391 |
| ATP11A | ATPase Phospholipid Transporting 11A | GC13P112690 |
| SAT1 | Spermidine/Spermine N1-Acetyltransferase 1 | GC0XP023784 |
| SSTR1 | Somatostatin Receptor 1 | GC14P038207 |
| HPD | 4-Hydroxyphenylpyruvate Dioxygenase | GC12M121839 |
| S100A9 | S100 Calcium Binding Protein A9 | GC01P153357 |
| SREBF2 | Sterol Regulatory Element Binding Transcription Factor 2 | GC22P041833 |
| STC2 | Stanniocalcin 2 | GC05M173314 |
| LYVE1 | Lymphatic Vessel Endothelial Hyaluronan Receptor 1 | GC11M010636 |
| IL15 | Interleukin 15 | GC04P141636 |
| MIR135A1 | MicroRNA 135a-1 | GC03M052296 |
| TP53BP2 | Tumor Protein P53 Binding Protein 2 | GC01M223779 |
| MIR128-1 | MicroRNA 128-1 | GC02P135665 |
| ETS2 | ETS Proto-Oncogene 2, Transcription Factor | GC21P038805 |
| ADAM12 | ADAM Metallopeptidase Domain 12 | GC10M126012 |
| NCOR1 | Nuclear Receptor Corepressor 1 | GC17M016029 |
| RAD23B | RAD23 Homolog B, Nucleotide Excision Repair Protein | GC09P107283 |
| ADAM17 | ADAM Metallopeptidase Domain 17 | GC02M009488 |
| DDX58 | DExD/H-Box Helicase 58 | GC09M032455 |
| SELENBP1 | Selenium Binding Protein 1 | GC01M151364 |
| BRMS1 | BRMS1 Transcriptional Repressor And Anoikis Regulator | GC11M066346 |
| NOS3 | Nitric Oxide Synthase 3 | GC07P150990 |
| MIR9-3 | MicroRNA 9-3 | GC15P089363 |
| PDPK1 | 3-Phosphoinositide Dependent Protein Kinase 1 | GC16P002537 |
| LATS1 | Large Tumor Suppressor Kinase 1 | GC06M149658 |
| S100A2 | S100 Calcium Binding Protein A2 | GC01M153561 |
| CDC6 | Cell Division Cycle 6 | GC17P040287 |
| FABP4 | Fatty Acid Binding Protein 4 | GC08M081478 |
| AFAP1 | Actin Filament Associated Protein 1 | GC04M007758 |
| MIR584 | MicroRNA 584 | GC05M149062 |
| RIPK1 | Receptor Interacting Serine/Threonine Kinase 1 | GC06P003064 |
| CSK | C-Terminal Src Kinase | GC15P074782 |
| NCOA2 | Nuclear Receptor Coactivator 2 | GC08M070109 |
| NNMT | Nicotinamide N-Methyltransferase | GC11P114257 |
| C7orf50 | Chromosome 7 Open Reading Frame 50 | GC07M000996 |
| SLC39A1 | Solute Carrier Family 39 Member 1 | GC01M153960 |
| MIR299 | MicroRNA 299 | GC14P104440 |
| ZFHX3 | Zinc Finger Homeobox 3 | GC16M072782 |
| MSMB | Microseminoprotein Beta | GC10M046033 |
| MIR130B | MicroRNA 130b | GC22P023989 |
| STAT5A | Signal Transducer And Activator Of Transcription 5A | GC17P042287 |
| USP53 | Ubiquitin Specific Peptidase 53 | GC04P119212 |
| B3GAT1 | Beta-1,3-Glucuronyltransferase 1 | GC11M134378 |
| COL18A1 | Collagen Type XVIII Alpha 1 Chain | GC21P045405 |
| BMP7 | Bone Morphogenetic Protein 7 | GC20M057168 |
| CD276 | CD276 Molecule | GC15P073683 |
| TMPRSS6 | Transmembrane Serine Protease 6 | GC22M037066 |
| PTPN12 | Protein Tyrosine Phosphatase Non-Receptor Type 12 | GC07P077537 |
| TNFAIP3 | TNF Alpha Induced Protein 3 | GC06P137866 |
| EGLN1 | Egl-9 Family Hypoxia Inducible Factor 1 | GC01M231363 |
| PRKCI | Protein Kinase C Iota | GC03P170222 |
| ASCC1 | Activating Signal Cointegrator 1 Complex Subunit 1 | GC10M072096 |
| FABP12 | Fatty Acid Binding Protein 12 | GC08M081524 |
| PTPN13 | Protein Tyrosine Phosphatase Non-Receptor Type 13 | GC04P086594 |
| TLR5 | Toll Like Receptor 5 | GC01M223109 |
| WNT10A | Wnt Family Member 10A | GC02P218880 |
| VTCN1 | V-Set Domain Containing T Cell Activation Inhibitor 1 | GC01M117143 |
| APOC3 | Apolipoprotein C3 | GC11P116829 |
| MTAP | Methylthioadenosine Phosphorylase | GC09P021792 |
| SLC11A2 | Solute Carrier Family 11 Member 2 | GC12M050979 |
| HCCAT5 | Hepatocellular Carcinoma Associated Transcript 5 | GC16P073092 |
| TCIM | Transcriptional And Immune Response Regulator | GC08P040153 |
| ASGR2 | Asialoglycoprotein Receptor 2 | GC17M007101 |
| NEDD9 | Neural Precursor Cell Expressed, Developmentally Down-Regulated 9 | GC06M011183 |
| CD69 | CD69 Molecule | GC12M013363 |
| GALT | Galactose-1-Phosphate Uridylyltransferase | GC09P034674 |
| ANXA10 | Annexin A10 | GC04P168081 |
| AMFR | Autocrine Motility Factor Receptor | GC16M056361 |
| FECH | Ferrochelatase | GC18M057544 |
| PON1 | Paraoxonase 1 | GC07M095297 |
| GNRH1 | Gonadotropin Releasing Hormone 1 | GC08M025419 |
| ACADL | Acyl-CoA Dehydrogenase Long Chain | GC02M210187 |
| THRB | Thyroid Hormone Receptor Beta | GC03M024117 |
| ALOX5 | Arachidonate 5-Lipoxygenase | GC10P045374 |
| TNFRSF10C | TNF Receptor Superfamily Member 10c | GC08P023102 |
| LAMTOR5 | Late Endosomal/Lysosomal Adaptor, MAPK And MTOR Activator 5 | GC01M110401 |
| MIR124-3 | MicroRNA 124-3 | GC20P063180 |
| ZBTB7A | Zinc Finger And BTB Domain Containing 7A | GC19M004045 |
| IFNL4 | Interferon Lambda 4 (Gene/Pseudogene) | GC19M039246 |
| GPX1 | Glutathione Peroxidase 1 | GC03M049370 |
| C11orf95 | Chromosome 11 Open Reading Frame 95 | GC11M063760 |
| THY1 | Thy-1 Cell Surface Antigen | GC11M119417 |
| IGF2BP1 | Insulin Like Growth Factor 2 MRNA Binding Protein 1 | GC17P048997 |
| MLXIPL | MLX Interacting Protein Like | GC07M073593 |
| ADAM9 | ADAM Metallopeptidase Domain 9 | GC08P038996 |
| GPER1 | G Protein-Coupled Estrogen Receptor 1 | GC07P001143 |
| KL | Klotho | GC13P033016 |
| HSPA8 | Heat Shock Protein Family A (Hsp70) Member 8 | GC11M123057 |
| RRAS2 | RAS Related 2 | GC11M014299 |
| H19-ICR | H19/IGF2 Imprinting Control Region | GC11U902738 |
| FLT3 | Fms Related Receptor Tyrosine Kinase 3 | GC13M028003 |
| STK4 | Serine/Threonine Kinase 4 | GC20P044966 |
| PDCD1LG2 | Programmed Cell Death 1 Ligand 2 | GC09P005510 |
| POLK | DNA Polymerase Kappa | GC05P075511 |
| MIR449A | MicroRNA 449a | GC05M055171 |
| EHMT2 | Euchromatic Histone Lysine Methyltransferase 2 | GC06M031879 |
| MIR199A2 | MicroRNA 199a-2 | GC01M172230 |
| PRDX2 | Peroxiredoxin 2 | GC19M012796 |
| ADIPOR1 | Adiponectin Receptor 1 | GC01M202940 |
| IFITM1 | Interferon Induced Transmembrane Protein 1 | GC11P000313 |
| OPCML | Opioid Binding Protein/Cell Adhesion Molecule Like | GC11M132414 |
| ALOX12 | Arachidonate 12-Lipoxygenase, 12S Type | GC17P006995 |
| BDNF | Brain Derived Neurotrophic Factor | GC11M027654 |
| RARG | Retinoic Acid Receptor Gamma | GC12M053210 |
| H4-16 | H4 Histone 16 | GC12M014768 |
| IL3 | Interleukin 3 | GC05P132060 |
| IGFBP5 | Insulin Like Growth Factor Binding Protein 5 | GC02M216672 |
| ECT2 | Epithelial Cell Transforming 2 | GC03P172750 |
| PRC1 | Protein Regulator Of Cytokinesis 1 | GC15M090966 |
| MSN | Moesin | GC0XP065588 |
| EFNA1 | Ephrin A1 | GC01P155127 |
| MFN2 | Mitofusin 2 | GC01P011980 |
| RBX1 | Ring-Box 1 | GC22P040951 |
| PAX5 | Paired Box 5 | GC09M036828 |
| TTR | Transthyretin | GC18P031557 |
| SIL1 | SIL1 Nucleotide Exchange Factor | GC05M138957 |
| MAP3K8 | Mitogen-Activated Protein Kinase Kinase Kinase 8 | GC10P030458 |
| KRT15 | Keratin 15 | GC17M041513 |
| NCL | Nucleolin | GC02M231453 |
| FZD4 | Frizzled Class Receptor 4 | GC11M086945 |
| STC1 | Stanniocalcin 1 | GC08M023841 |
| TLR7 | Toll Like Receptor 7 | GC0XP012867 |
| HHCM | Mahlavu Hepatocellular Carcinoma | GC08U900524 |
| RBL2 | RB Transcriptional Corepressor Like 2 | GC16P053433 |
| DSP | Desmoplakin | GC06P007541 |
| TGFBR3 | Transforming Growth Factor Beta Receptor 3 | GC01M091680 |
| VIP | Vasoactive Intestinal Peptide | GC06P152750 |
| NR1H3 | Nuclear Receptor Subfamily 1 Group H Member 3 | GC11P047248 |
| LINC01307 | Long Intergenic Non-Protein Coding RNA 1307 | GC01P101323 |
| ALCAM | Activated Leukocyte Cell Adhesion Molecule | GC03P105366 |
| CTCFL | CCCTC-Binding Factor Like | GC20M057495 |
| SEPSECS | Sep (O-Phosphoserine) TRNA:Sec (Selenocysteine) TRNA Synthase | GC04M025121 |
| ATF3 | Activating Transcription Factor 3 | GC01P212565 |
| MC2R | Melanocortin 2 Receptor | GC18M016790 |
| EREG | Epiregulin | GC04P074366 |
| CGB3 | Chorionic Gonadotropin Subunit Beta 3 | GC19M049024 |
| PLAT | Plasminogen Activator, Tissue Type | GC08M042174 |
| SULT1A1 | Sulfotransferase Family 1A Member 1 | GC16M028606 |
| BCAR1 | BCAR1 Scaffold Protein, Cas Family Member | GC16M075228 |
| TNK2 | Tyrosine Kinase Non Receptor 2 | GC03M195863 |
| ASGR1 | Asialoglycoprotein Receptor 1 | GC17M007173 |
| TH | Tyrosine Hydroxylase | GC11M002163 |
| CXCL5 | C-X-C Motif Chemokine Ligand 5 | GC04M073995 |
| PTPA | Protein Phosphatase 2 Phosphatase Activator | GC09P129111 |
| L1CAM | L1 Cell Adhesion Molecule | GC0XM153864 |
| ABCC8 | ATP Binding Cassette Subfamily C Member 8 | GC11M017392 |
| LINC01554 | Long Intergenic Non-Protein Coding RNA 1554 | GC05P095838 |
| CKAP4 | Cytoskeleton Associated Protein 4 | GC12M106237 |
| FURIN | Furin, Paired Basic Amino Acid Cleaving Enzyme | GC15P090868 |
| ACP1 | Acid Phosphatase 1 | GC02P000254 |
| EPHA1 | EPH Receptor A1 | GC07M143390 |
| BCS1L | BCS1 Homolog, Ubiquinol-Cytochrome C Reductase Complex Chaperone | GC02P218658 |
| KLF5 | Kruppel Like Factor 5 | GC13P073054 |
| NGF | Nerve Growth Factor | GC01M115285 |
| ITGA2 | Integrin Subunit Alpha 2 | GC05P052989 |
| MIR340 | MicroRNA 340 | GC05M180015 |
| MYO18B | Myosin XVIIIB | GC22P025742 |
| TAP2 | Transporter 2, ATP Binding Cassette Subfamily B Member | GC06M032821 |
| ADM | Adrenomedullin | GC11P010304 |
| YWHAE | Tyrosine 3-Monooxygenase/Tryptophan 5-Monooxygenase Activation Protein Epsilon | GC17M001346 |
| SEMA3B | Semaphorin 3B | GC03P050267 |
| YWHAZ | Tyrosine 3-Monooxygenase/Tryptophan 5-Monooxygenase Activation Protein Zeta | GC08M100917 |
| TGM2 | Transglutaminase 2 | GC20M038127 |
| TM6SF2 | Transmembrane 6 Superfamily Member 2 | GC19M019264 |
| EDNRB | Endothelin Receptor Type B | GC13M077895 |
| PRLR | Prolactin Receptor | GC05M035048 |
| CD55 | CD55 Molecule (Cromer Blood Group) | GC01P207321 |
| KLF14 | Kruppel Like Factor 14 | GC07M130731 |
| KAT2B | Lysine Acetyltransferase 2B | GC03P020081 |
| AKR1C1 | Aldo-Keto Reductase Family 1 Member C1 | GC10P004963 |
| PROX1 | Prospero Homeobox 1 | GC01P213983 |
| IRF3 | Interferon Regulatory Factor 3 | GC19M049659 |
| TRAF6 | TNF Receptor Associated Factor 6 | GC11M036467 |
| ST6GAL1 | ST6 Beta-Galactoside Alpha-2,6-Sialyltransferase 1 | GC03P186930 |
| APPL1 | Adaptor Protein, Phosphotyrosine Interacting With PH Domain And Leucine Zipper 1 | GC03P057227 |
| CNOT9 | CCR4-NOT Transcription Complex Subunit 9 | GC02P218569 |
| LAMA3 | Laminin Subunit Alpha 3 | GC18P023689 |
| ALAS2 | 5'-Aminolevulinate Synthase 2 | GC0XM055009 |
| HPN | Hepsin | GC19P035040 |
| FATE1 | Fetal And Adult Testis Expressed 1 | GC0XP151716 |
| SAA1 | Serum Amyloid A1 | GC11P018267 |
| CASP2 | Caspase 2 | GC07P144746 |
| TYK2 | Tyrosine Kinase 2 | GC19M010350 |
| EEF1A1 | Eukaryotic Translation Elongation Factor 1 Alpha 1 | GC06M073515 |
| KLK10 | Kallikrein Related Peptidase 10 | GC19M051012 |
| FOLR1 | Folate Receptor Alpha | GC11P072190 |
| FUS | FUS RNA Binding Protein | GC16P031418 |
| ANXA6 | Annexin A6 | GC05M151077 |
| LPL | Lipoprotein Lipase | GC08P019901 |
| PTGES | Prostaglandin E Synthase | GC09M129738 |
| CASR | Calcium Sensing Receptor | GC03P122183 |
| ANG | Angiogenin | GC14P020810 |
| APOBEC3B | Apolipoprotein B MRNA Editing Enzyme Catalytic Subunit 3B | GC22P038982 |
| DCUN1D1 | Defective In Cullin Neddylation 1 Domain Containing 1 | GC03M182938 |
| CASC9 | Cancer Susceptibility 9 | GC08M075132 |
| RB1CC1 | RB1 Inducible Coiled-Coil 1 | GC08M052622 |
| MIR574 | MicroRNA 574 | GC04P038872 |
| CXCL14 | C-X-C Motif Chemokine Ligand 14 | GC05M135617 |
| MCAM | Melanoma Cell Adhesion Molecule | GC11M119308 |
| CRYAB | Crystallin Alpha B | GC11M111908 |
| WEE1 | WEE1 G2 Checkpoint Kinase | GC11P009573 |
| ACE | Angiotensin I Converting Enzyme | GC17P063477 |
| ECM1 | Extracellular Matrix Protein 1 | GC01P150508 |
| MAD2L2 | Mitotic Arrest Deficient 2 Like 2 | GC01M011674 |
| CUL1 | Cullin 1 | GC07P148697 |
| PRDX3 | Peroxiredoxin 3 | GC10M119167 |
| MIR28 | MicroRNA 28 | GC03P188688 |
| PDIA3 | Protein Disulfide Isomerase Family A Member 3 | GC15P043746 |
| ABCG5 | ATP Binding Cassette Subfamily G Member 5 | GC02M043806 |
| ALPG | Alkaline Phosphatase, Germ Cell | GC02P232407 |
| PTH | Parathyroid Hormone | GC11M013492 |
| MDM4 | MDM4 Regulator Of P53 | GC01P204516 |
| HSPA1B | Heat Shock Protein Family A (Hsp70) Member 1B | GC06P033427 |
| PDE11A | Phosphodiesterase 11A | GC02M177624 |
| WNT10B | Wnt Family Member 10B | GC12M048965 |
| HSF1 | Heat Shock Transcription Factor 1 | GC08P144291 |
| VWF | Von Willebrand Factor | GC12M005917 |
| RBPJ | Recombination Signal Binding Protein For Immunoglobulin Kappa J Region | GC04P026165 |
| AHSG | Alpha 2-HS Glycoprotein | GC03P186612 |
| CD28 | CD28 Molecule | GC02P203706 |
| MCC | MCC Regulator Of WNT Signaling Pathway | GC05M113022 |
| HNRNPA2B1 | Heterogeneous Nuclear Ribonucleoprotein A2/B1 | GC07M026174 |
| ITGB3 | Integrin Subunit Beta 3 | GC17P047254 |
| KDM6A | Lysine Demethylase 6A | GC0XP044873 |
| FOXC1 | Forkhead Box C1 | GC06P001610 |
| SNCG | Synuclein Gamma | GC10P086957 |
| HERC2 | HECT And RLD Domain Containing E3 Ubiquitin Protein Ligase 2 | GC15M028111 |
| SIRT3 | Sirtuin 3 | GC11M000215 |
| SULF2 | Sulfatase 2 | GC20M047656 |
| ATAD2 | ATPase Family AAA Domain Containing 2 | GC08M123319 |
| TPD52 | Tumor Protein D52 | GC08M080034 |
| CTHRC1 | Collagen Triple Helix Repeat Containing 1 | GC08P103371 |
| CDK7 | Cyclin Dependent Kinase 7 | GC05P069242 |
| FDPS | Farnesyl Diphosphate Synthase | GC01P155308 |
| MIR32 | MicroRNA 32 | GC09M109046 |
| NCOR2 | Nuclear Receptor Corepressor 2 | GC12M124324 |
| GRAMD1A | GRAM Domain Containing 1A | GC19P037703 |
| PTPRN | Protein Tyrosine Phosphatase Receptor Type N | GC02M219289 |
| GHR | Growth Hormone Receptor | GC05P042429 |
| MIR218-2 | MicroRNA 218-2 | GC05M168768 |
| MAP1LC3A | Microtubule Associated Protein 1 Light Chain 3 Alpha | GC20P034546 |
| SRD5A1 | Steroid 5 Alpha-Reductase 1 | GC05P006633 |
| NET1 | Neuroepithelial Cell Transforming 1 | GC10P005444 |
| HDAC6 | Histone Deacetylase 6 | GC0XP048801 |
| LTF | Lactotransferrin | GC03M046435 |
| MIR103A1 | MicroRNA 103a-1 | GC05M168560 |
| MIR129-1 | MicroRNA 129-1 | GC07P128207 |
| ESRRA | Estrogen Related Receptor Alpha | GC11P064305 |
| LIF | LIF Interleukin 6 Family Cytokine | GC22M030240 |
| TMEM30A | Transmembrane Protein 30A | GC06M075252 |
| MIR455 | MicroRNA 455 | GC09P114209 |
| PLA2G2A | Phospholipase A2 Group IIA | GC01M019975 |
| CCL20 | C-C Motif Chemokine Ligand 20 | GC02P227813 |
| LBR | Lamin B Receptor | GC01M225401 |
| CRABP1 | Cellular Retinoic Acid Binding Protein 1 | GC15P078340 |
| CCK | Cholecystokinin | GC03M042274 |
| SNHG16 | Small Nucleolar RNA Host Gene 16 | GC17P076559 |
| ARTN | Artemin | GC01P043933 |
| LIFR | LIF Receptor Subunit Alpha | GC05M038475 |
| F10 | Coagulation Factor X | GC13P113122 |
| CCNL1 | Cyclin L1 | GC03M157146 |
| SIRT6 | Sirtuin 6 | GC19M004174 |
| CD5 | CD5 Molecule | GC11P061115 |
| KDM5C | Lysine Demethylase 5C | GC0XM053159 |
| GAL3ST1 | Galactose-3-O-Sulfotransferase 1 | GC22M030554 |
| UCP2 | Uncoupling Protein 2 | GC11M073974 |
| VCL | Vinculin | GC10P073995 |
| CHI3L1 | Chitinase 3 Like 1 | GC01M203148 |
| SLC16A4 | Solute Carrier Family 16 Member 4 | GC01M110362 |
| SLC29A1 | Solute Carrier Family 29 Member 1 (Augustine Blood Group) | GC06P044219 |
| SASH1 | SAM And SH3 Domain Containing 1 | GC06P148212 |
| CD58 | CD58 Molecule | GC01M116514 |
| MIR135B | MicroRNA 135b | GC01M205448 |
| NQO2 | N-Ribosyldihydronicotinamide:Quinone Reductase 2 | GC06P003004 |
| FGF8 | Fibroblast Growth Factor 8 | GC10M101770 |
| CLDN6 | Claudin 6 | GC16M003014 |
| CCN3 | Cellular Communication Network Factor 3 | GC08P119416 |
| KDM5B | Lysine Demethylase 5B | GC01M202696 |
| TJP1 | Tight Junction Protein 1 | GC15M029699 |
| LDHB | Lactate Dehydrogenase B | GC12M021635 |
| DAXX | Death Domain Associated Protein | GC06M033318 |
| SLC9A3R1 | SLC9A3 Regulator 1 | GC17P074749 |
| PRKD1 | Protein Kinase D1 | GC14M029576 |
| CDK14 | Cyclin Dependent Kinase 14 | GC07P090470 |
| KPNA2 | Karyopherin Subunit Alpha 2 | GC17P068035 |
| RRM1 | Ribonucleotide Reductase Catalytic Subunit M1 | GC11P004115 |
| PRKCE | Protein Kinase C Epsilon | GC02P045651 |
| HIF1AN | Hypoxia Inducible Factor 1 Subunit Alpha Inhibitor | GC10P100529 |
| DSG3 | Desmoglein 3 | GC18P031447 |
| DLL3 | Delta Like Canonical Notch Ligand 3 | GC19P039498 |
| LATS2 | Large Tumor Suppressor Kinase 2 | GC13M020973 |
| PPARD | Peroxisome Proliferator Activated Receptor Delta | GC06P046031 |
| GLS | Glutaminase | GC02P190880 |
| SERPINB1 | Serpin Family B Member 1 | GC06M002833 |
| MTSS1 | MTSS I-BAR Domain Containing 1 | GC08M124550 |
| CXADR | CXADR Ig-Like Cell Adhesion Molecule | GC21P017512 |
| FYN | FYN Proto-Oncogene, Src Family Tyrosine Kinase | GC06M111660 |
| CLDN8 | Claudin 8 | GC21M030214 |
| ATF1 | Activating Transcription Factor 1 | GC12P050763 |
| USP7 | Ubiquitin Specific Peptidase 7 | GC16M008892 |
| SLC39A6 | Solute Carrier Family 39 Member 6 | GC18M036108 |
| NR1I3 | Nuclear Receptor Subfamily 1 Group I Member 3 | GC01M161229 |
| DEK | DEK Proto-Oncogene | GC06M018224 |
| FTH1 | Ferritin Heavy Chain 1 | GC11M061959 |
| LGR5 | Leucine Rich Repeat Containing G Protein-Coupled Receptor 5 | GC12P071439 |
| ADIPOR2 | Adiponectin Receptor 2 | GC12P001670 |
| VCAN | Versican | GC05P083471 |
| CCR4 | C-C Motif Chemokine Receptor 4 | GC03P032951 |
| AQP3 | Aquaporin 3 (Gill Blood Group) | GC09M033431 |
| CCAR2 | Cell Cycle And Apoptosis Regulator 2 | GC08P022604 |
| TXNRD2 | Thioredoxin Reductase 2 | GC22M019863 |
| GFRA1 | GDNF Family Receptor Alpha 1 | GC10M116056 |
| CTAG2 | Cancer/Testis Antigen 2 | GC0XM154651 |
| CD68 | CD68 Molecule | GC17P007579 |
| MIR184 | MicroRNA 184 | GC15P079209 |
| MRPL58 | Mitochondrial Ribosomal Protein L58 | GC17P075013 |
| IL21 | Interleukin 21 | GC04M122612 |
| MIR16-2 | MicroRNA 16-2 | GC03P160415 |
| DCK | Deoxycytidine Kinase | GC04P070992 |
| APP | Amyloid Beta Precursor Protein | GC21M025880 |
| FOXA2 | Forkhead Box A2 | GC20M022581 |
| RETN | Resistin | GC19P007669 |
| ALAS1 | 5'-Aminolevulinate Synthase 1 | GC03P052198 |
| CDH5 | Cadherin 5 | GC16P066366 |
| ZMYND10 | Zinc Finger MYND-Type Containing 10 | GC03M050372 |
| HLA-E | Major Histocompatibility Complex, Class I, E | GC06P033358 |
| CCNH | Cyclin H | GC05M087377 |
| INTS6 | Integrator Complex Subunit 6 | GC13M051354 |
| CD46 | CD46 Molecule | GC01P207752 |
| ANXA4 | Annexin A4 | GC02P069644 |
| IL32 | Interleukin 32 | GC16P004058 |
| TPI1 | Triosephosphate Isomerase 1 | GC12P006867 |
| SFRP2 | Secreted Frizzled Related Protein 2 | GC04M153780 |
| FUT4 | Fucosyltransferase 4 | GC11P094544 |
| CYP3A5 | Cytochrome P450 Family 3 Subfamily A Member 5 | GC07M099648 |
| ACTN4 | Actinin Alpha 4 | GC19P038647 |
| JUNB | JunB Proto-Oncogene, AP-1 Transcription Factor Subunit | GC19P012791 |
| SPINT1 | Serine Peptidase Inhibitor, Kunitz Type 1 | GC15P040844 |
| FLG | Filaggrin | GC01M152274 |
| IRF7 | Interferon Regulatory Factor 7 | GC11M000612 |
| MIR376A1 | MicroRNA 376a-1 | GC14P104448 |
| HIC1 | HIC ZBTB Transcriptional Repressor 1 | GC17P002054 |
| MIR502 | MicroRNA 502 | GC0XP050014 |
| GHRL | Ghrelin And Obestatin Prepropeptide | GC03M010285 |
| DLL1 | Delta Like Canonical Notch Ligand 1 | GC06M170282 |
| PTPN1 | Protein Tyrosine Phosphatase Non-Receptor Type 1 | GC20P050510 |
| CRABP2 | Cellular Retinoic Acid Binding Protein 2 | GC01M156701 |
| RBP4 | Retinol Binding Protein 4 | GC10M093591 |
| TIMELESS | Timeless Circadian Regulator | GC12M056416 |
| PNOC | Prepronociceptin | GC08P028316 |
| EPHA7 | EPH Receptor A7 | GC06M093240 |
| MIR542 | MicroRNA 542 | GC0XM134620 |
| PRTN3 | Proteinase 3 | GC19P000840 |
| RABL3 | RAB, Member Of RAS Oncogene Family Like 3 | GC03M120686 |
| CTSG | Cathepsin G | GC14M024573 |
| PTPN6 | Protein Tyrosine Phosphatase Non-Receptor Type 6 | GC12P007875 |
| PGK1 | Phosphoglycerate Kinase 1 | GC0XP077925 |
| PRMT5 | Protein Arginine Methyltransferase 5 | GC14M022920 |
| LAMC1 | Laminin Subunit Gamma 1 | GC01P182992 |
| GNRHR | Gonadotropin Releasing Hormone Receptor | GC04M067737 |
| VCP | Valosin Containing Protein | GC09M035056 |
| AKR1C3 | Aldo-Keto Reductase Family 1 Member C3 | GC10P005035 |
| GFAP | Glial Fibrillary Acidic Protein | GC17M044905 |
| CYP11B1 | Cytochrome P450 Family 11 Subfamily B Member 1 | GC08M142872 |
| MDC1 | Mediator Of DNA Damage Checkpoint 1 | GC06M030795 |
| CDCP1 | CUB Domain Containing Protein 1 | GC03M045082 |
| TP53BP1 | Tumor Protein P53 Binding Protein 1 | GC15M043403 |
| CD79A | CD79a Molecule | GC19P041877 |
| MEST | Mesoderm Specific Transcript | GC07P130486 |
| PIM1 | Pim-1 Proto-Oncogene, Serine/Threonine Kinase | GC06P046065 |
| CYP24A1 | Cytochrome P450 Family 24 Subfamily A Member 1 | GC20M054153 |
| SLC2A4 | Solute Carrier Family 2 Member 4 | GC17P007282 |
| CCNE2 | Cyclin E2 | GC08M094879 |
| JAG2 | Jagged Canonical Notch Ligand 2 | GC14M105140 |
| SOX10 | SRY-Box Transcription Factor 10 | GC22M045379 |
| CDX1 | Caudal Type Homeobox 1 | GC05P150133 |
| MIR572 | MicroRNA 572 | GC04P013164 |
| IGFBP7 | Insulin Like Growth Factor Binding Protein 7 | GC04M057030 |
| ZNF148 | Zinc Finger Protein 148 | GC03M125225 |
| AIMP2 | Aminoacyl TRNA Synthetase Complex Interacting Multifunctional Protein 2 | GC07P006016 |
| ROR2 | Receptor Tyrosine Kinase Like Orphan Receptor 2 | GC09M091564 |
| HOXB9 | Homeobox B9 | GC17M048621 |
| HPGD | 15-Hydroxyprostaglandin Dehydrogenase | GC04M174490 |
| NDRG2 | NDRG Family Member 2 | GC14M021016 |
| NR4A3 | Nuclear Receptor Subfamily 4 Group A Member 3 | GC09P099821 |
| MIR675 | MicroRNA 675 | GC11M001997 |
| ELF3 | E74 Like ETS Transcription Factor 3 | GC01P202007 |
| MATK | Megakaryocyte-Associated Tyrosine Kinase | GC19M003777 |
| SELP | Selectin P | GC01M169558 |
| SCYL1 | SCY1 Like Pseudokinase 1 | GC11P065525 |
| SIAH1 | Siah E3 Ubiquitin Protein Ligase 1 | GC16M048357 |
| MAPK7 | Mitogen-Activated Protein Kinase 7 | GC17P019379 |
| HHIP | Hedgehog Interacting Protein | GC04P144645 |
| BBC3 | BCL2 Binding Component 3 | GC19M047220 |
| ARID4B | AT-Rich Interaction Domain 4B | GC01M235133 |
| TMPRSS4 | Transmembrane Serine Protease 4 | GC11P118077 |
| ITGAL | Integrin Subunit Alpha L | GC16P030472 |
| MIR29B2 | MicroRNA 29b-2 | GC01M207807 |
| LAMA5 | Laminin Subunit Alpha 5 | GC20M062307 |
| UBE2C | Ubiquitin Conjugating Enzyme E2 C | GC20P045812 |
| GAS8-AS1 | GAS8 Antisense RNA 1 | GC16M090028 |
| LOXL4 | Lysyl Oxidase Like 4 | GC10M098247 |
| EIF2AK3 | Eukaryotic Translation Initiation Factor 2 Alpha Kinase 3 | GC02M088637 |
| GSDMB | Gasdermin B | GC17M039904 |
| SLC3A2 | Solute Carrier Family 3 Member 2 | GC11P062856 |
| BTC | Betacellulin | GC04M074744 |
| MIR196B | MicroRNA 196b | GC07M027224 |
| MMP26 | Matrix Metallopeptidase 26 | GC11P004706 |
| PSEN2 | Presenilin 2 | GC01P226870 |
| PIWIL1 | Piwi Like RNA-Mediated Gene Silencing 1 | GC12P130337 |
| SMAD6 | SMAD Family Member 6 | GC15P066702 |
| FANCD2 | FA Complementation Group D2 | GC03P010026 |
| ACTG2 | Actin Gamma 2, Smooth Muscle | GC02P073892 |
| PRKAA2 | Protein Kinase AMP-Activated Catalytic Subunit Alpha 2 | GC01P056645 |
| PTPRJ | Protein Tyrosine Phosphatase Receptor Type J | GC11P048002 |
| TK1 | Thymidine Kinase 1 | GC17M078175 |
| CD109 | CD109 Molecule | GC06P073695 |
| AGO2 | Argonaute RISC Catalytic Component 2 | GC08M140522 |
| PPP1R13L | Protein Phosphatase 1 Regulatory Subunit 13 Like | GC19M045379 |
| GLI3 | GLI Family Zinc Finger 3 | GC07M041960 |
| ARNT | Aryl Hydrocarbon Receptor Nuclear Translocator | GC01M150809 |
| MAT2A | Methionine Adenosyltransferase 2A | GC02P085538 |
| ACVR1B | Activin A Receptor Type 1B | GC12P051951 |
| SRF | Serum Response Factor | GC06P043171 |
| HDAC4 | Histone Deacetylase 4 | GC02M239048 |
| RAN | RAN, Member RAS Oncogene Family | GC12P130871 |
| UBE3A | Ubiquitin Protein Ligase E3A | GC15M025333 |
| YES1 | YES Proto-Oncogene 1, Src Family Tyrosine Kinase | GC18M000721 |
| DROSHA | Drosha Ribonuclease III | GC05M031401 |
| NAFLD1 | Fatty Liver Disease 1, Susceptiblity To | GC22U901005 |
| NAFLD2 | Fatty Liver Disease, Nonalcoholic, Susceptibility To, 2 | GC11U901533 |
| FTL | Ferritin Light Chain | GC19P048965 |
| MYH11 | Myosin Heavy Chain 11 | GC16M015704 |
| PRAME | Preferentially Expressed Antigen In Melanoma | GC22M022547 |
| HOXA1 | Homeobox A1 | GC07M027092 |
| ADA | Adenosine Deaminase | GC20M044620 |
| LGALS9 | Galectin 9 | GC17P027629 |
| TINCR | TINCR Ubiquitin Domain Containing | GC19M005558 |
| FUT2 | Fucosyltransferase 2 | GC19P048695 |
| CASP1 | Caspase 1 | GC11M105025 |
| PKP3 | Plakophilin 3 | GC11P000394 |
| DUSP6 | Dual Specificity Phosphatase 6 | GC12M089347 |
| PLAGL1 | PLAG1 Like Zinc Finger 1 | GC06M143940 |
| ST13 | ST13 Hsp70 Interacting Protein | GC22M044564 |
| NEK2 | NIMA Related Kinase 2 | GC01M211658 |
| GSR | Glutathione-Disulfide Reductase | GC08M030678 |
| ONECUT1 | One Cut Homeobox 1 | GC15M059715 |
| MAP1LC3B | Microtubule Associated Protein 1 Light Chain 3 Beta | GC16P087384 |
| MIR217 | MicroRNA 217 | GC02M055982 |
| RALA | RAS Like Proto-Oncogene A | GC07P039622 |
| ITGA9 | Integrin Subunit Alpha 9 | GC03P037468 |
| TGM3 | Transglutaminase 3 | GC20P002276 |
| PFKFB3 | 6-Phosphofructo-2-Kinase/Fructose-2,6-Biphosphatase 3 | GC10P006144 |
| HSPB2 | Heat Shock Protein Family B (Small) Member 2 | GC11P111913 |
| AGTR1 | Angiotensin II Receptor Type 1 | GC03P148697 |
| CCS | Copper Chaperone For Superoxide Dismutase | GC11P066593 |
| HDAC3 | Histone Deacetylase 3 | GC05M141583 |
| PDE8B | Phosphodiesterase 8B | GC05P077180 |
| RPS27A | Ribosomal Protein S27a | GC02P055231 |
| MIR590 | MicroRNA 590 | GC07P074191 |
| EPHX2 | Epoxide Hydrolase 2 | GC08P027490 |
| NOX1 | NADPH Oxidase 1 | GC0XM100843 |
| DCLK1 | Doublecortin Like Kinase 1 | GC13M035768 |
| RHOB | Ras Homolog Family Member B | GC02P020447 |
| MIR425 | MicroRNA 425 | GC03M049203 |
| MIR302A | MicroRNA 302a | GC04M112674 |
| TOR1A | Torsin Family 1 Member A | GC09M129812 |
| SATB2 | SATB Homeobox 2 | GC02M199269 |
| FERMT1 | Fermitin Family Member 1 | GC20M006074 |
| LRP1 | LDL Receptor Related Protein 1 | GC12P057128 |
| MIR92A1 | MicroRNA 92a-1 | GC13P091420 |
| TLN1 | Talin 1 | GC09M035687 |
| GMNN | Geminin DNA Replication Inhibitor | GC06P024779 |
| TMEM8B | Transmembrane Protein 8B | GC09P035785 |
| TINF2 | TERF1 Interacting Nuclear Factor 2 | GC14M024234 |
| PKP1 | Plakophilin 1 | GC01P201252 |
| REG3A | Regenerating Family Member 3 Alpha | GC02M079157 |
| MIR129-2 | MicroRNA 129-2 | GC11P043633 |
| FOXD2-AS1 | FOXD2 Adjacent Opposite Strand RNA 1 | GC01M047432 |
| SLC31A1 | Solute Carrier Family 31 Member 1 | GC09P113221 |
| PTGER2 | Prostaglandin E Receptor 2 | GC14P052314 |
| TEP1 | Telomerase Associated Protein 1 | GC14M020365 |
| SIX1 | SIX Homeobox 1 | GC14M060643 |
| SMARCE1 | SWI/SNF Related, Matrix Associated, Actin Dependent Regulator Of Chromatin, Subfamily E, Member 1 | GC17M040624 |
| SPAG9 | Sperm Associated Antigen 9 | GC17M050962 |
| MYD88 | MYD88 Innate Immune Signal Transduction Adaptor | GC03P038179 |
| NEDD4 | NEDD4 E3 Ubiquitin Protein Ligase | GC15M055826 |
| CPS1 | Carbamoyl-Phosphate Synthase 1 | GC02P210477 |
| IL33 | Interleukin 33 | GC09P006206 |
| TUBB3 | Tubulin Beta 3 Class III | GC16P089919 |
| NR5A2 | Nuclear Receptor Subfamily 5 Group A Member 2 | GC01P199996 |
| BMX | BMX Non-Receptor Tyrosine Kinase | GC0XP015392 |
| TAGLN | Transgelin | GC11P117199 |
| GLOD4 | Glyoxalase Domain Containing 4 | GC17M000757 |
| CD63 | CD63 Molecule | GC12M055725 |
| ATF2 | Activating Transcription Factor 2 | GC02M175072 |
| MAN1B1 | Mannosidase Alpha Class 1B Member 1 | GC09P137086 |
| UBE2D1 | Ubiquitin Conjugating Enzyme E2 D1 | GC10P058334 |
| FGF5 | Fibroblast Growth Factor 5 | GC04P080266 |
| PNLIP | Pancreatic Lipase | GC10P116545 |
| DAB2IP | DAB2 Interacting Protein | GC09P121566 |
| COL1A1 | Collagen Type I Alpha 1 Chain | GC17M050183 |
| HCP5 | HLA Complex P5 | GC06P031400 |
| DLAT | Dihydrolipoamide S-Acetyltransferase | GC11P112024 |
| PYY | Peptide YY | GC17M043952 |
| ESM1 | Endothelial Cell Specific Molecule 1 | GC05M054977 |
| TSPY1 | Testis Specific Protein Y-Linked 1 | GC0YP009469 |
| CCL4 | C-C Motif Chemokine Ligand 4 | GC17P036103 |
| RRM2B | Ribonucleotide Reductase Regulatory TP53 Inducible Subunit M2B | GC08M102204 |
| PPOX | Protoporphyrinogen Oxidase | GC01P161167 |
| DCN | Decorin | GC12M091140 |
| UBC | Ubiquitin C | GC12M124911 |
| EFNB2 | Ephrin B2 | GC13M106489 |
| NUMB | NUMB Endocytic Adaptor Protein | GC14M073275 |
| APOD | Apolipoprotein D | GC03M195568 |
| LRPPRC | Leucine Rich Pentatricopeptide Repeat Containing | GC02M043850 |
| ARSH | Arylsulfatase Family Member H | GC0XP003006 |
| MIR608 | MicroRNA 608 | GC10P100974 |
| RAB25 | RAB25, Member RAS Oncogene Family | GC01P156061 |
| MBD4 | Methyl-CpG Binding Domain 4, DNA Glycosylase | GC03M129430 |
| MIR1-1 | MicroRNA 1-1 | GC20P062873 |
| ANXA3 | Annexin A3 | GC04P078551 |
| STIM1 | Stromal Interaction Molecule 1 | GC11P003855 |
| HAVCR1 | Hepatitis A Virus Cellular Receptor 1 | GC05M157007 |
| HOXA10 | Homeobox A10 | GC07M027229 |
| RIPK3 | Receptor Interacting Serine/Threonine Kinase 3 | GC14M024336 |
| GLB1 | Galactosidase Beta 1 | GC03M033013 |
| PLA2G4A | Phospholipase A2 Group IVA | GC01P186798 |
| PRKG1 | Protein Kinase CGMP-Dependent 1 | GC10P050991 |
| AVP | Arginine Vasopressin | GC20M003082 |
| CSTA | Cystatin A | GC03P122325 |
| KRT16 | Keratin 16 | GC17M041609 |
| PEA15 | Proliferation And Apoptosis Adaptor Protein 15 | GC01P160205 |
| TRIM28 | Tripartite Motif Containing 28 | GC19P058544 |
| CDH6 | Cadherin 6 | GC05P031193 |
| HTRA2 | HtrA Serine Peptidase 2 | GC02P074529 |
| NPRL2 | NPR2 Like, GATOR1 Complex Subunit | GC03M050352 |
| RING1 | Ring Finger Protein 1 | GC06P033208 |
| MIR17HG | MiR-17-92a-1 Cluster Host Gene | GC13P091347 |
| ANGPTL4 | Angiopoietin Like 4 | GC19P008363 |
| CYP21A2 | Cytochrome P450 Family 21 Subfamily A Member 2 | GC06P033435 |
| RYBP | RING1 And YY1 Binding Protein | GC03M072371 |
| MRPS27 | Mitochondrial Ribosomal Protein S27 | GC05M072219 |
| SULT2A1 | Sulfotransferase Family 2A Member 1 | GC19M047870 |
| DHDH | Dihydrodiol Dehydrogenase | GC19P048933 |
| CSE1L | Chromosome Segregation 1 Like | GC20P049046 |
| RHOD | Ras Homolog Family Member D | GC11P067057 |
| PRF1 | Perforin 1 | GC10M070597 |
| SEMA3A | Semaphorin 3A | GC07M083955 |
| FBLN1 | Fibulin 1 | GC22P045502 |
| ATG7 | Autophagy Related 7 | GC03P011273 |
| PON3 | Paraoxonase 3 | GC07M095359 |
| GATA4 | GATA Binding Protein 4 | GC08P011676 |
| CLDND1 | Claudin Domain Containing 1 | GC03M098497 |
| MIR885 | MicroRNA 885 | GC03M010413 |
| NKX3-1 | NK3 Homeobox 1 | GC08M023678 |
| MIR202 | MicroRNA 202 | GC10M133247 |
| BLZF1 | Basic Leucine Zipper Nuclear Factor 1 | GC01P169367 |
| CCR2 | C-C Motif Chemokine Receptor 2 | GC03P046356 |
| FGFBP1 | Fibroblast Growth Factor Binding Protein 1 | GC04M015937 |
| RAD52 | RAD52 Homolog, DNA Repair Protein | GC12M000912 |
| GNAS-AS1 | GNAS Antisense RNA 1 | GC20M058843 |
| GAL | Galanin And GMAP Prepropeptide | GC11P068701 |
| GPRC5A | G Protein-Coupled Receptor Class C Group 5 Member A | GC12P012890 |
| FOXP2 | Forkhead Box P2 | GC07P114086 |
| HDAC5 | Histone Deacetylase 5 | GC17M044076 |
| LIG4 | DNA Ligase 4 | GC13M108207 |
| PENK | Proenkephalin | GC08M056436 |
| BTD | Biotinidase | GC03P015621 |
| HIPK2 | Homeodomain Interacting Protein Kinase 2 | GC07M139561 |
| JUND | JunD Proto-Oncogene, AP-1 Transcription Factor Subunit | GC19M018279 |
| THRA | Thyroid Hormone Receptor Alpha | GC17P040058 |
| TRAF4 | TNF Receptor Associated Factor 4 | GC17P028763 |
| TNFRSF1B | TNF Receptor Superfamily Member 1B | GC01P012167 |
| S100P | S100 Calcium Binding Protein P | GC04P006700 |
| CRH | Corticotropin Releasing Hormone | GC08M066176 |
| CSMD1 | CUB And Sushi Multiple Domains 1 | GC08M002953 |
| NKX2-8 | NK2 Homeobox 8 | GC14M036580 |
| ATP8A1 | ATPase Phospholipid Transporting 8A1 | GC04M042410 |
| MYH8 | Myosin Heavy Chain 8 | GC17M010390 |
| RPS6 | Ribosomal Protein S6 | GC09M019366 |
| USF1 | Upstream Transcription Factor 1 | GC01M161039 |
| MTTP | Microsomal Triglyceride Transfer Protein | GC04P099563 |
| GSTZ1 | Glutathione S-Transferase Zeta 1 | GC14P077320 |
| FAF1 | Fas Associated Factor 1 | GC01M050439 |
| RUNX1 | RUNX Family Transcription Factor 1 | GC21M034787 |
| EIF5A2 | Eukaryotic Translation Initiation Factor 5A2 | GC03M170888 |
| CD99 | CD99 Molecule (Xg Blood Group) | GC0XP002691 |
| HEY1 | Hes Related Family BHLH Transcription Factor With YRPW Motif 1 | GC08M079764 |
| CASP6 | Caspase 6 | GC04M109688 |
| RASSF5 | Ras Association Domain Family Member 5 | GC01P206507 |
| DNASE1 | Deoxyribonuclease 1 | GC16P003611 |
| DAB2 | DAB Adaptor Protein 2 | GC05M039371 |
| MAP3K14 | Mitogen-Activated Protein Kinase Kinase Kinase 14 | GC17M045263 |
| ZFX | Zinc Finger Protein X-Linked | GC0XP024148 |
| PRKAB1 | Protein Kinase AMP-Activated Non-Catalytic Subunit Beta 1 | GC12P119632 |
| CGA | Glycoprotein Hormones, Alpha Polypeptide | GC06M087085 |
| ID2 | Inhibitor Of DNA Binding 2 | GC02P008772 |
| FTCD | Formimidoyltransferase Cyclodeaminase | GC21M047161 |
| CYP11A1 | Cytochrome P450 Family 11 Subfamily A Member 1 | GC15M074337 |
| EEF1A2 | Eukaryotic Translation Elongation Factor 1 Alpha 2 | GC20M063488 |
| DSE | Dermatan Sulfate Epimerase | GC06P116255 |
| CKB | Creatine Kinase B | GC14M103519 |
| KIFC1 | Kinesin Family Member C1 | GC06P033391 |
| ROR1 | Receptor Tyrosine Kinase Like Orphan Receptor 1 | GC01P063774 |
| PTMA | Prothymosin Alpha | GC02P231707 |
| PTK7 | Protein Tyrosine Kinase 7 (Inactive) | GC06P043076 |
| CYP2C19 | Cytochrome P450 Family 2 Subfamily C Member 19 | GC10P094762 |
| MIR432 | MicroRNA 432 | GC14P104254 |
| HSD17B1 | Hydroxysteroid 17-Beta Dehydrogenase 1 | GC17P042548 |
| ADAMTSL1 | ADAMTS Like 1 | GC09P017906 |
| TNFRSF11A | TNF Receptor Superfamily Member 11a | GC18P062325 |
| SOAT1 | Sterol O-Acyltransferase 1 | GC01P179262 |
| RPA1 | Replication Protein A1 | GC17P001829 |
| KLK6 | Kallikrein Related Peptidase 6 | GC19M050958 |
| ENAH | ENAH Actin Regulator | GC01M225486 |
| MIR4435-2HG | MIR4435-2 Host Gene | GC02M111037 |
| AGK | Acylglycerol Kinase | GC07P141551 |
| OSM | Oncostatin M | GC22M030262 |
| SERPINF1 | Serpin Family F Member 1 | GC17P001761 |
| KHK | Ketohexokinase | GC02P027086 |
| RTN4 | Reticulon 4 | GC02M054934 |
| CSTB | Cystatin B | GC21M043772 |
| MIR31HG | MIR31 Host Gene | GC09M021411 |
| IRAK1 | Interleukin 1 Receptor Associated Kinase 1 | GC0XM154010 |
| PC | Pyruvate Carboxylase | GC11M066848 |
| RAD54B | RAD54 Homolog B | GC08M094371 |
| MTR | 5-Methyltetrahydrofolate-Homocysteine Methyltransferase | GC01P236795 |
| ULK1 | Unc-51 Like Autophagy Activating Kinase 1 | GC12P131894 |
| GPR87 | G Protein-Coupled Receptor 87 | GC03M151294 |
| MIR663A | MicroRNA 663a | GC20M026189 |
| PTP4A1 | Protein Tyrosine Phosphatase 4A1 | GC06P063521 |
| PTGER1 | Prostaglandin E Receptor 1 | GC19M014444 |
| HLA-DRA | Major Histocompatibility Complex, Class II, DR Alpha | GC06P032439 |
| TRIM33 | Tripartite Motif Containing 33 | GC01M114392 |
| CCL18 | C-C Motif Chemokine Ligand 18 | GC17P036064 |
| ENSG00000266919 |  | GC17M030118 |
| SLC19A1 | Solute Carrier Family 19 Member 1 | GC21M045493 |
| RHOH | Ras Homolog Family Member H | GC04P040192 |
| EPB41L4A-DT | EPB41L4A Divergent Transcript | GC05P112421 |
| BCL3 | BCL3 Transcription Coactivator | GC19P044747 |
| CHKB | Choline Kinase Beta | GC22M050578 |
| DEPDC1 | DEP Domain Containing 1 | GC01M068474 |
| CCR1 | C-C Motif Chemokine Receptor 1 | GC03M046218 |
| SULT1E1 | Sulfotransferase Family 1E Member 1 | GC04M069841 |
| ACOX2 | Acyl-CoA Oxidase 2 | GC03M058490 |
| TSPAN32 | Tetraspanin 32 | GC11P002302 |
| MAGED2 | MAGE Family Member D2 | GC0XP054807 |
| DRD2 | Dopamine Receptor D2 | GC11M113409 |
| IL12B | Interleukin 12B | GC05M159314 |
| GPX2 | Glutathione Peroxidase 2 | GC14M064939 |
| SLC16A1 | Solute Carrier Family 16 Member 1 | GC01M112912 |
| ALOX15 | Arachidonate 15-Lipoxygenase | GC17M004630 |
| FAM215A | Family With Sequence Similarity 215 Member A | GC17P043917 |
| DNTT | DNA Nucleotidylexotransferase | GC10P096304 |
| NPY | Neuropeptide Y | GC07P024290 |
| ACY1 | Aminoacylase 1 | GC03P051983 |
| BLACAT1 | Bladder Cancer Associated Transcript 1 | GC01M205434 |
| TFAM | Transcription Factor A, Mitochondrial | GC10P058385 |
| CDC20 | Cell Division Cycle 20 | GC01P043358 |
| ALDH7A1 | Aldehyde Dehydrogenase 7 Family Member A1 | GC05M126541 |
| NR2F2 | Nuclear Receptor Subfamily 2 Group F Member 2 | GC15P096325 |
| MIR198 | MicroRNA 198 | GC03M120395 |
| DSG2 | Desmoglein 2 | GC18P031498 |
| MIR330 | MicroRNA 330 | GC19M045658 |
| SURF1 | SURF1 Cytochrome C Oxidase Assembly Factor | GC09M133351 |
| ERN1 | Endoplasmic Reticulum To Nucleus Signaling 1 | GC17M064039 |
| TNFAIP8L2 | TNF Alpha Induced Protein 8 Like 2 | GC01P151129 |
| NR3C2 | Nuclear Receptor Subfamily 3 Group C Member 2 | GC04M148078 |
| COL4A3 | Collagen Type IV Alpha 3 Chain | GC02P227164 |
| RAP1A | RAP1A, Member Of RAS Oncogene Family | GC01P111542 |
| EEF2 | Eukaryotic Translation Elongation Factor 2 | GC19M003976 |
| INPPL1 | Inositol Polyphosphate Phosphatase Like 1 | GC11P072223 |
| EIF3A | Eukaryotic Translation Initiation Factor 3 Subunit A | GC10M119034 |
| SCT | Secretin | GC11M000628 |
| OGFR | Opioid Growth Factor Receptor | GC20P062804 |
| NTN1 | Netrin 1 | GC17P009021 |
| RBL1 | RB Transcriptional Corepressor Like 1 | GC20M036996 |
| ALPL | Alkaline Phosphatase, Biomineralization Associated | GC01P021508 |
| ABI1 | Abl Interactor 1 | GC10M026746 |
| ANXA7 | Annexin A7 | GC10M073375 |
| SHC3 | SHC Adaptor Protein 3 | GC09M089005 |
| ITPR2 | Inositol 1,4,5-Trisphosphate Receptor Type 2 | GC12M026336 |
| PER1 | Period Circadian Regulator 1 | GC17M008761 |
| GATA2 | GATA Binding Protein 2 | GC03M128479 |
| HSP90AB1 | Heat Shock Protein 90 Alpha Family Class B Member 1 | GC06P044246 |
| F9 | Coagulation Factor IX | GC0XP139530 |
| HLA-DPA1 | Major Histocompatibility Complex, Class II, DP Alpha 1 | GC06M033064 |
| TSPAN8 | Tetraspanin 8 | GC12M071125 |
| TRADD | TNFRSF1A Associated Via Death Domain | GC16M067154 |
| DSG1 | Desmoglein 1 | GC18P031318 |
| FUT6 | Fucosyltransferase 6 | GC19M005830 |
| WNT2B | Wnt Family Member 2B | GC01P112466 |
| MAP2K7 | Mitogen-Activated Protein Kinase Kinase 7 | GC19P007903 |
| EPHB6 | EPH Receptor B6 | GC07P144739 |
| PAX6 | Paired Box 6 | GC11M031784 |
| CFL1 | Cofilin 1 | GC11M065823 |
| XRCC4 | X-Ray Repair Cross Complementing 4 | GC05P083077 |
| API5 | Apoptosis Inhibitor 5 | GC11P043311 |
| NOX4 | NADPH Oxidase 4 | GC11M089324 |
| NTSR1 | Neurotensin Receptor 1 | GC20P062708 |
| XAF1 | XIAP Associated Factor 1 | GC17P006757 |
| SCTR | Secretin Receptor | GC02M119439 |
| TRAF2 | TNF Receptor Associated Factor 2 | GC09P136881 |
| MIR509-1 | MicroRNA 509-1 | GC0XM147260 |
| E2F4 | E2F Transcription Factor 4 | GC16P067192 |
| ATF4 | Activating Transcription Factor 4 | GC22P039519 |
| TRIM25 | Tripartite Motif Containing 25 | GC17M056836 |
| SIRT7 | Sirtuin 7 | GC17M081911 |
| CLIC1 | Chloride Intracellular Channel 1 | GC06M032375 |
| FABP5 | Fatty Acid Binding Protein 5 | GC08P081282 |
| KLF9 | Kruppel Like Factor 9 | GC09M070384 |
| PTP4A3 | Protein Tyrosine Phosphatase 4A3 | GC08P141391 |
| MMP15 | Matrix Metallopeptidase 15 | GC16P058025 |
| XDH | Xanthine Dehydrogenase | GC02M031294 |
| PLEC | Plectin | GC08M143916 |
| AGR3 | Anterior Gradient 3, Protein Disulphide Isomerase Family Member | GC07M016854 |
| CTSC | Cathepsin C | GC11M088211 |
| F7 | Coagulation Factor VII | GC13P113105 |
| TRAP1 | TNF Receptor Associated Protein 1 | GC16M003652 |
| MT-CO3 | Mitochondrially Encoded Cytochrome C Oxidase III | GCMTP009209 |
| ITGB8 | Integrin Subunit Beta 8 | GC07P020329 |
| DST | Dystonin | GC06M056457 |
| LINC01194 | Long Intergenic Non-Protein Coding RNA 1194 | GC05P012578 |
| CD27 | CD27 Molecule | GC12P006608 |
| SNCA | Synuclein Alpha | GC04M089724 |
| FABP6 | Fatty Acid Binding Protein 6 | GC05P160187 |
| ABCC11 | ATP Binding Cassette Subfamily C Member 11 | GC16M048166 |
| FUT8 | Fucosyltransferase 8 | GC14P065411 |
| PDGFD | Platelet Derived Growth Factor D | GC11M103907 |
| FEZF1-AS1 | FEZF1 Antisense RNA 1 | GC07P122303 |
| CTNNBIP1 | Catenin Beta Interacting Protein 1 | GC01M009848 |
| P2RX7 | Purinergic Receptor P2X 7 | GC12P122630 |
| CYP8B1 | Cytochrome P450 Family 8 Subfamily B Member 1 | GC03M042856 |
| USP22 | Ubiquitin Specific Peptidase 22 | GC17M020999 |
| THBS4 | Thrombospondin 4 | GC05P079991 |
| BAG3 | BAG Cochaperone 3 | GC10P119651 |
| CCR9 | C-C Motif Chemokine Receptor 9 | GC03P045903 |
| PTN | Pleiotrophin | GC07M137227 |
| BTRC | Beta-Transducin Repeat Containing E3 Ubiquitin Protein Ligase | GC10P101354 |
| REEP5 | Receptor Accessory Protein 5 | GC05M112876 |
| HSPA9 | Heat Shock Protein Family A (Hsp70) Member 9 | GC05M138554 |
| MYOG | Myogenin | GC01M203083 |
| MT1G | Metallothionein 1G | GC16M056666 |
| FEN1 | Flap Structure-Specific Endonuclease 1 | GC11P061811 |
| BST2 | Bone Marrow Stromal Cell Antigen 2 | GC19M017403 |
| PROS1 | Protein S | GC03M093873 |
| PAWR | Pro-Apoptotic WT1 Regulator | GC12M079574 |
| CHD1L | Chromodomain Helicase DNA Binding Protein 1 Like | GC01P147242 |
| CLDN5 | Claudin 5 | GC22M019523 |
| PRSS8 | Serine Protease 8 | GC16M031132 |
| CCL3 | C-C Motif Chemokine Ligand 3 | GC17M036088 |
| F2RL3 | F2R Like Thrombin Or Trypsin Receptor 3 | GC19P016888 |
| OIT3 | Oncoprotein Induced Transcript 3 | GC10P072893 |
| PRMT1 | Protein Arginine Methyltransferase 1 | GC19P049675 |
| SERPINH1 | Serpin Family H Member 1 | GC11P075562 |
| SLC15A2 | Solute Carrier Family 15 Member 2 | GC03P121894 |
| DIRAS3 | DIRAS Family GTPase 3 | GC01M068045 |
| SMG1 | SMG1 Nonsense Mediated MRNA Decay Associated PI3K Related Kinase | GC16M018805 |
| ACO1 | Aconitase 1 | GC09P032374 |
| NME2 | NME/NM23 Nucleoside Diphosphate Kinase 2 | GC17P051165 |
| SOX8 | SRY-Box Transcription Factor 8 | GC16P000981 |
| ABCB7 | ATP Binding Cassette Subfamily B Member 7 | GC0XM075053 |
| MIR506 | MicroRNA 506 | GC0XM147230 |
| CCNA1 | Cyclin A1 | GC13P036431 |
| FGB | Fibrinogen Beta Chain | GC04P154564 |
| IFNAR1 | Interferon Alpha And Beta Receptor Subunit 1 | GC21P033324 |
| CISH | Cytokine Inducible SH2 Containing Protein | GC03M050618 |
| S100A14 | S100 Calcium Binding Protein A14 | GC01M153614 |
| SYNE1 | Spectrin Repeat Containing Nuclear Envelope Protein 1 | GC06M152121 |
| PRDX4 | Peroxiredoxin 4 | GC0XP023665 |
| STAT2 | Signal Transducer And Activator Of Transcription 2 | GC12M056341 |
| TGM1 | Transglutaminase 1 | GC14M024249 |
| EGFL7 | EGF Like Domain Multiple 7 | GC09P136658 |
| SOX1 | SRY-Box Transcription Factor 1 | GC13P112067 |
| MCRS1 | Microspherule Protein 1 | GC12M049557 |
| PSMD9 | Proteasome 26S Subunit, Non-ATPase 9 | GC12P122644 |
| CD247 | CD247 Molecule | GC01M167399 |
| FBXO11 | F-Box Protein 11 | GC02M047789 |
| GPBAR1 | G Protein-Coupled Bile Acid Receptor 1 | GC02P218259 |
| AMBP | Alpha-1-Microglobulin/Bikunin Precursor | GC09M114060 |
| TUBA1B | Tubulin Alpha 1b | GC12M049127 |
| TRPM7 | Transient Receptor Potential Cation Channel Subfamily M Member 7 | GC15M050552 |
| IAPP | Islet Amyloid Polypeptide | GC12P021354 |
| SLC2A2 | Solute Carrier Family 2 Member 2 | GC03M171024 |
| HGD | Homogentisate 1,2-Dioxygenase | GC03M120628 |
| ABCG8 | ATP Binding Cassette Subfamily G Member 8 | GC02P043828 |
| LAMA4 | Laminin Subunit Alpha 4 | GC06M112107 |
| ARMC10 | Armadillo Repeat Containing 10 | GC07P103074 |
| FUBP1 | Far Upstream Element Binding Protein 1 | GC01M077944 |
| GLO1 | Glyoxalase I | GC06M041312 |
| IL13RA2 | Interleukin 13 Receptor Subunit Alpha 2 | GC0XM115003 |
| TAGLN2 | Transgelin 2 | GC01M159918 |
| CUL4A | Cullin 4A | GC13P113208 |
| CSNK1A1 | Casein Kinase 1 Alpha 1 | GC05M149492 |
| PRDX6 | Peroxiredoxin 6 | GC01P173477 |
| MIR345 | MicroRNA 345 | GC14P100307 |
| TNXA | Tenascin XA (Pseudogene) | GC06M032396 |
| MIR708 | MicroRNA 708 | GC11M079402 |
| BTG2 | BTG Anti-Proliferation Factor 2 | GC01P203305 |
| PANTR1 | POU3F3 Adjacent Non-Coding Transcript 1 | GC02M104656 |
| KNG1 | Kininogen 1 | GC03P186717 |
| SPOP | Speckle Type BTB/POZ Protein | GC17M049598 |
| BCYRN1 | Brain Cytoplasmic RNA 1 | GC02P047331 |
| PPY | Pancreatic Polypeptide | GC17M043940 |
| ACOX1 | Acyl-CoA Oxidase 1 | GC17M075941 |
| FOXP1 | Forkhead Box P1 | GC03M070926 |
| HOXB7 | Homeobox B7 | GC17M048607 |
| ALPI | Alkaline Phosphatase, Intestinal | GC02P232456 |
| MBD2 | Methyl-CpG Binding Domain Protein 2 | GC18M054151 |
| NR4A1 | Nuclear Receptor Subfamily 4 Group A Member 1 | GC12P052022 |
| ST3GAL6 | ST3 Beta-Galactoside Alpha-2,3-Sialyltransferase 6 | GC03P098732 |
| SETDB1 | SET Domain Bifurcated Histone Lysine Methyltransferase 1 | GC01P150926 |
| CUL3 | Cullin 3 | GC02M224470 |
| SPAG5 | Sperm Associated Antigen 5 | GC17M028577 |
| MIR625 | MicroRNA 625 | GC14P065471 |
| PRKX | Protein Kinase X-Linked | GC0XM003604 |
| HEY2 | Hes Related Family BHLH Transcription Factor With YRPW Motif 2 | GC06P125730 |
| TIMP4 | TIMP Metallopeptidase Inhibitor 4 | GC03M012153 |
| VAV2 | Vav Guanine Nucleotide Exchange Factor 2 | GC09M133761 |
| PNP | Purine Nucleoside Phosphorylase | GC14P020468 |
| CD1D | CD1d Molecule | GC01P158178 |
| MIR92A2 | MicroRNA 92a-2 | GC0XM134215 |
| SERPINA7 | Serpin Family A Member 7 | GC0XM106032 |
| ACADVL | Acyl-CoA Dehydrogenase Very Long Chain | GC17P007219 |
| NEU1 | Neuraminidase 1 | GC06M031857 |
| MIR134 | MicroRNA 134 | GC14P104437 |
| AKR1A1 | Aldo-Keto Reductase Family 1 Member A1 | GC01P045550 |
| CBL | Cbl Proto-Oncogene | GC11P119206 |
| KIF11 | Kinesin Family Member 11 | GC10P092593 |
| IGFBP6 | Insulin Like Growth Factor Binding Protein 6 | GC12P053097 |
| DSC3 | Desmocollin 3 | GC18M030990 |
| CDK5RAP3 | CDK5 Regulatory Subunit Associated Protein 3 | GC17P047967 |
| SLC39A14 | Solute Carrier Family 39 Member 14 | GC08P022367 |
| ATP8A2 | ATPase Phospholipid Transporting 8A2 | GC13P025373 |
| MAML1 | Mastermind Like Transcriptional Coactivator 1 | GC05P179732 |
| PAK4 | P21 (RAC1) Activated Kinase 4 | GC19P039125 |
| MIR630 | MicroRNA 630 | GC15P072587 |
| TCF7L1 | Transcription Factor 7 Like 1 | GC02P085133 |
| MIR452 | MicroRNA 452 | GC0XM151959 |
| PTX3 | Pentraxin 3 | GC03P157436 |
| DLD | Dihydrolipoamide Dehydrogenase | GC07P107890 |
| SBF2-AS1 | SBF2 Antisense RNA 1 | GC11P009758 |
| DNAJC6 | DnaJ Heat Shock Protein Family (Hsp40) Member C6 | GC01P065248 |
| BRINP1 | BMP/Retinoic Acid Inducible Neural Specific 1 | GC09M119153 |
| TBX3 | T-Box Transcription Factor 3 | GC12M114670 |
| FZD5 | Frizzled Class Receptor 5 | GC02M207762 |
| TERF2 | Telomeric Repeat Binding Factor 2 | GC16M069355 |
| CRKL | CRK Like Proto-Oncogene, Adaptor Protein | GC22P020917 |
| MYOF | Myoferlin | GC10M093306 |
| PER2 | Period Circadian Regulator 2 | GC02M238244 |
| TMSB4X | Thymosin Beta 4 X-Linked | GC0XP012975 |
| RGS22 | Regulator Of G Protein Signaling 22 | GC08M099960 |
| GPI | Glucose-6-Phosphate Isomerase | GC19P034359 |
| LIN28A | Lin-28 Homolog A | GC01P026410 |
| STK33 | Serine/Threonine Kinase 33 | GC11M008391 |
| TERF1 | Telomeric Repeat Binding Factor 1 | GC08P073003 |
| RAB23 | RAB23, Member RAS Oncogene Family | GC06M057161 |
| PGD | Phosphogluconate Dehydrogenase | GC01P010398 |
| TNS4 | Tensin 4 | GC17M040475 |
| BIN1 | Bridging Integrator 1 | GC02M127048 |
| CGB5 | Chorionic Gonadotropin Subunit Beta 5 | GC19P049043 |
| CSNK2B | Casein Kinase 2 Beta | GC06P033402 |
| HELLS | Helicase, Lymphoid Specific | GC10P094501 |
| TACC3 | Transforming Acidic Coiled-Coil Containing Protein 3 | GC04P001723 |
| HBB-LCR | Beta-Globin Locus Control Region | GC11U902985 |
| FOXQ1 | Forkhead Box Q1 | GC06P001312 |
| CBX4 | Chromobox 4 | GC17M079833 |
| PSMG2 | Proteasome Assembly Chaperone 2 | GC18P013073 |
| GOLPH3 | Golgi Phosphoprotein 3 | GC05M032124 |
| PSMA7 | Proteasome 20S Subunit Alpha 7 | GC20M062136 |
| SND1 | Staphylococcal Nuclease And Tudor Domain Containing 1 | GC07P127652 |
| ACKR2 | Atypical Chemokine Receptor 2 | GC03P042804 |
| RPL6 | Ribosomal Protein L6 | GC12M112320 |
| MVK | Mevalonate Kinase | GC12P109573 |
| PVR | PVR Cell Adhesion Molecule | GC19P044644 |
| SERPINA2 | Serpin Family A Member 2 (Gene/Pseudogene) | GC14M094363 |
| SGK3 | Serum/Glucocorticoid Regulated Kinase Family Member 3 | GC08P066671 |
| FZD1 | Frizzled Class Receptor 1 | GC07P091264 |
| PHLPP2 | PH Domain And Leucine Rich Repeat Protein Phosphatase 2 | GC16M071637 |
| ASNS | Asparagine Synthetase (Glutamine-Hydrolyzing) | GC07M097854 |
| CTBP1 | C-Terminal Binding Protein 1 | GC04M001211 |
| TXNIP | Thioredoxin Interacting Protein | GC01M145992 |
| UCK2 | Uridine-Cytidine Kinase 2 | GC01P165796 |
| EIF6 | Eukaryotic Translation Initiation Factor 6 | GC20M035278 |
| FZD8 | Frizzled Class Receptor 8 | GC10M035638 |
| MT-ND6 | Mitochondrially Encoded NADH:Ubiquinone Oxidoreductase Core Subunit 6 | GCMTM014151 |
| DBH | Dopamine Beta-Hydroxylase | GC09P133636 |
| FLI1 | Fli-1 Proto-Oncogene, ETS Transcription Factor | GC11P128686 |
| GCLC | Glutamate-Cysteine Ligase Catalytic Subunit | GC06M053497 |
| KLF17 | Kruppel Like Factor 17 | GC01P044048 |
| GPNMB | Glycoprotein Nmb | GC07P023238 |
| MIR615 | MicroRNA 615 | GC12P054033 |
| H4C1 | H4 Clustered Histone 1 | GC06P026036 |
| LYPD3 | LY6/PLAUR Domain Containing 3 | GC19M043460 |
| CPA4 | Carboxypeptidase A4 | GC07P130293 |
| NFATC1 | Nuclear Factor Of Activated T Cells 1 | GC18P079395 |
| ERBIN | Erbb2 Interacting Protein | GC05P065931 |
| CDR1-AS | CDR1 Antisense RNA | GC0XU902169 |
| ATP11C | ATPase Phospholipid Transporting 11C | GC0XM139726 |
| FUT3 | Fucosyltransferase 3 (Lewis Blood Group) | GC19M005843 |
| HAND2-AS1 | HAND2 Antisense RNA 1 | GC04P173527 |
| HSD11B2 | Hydroxysteroid 11-Beta Dehydrogenase 2 | GC16P067433 |
| UGT2B7 | UDP Glucuronosyltransferase Family 2 Member B7 | GC04P069051 |
| NRIP1 | Nuclear Receptor Interacting Protein 1 | GC21M014961 |
| LINC00963 | Long Intergenic Non-Protein Coding RNA 963 | GC09P129483 |
| VIPR1 | Vasoactive Intestinal Peptide Receptor 1 | GC03P042490 |
| MAP3K7 | Mitogen-Activated Protein Kinase Kinase Kinase 7 | GC06M090513 |
| TGFBI | Transforming Growth Factor Beta Induced | GC05P136027 |
| FER | FER Tyrosine Kinase | GC05P108747 |
| CANX | Calnexin | GC05P179678 |
| PCK1 | Phosphoenolpyruvate Carboxykinase 1 | GC20P057561 |
| HUWE1 | HECT, UBA And WWE Domain Containing E3 Ubiquitin Protein Ligase 1 | GC0XM053532 |
| TPM3 | Tropomyosin 3 | GC01M154127 |
| SRRT | Serrate, RNA Effector Molecule | GC07P100875 |
| HJURP | Holliday Junction Recognition Protein | GC02M233834 |
| MELK | Maternal Embryonic Leucine Zipper Kinase | GC09P036572 |
| FLNA | Filamin A | GC0XM154348 |
| TAPBP | TAP Binding Protein | GC06M033299 |
| WNT7A | Wnt Family Member 7A | GC03M015408 |
| MIR519D | MicroRNA 519d | GC19P053713 |
| APOH | Apolipoprotein H | GC17M066212 |
| MT1M | Metallothionein 1M | GC16P056632 |
| LTBP1 | Latent Transforming Growth Factor Beta Binding Protein 1 | GC02P032946 |
| S100A11 | S100 Calcium Binding Protein A11 | GC01M152032 |
| KLF2 | Kruppel Like Factor 2 | GC19P022206 |
| RALBP1 | RalA Binding Protein 1 | GC18P009465 |
| RBM38 | RNA Binding Motif Protein 38 | GC20P057391 |
| MSI1 | Musashi RNA Binding Protein 1 | GC12M120341 |
| NCAN | Neurocan | GC19P022265 |
| BMPR2 | Bone Morphogenetic Protein Receptor Type 2 | GC02P202376 |
| KCNH1 | Potassium Voltage-Gated Channel Subfamily H Member 1 | GC01M210678 |
| PDGFC | Platelet Derived Growth Factor C | GC04M156760 |
| TAB1 | TGF-Beta Activated Kinase 1 (MAP3K7) Binding Protein 1 | GC22P039401 |
| SP3 | Sp3 Transcription Factor | GC02M173882 |
| MIR302B | MicroRNA 302b | GC04M112676 |
| CYP27B1 | Cytochrome P450 Family 27 Subfamily B Member 1 | GC12M057757 |
| HLCS | Holocarboxylase Synthetase | GC21M036750 |
| UROS | Uroporphyrinogen III Synthase | GC10M125784 |
| MVD | Mevalonate Diphosphate Decarboxylase | GC16M088651 |
| EMP2 | Epithelial Membrane Protein 2 | GC16M010541 |
| PCK2 | Phosphoenolpyruvate Carboxykinase 2, Mitochondrial | GC14P024094 |
| MIR99B | MicroRNA 99b | GC19P051692 |
| MMP16 | Matrix Metallopeptidase 16 | GC08M088032 |
| MIR211 | MicroRNA 211 | GC15M031065 |
| IGFBP4 | Insulin Like Growth Factor Binding Protein 4 | GC17P040443 |
| NUPR1 | Nuclear Protein 1, Transcriptional Regulator | GC16M028548 |
| PNKD | PNKD Metallo-Beta-Lactamase Domain Containing | GC02P218270 |
| MYBL2 | MYB Proto-Oncogene Like 2 | GC20P043667 |
| ACER3 | Alkaline Ceramidase 3 | GC11P076860 |
| SSTR3 | Somatostatin Receptor 3 | GC22M037204 |
| MAGEA9 | MAGE Family Member A9 | GC0XP149781 |
| ADPRH | ADP-Ribosylarginine Hydrolase | GC03P119579 |
| TRIM26 | Tripartite Motif Containing 26 | GC06M030184 |
| CCNB2 | Cyclin B2 | GC15P059105 |
| ADGRE5 | Adhesion G Protein-Coupled Receptor E5 | GC19P014381 |
| MIR101-2 | MicroRNA 101-2 | GC09P004864 |
| SLC9A1 | Solute Carrier Family 9 Member A1 | GC01M027109 |
| TNFSF13 | TNF Superfamily Member 13 | GC17P007558 |
| MIR371A | MicroRNA 371a | GC19P053787 |
| RSS | Russell Silver Syndrome | GC17U901765 |
| PSC | Cholangitis, Primary Sclerosing | GC03U901834 |
| PBC2 | Biliary Cirrhosis, Primary, 2 | GC06U901769 |
| PBC3 | Biliary Cirrhosis, Primary, 3 | GC01U902479 |
| PBC4 | Biliary Cirrhosis, Primary, 4 | GC07U903249 |
| PBC5 | Biliary Cirrhosis, Primary, 5 | GC17U901708 |
| SNHG7 | Small Nucleolar RNA Host Gene 7 | GC09M136721 |
| IFI16 | Interferon Gamma Inducible Protein 16 | GC01P158969 |
| CAPNS1 | Calpain Small Subunit 1 | GC19P037745 |
| MB | Myoglobin | GC22M035606 |
| MAGT1 | Magnesium Transporter 1 | GC0XM077826 |
| ADRB2 | Adrenoceptor Beta 2 | GC05P148825 |
| LGALS4 | Galectin 4 | GC19M041436 |
| MIR339 | MicroRNA 339 | GC07M001022 |
| MARCKS | Myristoylated Alanine Rich Protein Kinase C Substrate | GC06P113857 |
| COL1A2 | Collagen Type I Alpha 2 Chain | GC07P094394 |
| EGLN2 | Egl-9 Family Hypoxia Inducible Factor 2 | GC19P040799 |
| PKLR | Pyruvate Kinase L/R | GC01M155289 |
| CCDC88A | Coiled-Coil Domain Containing 88A | GC02M055287 |
| GPX4 | Glutathione Peroxidase 4 | GC19P001103 |
| CCL22 | C-C Motif Chemokine Ligand 22 | GC16P057359 |
| CD14 | CD14 Molecule | GC05M140631 |
| ADCY10 | Adenylate Cyclase 10 | GC01M167809 |
| TMEFF2 | Transmembrane Protein With EGF Like And Two Follistatin Like Domains 2 | GC02M191950 |
| DSC2 | Desmocollin 2 | GC18M031058 |
| PRDX5 | Peroxiredoxin 5 | GC11P064317 |
| TOP2B | DNA Topoisomerase II Beta | GC03M025598 |
| BCR | BCR Activator Of RhoGEF And GTPase | GC22P023179 |
| PLIN1 | Perilipin 1 | GC15M089664 |
| AIRE | Autoimmune Regulator | GC21P044285 |
| FKSG49 | Hepatocellular Carcinoma-Associated Antigen HCA25b | GC05U901010 |
| FXYD5 | FXYD Domain Containing Ion Transport Regulator 5 | GC19P035154 |
| CCL26 | C-C Motif Chemokine Ligand 26 | GC07M075769 |
| TTF2 | Transcription Termination Factor 2 | GC01P117060 |
| SSTR4 | Somatostatin Receptor 4 | GC20P023035 |
| EHHADH | Enoyl-CoA Hydratase And 3-Hydroxyacyl CoA Dehydrogenase | GC03M185190 |
| CDK5 | Cyclin Dependent Kinase 5 | GC07M151053 |
| CX3CR1 | C-X3-C Motif Chemokine Receptor 1 | GC03M039279 |
| MEF2D | Myocyte Enhancer Factor 2D | GC01M156463 |
| SIRPA | Signal Regulatory Protein Alpha | GC20P001894 |
| TET1 | Tet Methylcytosine Dioxygenase 1 | GC10P068560 |
| IL1RAPL2 | Interleukin 1 Receptor Accessory Protein Like 2 | GC0XP104566 |
| SPRY2 | Sprouty RTK Signaling Antagonist 2 | GC13M080335 |
| FERMT2 | Fermitin Family Member 2 | GC14M052857 |
| CKS2 | CDC28 Protein Kinase Regulatory Subunit 2 | GC09P089311 |
| KAT5 | Lysine Acetyltransferase 5 | GC11P065711 |
| MIR194-1 | MicroRNA 194-1 | GC01M220118 |
| CBS | Cystathionine Beta-Synthase | GC21M043053 |
| RCVRN | Recoverin | GC17M009896 |
| FLNB | Filamin B | GC03P058008 |
| SPTBN1 | Spectrin Beta, Non-Erythrocytic 1 | GC02P054456 |
| SCD | Stearoyl-CoA Desaturase | GC10P100347 |
| MIR365A | MicroRNA 365a | GC16P014309 |
| SLC1A5 | Solute Carrier Family 1 Member 5 | GC19M046760 |
| E2F5 | E2F Transcription Factor 5 | GC08P085177 |
| P4HB | Prolyl 4-Hydroxylase Subunit Beta | GC17M081843 |
| PAK6 | P21 (RAC1) Activated Kinase 6 | GC15P040217 |
| ID4 | Inhibitor Of DNA Binding 4, HLH Protein | GC06P019837 |
| PFN1 | Profilin 1 | GC17M004945 |
| CCT3 | Chaperonin Containing TCP1 Subunit 3 | GC01M156308 |
| FXYD2 | FXYD Domain Containing Ion Transport Regulator 2 | GC11M117800 |
| ST3GAL4 | ST3 Beta-Galactoside Alpha-2,3-Sialyltransferase 4 | GC11P126355 |
| TET2 | Tet Methylcytosine Dioxygenase 2 | GC04P105145 |
| PI4KA | Phosphatidylinositol 4-Kinase Alpha | GC22M020707 |
| PELP1 | Proline, Glutamate And Leucine Rich Protein 1 | GC17M004669 |
| MT-ND5 | Mitochondrially Encoded NADH:Ubiquinone Oxidoreductase Core Subunit 5 | GCMTP012339 |
| STUB1 | STIP1 Homology And U-Box Containing Protein 1 | GC16P001253 |
| SYNM | Synemin | GC15P099098 |
| MT-ND1 | Mitochondrially Encoded NADH:Ubiquinone Oxidoreductase Core Subunit 1 | GCMTP003309 |
| MAPK12 | Mitogen-Activated Protein Kinase 12 | GC22M050246 |
| CORO1C | Coronin 1C | GC12M108645 |
| LPAR2 | Lysophosphatidic Acid Receptor 2 | GC19M019624 |
| CD163 | CD163 Molecule | GC12M007471 |
| RUVBL2 | RuvB Like AAA ATPase 2 | GC19P048993 |
| AGL | Amylo-Alpha-1, 6-Glucosidase, 4-Alpha-Glucanotransferase | GC01P099850 |
| MIR637 | MicroRNA 637 | GC19M003961 |
| FCGBP | Fc Fragment Of IgG Binding Protein | GC19M039863 |
| IGSF8 | Immunoglobulin Superfamily Member 8 | GC01M160061 |
| PIR | Pirin | GC0XM015402 |
| BLCAP | BLCAP Apoptosis Inducing Factor | GC20M037492 |
| RDX | Radixin | GC11M109864 |
| CAV2 | Caveolin 2 | GC07P116287 |
| TBL1XR1 | TBL1X Receptor 1 | GC03M177019 |
| HNRNPD | Heterogeneous Nuclear Ribonucleoprotein D | GC04M082352 |
| TTC33 | Tetratricopeptide Repeat Domain 33 | GC05M040512 |
| MIR512-1 | MicroRNA 512-1 | GC19P054092 |
| PPHLN1 | Periphilin 1 | GC12P042238 |
| RARRES1 | Retinoic Acid Receptor Responder 1 | GC03M158696 |
| F13A1 | Coagulation Factor XIII A Chain | GC06M006144 |
| AGRN | Agrin | GC01P001020 |
| ING4 | Inhibitor Of Growth Family Member 4 | GC12M006650 |
| PTPN9 | Protein Tyrosine Phosphatase Non-Receptor Type 9 | GC15M075463 |
| CPA1 | Carboxypeptidase A1 | GC07P130380 |
| MIR874 | MicroRNA 874 | GC05M137647 |
| SCG5 | Secretogranin V | GC15P032641 |
| SUCLA2 | Succinate-CoA Ligase ADP-Forming Subunit Beta | GC13M047745 |
| FBLN5 | Fibulin 5 | GC14M091869 |
| VKORC1 | Vitamin K Epoxide Reductase Complex Subunit 1 | GC16M031105 |
| RPS6KA3 | Ribosomal Protein S6 Kinase A3 | GC0XM020149 |
| LAG3 | Lymphocyte Activating 3 | GC12P006857 |
| UPF1 | UPF1 RNA Helicase And ATPase | GC19P018831 |
| AOPEP | Aminopeptidase O (Putative) | GC09P094728 |
| IL36A | Interleukin 36 Alpha | GC02P113005 |
| PLPBP | Pyridoxal Phosphate Binding Protein | GC08P037763 |
| HGFAC | HGF Activator | GC04P003443 |
| CLEC3B | C-Type Lectin Domain Family 3 Member B | GC03P045293 |
| ING5 | Inhibitor Of Growth Family Member 5 | GC02P241702 |
| EEF1G | Eukaryotic Translation Elongation Factor 1 Gamma | GC11M062560 |
| SLIT2 | Slit Guidance Ligand 2 | GC04P020287 |
| CTSK | Cathepsin K | GC01M150796 |
| BAGE | B Melanoma Antigen | GC21U900396 |
| MIR383 | MicroRNA 383 | GC08M014853 |
| TIA1 | TIA1 Cytotoxic Granule Associated RNA Binding Protein | GC02M070209 |
| CCL15 | C-C Motif Chemokine Ligand 15 | GC17M035996 |
| BCL2L2 | BCL2 Like 2 | GC14P025033 |
| HPR | Haptoglobin-Related Protein | GC16P072097 |
| EPHA5 | EPH Receptor A5 | GC04M065319 |
| RXRG | Retinoid X Receptor Gamma | GC01M165401 |
| MGAT3 | Beta-1,4-Mannosyl-Glycoprotein 4-Beta-N-Acetylglucosaminyltransferase | GC22P039447 |
| PSMC4 | Proteasome 26S Subunit, ATPase 4 | GC19P039972 |
| LRRFIP2 | LRR Binding FLII Interacting Protein 2 | GC03M037052 |
| MIR498 | MicroRNA 498 | GC19P054090 |
| HBA1 | Hemoglobin Subunit Alpha 1 | GC16P001212 |
| TARS1 | Threonyl-TRNA Synthetase 1 | GC05P033441 |
| WASF2 | WASP Family Member 2 | GC01M027404 |
| LCAT | Lecithin-Cholesterol Acyltransferase | GC16M067939 |
| RXRB | Retinoid X Receptor Beta | GC06M033193 |
| ANLN | Anillin Actin Binding Protein | GC07P036389 |
| MADCAM1 | Mucosal Vascular Addressin Cell Adhesion Molecule 1 | GC19P000499 |
| PTPRO | Protein Tyrosine Phosphatase Receptor Type O | GC12P015366 |
| FAM3C | FAM3 Metabolism Regulating Signaling Molecule C | GC07M121349 |
| STIP1 | Stress Induced Phosphoprotein 1 | GC11P064203 |
| MIR362 | MicroRNA 362 | GC0XP050056 |
| ACTN1 | Actinin Alpha 1 | GC14M068874 |
| EFEMP1 | EGF Containing Fibulin Extracellular Matrix Protein 1 | GC02M055865 |
| ATP11B | ATPase Phospholipid Transporting 11B (Putative) | GC03P182793 |
| SETX | Senataxin | GC09M132261 |
| LPAR1 | Lysophosphatidic Acid Receptor 1 | GC09M110873 |
| CXCL2 | C-X-C Motif Chemokine Ligand 2 | GC04M074097 |
| TRIM29 | Tripartite Motif Containing 29 | GC11M120111 |
| RND3 | Rho Family GTPase 3 | GC02M150468 |
| IL7R | Interleukin 7 Receptor | GC05P035852 |
| RPLP0 | Ribosomal Protein Lateral Stalk Subunit P0 | GC12M120196 |
| SPHK2 | Sphingosine Kinase 2 | GC19P048619 |
| LEPQTL1 | Leptin, Serum Levels Of | GC02U903086 |
| MIR1202 | MicroRNA 1202 | GC06P155946 |
| DLGAP5 | DLG Associated Protein 5 | GC14M055148 |
| AHNAK | AHNAK Nucleoprotein | GC11M062511 |
| FOXC2 | Forkhead Box C2 | GC16P086567 |
| SERPINI1 | Serpin Family I Member 1 | GC03P167735 |
| NOVA1 | NOVA Alternative Splicing Regulator 1 | GC14M026443 |
| PTH1R | Parathyroid Hormone 1 Receptor | GC03P046877 |
| POTEKP | POTE Ankyrin Domain Family Member K, Pseudogene | GC02P131591 |
| HBA2 | Hemoglobin Subunit Alpha 2 | GC16P001213 |
| GNL3 | G Protein Nucleolar 3 | GC03P052681 |
| MIR138-2 | MicroRNA 138-2 | GC16P056859 |
| TNFRSF9 | TNF Receptor Superfamily Member 9 | GC01M007915 |
| LINC00052 | Long Intergenic Non-Protein Coding RNA 52 | GC15P087576 |
| RAB5A | RAB5A, Member RAS Oncogene Family | GC03P019963 |
| UGT1A9 | UDP Glucuronosyltransferase Family 1 Member A9 | GC02P233671 |
| EPHA4 | EPH Receptor A4 | GC02M221418 |
| MR1 | Major Histocompatibility Complex, Class I-Related | GC01P181033 |
| TRIP13 | Thyroid Hormone Receptor Interactor 13 | GC05P000892 |
| POMGNT2 | Protein O-Linked Mannose N-Acetylglucosaminyltransferase 2 (Beta 1,4-) | GC03M043121 |
| AREL1 | Apoptosis Resistant E3 Ubiquitin Protein Ligase 1 | GC14M074653 |
| SRPRB | SRP Receptor Subunit Beta | GC03P133784 |
| TTK | TTK Protein Kinase | GC06P080003 |
| VIL1 | Villin 1 | GC02P218419 |
| TNFRSF25 | TNF Receptor Superfamily Member 25 | GC01M006460 |
| UBE4B | Ubiquitination Factor E4B | GC01P010032 |
| RGN | Regucalcin | GC0XP047080 |
| WNT16 | Wnt Family Member 16 | GC07P121325 |
| AGTR2 | Angiotensin II Receptor Type 2 | GC0XP116170 |
| CYBRD1 | Cytochrome B Reductase 1 | GC02P171522 |
| KMT2B | Lysine Methyltransferase 2B | GC19P037722 |
| HK1 | Hexokinase 1 | GC10P069269 |
| KMT2D | Lysine Methyltransferase 2D | GC12M049018 |
| FLOT2 | Flotillin 2 | GC17M029702 |
| GATA1 | GATA Binding Protein 1 | GC0XP048786 |
| IL23A | Interleukin 23 Subunit Alpha | GC12P056335 |
| TUSC3 | Tumor Suppressor Candidate 3 | GC08P015417 |
| CLOCK | Clock Circadian Regulator | GC04M055427 |
| SKA1 | Spindle And Kinetochore Associated Complex Subunit 1 | GC18P050374 |
| PSMD4 | Proteasome 26S Subunit, Non-ATPase 4 | GC01P151227 |
| POU5F1B | POU Class 5 Homeobox 1B | GC08P127322 |
| CLCA4 | Chloride Channel Accessory 4 | GC01P086547 |
| TUBA1A | Tubulin Alpha 1a | GC12M049184 |
| VASH1 | Vasohibin 1 | GC14P076761 |
| MLKL | Mixed Lineage Kinase Domain Like Pseudokinase | GC16M074672 |
| SMAD1 | SMAD Family Member 1 | GC04P145481 |
| ACHE | Acetylcholinesterase (Cartwright Blood Group) | GC07M100889 |
| RELB | RELB Proto-Oncogene, NF-KB Subunit | GC19P045002 |
| MIR744 | MicroRNA 744 | GC17P012081 |
| VRK1 | VRK Serine/Threonine Kinase 1 | GC14P096797 |
| DCTN1 | Dynactin Subunit 1 | GC02M074361 |
| MIR501 | MicroRNA 501 | GC0XP050057 |
| OSCP1 | Organic Solute Carrier Partner 1 | GC01M036415 |
| DCXR | Dicarbonyl And L-Xylulose Reductase | GC17M082036 |
| CENPF | Centromere Protein F | GC01P214603 |
| TNFRSF12A | TNF Receptor Superfamily Member 12A | GC16P003018 |
| ARFGAP1 | ADP Ribosylation Factor GTPase Activating Protein 1 | GC20P063272 |
| EIF3H | Eukaryotic Translation Initiation Factor 3 Subunit H | GC08M116642 |
| AQP9 | Aquaporin 9 | GC15P058138 |
| WNT6 | Wnt Family Member 6 | GC02P218859 |
| AGGF1 | Angiogenic Factor With G-Patch And FHA Domains 1 | GC05P077029 |
| CX3CL1 | C-X3-C Motif Chemokine Ligand 1 | GC16P057372 |
| CCKAR | Cholecystokinin A Receptor | GC04M026483 |
| AGMAT | Agmatinase | GC01M015571 |
| DDB1 | Damage Specific DNA Binding Protein 1 | GC11M061301 |
| TSPAN1 | Tetraspanin 1 | GC01P046175 |
| SEPTIN9 | Septin 9 | GC17P077282 |
| AGAP2-AS1 | AGAP2 Antisense RNA 1 | GC12P057726 |
| PBOV1 | Prostate And Breast Cancer Overexpressed 1 | GC06M138215 |
| FZR1 | Fizzy And Cell Division Cycle 20 Related 1 | GC19P003506 |
| LOC111589215 | BRCA1 Promoter Region | GC17U902746 |
| KDM6B | Lysine Demethylase 6B | GC17P007834 |
| PFKP | Phosphofructokinase, Platelet | GC10P003066 |
| ATOX1 | Antioxidant 1 Copper Chaperone | GC05M151743 |
| RGMB-AS1 | RGMB Antisense RNA 1 | GC05M098769 |
| KLHL22 | Kelch Like Family Member 22 | GC22M020442 |
| RNF169 | Ring Finger Protein 169 | GC11P074797 |
| GPATCH4 | G-Patch Domain Containing 4 | GC01M156565 |
| MED16 | Mediator Complex Subunit 16 | GC19M000868 |
| CAPRIN2 | Caprin Family Member 2 | GC12M030709 |
| FLOT1 | Flotillin 1 | GC06M030808 |
| CCN6 | Cellular Communication Network Factor 6 | GC06P112053 |
| RAP1B | RAP1B, Member Of RAS Oncogene Family | GC12P068610 |
| PLIN3 | Perilipin 3 | GC19M004839 |
| CAPN2 | Calpain 2 | GC01P223701 |
| DUSP19 | Dual Specificity Phosphatase 19 | GC02P183078 |
| SLC25A5 | Solute Carrier Family 25 Member 5 | GC0XP119468 |
| IFIH1 | Interferon Induced With Helicase C Domain 1 | GC02M162267 |
| CCL25 | C-C Motif Chemokine Ligand 25 | GC19P008117 |
| SRSF1 | Serine And Arginine Rich Splicing Factor 1 | GC17M058000 |
| POLR2E | RNA Polymerase II Subunit E | GC19M001086 |
| TEAD1 | TEA Domain Transcription Factor 1 | GC11P012674 |
| IST1 | IST1 Factor Associated With ESCRT-III | GC16P071846 |
| TRIM31 | Tripartite Motif Containing 31 | GC06M030652 |
| SMURF2 | SMAD Specific E3 Ubiquitin Protein Ligase 2 | GC17M064542 |
| SESN2 | Sestrin 2 | GC01P028270 |
| CD47 | CD47 Molecule | GC03M108043 |
| MYCT1 | MYC Target 1 | GC06P152697 |
| ELP3 | Elongator Acetyltransferase Complex Subunit 3 | GC08P028089 |
| SPA17 | Sperm Autoantigenic Protein 17 | GC11P124673 |
| GAS6 | Growth Arrest Specific 6 | GC13M113820 |
| CXCR6 | C-X-C Motif Chemokine Receptor 6 | GC03P045982 |
| DACT2 | Dishevelled Binding Antagonist Of Beta Catenin 2 | GC06M168292 |
| BHLHE40 | Basic Helix-Loop-Helix Family Member E40 | GC03P004997 |
| MT2A | Metallothionein 2A | GC16P056626 |
| MIR511 | MicroRNA 511 | GC10P017845 |
| TBCD | Tubulin Folding Cofactor D | GC17P082752 |
| FAT1 | FAT Atypical Cadherin 1 | GC04M186587 |
| LRP5 | LDL Receptor Related Protein 5 | GC11P068298 |
| CDIPT | CDP-Diacylglycerol--Inositol 3-Phosphatidyltransferase | GC16M029870 |
| TUBB2A | Tubulin Beta 2A Class IIa | GC06M003153 |
| RPS6KB2 | Ribosomal Protein S6 Kinase B2 | GC11P067428 |
| LOC100505549 | Uncharacterized LOC100505549 | GC18P057630 |
| AQP5 | Aquaporin 5 | GC12P049961 |
| COMMD7 | COMM Domain Containing 7 | GC20M032702 |
| SLC22A1 | Solute Carrier Family 22 Member 1 | GC06P160121 |
| LPAR3 | Lysophosphatidic Acid Receptor 3 | GC01M084811 |
| TIE1 | Tyrosine Kinase With Immunoglobulin Like And EGF Like Domains 1 | GC01P043300 |
| DEPTOR | DEP Domain Containing MTOR Interacting Protein | GC08P119873 |
| EIF4B | Eukaryotic Translation Initiation Factor 4B | GC12P053006 |
| APOM | Apolipoprotein M | GC06P033408 |
| FCGR3A | Fc Fragment Of IgG Receptor IIIa | GC01M161541 |
| PRNP | Prion Protein | GC20P004686 |
| HPS3 | HPS3 Biogenesis Of Lysosomal Organelles Complex 2 Subunit 1 | GC03P149129 |
| DDX5 | DEAD-Box Helicase 5 | GC17M064498 |
| LCK | LCK Proto-Oncogene, Src Family Tyrosine Kinase | GC01P032251 |
| GLUD1 | Glutamate Dehydrogenase 1 | GC10M087050 |
| HSPG2 | Heparan Sulfate Proteoglycan 2 | GC01M021822 |
| SLC2A9 | Solute Carrier Family 2 Member 9 | GC04M009772 |
| BTG1 | BTG Anti-Proliferation Factor 1 | GC12M092140 |
| VDAC1 | Voltage Dependent Anion Channel 1 | GC05M133975 |
| ETV7 | ETS Variant Transcription Factor 7 | GC06M041299 |
| MDH2 | Malate Dehydrogenase 2 | GC07P076048 |
| MSI2 | Musashi RNA Binding Protein 2 | GC17P057255 |
| NISCH | Nischarin | GC03P052455 |
| MMD | Monocyte To Macrophage Differentiation Associated | GC17M055392 |
| EPHB1 | EPH Receptor B1 | GC03P134598 |
| RAD21 | RAD21 Cohesin Complex Component | GC08M116846 |
| PPP2CA | Protein Phosphatase 2 Catalytic Subunit Alpha | GC05M134194 |
| PLXNA3 | Plexin A3 | GC0XP154458 |
| MIR367 | MicroRNA 367 | GC04M112647 |
| EIF2S1 | Eukaryotic Translation Initiation Factor 2 Subunit Alpha | GC14P067359 |
| STYK1 | Serine/Threonine/Tyrosine Kinase 1 | GC12M013380 |
| ASIP | Agouti Signaling Protein | GC20P034194 |
| IL23R | Interleukin 23 Receptor | GC01P067138 |
| PES1 | Pescadillo Ribosomal Biogenesis Factor 1 | GC22M030576 |
| AKR1C2 | Aldo-Keto Reductase Family 1 Member C2 | GC10M004987 |
| GUSB | Glucuronidase Beta | GC07M065960 |
| IL5 | Interleukin 5 | GC05M132541 |
| PLA2G12B | Phospholipase A2 Group XIIB | GC10M072934 |
| GPSM2 | G Protein Signaling Modulator 2 | GC01P108875 |
| HEPH | Hephaestin | GC0XP066162 |
| POLG | DNA Polymerase Gamma, Catalytic Subunit | GC15M089316 |
| PTPRD | Protein Tyrosine Phosphatase Receptor Type D | GC09M008307 |
| LLGL1 | LLGL Scribble Cell Polarity Complex Component 1 | GC17P018225 |
| AGT | Angiotensinogen | GC01M230702 |
| TCF19 | Transcription Factor 19 | GC06P033377 |
| ESRRG | Estrogen Related Receptor Gamma | GC01M216503 |
| MIR1468 | MicroRNA 1468 | GC0XM063786 |
| SKP1 | S-Phase Kinase Associated Protein 1 | GC05M134148 |
| AIP | Aryl Hydrocarbon Receptor Interacting Protein | GC11P067483 |
| TRPC1 | Transient Receptor Potential Cation Channel Subfamily C Member 1 | GC03P142724 |
| WDR77 | WD Repeat Domain 77 | GC01M111439 |
| TWNK | Twinkle MtDNA Helicase | GC10P100994 |
| FBN1 | Fibrillin 1 | GC15M048408 |
| RPA2 | Replication Protein A2 | GC01M027902 |
| TSPO | Translocator Protein | GC22P043151 |
| SFRP4 | Secreted Frizzled Related Protein 4 | GC07M037912 |
| CCL21 | C-C Motif Chemokine Ligand 21 | GC09M034709 |
| BCAM | Basal Cell Adhesion Molecule (Lutheran Blood Group) | GC19P044810 |
| BRD2 | Bromodomain Containing 2 | GC06P033446 |
| USF2 | Upstream Transcription Factor 2, C-Fos Interacting | GC19P035268 |
| GALNT12 | Polypeptide N-Acetylgalactosaminyltransferase 12 | GC09P098807 |
| RPS27 | Ribosomal Protein S27 | GC01P153991 |
| REV3L | REV3 Like, DNA Directed Polymerase Zeta Catalytic Subunit | GC06M111299 |
| TACC2 | Transforming Acidic Coiled-Coil Containing Protein 2 | GC10P121989 |
| STK3 | Serine/Threonine Kinase 3 | GC08M098372 |
| CXCL13 | C-X-C Motif Chemokine Ligand 13 | GC04P077511 |
| GCK | Glucokinase | GC07M044145 |
| MIR582 | MicroRNA 582 | GC05M059703 |
| CLCN3 | Chloride Voltage-Gated Channel 3 | GC04P169612 |
| PRKCQ | Protein Kinase C Theta | GC10M006393 |
| ILF3 | Interleukin Enhancer Binding Factor 3 | GC19P010625 |
| THBS2 | Thrombospondin 2 | GC06M169215 |
| UGCG | UDP-Glucose Ceramide Glucosyltransferase | GC09P111896 |
| NLRP3 | NLR Family Pyrin Domain Containing 3 | GC01P247415 |
| NAGS | N-Acetylglutamate Synthase | GC17P044004 |
| PYCARD | PYD And CARD Domain Containing | GC16M031201 |
| KLF8 | Kruppel Like Factor 8 | GC0XP056275 |
| SLC5A8 | Solute Carrier Family 5 Member 8 | GC12M101155 |
| PAK2 | P21 (RAC1) Activated Kinase 2 | GC03P196739 |
| NR0B1 | Nuclear Receptor Subfamily 0 Group B Member 1 | GC0XM030304 |
| REN | Renin | GC01M204154 |
| LIPC | Lipase C, Hepatic Type | GC15P058410 |
| MTA2 | Metastasis Associated 1 Family Member 2 | GC11M062594 |
| DGUOK | Deoxyguanosine Kinase | GC02P073926 |
| TMSB10 | Thymosin Beta 10 | GC02P084905 |
| TFCP2 | Transcription Factor CP2 | GC12M051093 |
| PAH | Phenylalanine Hydroxylase | GC12M102836 |
| PHLDA2 | Pleckstrin Homology Like Domain Family A Member 2 | GC11M002928 |
| SPTB | Spectrin Beta, Erythrocytic | GC14M064746 |
| CEP55 | Centrosomal Protein 55 | GC10P093496 |
| NAP1L5 | Nucleosome Assembly Protein 1 Like 5 | GC04M088695 |
| AKAP12 | A-Kinase Anchoring Protein 12 | GC06P151239 |
| MARCHF6 | Membrane Associated Ring-CH-Type Finger 6 | GC05P010356 |
| WWP1 | WW Domain Containing E3 Ubiquitin Protein Ligase 1 | GC08P086342 |
| RERG | RAS Like Estrogen Regulated Growth Inhibitor | GC12M015151 |
| IFT88 | Intraflagellar Transport 88 | GC13P020566 |
| CDC27 | Cell Division Cycle 27 | GC17M047117 |
| SF3B4 | Splicing Factor 3b Subunit 4 | GC01M149923 |
| TUSC1 | Tumor Suppressor Candidate 1 | GC09M025668 |
| DUOX1 | Dual Oxidase 1 | GC15P045129 |
| PSME3 | Proteasome Activator Subunit 3 | GC17P042824 |
| MAP4K4 | Mitogen-Activated Protein Kinase Kinase Kinase Kinase 4 | GC02P101773 |
| RBBP4 | RB Binding Protein 4, Chromatin Remodeling Factor | GC01P032651 |
| BPTF | Bromodomain PHD Finger Transcription Factor | GC17P067825 |
| HSPA1L | Heat Shock Protein Family A (Hsp70) Member 1 Like | GC06M031809 |
| VPS33B | VPS33B Late Endosome And Lysosome Associated | GC15M090998 |
| MIR20B | MicroRNA 20b | GC0XM134245 |
| SOCS2 | Suppressor Of Cytokine Signaling 2 | GC12P093569 |
| HDGFL2 | HDGF Like 2 | GC19P004473 |
| SSB | Small RNA Binding Exonuclease Protection Factor La | GC02P169791 |
| MX1 | MX Dynamin Like GTPase 1 | GC21P041420 |
| APOA4 | Apolipoprotein A4 | GC11M116820 |
| CARD16 | Caspase Recruitment Domain Family Member 16 | GC11M105041 |
| DDX39B | DExD-Box Helicase 39B | GC06M031530 |
| ADAM8 | ADAM Metallopeptidase Domain 8 | GC10M133262 |
| PIAS1 | Protein Inhibitor Of Activated STAT 1 | GC15P068054 |
| MECP2 | Methyl-CpG Binding Protein 2 | GC0XM154021 |
| CHD5 | Chromodomain Helicase DNA Binding Protein 5 | GC01M006103 |
| TUBG1 | Tubulin Gamma 1 | GC17P042609 |
| TBXT | T-Box Transcription Factor T | GC06M166158 |
| ABCC5 | ATP Binding Cassette Subfamily C Member 5 | GC03M183919 |
| SELENOP | Selenoprotein P | GC05M042800 |
| DHX9 | DExH-Box Helicase 9 | GC01P182839 |
| SUMO1 | Small Ubiquitin Like Modifier 1 | GC02M202206 |
| MAP2 | Microtubule Associated Protein 2 | GC02P209424 |
| MT-ND4 | Mitochondrially Encoded NADH:Ubiquinone Oxidoreductase Core Subunit 4 | GCMTP010762 |
| MRC1 | Mannose Receptor C-Type 1 | GC10P017809 |
| FGF21 | Fibroblast Growth Factor 21 | GC19P048764 |
| MIR489 | MicroRNA 489 | GC07M093483 |
| NSD2 | Nuclear Receptor Binding SET Domain Protein 2 | GC04P001872 |
| GNLY | Granulysin | GC02P085685 |
| USP8 | Ubiquitin Specific Peptidase 8 | GC15P050424 |
| KLF10 | Kruppel Like Factor 10 | GC08M102648 |
| NUAK1 | NUAK Family Kinase 1 | GC12M106063 |
| PHF20L1 | PHD Finger Protein 20 Like 1 | GC08P132775 |
| RPS3A | Ribosomal Protein S3A | GC04P151099 |
| GGT2 | Gamma-Glutamyltransferase 2 | GC22M021207 |
| GGTLC3 | Gamma-Glutamyltransferase Light Chain Family Member 3 | GC22M018516 |
| MAPRE1 | Microtubule Associated Protein RP/EB Family Member 1 | GC20P032819 |
| ERP29 | Endoplasmic Reticulum Protein 29 | GC12P112013 |
| HOXA9 | Homeobox A9 | GC07M027162 |
| ACTR3 | Actin Related Protein 3 | GC02P113889 |
| WNT8B | Wnt Family Member 8B | GC10P100463 |
| SRSF2 | Serine And Arginine Rich Splicing Factor 2 | GC17M076734 |
| HINT1 | Histidine Triad Nucleotide Binding Protein 1 | GC05M131159 |
| UGT1A6 | UDP Glucuronosyltransferase Family 1 Member A6 | GC02P233691 |
| ITGB5 | Integrin Subunit Beta 5 | GC03M124761 |
| SULT1A3 | Sulfotransferase Family 1A Member 3 | GC16P030199 |
| KHDRBS1 | KH RNA Binding Domain Containing, Signal Transduction Associated 1 | GC01P031981 |
| NR2C2 | Nuclear Receptor Subfamily 2 Group C Member 2 | GC03P014947 |
| KDM3A | Lysine Demethylase 3A | GC02P086440 |
| CETP | Cholesteryl Ester Transfer Protein | GC16P056961 |
| IL18R1 | Interleukin 18 Receptor 1 | GC02P102311 |
| PREX2 | Phosphatidylinositol-3,4,5-Trisphosphate Dependent Rac Exchange Factor 2 | GC08P067952 |
| MIR454 | MicroRNA 454 | GC17M059137 |
| MIR622 | MicroRNA 622 | GC13P090231 |
| IL22 | Interleukin 22 | GC12M068248 |
| MCM3 | Minichromosome Maintenance Complex Component 3 | GC06M052264 |
| ADORA3 | Adenosine A3 Receptor | GC01M111499 |
| ISG15 | ISG15 Ubiquitin Like Modifier | GC01P001001 |
| FZD10 | Frizzled Class Receptor 10 | GC12P130162 |
| WTAP | WT1 Associated Protein | GC06P159725 |
| WNT9A | Wnt Family Member 9A | GC01M227920 |
| HTRA1 | HtrA Serine Peptidase 1 | GC10P122461 |
| CEACAM4 | CEA Cell Adhesion Molecule 4 | GC19M041767 |
| GCKR | Glucokinase Regulator | GC02P027496 |
| SCO2 | Synthesis Of Cytochrome C Oxidase 2 | GC22M050523 |
| H4C13 | H4 Clustered Histone 13 | GC06M028298 |
| RAD51L3-RFFL | RAD51L3-RFFL Readthrough | GC17M035009 |
| SOX30 | SRY-Box Transcription Factor 30 | GC05M157624 |
| COX10 | Cytochrome C Oxidase Assembly Factor Heme A:Farnesyltransferase COX10 | GC17P014069 |
| PLEK | Pleckstrin | GC02P068365 |
| NPPC | Natriuretic Peptide C | GC02M231921 |
| PTCSC1 | Papillary Thyroid Carcinoma Susceptibility Candidate 1 | GC08P133054 |
| MIR381 | MicroRNA 381 | GC14P104457 |
| POU2F1 | POU Class 2 Homeobox 1 | GC01P167190 |
| TRPV1 | Transient Receptor Potential Cation Channel Subfamily V Member 1 | GC17M003565 |
| RAB27A | RAB27A, Member RAS Oncogene Family | GC15M055202 |
| ENTPD1 | Ectonucleoside Triphosphate Diphosphohydrolase 1 | GC10P095711 |
| UBE2I | Ubiquitin Conjugating Enzyme E2 I | GC16P001312 |
| AZGP1 | Alpha-2-Glycoprotein 1, Zinc-Binding | GC07M099967 |
| GAB2 | GRB2 Associated Binding Protein 2 | GC11M078215 |
| CBX7 | Chromobox 7 | GC22M044552 |
| BACH1 | BTB Domain And CNC Homolog 1 | GC21P029194 |
| NDUFA4L2 | NDUFA4 Mitochondrial Complex Associated Like 2 | GC12M057234 |
| NT5E | 5'-Nucleotidase Ecto | GC06P085449 |
| UGT1A | UDP Glucuronosyltransferase Family 1 Member A Complex Locus | GC02P233586 |
| DUSP10 | Dual Specificity Phosphatase 10 | GC01M221701 |
| CA1 | Carbonic Anhydrase 1 | GC08M085327 |
| NID1 | Nidogen 1 | GC01M235975 |
| SOAT2 | Sterol O-Acyltransferase 2 | GC12P053103 |
| DNM2 | Dynamin 2 | GC19P010718 |
| SRPK1 | SRSF Protein Kinase 1 | GC06M041295 |
| CR1 | Complement C3b/C4b Receptor 1 (Knops Blood Group) | GC01P207496 |
| CS | Citrate Synthase | GC12M056271 |
| TCF21 | Transcription Factor 21 | GC06P133889 |
| SKIL | SKI Like Proto-Oncogene | GC03P170357 |
| UBE3C | Ubiquitin Protein Ligase E3C | GC07P157138 |
| AIM2 | Absent In Melanoma 2 | GC01M159062 |
| PCBP2 | Poly(RC) Binding Protein 2 | GC12P053452 |
| HCK | HCK Proto-Oncogene, Src Family Tyrosine Kinase | GC20P032052 |
| RELN | Reelin | GC07M103471 |
| MACROH2A1 | MacroH2A.1 Histone | GC05M135334 |
| SCAP | SREBF Chaperone | GC03M047413 |
| DDC | Dopa Decarboxylase | GC07M050458 |
| CRAT | Carnitine O-Acetyltransferase | GC09M129094 |
| CSF3R | Colony Stimulating Factor 3 Receptor | GC01M036466 |
| AKAP13 | A-Kinase Anchoring Protein 13 | GC15P085381 |
| USP14 | Ubiquitin Specific Peptidase 14 | GC18P000158 |
| ATF5 | Activating Transcription Factor 5 | GC19P049931 |
| MAGI1 | Membrane Associated Guanylate Kinase, WW And PDZ Domain Containing 1 | GC03M065330 |
| KTN1 | Kinectin 1 | GC14P055559 |
| SEPTIN4 | Septin 4 | GC17M058522 |
| CYP4B1 | Cytochrome P450 Family 4 Subfamily B Member 1 | GC01P046757 |
| PCDH9 | Protocadherin 9 | GC13M066302 |
| EFCAB11 | EF-Hand Calcium Binding Domain 11 | GC14M093556 |
| C2 | Complement C2 | GC06P031897 |
| TCF3 | Transcription Factor 3 | GC19M001609 |
| ESRRB | Estrogen Related Receptor Beta | GC14P076310 |
| KCNN4 | Potassium Calcium-Activated Channel Subfamily N Member 4 | GC19M043767 |
| NFKB2 | Nuclear Factor Kappa B Subunit 2 | GC10P102394 |
| RNF2 | Ring Finger Protein 2 | GC01P185045 |
| TRIM11 | Tripartite Motif Containing 11 | GC01M228393 |
| CSH1 | Chorionic Somatomammotropin Hormone 1 | GC17M063894 |
| ALDH9A1 | Aldehyde Dehydrogenase 9 Family Member A1 | GC01M165665 |
| IL37 | Interleukin 37 | GC02P114902 |
| UFL1 | UFM1 Specific Ligase 1 | GC06P096521 |
| PRPF19 | Pre-MRNA Processing Factor 19 | GC11M060890 |
| AKIP1 | A-Kinase Interacting Protein 1 | GC11P008911 |
| LPXN | Leupaxin | GC11M059798 |
| PRPF8 | Pre-MRNA Processing Factor 8 | GC17M001650 |
| FZD6 | Frizzled Class Receptor 6 | GC08P103298 |
| KLF11 | Kruppel Like Factor 11 | GC02P010044 |
| MAFB | MAF BZIP Transcription Factor B | GC20M040685 |
| PPP1R13B | Protein Phosphatase 1 Regulatory Subunit 13B | GC14M103733 |
| CLDN9 | Claudin 9 | GC16P003012 |
| FZD3 | Frizzled Class Receptor 3 | GC08P028494 |
| GSK3A | Glycogen Synthase Kinase 3 Alpha | GC19M042230 |
| ARHGDIA | Rho GDP Dissociation Inhibitor Alpha | GC17M081867 |
| STAT6 | Signal Transducer And Activator Of Transcription 6 | GC12M057095 |
| IRF9 | Interferon Regulatory Factor 9 | GC14P024161 |
| CCT8 | Chaperonin Containing TCP1 Subunit 8 | GC21M029055 |
| TPMT | Thiopurine S-Methyltransferase | GC06M018128 |
| PAK5 | P21 (RAC1) Activated Kinase 5 | GC20M009538 |
| GGPS1 | Geranylgeranyl Diphosphate Synthase 1 | GC01P235327 |
| KRT37 | Keratin 37 | GC17M041422 |
| VWCE | Von Willebrand Factor C And EGF Domains | GC11M061258 |
| YBX3 | Y-Box Binding Protein 3 | GC12M013382 |
| POLD2 | DNA Polymerase Delta 2, Accessory Subunit | GC07M044114 |
| PARD3 | Par-3 Family Cell Polarity Regulator | GC10M034110 |
| DOT1L | DOT1 Like Histone Lysine Methyltransferase | GC19P002164 |
| ABCD4 | ATP Binding Cassette Subfamily D Member 4 | GC14M074285 |
| GGT3P | Gamma-Glutamyltransferase 3 Pseudogene | GC22M018773 |
| FDFT1 | Farnesyl-Diphosphate Farnesyltransferase 1 | GC08P011795 |
| RECQL | RecQ Like Helicase | GC12M021468 |
| MAL2 | Mal, T Cell Differentiation Protein 2 (Gene/Pseudogene) | GC08P119165 |
| PGRMC1 | Progesterone Receptor Membrane Component 1 | GC0XP119236 |
| FGL2 | Fibrinogen Like 2 | GC07M077193 |
| PCDH10 | Protocadherin 10 | GC04P133149 |
| SFRP5 | Secreted Frizzled Related Protein 5 | GC10M097766 |
| FXN | Frataxin | GC09P069035 |
| HSPB8 | Heat Shock Protein Family B (Small) Member 8 | GC12P119178 |
| MORC2 | MORC Family CW-Type Zinc Finger 2 | GC22M030925 |
| HOXD10 | Homeobox D10 | GC02P176108 |
| XAGE1A | X Antigen Family Member 1A | GC0XP052496 |
| TRIM21 | Tripartite Motif Containing 21 | GC11M004384 |
| CENPH | Centromere Protein H | GC05P069189 |
| SQLE | Squalene Epoxidase | GC08P124998 |
| LMNB1 | Lamin B1 | GC05P126776 |
| FBXO5 | F-Box Protein 5 | GC06M152970 |
| PNLIPRP3 | Pancreatic Lipase Related Protein 3 | GC10P116427 |
| MED19 | Mediator Complex Subunit 19 | GC11M057703 |
| AIF1 | Allograft Inflammatory Factor 1 | GC06P033396 |
| LOC105274310 | H19/IGF2 Enhancer Region | GC11U902975 |
| PIK3C2A | Phosphatidylinositol-4-Phosphate 3-Kinase Catalytic Subunit Type 2 Alpha | GC11M017165 |
| ACLY | ATP Citrate Lyase | GC17M041866 |
| NEO1 | Neogenin 1 | GC15P073051 |
| MAGEB2 | MAGE Family Member B2 | GC0XP030215 |
| FZD9 | Frizzled Class Receptor 9 | GC07P073433 |
| FOSL2 | FOS Like 2, AP-1 Transcription Factor Subunit | GC02P028392 |
| HNRNPH1 | Heterogeneous Nuclear Ribonucleoprotein H1 | GC05M179614 |
| CD74 | CD74 Molecule | GC05M150378 |
| P2RY2 | Purinergic Receptor P2Y2 | GC11P073217 |
| CD83 | CD83 Molecule | GC06P014117 |
| POR | Cytochrome P450 Oxidoreductase | GC07P075899 |
| PLK4 | Polo Like Kinase 4 | GC04P127880 |
| MAP2K3 | Mitogen-Activated Protein Kinase Kinase 3 | GC17P026156 |
| MBOAT7 | Membrane Bound O-Acyltransferase Domain Containing 7 | GC19M054173 |
| LOC110386951 | CYP19A1 Promoter II/1.3 | GC15U902412 |
| CDH15 | Cadherin 15 | GC16P089171 |
| TRIM44 | Tripartite Motif Containing 44 | GC11P035684 |
| SERPINA6 | Serpin Family A Member 6 | GC14M094305 |
| G6PC3 | Glucose-6-Phosphatase Catalytic Subunit 3 | GC17P044070 |
| EIF4G1 | Eukaryotic Translation Initiation Factor 4 Gamma 1 | GC03P184314 |
| HSPB6 | Heat Shock Protein Family B (Small) Member 6 | GC19M041410 |
| UBAP2L | Ubiquitin Associated Protein 2 Like | GC01P154219 |
| IL1R1 | Interleukin 1 Receptor Type 1 | GC02P102136 |
| CEBPD | CCAAT Enhancer Binding Protein Delta | GC08M047759 |
| IL27 | Interleukin 27 | GC16M028511 |
| CPD | Carboxypeptidase D | GC17P030378 |
| EPHB3 | EPH Receptor B3 | GC03P184561 |
| LINC00668 | Long Intergenic Non-Protein Coding RNA 668 | GC18M006922 |
| MEF2A | Myocyte Enhancer Factor 2A | GC15P099565 |
| CNR1 | Cannabinoid Receptor 1 | GC06M088139 |
| SAA4 | Serum Amyloid A4, Constitutive | GC11M018234 |
| PRKAR2B | Protein Kinase CAMP-Dependent Type II Regulatory Subunit Beta | GC07P107044 |
| NELFE | Negative Elongation Factor Complex Member E | GC06M031952 |
| COA8 | Cytochrome C Oxidase Assembly Factor 8 | GC14P104334 |
| CCT2 | Chaperonin Containing TCP1 Subunit 2 | GC12P069585 |
| LRG1 | Leucine Rich Alpha-2-Glycoprotein 1 | GC19M004538 |
| LOC110283621 | SPP1 5' Regulatory Region | GC04U902747 |
| SOS2 | SOS Ras/Rho Guanine Nucleotide Exchange Factor 2 | GC14M050117 |
| GPC1 | Glypican 1 | GC02P240435 |
| ABO | ABO, Alpha 1-3-N-Acetylgalactosaminyltransferase And Alpha 1-3-Galactosyltransferase | GC09M133250 |
| RLN2 | Relaxin 2 | GC09M005306 |
| TRPV4 | Transient Receptor Potential Cation Channel Subfamily V Member 4 | GC12M109783 |
| MGLL | Monoglyceride Lipase | GC03M127689 |
| OAT | Ornithine Aminotransferase | GC10M124397 |
| NLK | Nemo Like Kinase | GC17P028042 |
| CST3 | Cystatin C | GC20M023608 |
| ELL2 | Elongation Factor For RNA Polymerase II 2 | GC05M095885 |
| CRHR2 | Corticotropin Releasing Hormone Receptor 2 | GC07M030651 |
| IRS4 | Insulin Receptor Substrate 4 | GC0XM108720 |
| THORLNC | Testis Associated Oncogenic LncRNA | GC02M118133 |
| YWHAQ | Tyrosine 3-Monooxygenase/Tryptophan 5-Monooxygenase Activation Protein Theta | GC02M009583 |
| H4C14 | H4 Clustered Histone 14 | GC01P149832 |
| HDLBP | High Density Lipoprotein Binding Protein | GC02M241227 |
| CUL7 | Cullin 7 | GC06M043037 |
| ZHX2 | Zinc Fingers And Homeoboxes 2 | GC08P122781 |
| APOBEC3A | Apolipoprotein B MRNA Editing Enzyme Catalytic Subunit 3A | GC22P038952 |
| ZNRD1 | Zinc Ribbon Domain Containing 1 | GC06P033214 |
| GC | GC Vitamin D Binding Protein | GC04M071741 |
| BCL9 | BCL9 Transcription Coactivator | GC01P147541 |
| NOB1 | NIN1 (RPN12) Binding Protein 1 Homolog | GC16M069747 |
| KIF4A | Kinesin Family Member 4A | GC0XP070290 |
| KLK11 | Kallikrein Related Peptidase 11 | GC19M051023 |
| NHLRC2 | NHL Repeat Containing 2 | GC10P113854 |
| NPPA | Natriuretic Peptide A | GC01M011846 |
| LAMP2 | Lysosomal Associated Membrane Protein 2 | GC0XM120426 |
| ANKRD11 | Ankyrin Repeat Domain 11 | GC16M089267 |
| TXNRD3 | Thioredoxin Reductase 3 | GC03M126607 |
| RLIM | Ring Finger Protein, LIM Domain Interacting | GC0XM074583 |
| RICTOR | RPTOR Independent Companion Of MTOR Complex 2 | GC05M038939 |
| THPO | Thrombopoietin | GC03M184371 |
| RPA3 | Replication Protein A3 | GC07M007637 |
| H4C11 | H4 Clustered Histone 11 | GC06P028137 |
| H4C12 | H4 Clustered Histone 12 | GC06M028295 |
| CXCL11 | C-X-C Motif Chemokine Ligand 11 | GC04M076033 |
| ISL1 | ISL LIM Homeobox 1 | GC05P051383 |
| KCNK12 | Potassium Two Pore Domain Channel Subfamily K Member 12 | GC02M047516 |
| ERCC8 | ERCC Excision Repair 8, CSA Ubiquitin Ligase Complex Subunit | GC05M060873 |
| RINT1 | RAD50 Interactor 1 | GC07P105532 |
| SIRT2 | Sirtuin 2 | GC19M038878 |
| GRIK1 | Glutamate Ionotropic Receptor Kainate Type Subunit 1 | GC21M029536 |
| MIR181D | MicroRNA 181d | GC19P013874 |
| ILF2 | Interleukin Enhancer Binding Factor 2 | GC01M153661 |
| SPON2 | Spondin 2 | GC04M001166 |
| MAP4K3 | Mitogen-Activated Protein Kinase Kinase Kinase Kinase 3 | GC02M039249 |
| FOXO4 | Forkhead Box O4 | GC0XP071096 |
| KAT8 | Lysine Acetyltransferase 8 | GC16P031411 |
| USP4 | Ubiquitin Specific Peptidase 4 | GC03M049277 |
| CRK | CRK Proto-Oncogene, Adaptor Protein | GC17M001420 |
| SLC22A17 | Solute Carrier Family 22 Member 17 | GC14M023346 |
| SOCS6 | Suppressor Of Cytokine Signaling 6 | GC18P070288 |
| MIR638 | MicroRNA 638 | GC19P010719 |
| MIR493 | MicroRNA 493 | GC14P104470 |
| MTHFD1 | Methylenetetrahydrofolate Dehydrogenase, Cyclohydrolase And Formyltetrahydrofolate Synthetase 1 | GC14P064388 |
| PRRX1 | Paired Related Homeobox 1 | GC01P170662 |
| ACVR2A | Activin A Receptor Type 2A | GC02P147844 |
| LIMS1 | LIM Zinc Finger Domain Containing 1 | GC02P108609 |
| RSAD2 | Radical S-Adenosyl Methionine Domain Containing 2 | GC02P006865 |
| RAB1A | RAB1A, Member RAS Oncogene Family | GC02M065048 |
| PTPRT | Protein Tyrosine Phosphatase Receptor Type T | GC20M042072 |
| MIR422A | MicroRNA 422a | GC15M063870 |
| ABHD16A | Abhydrolase Domain Containing 16A | GC06M032373 |
| GALNT3 | Polypeptide N-Acetylgalactosaminyltransferase 3 | GC02M165747 |
| ACACA | Acetyl-CoA Carboxylase Alpha | GC17M037084 |
| ATG12 | Autophagy Related 12 | GC05M115828 |
| MIR421 | MicroRNA 421 | GC0XM074218 |
| ARHGAP35 | Rho GTPase Activating Protein 35 | GC19P046860 |
| HNRNPA1 | Heterogeneous Nuclear Ribonucleoprotein A1 | GC12P054280 |
| VILL | Villin Like | GC03P037989 |
| DMTF1 | Cyclin D Binding Myb Like Transcription Factor 1 | GC07P087155 |
| E2F7 | E2F Transcription Factor 7 | GC12M077021 |
| CYB5A | Cytochrome B5 Type A | GC18M074250 |
| KIF14 | Kinesin Family Member 14 | GC01M200521 |
| SGO1 | Shugoshin 1 | GC03M020159 |
| TXNDC5 | Thioredoxin Domain Containing 5 | GC06M007893 |
| SOX5 | SRY-Box Transcription Factor 5 | GC12M023529 |
| VPS52 | VPS52 Subunit Of GARP Complex | GC06M033251 |
| ZBTB12 | Zinc Finger And BTB Domain Containing 12 | GC06M031899 |
| PROK1 | Prokineticin 1 | GC01P110451 |
| LPA | Lipoprotein(A) | GC06M160531 |
| TKTL1 | Transketolase Like 1 | GC0XP154295 |
| OCLN | Occludin | GC05P069492 |
| DHRS9 | Dehydrogenase/Reductase 9 | GC02P169064 |
| CNN1 | Calponin 1 | GC19P011539 |
| CEMIP | Cell Migration Inducing Hyaluronidase 1 | GC15P080779 |
| RPN2 | Ribophorin II | GC20P037178 |
| PABPC1 | Poly(A) Binding Protein Cytoplasmic 1 | GC08M100685 |
| BOK | BCL2 Family Apoptosis Regulator BOK | GC02P241558 |
| LAMP1 | Lysosomal Associated Membrane Protein 1 | GC13P113297 |
| SERBP1 | SERPINE1 MRNA Binding Protein 1 | GC01M067407 |
| NID2 | Nidogen 2 | GC14M052004 |
| ADAM28 | ADAM Metallopeptidase Domain 28 | GC08P024294 |
| MUS81 | MUS81 Structure-Specific Endonuclease Subunit | GC11P065878 |
| TPT1 | Tumor Protein, Translationally-Controlled 1 | GC13M045333 |
| SUMO1P3 | SUMO1 Pseudogene 3 | GC01P160317 |
| CAP1 | Cyclase Associated Actin Cytoskeleton Regulatory Protein 1 | GC01P040041 |
| MYO6 | Myosin VI | GC06P075749 |
| SMC1A | Structural Maintenance Of Chromosomes 1A | GC0XM053374 |
| DND1 | DND MicroRNA-Mediated Repression Inhibitor 1 | GC05M140670 |
| KIF20A | Kinesin Family Member 20A | GC05P138189 |
| MIR384 | MicroRNA 384 | GC0XM076919 |
| DACH1 | Dachshund Family Transcription Factor 1 | GC13M071437 |
| SLC30A1 | Solute Carrier Family 30 Member 1 | GC01M211571 |
| TGFB1I1 | Transforming Growth Factor Beta 1 Induced Transcript 1 | GC16P031479 |
| WSPAR | WNT Signaling Pathway Activating Non-Coding RNA | GC05P133914 |
| MAPT | Microtubule Associated Protein Tau | GC17P045894 |
| HBS1L | HBS1 Like Translational GTPase | GC06M134960 |
| CT83 | Cancer/Testis Antigen 83 | GC0XM116461 |
| H3C1 | H3 Clustered Histone 1 | GC06P026032 |
| PDSS2 | Decaprenyl Diphosphate Synthase Subunit 2 | GC06M107152 |
| GRAMD4 | GRAM Domain Containing 4 | GC22P046576 |
| PDP1 | Pyruvate Dehyrogenase Phosphatase Catalytic Subunit 1 | GC08P093857 |
| APRT | Adenine Phosphoribosyltransferase | GC16M088810 |
| SPINK7 | Serine Peptidase Inhibitor Kazal Type 7 | GC05P148312 |
| PSMB4 | Proteasome 20S Subunit Beta 4 | GC01P151372 |
| TUFM | Tu Translation Elongation Factor, Mitochondrial | GC16M028853 |
| BABAM2 | BRISC And BRCA1 A Complex Member 2 | GC02P027889 |
| ATRX | ATRX Chromatin Remodeler | GC0XM077504 |
| TTF1 | Transcription Termination Factor 1 | GC09M132375 |
| UTS2 | Urotensin 2 | GC01M007904 |
| ARHGAP1 | Rho GTPase Activating Protein 1 | GC11M059676 |
| PIM3 | Pim-3 Proto-Oncogene, Serine/Threonine Kinase | GC22P049960 |
| SOX11 | SRY-Box Transcription Factor 11 | GC02P005704 |
| MIR613 | MicroRNA 613 | GC12P012766 |
| LAP3 | Leucine Aminopeptidase 3 | GC04P017578 |
| C5AR1 | Complement C5a Receptor 1 | GC19P047290 |
| MYCBP | MYC Binding Protein | GC01M038862 |
| SOX7 | SRY-Box Transcription Factor 7 | GC08M010723 |
| MIR940 | MicroRNA 940 | GC16P002271 |
| TM4SF1 | Transmembrane 4 L Six Family Member 1 | GC03M149370 |
| SOX17 | SRY-Box Transcription Factor 17 | GC08P054457 |
| ARF1 | ADP Ribosylation Factor 1 | GC01P228082 |
| ERRFI1 | ERBB Receptor Feedback Inhibitor 1 | GC01M008004 |
| FNDC3B | Fibronectin Type III Domain Containing 3B | GC03P172039 |
| DCST1-AS1 | DCST1 Antisense RNA 1 | GC01M155046 |
| USP10 | Ubiquitin Specific Peptidase 10 | GC16P084734 |
| LTBP2 | Latent Transforming Growth Factor Beta Binding Protein 2 | GC14M074498 |
| TRRAP | Transformation/Transcription Domain Associated Protein | GC07P098877 |
| CHL1 | Cell Adhesion Molecule L1 Like | GC03P000213 |
| HILPDA | Hypoxia Inducible Lipid Droplet Associated | GC07P129281 |
| KANK1 | KN Motif And Ankyrin Repeat Domains 1 | GC09P000474 |
| STMN2 | Stathmin 2 | GC08P079610 |
| PLA2G6 | Phospholipase A2 Group VI | GC22M045345 |
| MAPK11 | Mitogen-Activated Protein Kinase 11 | GC22M050263 |
| NEDD8 | NEDD8 Ubiquitin Like Modifier | GC14M024216 |
| C1RL | Complement C1r Subcomponent Like | GC12M007114 |
| PNPLA2 | Patatin Like Phospholipase Domain Containing 2 | GC11P000884 |
| SMARCAD1 | SWI/SNF-Related, Matrix-Associated Actin-Dependent Regulator Of Chromatin, Subfamily A, Containing DEAD/H Box 1 | GC04P094207 |
| APOBEC1 | Apolipoprotein B MRNA Editing Enzyme Catalytic Subunit 1 | GC12M007649 |
| ICAM2 | Intercellular Adhesion Molecule 2 | GC17M064002 |
| TBP | TATA-Box Binding Protein | GC06P170554 |
| PIAS3 | Protein Inhibitor Of Activated STAT 3 | GC01M145848 |
| ABCC6 | ATP Binding Cassette Subfamily C Member 6 | GC16M016148 |
| PBK | PDZ Binding Kinase | GC08M027809 |
| ABCA1 | ATP Binding Cassette Subfamily A Member 1 | GC09M104781 |
| CDK8 | Cyclin Dependent Kinase 8 | GC13P026254 |
| COMMD1 | Copper Metabolism Domain Containing 1 | GC02P061888 |
| SP100 | SP100 Nuclear Antigen | GC02P230415 |
| SMTN | Smoothelin | GC22P031066 |
| MIR92B | MicroRNA 92b | GC01P155195 |
| ADAMTS1 | ADAM Metallopeptidase With Thrombospondin Type 1 Motif 1 | GC21M026835 |
| SIRT5 | Sirtuin 5 | GC06P013574 |
| MIR1297 | MicroRNA 1297 | GC13M054311 |
| SMURF1 | SMAD Specific E3 Ubiquitin Protein Ligase 1 | GC07M099027 |
| FSTL1 | Follistatin Like 1 | GC03M120392 |
| CERS2 | Ceramide Synthase 2 | GC01M150934 |
| ST7 | Suppression Of Tumorigenicity 7 | GC07P117013 |
| UCHL5 | Ubiquitin C-Terminal Hydrolase L5 | GC01M193012 |
| GNA12 | G Protein Subunit Alpha 12 | GC07M002728 |
| FOXD3 | Forkhead Box D3 | GC01P063323 |
| DTL | Denticleless E3 Ubiquitin Protein Ligase Homolog | GC01P212035 |
| CBFB | Core-Binding Factor Subunit Beta | GC16P067063 |
| GJC1 | Gap Junction Protein Gamma 1 | GC17M044800 |
| PDHA1 | Pyruvate Dehydrogenase E1 Subunit Alpha 1 | GC0XP019343 |
| CDC37 | Cell Division Cycle 37 | GC19M010391 |
| CLDN2 | Claudin 2 | GC0XP106900 |
| QKI | QKI, KH Domain Containing RNA Binding | GC06P163414 |
| ANTXR1 | ANTXR Cell Adhesion Molecule 1 | GC02P068977 |
| FBXO30 | F-Box Protein 30 | GC06M145795 |
| RUVBL1 | RuvB Like AAA ATPase 1 | GC03M128064 |
| MIR7-3 | MicroRNA 7-3 | GC19P004770 |
| TBK1 | TANK Binding Kinase 1 | GC12P064451 |
| NUP210 | Nucleoporin 210 | GC03M015402 |
| PGAM1 | Phosphoglycerate Mutase 1 | GC10P097426 |
| EFNB1 | Ephrin B1 | GC0XP068828 |
| EYA4 | EYA Transcriptional Coactivator And Phosphatase 4 | GC06P133240 |
| CRYAA | Crystallin Alpha A | GC21P043169 |
| PHB2 | Prohibitin 2 | GC12M006965 |
| GRB10 | Growth Factor Receptor Bound Protein 10 | GC07M050590 |
| ERO1A | Endoplasmic Reticulum Oxidoreductase 1 Alpha | GC14M052640 |
| BCAT1 | Branched Chain Amino Acid Transaminase 1 | GC12M024732 |
| FOSB | FosB Proto-Oncogene, AP-1 Transcription Factor Subunit | GC19P045467 |
| ANXA2P2 | Annexin A2 Pseudogene 2 | GC09P034631 |
| PER3 | Period Circadian Regulator 3 | GC01P007785 |
| SNHG8 | Small Nucleolar RNA Host Gene 8 | GC04P118278 |
| CCAR1 | Cell Division Cycle And Apoptosis Regulator 1 | GC10P068721 |
| DSC1 | Desmocollin 1 | GC18M031129 |
| BCL2L12 | BCL2 Like 12 | GC19P049665 |
| WASF3 | WASP Family Member 3 | GC13P026557 |
| TMEM30B | Transmembrane Protein 30B | GC14M061277 |
| INS-IGF2 | INS-IGF2 Readthrough | GC11M002177 |
| CLEC4M | C-Type Lectin Domain Family 4 Member M | GC19P007763 |
| ADH4 | Alcohol Dehydrogenase 4 (Class II), Pi Polypeptide | GC04M099123 |
| CAMK2G | Calcium/Calmodulin Dependent Protein Kinase II Gamma | GC10M073812 |
| UGT8 | UDP Glycosyltransferase 8 | GC04P114598 |
| CTSS | Cathepsin S | GC01M150730 |
| GEMIN4 | Gem Nuclear Organelle Associated Protein 4 | GC17M000744 |
| TLR6 | Toll Like Receptor 6 | GC04M038828 |
| PPM1A | Protein Phosphatase, Mg2+/Mn2+ Dependent 1A | GC14P060245 |
| PEG3 | Paternally Expressed 3 | GC19M056810 |
| GPLD1 | Glycosylphosphatidylinositol Specific Phospholipase D1 | GC06M024425 |
| GATA5 | GATA Binding Protein 5 | GC20M062464 |
| MMP21 | Matrix Metallopeptidase 21 | GC10M125766 |
| TLL1 | Tolloid Like 1 | GC04P165873 |
| USP9X | Ubiquitin Specific Peptidase 9 X-Linked | GC0XP041085 |
| CAPG | Capping Actin Protein, Gelsolin Like | GC02M085394 |
| IL17F | Interleukin 17F | GC06M052209 |
| HNRNPU | Heterogeneous Nuclear Ribonucleoprotein U | GC01M244850 |
| MAEL | Maelstrom Spermatogenic Transposon Silencer | GC01P166958 |
| SERPINA4 | Serpin Family A Member 4 | GC14P094561 |
| FKBP4 | FKBP Prolyl Isomerase 4 | GC12P002795 |
| FLVCR1 | FLVCR Heme Transporter 1 | GC01P212858 |
| NDRG3 | NDRG Family Member 3 | GC20M036651 |
| SDC4 | Syndecan 4 | GC20M045325 |
| CNR2 | Cannabinoid Receptor 2 | GC01M023870 |
| MIR539 | MicroRNA 539 | GC14P104474 |
| PTAFR | Platelet Activating Factor Receptor | GC01M028147 |
| MIR302C | MicroRNA 302c | GC04M112675 |
| G3BP1 | G3BP Stress Granule Assembly Factor 1 | GC05P151771 |
| CSNK2A2 | Casein Kinase 2 Alpha 2 | GC16M058157 |
| ALKBH1 | AlkB Homolog 1, Histone H2A Dioxygenase | GC14M077672 |
| SLC12A2 | Solute Carrier Family 12 Member 2 | GC05P128083 |
| MINCR | MYC-Induced Long Non-Coding RNA | GC08M143281 |
| H3-3A | H3.3 Histone A | GC01P226062 |
| ZFP57 | ZFP57 Zinc Finger Protein | GC06M029672 |
| SAFB | Scaffold Attachment Factor B | GC19P005623 |
| GTF2B | General Transcription Factor IIB | GC01M088853 |
| CYP3A7 | Cytochrome P450 Family 3 Subfamily A Member 7 | GC07M099705 |
| EPRS1 | Glutamyl-Prolyl-TRNA Synthetase 1 | GC01M219969 |
| MPL | MPL Proto-Oncogene, Thrombopoietin Receptor | GC01P043337 |
| MIR7-2 | MicroRNA 7-2 | GC15P088611 |
| SYCP1 | Synaptonemal Complex Protein 1 | GC01P114854 |
| SOX12 | SRY-Box Transcription Factor 12 | GC20P000325 |
| GART | Phosphoribosylglycinamide Formyltransferase, Phosphoribosylglycinamide Synthetase, Phosphoribosylaminoimidazole Synthetase | GC21M033503 |
| KPNB1 | Karyopherin Subunit Beta 1 | GC17P047649 |
| CDK12 | Cyclin Dependent Kinase 12 | GC17P039461 |
| AGO1 | Argonaute RISC Component 1 | GC01P035869 |
| SLC25A11 | Solute Carrier Family 25 Member 11 | GC17M004937 |
| PDLIM5 | PDZ And LIM Domain 5 | GC04P094451 |
| DGAT1 | Diacylglycerol O-Acyltransferase 1 | GC08M144316 |
| MIR190A | MicroRNA 190a | GC15P071994 |
| STK10 | Serine/Threonine Kinase 10 | GC05M172042 |
| CTBP2 | C-Terminal Binding Protein 2 | GC10M124984 |
| DMPK | DM1 Protein Kinase | GC19M045769 |
| RPS6KA6 | Ribosomal Protein S6 Kinase A6 | GC0XM084058 |
| CDK9 | Cyclin Dependent Kinase 9 | GC09P127810 |
| PHGDH | Phosphoglycerate Dehydrogenase | GC01P119660 |
| SIAH2 | Siah E3 Ubiquitin Protein Ligase 2 | GC03M150741 |
| PMVK | Phosphomevalonate Kinase | GC01M154924 |
| BAMBI | BMP And Activin Membrane Bound Inhibitor | GC10P028685 |
| DUOX2 | Dual Oxidase 2 | GC15M045092 |
| ADAMTS5 | ADAM Metallopeptidase With Thrombospondin Type 1 Motif 5 | GC21M026918 |
| NAP1L1 | Nucleosome Assembly Protein 1 Like 1 | GC12M076036 |
| MIR508 | MicroRNA 508 | GC0XM147236 |
| CAMK4 | Calcium/Calmodulin Dependent Protein Kinase IV | GC05P111223 |
| HOXA11 | Homeobox A11 | GC07M027223 |
| ESD | Esterase D | GC13M046771 |
| CDK10 | Cyclin Dependent Kinase 10 | GC16P089680 |
| B3GALT5 | Beta-1,3-Galactosyltransferase 5 | GC21P039556 |
| FOXF2 | Forkhead Box F2 | GC06P001390 |
| SOD3 | Superoxide Dismutase 3 | GC04P024798 |
| OLA1 | Obg Like ATPase 1 | GC02M174072 |
| ACVR1 | Activin A Receptor Type 1 | GC02M157736 |
| XPO5 | Exportin 5 | GC06M043522 |
| FLNC | Filamin C | GC07P128830 |
| SGK1 | Serum/Glucocorticoid Regulated Kinase 1 | GC06M134169 |
| TFAP4 | Transcription Factor AP-4 | GC16M004247 |
| BIRC6 | Baculoviral IAP Repeat Containing 6 | GC02P032357 |
| CBR3 | Carbonyl Reductase 3 | GC21P036134 |
| TCP1 | T-Complex 1 | GC06M159778 |
| TNFRSF14 | TNF Receptor Superfamily Member 14 | GC01P002555 |
| EIF4A1 | Eukaryotic Translation Initiation Factor 4A1 | GC17P007572 |
| MIR19B1 | MicroRNA 19b-1 | GC13P091428 |
| GPR101 | G Protein-Coupled Receptor 101 | GC0XM137030 |
| CYP2B6 | Cytochrome P450 Family 2 Subfamily B Member 6 | GC19P040991 |
| RACGAP1 | Rac GTPase Activating Protein 1 | GC12M049978 |
| TERF2IP | TERF2 Interacting Protein | GC16P075647 |
| HBG2 | Hemoglobin Subunit Gamma 2 | GC11M005349 |
| UNC119 | Unc-119 Lipid Binding Chaperone | GC17M028546 |
| NUP133 | Nucleoporin 133 | GC01M229441 |
| NUP85 | Nucleoporin 85 | GC17P075205 |
| WASHC4 | WASH Complex Subunit 4 | GC12P105108 |
| UBE2Q1 | Ubiquitin Conjugating Enzyme E2 Q1 | GC01M154521 |
| CREB3L4 | CAMP Responsive Element Binding Protein 3 Like 4 | GC01P153967 |
| HCG11 | HLA Complex Group 11 | GC06P028026 |
| OPRM1 | Opioid Receptor Mu 1 | GC06P154075 |
| SUV39H1 | Suppressor Of Variegation 3-9 Homolog 1 | GC0XP048697 |
| CAPN5 | Calpain 5 | GC11P077066 |
| WDR20 | WD Repeat Domain 20 | GC14P104309 |
| GAGE1 | G Antigen 1 | GC0XP049590 |
| MUCL1 | Mucin Like 1 | GC12P054830 |
| OGDH | Oxoglutarate Dehydrogenase | GC07P044606 |
| HSD17B13 | Hydroxysteroid 17-Beta Dehydrogenase 13 | GC04M087303 |
| MZF1 | Myeloid Zinc Finger 1 | GC19M058577 |
| APLN | Apelin | GC0XM129645 |
| CDO1 | Cysteine Dioxygenase Type 1 | GC05M115804 |
| PCSK1 | Proprotein Convertase Subtilisin/Kexin Type 1 | GC05M096391 |
| GRK2 | G Protein-Coupled Receptor Kinase 2 | GC11P067266 |
| CREB3 | CAMP Responsive Element Binding Protein 3 | GC09P035722 |
| JDP2 | Jun Dimerization Protein 2 | GC14P075427 |
| FGF18 | Fibroblast Growth Factor 18 | GC05P171419 |
| PTOV1 | PTOV1 Extended AT-Hook Containing Adaptor Protein | GC19P049850 |
| BLVRB | Biliverdin Reductase B | GC19M040447 |
| MBD6 | Methyl-CpG Binding Domain Protein 6 | GC12P057520 |
| PRIMPOL | Primase And DNA Directed Polymerase | GC04P184649 |
| ERFE | Erythroferrone | GC02P238159 |
| ZIC1 | Zic Family Member 1 | GC03P147393 |
| TPTE2 | Transmembrane Phosphoinositide 3-Phosphatase And Tensin Homolog 2 | GC13M019422 |
| OSGIN1 | Oxidative Stress Induced Growth Inhibitor 1 | GC16P083982 |
| FOXN3 | Forkhead Box N3 | GC14M093525 |
| NR4A2 | Nuclear Receptor Subfamily 4 Group A Member 2 | GC02M156324 |
| TNFSF9 | TNF Superfamily Member 9 | GC19P006531 |
| NEK7 | NIMA Related Kinase 7 | GC01P198156 |
| PDK1 | Pyruvate Dehydrogenase Kinase 1 | GC02P172555 |
| RETREG1 | Reticulophagy Regulator 1 | GC05M016472 |
| HNRNPF | Heterogeneous Nuclear Ribonucleoprotein F | GC10M043385 |
| EGR2 | Early Growth Response 2 | GC10M062811 |
| CXCL17 | C-X-C Motif Chemokine Ligand 17 | GC19M042428 |
| MIB1 | Mindbomb E3 Ubiquitin Protein Ligase 1 | GC18P021704 |
| H1-3 | H1.3 Linker Histone, Cluster Member | GC06M027051 |
| BCL6 | BCL6 Transcription Repressor | GC03M187721 |
| DIO3 | Iodothyronine Deiodinase 3 | GC14P104335 |
| GZMA | Granzyme A | GC05P055102 |
| MTA3 | Metastasis Associated 1 Family Member 3 | GC02P042494 |
| FHL2 | Four And A Half LIM Domains 2 | GC02M105343 |
| NDUFAF2 | NADH:Ubiquinone Oxidoreductase Complex Assembly Factor 2 | GC05P060945 |
| SCARA5 | Scavenger Receptor Class A Member 5 | GC08M027869 |
| CASP14 | Caspase 14 | GC19P015049 |
| PITX1 | Paired Like Homeodomain 1 | GC05M135027 |
| S1PR1 | Sphingosine-1-Phosphate Receptor 1 | GC01P101236 |
| GRK6 | G Protein-Coupled Receptor Kinase 6 | GC05P177403 |
| CALD1 | Caldesmon 1 | GC07P134744 |
| PKD1 | Polycystin 1, Transient Receptor Potential Channel Interacting | GC16M002170 |
| DDX53 | DEAD-Box Helicase 53 | GC0XP022999 |
| NEFH | Neurofilament Heavy | GC22P029480 |
| POU5F1P4 | POU Class 5 Homeobox 1 Pseudogene 4 | GC01P155445 |
| FOXK1 | Forkhead Box K1 | GC07P004644 |
| PYGM | Glycogen Phosphorylase, Muscle Associated | GC11M064746 |
| CASK | Calcium/Calmodulin Dependent Serine Protein Kinase | GC0XM041514 |
| HNRNPDL | Heterogeneous Nuclear Ribonucleoprotein D Like | GC04M082422 |
| BCL11A | BAF Chromatin Remodeling Complex Subunit BCL11A | GC02M060451 |
| PTPRA | Protein Tyrosine Phosphatase Receptor Type A | GC20P002864 |
| PPFIBP2 | PPFIA Binding Protein 2 | GC11P007491 |
| TRIM65 | Tripartite Motif Containing 65 | GC17M075880 |
| CCL19 | C-C Motif Chemokine Ligand 19 | GC09M034692 |
| SOX6 | SRY-Box Transcription Factor 6 | GC11M015949 |
| PTGES2 | Prostaglandin E Synthase 2 | GC09M128120 |
| GP5 | Glycoprotein V Platelet | GC03M194395 |
| C10orf99 | Chromosome 10 Open Reading Frame 99 | GC10P084173 |
| KHSRP | KH-Type Splicing Regulatory Protein | GC19M006413 |
| TNFRSF18 | TNF Receptor Superfamily Member 18 | GC01M001203 |
| RDH11 | Retinol Dehydrogenase 11 | GC14M067676 |
| LTBR | Lymphotoxin Beta Receptor | GC12P006375 |
| SLC5A1 | Solute Carrier Family 5 Member 1 | GC22P032043 |
| BLK | BLK Proto-Oncogene, Src Family Tyrosine Kinase | GC08P011486 |
| CCT4 | Chaperonin Containing TCP1 Subunit 4 | GC02M061868 |
| PPIG | Peptidylprolyl Isomerase G | GC02P169584 |
| ATG3 | Autophagy Related 3 | GC03M112532 |
| FRS2 | Fibroblast Growth Factor Receptor Substrate 2 | GC12P069471 |
| ASL | Argininosuccinate Lyase | GC07P066075 |
| CDKL2 | Cyclin Dependent Kinase Like 2 | GC04M075576 |
| ZYX | Zyxin | GC07P144749 |
| CELF1 | CUGBP Elav-Like Family Member 1 | GC11M059698 |
| SKI | SKI Proto-Oncogene | GC01P002228 |
| E4F1 | E4F Transcription Factor 1 | GC16P002223 |
| PYGO2 | Pygopus Family PHD Finger 2 | GC01M154957 |
| MIR1269A | MicroRNA 1269a | GC04P066276 |
| MTRR | 5-Methyltetrahydrofolate-Homocysteine Methyltransferase Reductase | GC05P007851 |
| PLAAT4 | Phospholipase A And Acyltransferase 4 | GC11P063538 |
| FAT4 | FAT Atypical Cadherin 4 | GC04P125315 |
| SPRED2 | Sprouty Related EVH1 Domain Containing 2 | GC02M065307 |
| HDAC8 | Histone Deacetylase 8 | GC0XM072329 |
| CCT6A | Chaperonin Containing TCP1 Subunit 6A | GC07P056051 |
| CD19 | CD19 Molecule | GC16P028943 |
| CYP20A1 | Cytochrome P450 Family 20 Subfamily A Member 1 | GC02P203238 |
| NODAL | Nodal Growth Differentiation Factor | GC10M070431 |
| APLNR | Apelin Receptor | GC11M057233 |
| UIMC1 | Ubiquitin Interaction Motif Containing 1 | GC05M176905 |
| NAT10 | N-Acetyltransferase 10 | GC11P034105 |
| ATP8B2 | ATPase Phospholipid Transporting 8B2 | GC01P154325 |
| ATP8B4 | ATPase Phospholipid Transporting 8B4 (Putative) | GC15M049858 |
| TMEM30CP | Transmembrane Protein 30C, Pseudogene | GC03P100186 |
| RASSF10 | Ras Association Domain Family Member 10 | GC11P012990 |
| MIR766 | MicroRNA 766 | GC0XM119646 |
| TFPI | Tissue Factor Pathway Inhibitor | GC02M187464 |
| TP53INP1 | Tumor Protein P53 Inducible Nuclear Protein 1 | GC08M094925 |
| APOC2 | Apolipoprotein C2 | GC19P044945 |
| KLKB1 | Kallikrein B1 | GC04P186208 |
| LIME1 | Lck Interacting Transmembrane Adaptor 1 | GC20P063736 |
| UCN | Urocortin | GC02M027308 |
| HRH2 | Histamine Receptor H2 | GC05P175659 |
| ITCH | Itchy E3 Ubiquitin Protein Ligase | GC20P034363 |
| SGCE | Sarcoglycan Epsilon | GC07M094585 |
| ITPA | Inosine Triphosphatase | GC20P003189 |
| TMC2 | Transmembrane Channel Like 2 | GC20P002536 |
| HLA-F | Major Histocompatibility Complex, Class I, F | GC06P033159 |
| CENPE | Centromere Protein E | GC04M103105 |
| SETD7 | SET Domain Containing 7, Histone Lysine Methyltransferase | GC04M139495 |
| SERPINA5 | Serpin Family A Member 5 | GC14P094563 |
| KDM5A | Lysine Demethylase 5A | GC12M000280 |
| MIR1246 | MicroRNA 1246 | GC02M176600 |
| EAF2 | ELL Associated Factor 2 | GC03P121835 |
| PLAC1 | Placenta Enriched 1 | GC0XM134565 |
| HIBCH | 3-Hydroxyisobutyryl-CoA Hydrolase | GC02M190189 |
| NEFL | Neurofilament Light | GC08M024950 |
| DCD | Dermcidin | GC12M054644 |
| HAX1 | HCLS1 Associated Protein X-1 | GC01P154273 |
| GSDME | Gasdermin E | GC07M024699 |
| EFNA3 | Ephrin A3 | GC01P155078 |
| ELOVL6 | ELOVL Fatty Acid Elongase 6 | GC04M110045 |
| TRIM35 | Tripartite Motif Containing 35 | GC08M027284 |
| SRY | Sex Determining Region Y | GC0YM002698 |
| QSOX1 | Quiescin Sulfhydryl Oxidase 1 | GC01P180154 |
| MIR133A2 | MicroRNA 133a-2 | GC20P062877 |
| TARBP2 | TARBP2 Subunit Of RISC Loading Complex | GC12P053499 |
| PTBP1 | Polypyrimidine Tract Binding Protein 1 | GC19P000797 |
| DKK4 | Dickkopf WNT Signaling Pathway Inhibitor 4 | GC08M042373 |
| NT5C | 5', 3'-Nucleotidase, Cytosolic | GC17M075130 |
| ARFGAP3 | ADP Ribosylation Factor GTPase Activating Protein 3 | GC22M042796 |
| C5 | Complement C5 | GC09M120952 |
| KRT6B | Keratin 6B | GC12M052446 |
| MAGED4B | MAGE Family Member D4B | GC0XM052061 |
| FUT7 | Fucosyltransferase 7 | GC09M137030 |
| HNF4G | Hepatocyte Nuclear Factor 4 Gamma | GC08P075407 |
| ABCB5 | ATP Binding Cassette Subfamily B Member 5 | GC07P020615 |
| NUP160 | Nucleoporin 160 | GC11M059719 |
| PPP1R15A | Protein Phosphatase 1 Regulatory Subunit 15A | GC19P048872 |
| ING2 | Inhibitor Of Growth Family Member 2 | GC04P183504 |
| CD226 | CD226 Molecule | GC18M069831 |
| PCDH17 | Protocadherin 17 | GC13P057630 |
| PTPRU | Protein Tyrosine Phosphatase Receptor Type U | GC01P029236 |
| CYP2J2 | Cytochrome P450 Family 2 Subfamily J Member 2 | GC01M059893 |
| MIR1307 | MicroRNA 1307 | GC10M103394 |
| TCTE3 | T-Complex-Associated-Testis-Expressed 3 | GC06M169739 |
| ZNF384 | Zinc Finger Protein 384 | GC12M006668 |
| ANAPC1 | Anaphase Promoting Complex Subunit 1 | GC02M111611 |
| FGA | Fibrinogen Alpha Chain | GC04M154583 |
| CAMK1 | Calcium/Calmodulin Dependent Protein Kinase I | GC03M009774 |
| HM13 | Histocompatibility Minor 13 | GC20P031514 |
| FKBP8 | FKBP Prolyl Isomerase 8 | GC19M018503 |
| CORO1A | Coronin 1A | GC16P030194 |
| BAZ2A | Bromodomain Adjacent To Zinc Finger Domain 2A | GC12M056595 |
| PCCA | Propionyl-CoA Carboxylase Subunit Alpha | GC13P100089 |
| SAR1A | Secretion Associated Ras Related GTPase 1A | GC10M070147 |
| PRRT2 | Proline Rich Transmembrane Protein 2 | GC16P029811 |
| PSMC1 | Proteasome 26S Subunit, ATPase 1 | GC14P090256 |
| DPP9 | Dipeptidyl Peptidase 9 | GC19M004675 |
| OGT | O-Linked N-Acetylglucosamine (GlcNAc) Transferase | GC0XP071534 |
| MICA-AS1 | MICA Antisense RNA 1 | GC06M031478 |
| FNDC5 | Fibronectin Type III Domain Containing 5 | GC01M032864 |
| CDC5L | Cell Division Cycle 5 Like | GC06P044387 |
| VMP1 | Vacuole Membrane Protein 1 | GC17P059707 |
| ADI1 | Acireductone Dioxygenase 1 | GC02M003501 |
| CYP26B1 | Cytochrome P450 Family 26 Subfamily B Member 1 | GC02M072129 |
| ARG2 | Arginase 2 | GC14P067619 |
| BTBD7 | BTB Domain Containing 7 | GC14M093237 |
| ATP5F1A | ATP Synthase F1 Subunit Alpha | GC18M046081 |
| BAIAP2L1 | BAR/IMD Domain Containing Adaptor Protein 2 Like 1 | GC07M098292 |
| AICDA | Activation Induced Cytidine Deaminase | GC12M008602 |
| HYOU1 | Hypoxia Up-Regulated 1 | GC11M119045 |
| OCIAD2 | OCIA Domain Containing 2 | GC04M048887 |
| COX6B1 | Cytochrome C Oxidase Subunit 6B1 | GC19P037718 |
| BCL6B | BCL6B Transcription Repressor | GC17P007023 |
| LRIG3 | Leucine Rich Repeats And Immunoglobulin Like Domains 3 | GC12M058872 |
| MYOD1 | Myogenic Differentiation 1 | GC11P017741 |
| ID3 | Inhibitor Of DNA Binding 3, HLH Protein | GC01M023557 |
| UBB | Ubiquitin B | GC17P016380 |
| MBD3 | Methyl-CpG Binding Domain Protein 3 | GC19M001690 |
| RPTOR | Regulatory Associated Protein Of MTOR Complex 1 | GC17P080544 |
| TRIP11 | Thyroid Hormone Receptor Interactor 11 | GC14M091965 |
| MIG7 | Mig-7 | GC01U901611 |
| TUBA1C | Tubulin Alpha 1c | GC12P049188 |
| F2RL2 | Coagulation Factor II Thrombin Receptor Like 2 | GC05M076615 |
| FBLIM1 | Filamin Binding LIM Protein 1 | GC01P015756 |
| XAGE1B | X Antigen Family Member 1B | GC0XM052513 |
| NCK1 | NCK Adaptor Protein 1 | GC03P136862 |
| NCOA6 | Nuclear Receptor Coactivator 6 | GC20M034696 |
| BCL2L10 | BCL2 Like 10 | GC15M059761 |
| TNKS2 | Tankyrase 2 | GC10P091798 |
| ACIN1 | Apoptotic Chromatin Condensation Inducer 1 | GC14M023058 |
| EDN3 | Endothelin 3 | GC20P059300 |
| SLC34A2 | Solute Carrier Family 34 Member 2 | GC04P025657 |
| MAOA | Monoamine Oxidase A | GC0XP043654 |
| RIPK4 | Receptor Interacting Serine/Threonine Kinase 4 | GC21M041739 |
| PRKACG | Protein Kinase CAMP-Activated Catalytic Subunit Gamma | GC09M069013 |
| UNC13D | Unc-13 Homolog D | GC17M075827 |
| NUP107 | Nucleoporin 107 | GC12P068686 |
| NUP43 | Nucleoporin 43 | GC06M149724 |
| AHCTF1 | AT-Hook Containing Transcription Factor 1 | GC01M246840 |
| TUBB4B | Tubulin Beta 4B Class IVb | GC09P137241 |
| PRKD3 | Protein Kinase D3 | GC02M037251 |
| PCAT14 | Prostate Cancer Associated Transcript 14 | GC22P023846 |
| DDAH2 | Dimethylarginine Dimethylaminohydrolase 2 | GC06M031727 |
| NDC80 | NDC80 Kinetochore Complex Component | GC18P002571 |
| TIGAR | TP53 Induced Glycolysis Regulatory Phosphatase | GC12P006580 |
| MIR300 | MicroRNA 300 | GC14P104441 |
| PSAP | Prosaposin | GC10M071816 |
| GPS2 | G Protein Pathway Suppressor 2 | GC17M007311 |
| ACP5 | Acid Phosphatase 5, Tartrate Resistant | GC19M011574 |
| EVA1A | Eva-1 Homolog A, Regulator Of Programmed Cell Death | GC02M075469 |
| SRSF3 | Serine And Arginine Rich Splicing Factor 3 | GC06P046053 |
| ORM2 | Orosomucoid 2 | GC09P114329 |
| TSTA3 | Tissue Specific Transplantation Antigen P35B | GC08M143612 |
| NFATC2 | Nuclear Factor Of Activated T Cells 2 | GC20M051386 |
| MAP3K3 | Mitogen-Activated Protein Kinase Kinase Kinase 3 | GC17P063622 |
| RIT1 | Ras Like Without CAAX 1 | GC01M155897 |
| NDN | Necdin, MAGE Family Member | GC15M023686 |
| NUDT1 | Nudix Hydrolase 1 | GC07P002242 |
| ZKSCAN1 | Zinc Finger With KRAB And SCAN Domains 1 | GC07P100015 |
| HSPE1 | Heat Shock Protein Family E (Hsp10) Member 1 | GC02P197501 |
| SGTA | Small Glutamine Rich Tetratricopeptide Repeat Containing Alpha | GC19M002754 |
| RPH3AL | Rabphilin 3A Like (Without C2 Domains) | GC17M000212 |
| DLX4 | Distal-Less Homeobox 4 | GC17P049968 |
| MT1F | Metallothionein 1F | GC16P056657 |
| ALYREF | Aly/REF Export Factor | GC17M081887 |
| MT1X | Metallothionein 1X | GC16P056698 |
| IGBP1 | Immunoglobulin Binding Protein 1 | GC0XP070133 |
| ENDOG | Endonuclease G | GC09P128818 |
| TSTD1 | Thiosulfate Sulfurtransferase Like Domain Containing 1 | GC01M161037 |
| IER3 | Immediate Early Response 3 | GC06M030743 |
| GABPA | GA Binding Protein Transcription Factor Subunit Alpha | GC21P025734 |
| CCL17 | C-C Motif Chemokine Ligand 17 | GC16P057399 |
| HNRNPL | Heterogeneous Nuclear Ribonucleoprotein L | GC19M038836 |
| MAGEA2 | MAGE Family Member A2 | GC0XM152749 |
| PDHX | Pyruvate Dehydrogenase Complex Component X | GC11P034894 |
| TRERNA1 | Translation Regulatory Long Non-Coding RNA 1 | GC20M050040 |
| MIR545 | MicroRNA 545 | GC0XM074287 |
| ZNF382 | Zinc Finger Protein 382 | GC19P037751 |
| RORA | RAR Related Orphan Receptor A | GC15M060488 |
| BTG3 | BTG Anti-Proliferation Factor 3 | GC21M017594 |
| DNM3 | Dynamin 3 | GC01P171810 |
| RAB11B | RAB11B, Member RAS Oncogene Family | GC19P008393 |
| ANP32A | Acidic Nuclear Phosphoprotein 32 Family Member A | GC15M068778 |
| MAGED1 | MAGE Family Member D1 | GC0XP051803 |
| TNIP1 | TNFAIP3 Interacting Protein 1 | GC05M151029 |
| EIF5A | Eukaryotic Translation Initiation Factor 5A | GC17P007306 |
| PHLPP1 | PH Domain And Leucine Rich Repeat Protein Phosphatase 1 | GC18P062715 |
| SUN2 | Sad1 And UNC84 Domain Containing 2 | GC22M044549 |
| CLPS | Colipase | GC06M041294 |
| EPM2AIP1 | EPM2A Interacting Protein 1 | GC03M036986 |
| GALR2 | Galanin Receptor 2 | GC17P076075 |
| CCR3 | C-C Motif Chemokine Receptor 3 | GC03P046227 |
| EHD2 | EH Domain Containing 2 | GC19P047713 |
| HOXC6 | Homeobox C6 | GC12P053990 |
| CAMP | Cathelicidin Antimicrobial Peptide | GC03P048250 |
| KRT9 | Keratin 9 | GC17M041565 |
| MIR657 | MicroRNA 657 | GC17M081125 |
| MIR216B | MicroRNA 216b | GC02M056000 |
| MUC15 | Mucin 15, Cell Surface Associated | GC11M026537 |
| PNN | Pinin, Desmosome Associated Protein | GC14P039175 |
| COMP | Cartilage Oligomeric Matrix Protein | GC19M018783 |
| PLOD2 | Procollagen-Lysine,2-Oxoglutarate 5-Dioxygenase 2 | GC03M146069 |
| PAM16 | Presequence Translocase Associated Motor 16 | GC16M004332 |
| HOXC8 | Homeobox C8 | GC12P054166 |
| TICAM1 | Toll Like Receptor Adaptor Molecule 1 | GC19M004815 |
| MCM6 | Minichromosome Maintenance Complex Component 6 | GC02M135839 |
| NKD1 | NKD Inhibitor Of WNT Signaling Pathway 1 | GC16P050548 |
| SPRY1 | Sprouty RTK Signaling Antagonist 1 | GC04P123396 |
| MIR194-2 | MicroRNA 194-2 | GC11M065003 |
| IKZF3 | IKAROS Family Zinc Finger 3 | GC17M039759 |
| LAMA2 | Laminin Subunit Alpha 2 | GC06P128863 |
| FUT1 | Fucosyltransferase 1 (H Blood Group) | GC19M048748 |
| CENPA | Centromere Protein A | GC02P026750 |
| ARL6IP5 | ADP Ribosylation Factor Like GTPase 6 Interacting Protein 5 | GC03P069084 |
| IL11RA | Interleukin 11 Receptor Subunit Alpha | GC09P034650 |
| KLB | Klotho Beta | GC04P039408 |
| KRT81 | Keratin 81 | GC12M052286 |
| ATXN2 | Ataxin 2 | GC12M111443 |
| STRAP | Serine/Threonine Kinase Receptor Associated Protein | GC12P015882 |
| SPOCK1 | SPARC (Osteonectin), Cwcv And Kazal Like Domains Proteoglycan 1 | GC05M136975 |
| GPSM3 | G Protein Signaling Modulator 3 | GC06M032406 |
| PNPT1 | Polyribonucleotide Nucleotidyltransferase 1 | GC02M055634 |
| TIMM8A | Translocase Of Inner Mitochondrial Membrane 8A | GC0XM101345 |
| GET1 | Guided Entry Of Tail-Anchored Proteins Factor 1 | GC21P039377 |
| HOTAIRM1 | HOXA Transcript Antisense RNA, Myeloid-Specific 1 | GC07P027095 |
| ASH2L | ASH2 Like, Histone Lysine Methyltransferase Complex Subunit | GC08P038104 |
| RGS17 | Regulator Of G Protein Signaling 17 | GC06M153004 |
| ISX | Intestine Specific Homeobox | GC22P035066 |
| SPI1 | Spi-1 Proto-Oncogene | GC11M059694 |
| H1-4 | H1.4 Linker Histone, Cluster Member | GC06P028369 |
| MIR570 | MicroRNA 570 | GC03P195741 |
| DDX39A | DExD-Box Helicase 39A | GC19M014408 |
| CHRNA7 | Cholinergic Receptor Nicotinic Alpha 7 Subunit | GC15P031923 |
| AZU1 | Azurocidin 1 | GC19P000825 |
| MIR589 | MicroRNA 589 | GC07M005495 |
| MSBP1 | Minisatellite Binding Protein 1 | GC00U990213 |
| CBX5 | Chromobox 5 | GC12M054230 |
| CFHR1 | Complement Factor H Related 1 | GC01P196788 |
| BCL2A1 | BCL2 Related Protein A1 | GC15M079961 |
| RBBP6 | RB Binding Protein 6, Ubiquitin Ligase | GC16P024537 |
| NOG | Noggin | GC17P056593 |
| PTP4A2 | Protein Tyrosine Phosphatase 4A2 | GC01M031907 |
| TLK1 | Tousled Like Kinase 1 | GC02M170990 |
| MIR448 | MicroRNA 448 | GC0XP114823 |
| IFITM3 | Interferon Induced Transmembrane Protein 3 | GC11M000319 |
| SH2D1A | SH2 Domain Containing 1A | GC0XP124227 |
| RPS6KA1 | Ribosomal Protein S6 Kinase A1 | GC01P026540 |
| RPLP2 | Ribosomal Protein Lateral Stalk Subunit P2 | GC11P000875 |
| CHD4 | Chromodomain Helicase DNA Binding Protein 4 | GC12M006570 |
| MUC13 | Mucin 13, Cell Surface Associated | GC03M124905 |
| ATP9A | ATPase Phospholipid Transporting 9A (Putative) | GC20M051596 |
| ATP10D | ATPase Phospholipid Transporting 10D (Putative) | GC04P047487 |
| LIVAR | Liver Cell Viability Associated LncRNA | GC18M070336 |
| IRF2 | Interferon Regulatory Factor 2 | GC04M184387 |
| ENTPD2 | Ectonucleoside Triphosphate Diphosphohydrolase 2 | GC09M137048 |
| RPL18A | Ribosomal Protein L18a | GC19P022238 |
| COPG2 | COPI Coat Complex Subunit Gamma 2 | GC07M130506 |
| CBX8 | Chromobox 8 | GC17M079794 |
| ARRB1 | Arrestin Beta 1 | GC11M075261 |
| MCPH1 | Microcephalin 1 | GC08P006406 |
| CUX1 | Cut Like Homeobox 1 | GC07P101815 |
| MEF2C | Myocyte Enhancer Factor 2C | GC05M088718 |
| GOT1 | Glutamic-Oxaloacetic Transaminase 1 | GC10M099396 |
| HSPH1 | Heat Shock Protein Family H (Hsp110) Member 1 | GC13M031134 |
| WFDC1 | WAP Four-Disulfide Core Domain 1 | GC16P084328 |
| P4HA2 | Prolyl 4-Hydroxylase Subunit Alpha 2 | GC05M132191 |
| CCT5 | Chaperonin Containing TCP1 Subunit 5 | GC05P010236 |
| PSMD2 | Proteasome 26S Subunit, Non-ATPase 2 | GC03P184298 |
| CKS1BP7 | CDC28 Protein Kinase Regulatory Subunit 1B Pseudogene 7 | GC08M080644 |
| SETD6 | SET Domain Containing 6, Protein Lysine Methyltransferase | GC16P058514 |
| CDK3 | Cyclin Dependent Kinase 3 | GC17P076006 |
| MACROD1 | Mono-ADP Ribosylhydrolase 1 | GC11M063998 |
| ALDH1L1 | Aldehyde Dehydrogenase 1 Family Member L1 | GC03M126103 |
| SRGN | Serglycin | GC10P069088 |
| ACSL5 | Acyl-CoA Synthetase Long Chain Family Member 5 | GC10P112374 |
| PIWIL4 | Piwi Like RNA-Mediated Gene Silencing 4 | GC11P094543 |
| DRG2 | Developmentally Regulated GTP Binding Protein 2 | GC17P018088 |
| GALNT14 | Polypeptide N-Acetylgalactosaminyltransferase 14 | GC02M030910 |
| HOXD3 | Homeobox D3 | GC02P176136 |
| PPP1R12A | Protein Phosphatase 1 Regulatory Subunit 12A | GC12M079773 |
| LOC111832671 | Albumin (ALB) 5' Regulatory Region | GC04U902769 |
| LCP1 | Lymphocyte Cytosolic Protein 1 | GC13M046132 |
| KIF20B | Kinesin Family Member 20B | GC10P089701 |
| HNRNPC | Heterogeneous Nuclear Ribonucleoprotein C | GC14M021210 |
| MAP3K6 | Mitogen-Activated Protein Kinase Kinase Kinase 6 | GC01M027365 |
| SLC6A2 | Solute Carrier Family 6 Member 2 | GC16P055656 |
| PTS | 6-Pyruvoyltetrahydropterin Synthase | GC11P112226 |
| H4C8 | H4 Clustered Histone 8 | GC06M027067 |
| NCAPG | Non-SMC Condensin I Complex Subunit G | GC04P017812 |
| MFF | Mitochondrial Fission Factor | GC02P227325 |
| ARHGDIB | Rho GDP Dissociation Inhibitor Beta | GC12M014942 |
| NEK3 | NIMA Related Kinase 3 | GC13M052132 |
| CRISP3 | Cysteine Rich Secretory Protein 3 | GC06M049727 |
| NUF2 | NUF2 Component Of NDC80 Kinetochore Complex | GC01P163266 |
| MIR612 | MicroRNA 612 | GC11P065619 |
| GSS | Glutathione Synthetase | GC20M034928 |
| IRAK4 | Interleukin 1 Receptor Associated Kinase 4 | GC12P043758 |
| MRTFA | Myocardin Related Transcription Factor A | GC22M044778 |
| VASH2 | Vasohibin 2 | GC01P212935 |
| CTSH | Cathepsin H | GC15M078925 |
| STOML2 | Stomatin Like 2 | GC09M035099 |
| NR6A1 | Nuclear Receptor Subfamily 6 Group A Member 1 | GC09M124517 |
| PRKAR1B | Protein Kinase CAMP-Dependent Type I Regulatory Subunit Beta | GC07M000549 |
| SNRPN | Small Nuclear Ribonucleoprotein Polypeptide N | GC15P024823 |
| EIF2B2 | Eukaryotic Translation Initiation Factor 2B Subunit Beta | GC14P075002 |
| LRBA | LPS Responsive Beige-Like Anchor Protein | GC04M150264 |
| NUP37 | Nucleoporin 37 | GC12M102073 |
| RAB3IL1 | RAB3A Interacting Protein Like 1 | GC11M061897 |
| ARFGAP2 | ADP Ribosylation Factor GTPase Activating Protein 2 | GC11M059684 |
| ANKRD49 | Ankyrin Repeat Domain 49 | GC11P094493 |
| CAB39L | Calcium Binding Protein 39 Like | GC13M049308 |
| ORMDL1 | ORMDL Sphingolipid Biosynthesis Regulator 1 | GC02M189770 |
| MAS1L | MAS1 Proto-Oncogene Like, G Protein-Coupled Receptor | GC06M029519 |
| FAM50B | Family With Sequence Similarity 50 Member B | GC06P003849 |
| MT-TF | Mitochondrially Encoded TRNA-Phe (UUU/C) | GCMTP000580 |
| MT-TH | Mitochondrially Encoded TRNA-His (CAU/C) | GCMTP012140 |
| MAGI2 | Membrane Associated Guanylate Kinase, WW And PDZ Domain Containing 2 | GC07M078017 |
| CXXC5 | CXXC Finger Protein 5 | GC05P139647 |
| PDCD5 | Programmed Cell Death 5 | GC19P032581 |
| TLR8 | Toll Like Receptor 8 | GC0XP012924 |
| C1GALT1 | Core 1 Synthase, Glycoprotein-N-Acetylgalactosamine 3-Beta-Galactosyltransferase 1 | GC07P007156 |
| VPS53 | VPS53 Subunit Of GARP Complex | GC17M000508 |
| MTHFD1L | Methylenetetrahydrofolate Dehydrogenase (NADP+ Dependent) 1 Like | GC06P150865 |
| UAP1 | UDP-N-Acetylglucosamine Pyrophosphorylase 1 | GC01P162561 |
| LRAT | Lecithin Retinol Acyltransferase | GC04P154626 |
| FSHB | Follicle Stimulating Hormone Subunit Beta | GC11P030210 |
| RASSF2 | Ras Association Domain Family Member 2 | GC20M004780 |
| OPTN | Optineurin | GC10P013141 |
| SPNS1 | Sphingolipid Transporter 1 (Putative) | GC16P028974 |
| E2F8 | E2F Transcription Factor 8 | GC11M019203 |
| TAB3 | TGF-Beta Activated Kinase 1 (MAP3K7) Binding Protein 3 | GC0XM030845 |
| RPL11 | Ribosomal Protein L11 | GC01P023691 |
| AVPR1A | Arginine Vasopressin Receptor 1A | GC12M063142 |
| PHF10 | PHD Finger Protein 10 | GC06M169679 |
| TANC1 | Tetratricopeptide Repeat, Ankyrin Repeat And Coiled-Coil Containing 1 | GC02P158968 |
| CADM2 | Cell Adhesion Molecule 2 | GC03P085008 |
| GGCX | Gamma-Glutamyl Carboxylase | GC02M085544 |
| PDCD6 | Programmed Cell Death 6 | GC05P000272 |
| CD5L | CD5 Molecule Like | GC01M157800 |
| KIF7 | Kinesin Family Member 7 | GC15M089608 |
| MIR484 | MicroRNA 484 | GC16P015660 |
| H1-1 | H1.1 Linker Histone, Cluster Member | GC06M026018 |
| B4GALT1 | Beta-1,4-Galactosyltransferase 1 | GC09M033100 |
| ATP1A1 | ATPase Na+/K+ Transporting Subunit Alpha 1 | GC01P116372 |
| PKHD1 | PKHD1 Ciliary IPT Domain Containing Fibrocystin/Polyductin | GC06M051588 |
| RGS5 | Regulator Of G Protein Signaling 5 | GC01M163111 |
| SLC8A1 | Solute Carrier Family 8 Member A1 | GC02M040078 |
| SUZ12 | SUZ12 Polycomb Repressive Complex 2 Subunit | GC17P031937 |
| LNCNEF | LncRNA Neighboring Enhancer Of FOXA2 | GC20M022588 |
| H4C3 | H4 Clustered Histone 3 | GC06P026112 |
| MAGEA12 | MAGE Family Member A12 | GC0XP152733 |
| JAZF1 | JAZF Zinc Finger 1 | GC07M027830 |
| HIF3A | Hypoxia Inducible Factor 3 Subunit Alpha | GC19P046297 |
| NFAT5 | Nuclear Factor Of Activated T Cells 5 | GC16P069565 |
| CAPZA1 | Capping Actin Protein Of Muscle Z-Line Subunit Alpha 1 | GC01P112619 |
| NACA | Nascent Polypeptide Associated Complex Subunit Alpha | GC12M056712 |
| DFFB | DNA Fragmentation Factor Subunit Beta | GC01P003797 |
| AQP8 | Aquaporin 8 | GC16P026273 |
| NHEJ1 | Non-Homologous End Joining Factor 1 | GC02M219075 |
| DHCR24 | 24-Dehydrocholesterol Reductase | GC01M054849 |
| NAP1L4 | Nucleosome Assembly Protein 1 Like 4 | GC11M002944 |
| FADS2 | Fatty Acid Desaturase 2 | GC11P061792 |
| PTPRF | Protein Tyrosine Phosphatase Receptor Type F | GC01P043527 |
| SUOX | Sulfite Oxidase | GC12P055997 |
| RCAN1 | Regulator Of Calcineurin 1 | GC21M034513 |
| MIR505 | MicroRNA 505 | GC0XM139924 |
| PIWIL2 | Piwi Like RNA-Mediated Gene Silencing 2 | GC08P022275 |
| MIR135A2 | MicroRNA 135a-2 | GC12P097563 |
| YWHAG | Tyrosine 3-Monooxygenase/Tryptophan 5-Monooxygenase Activation Protein Gamma | GC07M076327 |
| PON2 | Paraoxonase 2 | GC07M095404 |
| DMD | Dystrophin | GC0XM031047 |
| CTPS1 | CTP Synthase 1 | GC01P040979 |
| PCDH20 | Protocadherin 20 | GC13M061409 |
| SOX18 | SRY-Box Transcription Factor 18 | GC20M064047 |
| GCNT2 | Glucosaminyl (N-Acetyl) Transferase 2 (I Blood Group) | GC06P010492 |
| P2RX4 | Purinergic Receptor P2X 4 | GC12P122632 |
| ACSS1 | Acyl-CoA Synthetase Short Chain Family Member 1 | GC20M024986 |
| STK17B | Serine/Threonine Kinase 17b | GC02M196133 |
| ATG4B | Autophagy Related 4B Cysteine Peptidase | GC02P241637 |
| PADI4 | Peptidyl Arginine Deiminase 4 | GC01P017308 |
| CSN2 | Casein Beta | GC04M069955 |
| VANGL1 | VANGL Planar Cell Polarity Protein 1 | GC01P115641 |
| TGFBRAP1 | Transforming Growth Factor Beta Receptor Associated Protein 1 | GC02M105250 |
| ANXA8 | Annexin A8 | GC10M047460 |
| VSIR | V-Set Immunoregulatory Receptor | GC10M071748 |
| MCM3AP | Minichromosome Maintenance Complex Component 3 Associated Protein | GC21M046235 |
| ARMC8 | Armadillo Repeat Containing 8 | GC03P138187 |
| TRPC6 | Transient Receptor Potential Cation Channel Subfamily C Member 6 | GC11M101356 |
| TNFAIP8 | TNF Alpha Induced Protein 8 | GC05P119268 |
| SLC39A4 | Solute Carrier Family 39 Member 4 | GC08M144409 |
| PYM1 | PYM Homolog 1, Exon Junction Complex Associated Factor | GC12M055902 |
| KIR3DS1 | Killer Cell Immunoglobulin Like Receptor, Three Ig Domains And Short Cytoplasmic Tail 1 | GC19MR00058 |
| MIR504 | MicroRNA 504 | GC0XM138667 |
| SHOX2 | Short Stature Homeobox 2 | GC03M158095 |
| BCLAF1 | BCL2 Associated Transcription Factor 1 | GC06M136256 |
| PKP2 | Plakophilin 2 | GC12M032790 |
| MARK3 | Microtubule Affinity Regulating Kinase 3 | GC14P103385 |
| PIEZO1 | Piezo Type Mechanosensitive Ion Channel Component 1 | GC16M088715 |
| HLF | HLF Transcription Factor, PAR BZIP Family Member | GC17P055264 |
| ADAMTS14 | ADAM Metallopeptidase With Thrombospondin Type 1 Motif 14 | GC10P070672 |
| CCL14 | C-C Motif Chemokine Ligand 14 | GC17M036293 |
| TNXB | Tenascin XB | GC06M032383 |
| NKX6-1 | NK6 Homeobox 1 | GC04M084491 |
| GRHL2 | Grainyhead Like Transcription Factor 2 | GC08P101492 |
| PRKAR2A | Protein Kinase CAMP-Dependent Type II Regulatory Subunit Alpha | GC03M048744 |
| CMTM3 | CKLF Like MARVEL Transmembrane Domain Containing 3 | GC16P066609 |
| SETD1A | SET Domain Containing 1A, Histone Lysine Methyltransferase | GC16P030948 |
| CCT7 | Chaperonin Containing TCP1 Subunit 7 | GC02P073233 |
| MTF1 | Metal Regulatory Transcription Factor 1 | GC01M037810 |
| CHSY1 | Chondroitin Sulfate Synthase 1 | GC15M101175 |
| MIR655 | MicroRNA 655 | GC14P104478 |
| CSN1S1 | Casein Alpha S1 | GC04P069932 |
| HUS1 | HUS1 Checkpoint Clamp Component | GC07M047970 |
| MAP2K6 | Mitogen-Activated Protein Kinase Kinase 6 | GC17P069414 |
| POLR2L | RNA Polymerase II Subunit L | GC11M000829 |
| LOC113687175 | Sharpr-MPRA Regulatory Region 4647 | GC07U904880 |
| GSTA2 | Glutathione S-Transferase Alpha 2 | GC06M052750 |
| PPP1R9B | Protein Phosphatase 1 Regulatory Subunit 9B | GC17M050133 |
| RTKN | Rhotekin | GC02M074454 |
| DNAJB4 | DnaJ Heat Shock Protein Family (Hsp40) Member B4 | GC01P077979 |
| RFX1 | Regulatory Factor X1 | GC19M013961 |
| PLCG2 | Phospholipase C Gamma 2 | GC16P081773 |
| NLRC4 | NLR Family CARD Domain Containing 4 | GC02M032224 |
| CDCA5 | Cell Division Cycle Associated 5 | GC11M065068 |
| NCOA5 | Nuclear Receptor Coactivator 5 | GC20M046060 |
| MIR365B | MicroRNA 365b | GC17P031572 |
| SOX21 | SRY-Box Transcription Factor 21 | GC13M094709 |
| SYMPK | Symplekin | GC19M045815 |
| IL16 | Interleukin 16 | GC15P081159 |
| ZNF592 | Zinc Finger Protein 592 | GC15P084751 |
| ATIC | 5-Aminoimidazole-4-Carboxamide Ribonucleotide Formyltransferase/IMP Cyclohydrolase | GC02P215311 |
| QDPR | Quinoid Dihydropteridine Reductase | GC04M017460 |
| SECISBP2 | SECIS Binding Protein 2 | GC09P089318 |
| RHD | Rh Blood Group D Antigen | GC01P025272 |
| ZKSCAN4 | Zinc Finger With KRAB And SCAN Domains 4 | GC06M028284 |
| PHF8 | PHD Finger Protein 8 | GC0XM053936 |
| CPEB4 | Cytoplasmic Polyadenylation Element Binding Protein 4 | GC05P173888 |
| EFNA5 | Ephrin A5 | GC05M107376 |
| CASP5 | Caspase 5 | GC11M104995 |
| SIGLEC1 | Sialic Acid Binding Ig Like Lectin 1 | GC20M003686 |
| FSTL5 | Follistatin Like 5 | GC04M161383 |
| GLIS3 | GLIS Family Zinc Finger 3 | GC09M003816 |
| CORT | Cortistatin | GC01P010449 |
| MIR302D | MicroRNA 302d | GC04M112648 |
| CLDN11 | Claudin 11 | GC03P170418 |
| HSPA2 | Heat Shock Protein Family A (Hsp70) Member 2 | GC14P064535 |
| SP140 | SP140 Nuclear Body Protein | GC02P230203 |
| CES1 | Carboxylesterase 1 | GC16M055836 |
| MASP2 | Mannan Binding Lectin Serine Peptidase 2 | GC01M011026 |
| RTCB | RNA 2',3'-Cyclic Phosphate And 5'-OH Ligase | GC22M032387 |
| MTBP | MDM2 Binding Protein | GC08P120426 |
| SMOC2 | SPARC Related Modular Calcium Binding 2 | GC06P168441 |
| HMGB3 | High Mobility Group Box 3 | GC0XP150980 |
| HLA-DMA | Major Histocompatibility Complex, Class II, DM Alpha | GC06M032950 |
| CSPG4 | Chondroitin Sulfate Proteoglycan 4 | GC15M075674 |
| SLIT3 | Slit Guidance Ligand 3 | GC05M168661 |
| HACE1 | HECT Domain And Ankyrin Repeat Containing E3 Ubiquitin Protein Ligase 1 | GC06M104682 |
| LFNG | LFNG O-Fucosylpeptide 3-Beta-N-Acetylglucosaminyltransferase | GC07P002512 |
| CBLB | Cbl Proto-Oncogene B | GC03M105655 |
| CDKN2D | Cyclin Dependent Kinase Inhibitor 2D | GC19M010566 |
| CYP2W1 | Cytochrome P450 Family 2 Subfamily W Member 1 | GC07P000983 |
| RP1 | RP1 Axonemal Microtubule Associated | GC08P054555 |
| UPP1 | Uridine Phosphorylase 1 | GC07P048088 |
| OTUD7B | OTU Deubiquitinase 7B | GC01M149937 |
| NLRC5 | NLR Family CARD Domain Containing 5 | GC16P056990 |
| ZIC2 | Zic Family Member 2 | GC13P099981 |
| ATOH8 | Atonal BHLH Transcription Factor 8 | GC02P085751 |
| PPP1CA | Protein Phosphatase 1 Catalytic Subunit Alpha | GC11M067415 |
| SAR1B | Secretion Associated Ras Related GTPase 1B | GC05M134601 |
| BNC1 | Basonuclin 1 | GC15M083255 |
| MIR548C | MicroRNA 548c | GC12P064622 |
| STK38 | Serine/Threonine Kinase 38 | GC06M036493 |
| SELL | Selectin L | GC01M169690 |
| EDIL3 | EGF Like Repeats And Discoidin Domains 3 | GC05M083940 |
| SDC2 | Syndecan 2 | GC08P096494 |
| FAM83D | Family With Sequence Similarity 83 Member D | GC20P038926 |
| NKX2-2 | NK2 Homeobox 2 | GC20M021511 |
| LOC108251797 | HCCAT5-C16orf47 Intergenic CAGE-Defined High Expression Enhancer | GC16U902433 |
| H4C2 | H4 Clustered Histone 2 | GC06M026026 |
| H4C4 | H4 Clustered Histone 4 | GC06M027065 |
| CCNC | Cyclin C | GC06M099542 |
| UBE2T | Ubiquitin Conjugating Enzyme E2 T | GC01M202300 |
| COX8A | Cytochrome C Oxidase Subunit 8A | GC11P063977 |
| FASTKD2 | FAST Kinase Domains 2 | GC02P206766 |
| CIZ1 | CDKN1A Interacting Zinc Finger Protein 1 | GC09M128166 |
| BCL2L15 | BCL2 Like 15 | GC01M113876 |
| FAM189B | Family With Sequence Similarity 189 Member B | GC01M155248 |
| ITIH4 | Inter-Alpha-Trypsin Inhibitor Heavy Chain 4 | GC03M052812 |
| HERC5 | HECT And RLD Domain Containing E3 Ubiquitin Protein Ligase 5 | GC04P088457 |
| S100A10 | S100 Calcium Binding Protein A10 | GC01M151955 |
| BCORL1 | BCL6 Corepressor Like 1 | GC0XP129980 |
| PPP1CC | Protein Phosphatase 1 Catalytic Subunit Gamma | GC12M110709 |
| PPP1R3B | Protein Phosphatase 1 Regulatory Subunit 3B | GC08M009136 |
| KCNJ5 | Potassium Inwardly Rectifying Channel Subfamily J Member 5 | GC11P128891 |
| SSRP1 | Structure Specific Recognition Protein 1 | GC11M059784 |
| GMPR | Guanosine Monophosphate Reductase | GC06P016238 |
| SSBP1 | Single Stranded DNA Binding Protein 1 | GC07P144876 |
| MGAT4A | Alpha-1,3-Mannosyl-Glycoprotein 4-Beta-N-Acetylglucosaminyltransferase A | GC02M098619 |
| ATP5IF1 | ATP Synthase Inhibitory Factor Subunit 1 | GC01P028237 |
| PDLIM7 | PDZ And LIM Domain 7 | GC05M177483 |
| MRPL45 | Mitochondrial Ribosomal Protein L45 | GC17P038297 |
| BMF | Bcl2 Modifying Factor | GC15M040087 |
| ADAMTS4 | ADAM Metallopeptidase With Thrombospondin Type 1 Motif 4 | GC01M161184 |
| NEIL1 | Nei Like DNA Glycosylase 1 | GC15P075346 |
| GKN1 | Gastrokine 1 | GC02P068974 |
| RASSF6 | Ras Association Domain Family Member 6 | GC04M073571 |
| ST7L | Suppression Of Tumorigenicity 7 Like | GC01M112523 |
| FKBP10 | FKBP Prolyl Isomerase 10 | GC17P041812 |
| AJAP1 | Adherens Junctions Associated Protein 1 | GC01P004654 |
| PGM1 | Phosphoglucomutase 1 | GC01P063593 |
| SPG11 | SPG11 Vesicle Trafficking Associated, Spatacsin | GC15M044562 |
| H2BC21 | H2B Clustered Histone 21 | GC01M149925 |
| HNRNPAB | Heterogeneous Nuclear Ribonucleoprotein A/B | GC05P178204 |
| PIGU | Phosphatidylinositol Glycan Anchor Biosynthesis Class U | GC20M034560 |
| MIR30C2 | MicroRNA 30c-2 | GC06M071382 |
| ACSS2 | Acyl-CoA Synthetase Short Chain Family Member 2 | GC20P034873 |
| PFKFB2 | 6-Phosphofructo-2-Kinase/Fructose-2,6-Biphosphatase 2 | GC01P207034 |
| ADAMTS13 | ADAM Metallopeptidase With Thrombospondin Type 1 Motif 13 | GC09P133414 |
| KIR3DL1 | Killer Cell Immunoglobulin Like Receptor, Three Ig Domains And Long Cytoplasmic Tail 1 | GC19P055293 |
| ARHGAP5 | Rho GTPase Activating Protein 5 | GC14P032076 |
| PDE2A | Phosphodiesterase 2A | GC11M072576 |
| RPL4 | Ribosomal Protein L4 | GC15M066498 |
| LOC108961161 | POU5F1 5' Regulatory Region | GC06U903606 |
| SIK2 | Salt Inducible Kinase 2 | GC11P111605 |
| HLA-DQB2 | Major Histocompatibility Complex, Class II, DQ Beta 2 | GC06M032756 |
| POLI | DNA Polymerase Iota | GC18P054274 |
| PARP2 | Poly(ADP-Ribose) Polymerase 2 | GC14P020343 |
| POLR2A | RNA Polymerase II Subunit A | GC17P007934 |
| MIR1285-1 | MicroRNA 1285-1 | GC07M092204 |
| ASIC1 | Acid Sensing Ion Channel Subunit 1 | GC12P050057 |
| H1-5 | H1.5 Linker Histone, Cluster Member | GC06M028286 |
| EMP3 | Epithelial Membrane Protein 3 | GC19P048321 |
| KRT78 | Keratin 78 | GC12M052837 |
| BTF3 | Basic Transcription Factor 3 | GC05P073498 |
| MIR644A | MicroRNA 644a | GC20P034468 |
| MIR1271 | MicroRNA 1271 | GC05P176367 |
| NBPF12 | NBPF Member 12 | GC01P146938 |
| PSEN1 | Presenilin 1 | GC14P073136 |
| ME1 | Malic Enzyme 1 | GC06M083210 |
| CPA5 | Carboxypeptidase A5 | GC07P130344 |
| ABCB6 | ATP Binding Cassette Subfamily B Member 6 (Langereis Blood Group) | GC02M219209 |
| EXT1 | Exostosin Glycosyltransferase 1 | GC08M117798 |
| MIR411 | MicroRNA 411 | GC14P104465 |
| SPTBN2 | Spectrin Beta, Non-Erythrocytic 2 | GC11M066684 |
| IMPA1 | Inositol Monophosphatase 1 | GC08M081656 |
| STING1 | Stimulator Of Interferon Response CGAMP Interactor 1 | GC05M139476 |
| ASPRV1 | Aspartic Peptidase Retroviral Like 1 | GC02M069960 |
| UBR5 | Ubiquitin Protein Ligase E3 Component N-Recognin 5 | GC08M102252 |
| COPS3 | COP9 Signalosome Subunit 3 | GC17M017246 |
| PRR11 | Proline Rich 11 | GC17P059155 |
| BNIP3L | BCL2 Interacting Protein 3 Like | GC08P026296 |
| UBE2L3 | Ubiquitin Conjugating Enzyme E2 L3 | GC22P021549 |
| MIR433 | MicroRNA 433 | GC14P104250 |
| FAIM2 | Fas Apoptotic Inhibitory Molecule 2 | GC12M049866 |
| KIF18A | Kinesin Family Member 18A | GC11M028042 |
| CCNL2 | Cyclin L2 | GC01M001385 |
| CIITA | Class II Major Histocompatibility Complex Transactivator | GC16P010879 |
| SLC25A37 | Solute Carrier Family 25 Member 37 | GC08P023528 |
| CLEC16A | C-Type Lectin Domain Containing 16A | GC16P010944 |
| SSX5 | SSX Family Member 5 | GC0XM048186 |
| POU2F3 | POU Class 2 Homeobox 3 | GC11P120236 |
| UTS2R | Urotensin 2 Receptor | GC17P082374 |
| ARHGEF5 | Rho Guanine Nucleotide Exchange Factor 5 | GC07P144355 |
| USP15 | Ubiquitin Specific Peptidase 15 | GC12P062260 |
| FRK | Fyn Related Src Family Tyrosine Kinase | GC06M115931 |
| CCL1 | C-C Motif Chemokine Ligand 1 | GC17M034398 |
| PPP6C | Protein Phosphatase 6 Catalytic Subunit | GC09M125147 |
| CRNKL1 | Crooked Neck Pre-MRNA Splicing Factor 1 | GC20M020034 |
| P2RX3 | Purinergic Receptor P2X 3 | GC11P057356 |
| FOXJ2 | Forkhead Box J2 | GC12P008032 |
| RXFP2 | Relaxin Family Peptide Receptor 2 | GC13P031739 |
| TATDN1 | TatD DNase Domain Containing 1 | GC08M124488 |
| PSMC5 | Proteasome 26S Subunit, ATPase 5 | GC17P063827 |
| DHX15 | DEAH-Box Helicase 15 | GC04M024519 |
| GARS1 | Glycyl-TRNA Synthetase 1 | GC07P030595 |
| DNM1L | Dynamin 1 Like | GC12P032679 |
| ORMDL3 | ORMDL Sphingolipid Biosynthesis Regulator 3 | GC17M039921 |
| ANKLE1 | Ankyrin Repeat And LEM Domain Containing 1 | GC19P022226 |
| HES7 | Hes Family BHLH Transcription Factor 7 | GC17M008120 |
| CFC1 | Cripto, FRL-1, Cryptic Family 1 | GC02M130592 |
| MESP2 | Mesoderm Posterior BHLH Transcription Factor 2 | GC15P089764 |
| PAGR1 | PAXIP1 Associated Glutamate Rich Protein 1 | GC16P030050 |
| MYHAS | Myosin Heavy Chain Gene Cluster Antisense RNA | GC17P010383 |
| COL3A1 | Collagen Type III Alpha 1 Chain | GC02P188974 |
| PFKFB4 | 6-Phosphofructo-2-Kinase/Fructose-2,6-Biphosphatase 4 | GC03M048517 |
| PRSS3 | Serine Protease 3 | GC09P033750 |
| SCYL2 | SCY1 Like Pseudokinase 2 | GC12P100267 |
| RNF8 | Ring Finger Protein 8 | GC06P046068 |
| GLRX | Glutaredoxin | GC05M095752 |
| GIT1 | GIT ArfGAP 1 | GC17M029573 |
| AOC3 | Amine Oxidase Copper Containing 3 | GC17P042851 |
| XCR1 | X-C Motif Chemokine Receptor 1 | GC03M046037 |
| APC2 | APC Regulator Of WNT Signaling Pathway 2 | GC19P001473 |
| CMTM5 | CKLF Like MARVEL Transmembrane Domain Containing 5 | GC14P025012 |
| BCL2L14 | BCL2 Like 14 | GC12P012049 |
| CPT2 | Carnitine Palmitoyltransferase 2 | GC01P053196 |
| GBA3 | Glucosylceramidase Beta 3 (Gene/Pseudogene) | GC04P022694 |
| PI4K2A | Phosphatidylinositol 4-Kinase Type 2 Alpha | GC10P097640 |
| TROAP | Trophinin Associated Protein | GC12P049323 |
| CISD2 | CDGSH Iron Sulfur Domain 2 | GC04P102868 |
| PATZ1 | POZ/BTB And AT Hook Containing Zinc Finger 1 | GC22M031325 |
| NSUN2 | NOP2/Sun RNA Methyltransferase 2 | GC05M006599 |
| CFAP45 | Cilia And Flagella Associated Protein 45 | GC01M159873 |
| ASAP3 | ArfGAP With SH3 Domain, Ankyrin Repeat And PH Domain 3 | GC01M023428 |
| IFI6 | Interferon Alpha Inducible Protein 6 | GC01M027666 |
| TREM1 | Triggering Receptor Expressed On Myeloid Cells 1 | GC06M041267 |
| C9orf72 | C9orf72-SMCR8 Complex Subunit | GC09M027539 |
| SMYD4 | SET And MYND Domain Containing 4 | GC17M001779 |
| MIR203B | MicroRNA 203b | GC14M104118 |
| RLF | Rearranged L-Myc Fusion | GC01P040161 |
| GLG1 | Golgi Glycoprotein 1 | GC16M074448 |
| CTNNA3 | Catenin Alpha 3 | GC10M065912 |
| GNAO1 | G Protein Subunit Alpha O1 | GC16P056231 |
| ADAMTS9 | ADAM Metallopeptidase With Thrombospondin Type 1 Motif 9 | GC03M064501 |
| HOXA7 | Homeobox A7 | GC07M027153 |
| CASP4 | Caspase 4 | GC11M104942 |
| PUF60 | Poly(U) Binding Splicing Factor 60 | GC08M143816 |
| AARS1 | Alanyl-TRNA Synthetase 1 | GC16M070257 |
| NUDT21 | Nudix Hydrolase 21 | GC16M056429 |
| KRT12 | Keratin 12 | GC17M040861 |
| CUEDC2 | CUE Domain Containing 2 | GC10M102424 |
| CD200R1 | CD200 Receptor 1 | GC03M112921 |
| MT1A | Metallothionein 1A | GC16P056638 |
| PDIA6 | Protein Disulfide Isomerase Family A Member 6 | GC02M010774 |
| LOC108942766 | NANOG 5' Regulatory Region | GC12U902703 |
| CDCA7L | Cell Division Cycle Associated 7 Like | GC07M021900 |
| WARS1 | Tryptophanyl-TRNA Synthetase 1 | GC14M100334 |
| FBXO22 | F-Box Protein 22 | GC15P075903 |
| MAGEC1 | MAGE Family Member C1 | GC0XP141905 |
| CPEB3 | Cytoplasmic Polyadenylation Element Binding Protein 3 | GC10M092046 |
| SALL3 | Spalt Like Transcription Factor 3 | GC18P078980 |
| ENPP7 | Ectonucleotide Pyrophosphatase/Phosphodiesterase 7 | GC17P079730 |
| H3-4 | H3.4 Histone | GC01M228427 |
| DAPK2 | Death Associated Protein Kinase 2 | GC15M063907 |
| DBT | Dihydrolipoamide Branched Chain Transacylase E2 | GC01M100186 |
| DPP6 | Dipeptidyl Peptidase Like 6 | GC07P153887 |
| SENP2 | SUMO Specific Peptidase 2 | GC03P185582 |
| ALX4 | ALX Homeobox 4 | GC11M044238 |
| MIR153-2 | MicroRNA 153-2 | GC07M157574 |
| MIR3127 | MicroRNA 3127 | GC02P096798 |
| TAF1 | TATA-Box Binding Protein Associated Factor 1 | GC0XP071366 |
| RBMY1A1 | RNA Binding Motif Protein Y-Linked Family 1 Member A1 | GC0YP021534 |
| RSPO2 | R-Spondin 2 | GC08M107899 |
| SMARCC1 | SWI/SNF Related, Matrix Associated, Actin Dependent Regulator Of Chromatin Subfamily C Member 1 | GC03M047585 |
| NCBP1 | Nuclear Cap Binding Protein Subunit 1 | GC09P097633 |
| SRXN1 | Sulfiredoxin 1 | GC20M000647 |
| TRPM2 | Transient Receptor Potential Cation Channel Subfamily M Member 2 | GC21P044350 |
| CDK13 | Cyclin Dependent Kinase 13 | GC07P039983 |
| AMPD3 | Adenosine Monophosphate Deaminase 3 | GC11P010309 |
| CSNK1D | Casein Kinase 1 Delta | GC17M082239 |
| GLYATL1 | Glycine-N-Acyltransferase Like 1 | GC11P058906 |
| CTH | Cystathionine Gamma-Lyase | GC01P070411 |
| MRC2 | Mannose Receptor C Type 2 | GC17P062627 |
| FBXO31 | F-Box Protein 31 | GC16M087326 |
| TSPYL5 | TSPY Like 5 | GC08M097273 |
| IRX3 | Iroquois Homeobox 3 | GC16M054283 |
| GYS2 | Glycogen Synthase 2 | GC12M021531 |
| PROCR | Protein C Receptor | GC20P035171 |
| IL12RB2 | Interleukin 12 Receptor Subunit Beta 2 | GC01P067307 |
| BNC2 | Basonuclin 2 | GC09M016410 |
| MIR650 | MicroRNA 650 | GC22P022822 |
| PCSK9 | Proprotein Convertase Subtilisin/Kexin Type 9 | GC01P055039 |
| C1QTNF6 | C1q And TNF Related 6 | GC22M037180 |
| GABBR1 | Gamma-Aminobutyric Acid Type B Receptor Subunit 1 | GC06M029555 |
| LRPAP1 | LDL Receptor Related Protein Associated Protein 1 | GC04M003508 |
| FCN3 | Ficolin 3 | GC01M027379 |
| IQCE | IQ Motif Containing E | GC07P002558 |
| RPL5 | Ribosomal Protein L5 | GC01P092832 |
| RPS15A | Ribosomal Protein S15a | GC16M018781 |
| MTHFD2 | Methylenetetrahydrofolate Dehydrogenase (NADP+ Dependent) 2, Methenyltetrahydrofolate Cyclohydrolase | GC02P074198 |
| SIX2 | SIX Homeobox 2 | GC02M045005 |
| PPA1 | Inorganic Pyrophosphatase 1 | GC10M070202 |
| H4C6 | H4 Clustered Histone 6 | GC06P028390 |
| SLC46A1 | Solute Carrier Family 46 Member 1 | GC17M029697 |
| FCGRT | Fc Fragment Of IgG Receptor And Transporter | GC19P049506 |
| ITPR3 | Inositol 1,4,5-Trisphosphate Receptor Type 3 | GC06P033620 |
| SSX2 | SSX Family Member 2 | GC0XM052696 |
| MPG | N-Methylpurine DNA Glycosylase | GC16P001209 |
| SMARCA5 | SWI/SNF Related, Matrix Associated, Actin Dependent Regulator Of Chromatin, Subfamily A, Member 5 | GC04P143513 |
| FMNL2 | Formin Like 2 | GC02P152335 |
| VPS35 | VPS35 Retromer Complex Component | GC16M046678 |
| PSG9 | Pregnancy Specific Beta-1-Glycoprotein 9 | GC19M043212 |
| INCENP | Inner Centromere Protein | GC11P062142 |
| SUMO3 | Small Ubiquitin Like Modifier 3 | GC21M044805 |
| POFUT1 | Protein O-Fucosyltransferase 1 | GC20P032207 |
| MIR592 | MicroRNA 592 | GC07M127058 |
| TEX14 | Testis Expressed 14, Intercellular Bridge Forming Factor | GC17M058556 |
| ATP10A | ATPase Phospholipid Transporting 10A (Putative) | GC15M025666 |
| KLHL12 | Kelch Like Family Member 12 | GC01M202891 |
| ATP9B | ATPase Phospholipid Transporting 9B (Putative) | GC18P079047 |
| MOB3B | MOB Kinase Activator 3B | GC09M027316 |
| ZPBP2 | Zona Pellucida Binding Protein 2 | GC17P039869 |
| ZIM2-AS1 | ZIM2 Antisense RNA 1 | GC19P056596 |
| TAT-AS1 | TAT Antisense RNA 1 | GC16P071565 |
| MAGEE1 | MAGE Family Member E1 | GC0XP076427 |
| NSMCE3 | NSE3 Homolog, SMC5-SMC6 Complex Component | GC15M029269 |
| MAGEC3 | MAGE Family Member C3 | GC0XP141838 |
| MAGEE2 | MAGE Family Member E2 | GC0XM075782 |
| TWIST2 | Twist Family BHLH Transcription Factor 2 | GC02P238848 |
| REXO1L1P | REXO1 Like 1, Pseudogene | GC08M085656 |
| YTHDF2 | YTH N6-Methyladenosine RNA Binding Protein 2 | GC01P028751 |
| INTS5 | Integrator Complex Subunit 5 | GC11M063075 |
| IQGAP2 | IQ Motif Containing GTPase Activating Protein 2 | GC05P076403 |
| PHF19 | PHD Finger Protein 19 | GC09M120855 |
| MIR876 | MicroRNA 876 | GC09M028855 |
| NCR3 | Natural Cytotoxicity Triggering Receptor 3 | GC06M031588 |
| MRTFB | Myocardin Related Transcription Factor B | GC16P014071 |
| ORAI1 | ORAI Calcium Release-Activated Calcium Modulator 1 | GC12P122635 |
| MT1H | Metallothionein 1H | GC16P056670 |
| RAB10 | RAB10, Member RAS Oncogene Family | GC02P026033 |
| PRDM5 | PR/SET Domain 5 | GC04M120686 |
| RNF38 | Ring Finger Protein 38 | GC09M036336 |
| LOC110594336 | MS1 Minisatellite Repeat Instability Region | GC01U905800 |
| MIR492 | MicroRNA 492 | GC12P094834 |
| SEPTIN2 | Septin 2 | GC02P241316 |
| PCBP1 | Poly(RC) Binding Protein 1 | GC02P070052 |
| TLE3 | TLE Family Member 3, Transcriptional Corepressor | GC15M070047 |
| TCIRG1 | T Cell Immune Regulator 1, ATPase H+ Transporting V0 Subunit A3 | GC11P068038 |
| PSMC3 | Proteasome 26S Subunit, ATPase 3 | GC11M059696 |
| DOK1 | Docking Protein 1 | GC02P074549 |
| MIR379 | MicroRNA 379 | GC14P104455 |
| ATP6V1C1 | ATPase H+ Transporting V1 Subunit C1 | GC08P103038 |
| SH3PXD2A | SH3 And PX Domains 2A | GC10M103594 |
| NDUFS4 | NADH:Ubiquinone Oxidoreductase Subunit S4 | GC05P053560 |
| LOC107982234 | WT1/WT1-AS Bi-Directional Promoter Region | GC11U903004 |
| MKNK1 | MAPK Interacting Serine/Threonine Kinase 1 | GC01M046557 |
| EP400 | E1A Binding Protein P400 | GC12P131949 |
| MAPK6 | Mitogen-Activated Protein Kinase 6 | GC15P051952 |
| LPAR6 | Lysophosphatidic Acid Receptor 6 | GC13M048389 |
| CRYL1 | Crystallin Lambda 1 | GC13M020403 |
| GSTA3 | Glutathione S-Transferase Alpha 3 | GC06M052896 |
| WEE2 | WEE2 Oocyte Meiosis Inhibiting Kinase | GC07P141708 |
| RPL38 | Ribosomal Protein L38 | GC17P074204 |
| RAB18 | RAB18, Member RAS Oncogene Family | GC10P027504 |
| ASRGL1 | Asparaginase And Isoaspartyl Peptidase 1 | GC11P062337 |
| SEC62 | SEC62 Homolog, Preprotein Translocation Factor | GC03P169966 |
| EBI3 | Epstein-Barr Virus Induced 3 | GC19P004231 |
| OVOL2 | Ovo Like Zinc Finger 2 | GC20M017956 |
| CARD10 | Caspase Recruitment Domain Family Member 10 | GC22M044545 |
| INSL3 | Insulin Like 3 | GC19M017816 |
| LRIG2 | Leucine Rich Repeats And Immunoglobulin Like Domains 2 | GC01P113073 |
| MCUR1 | Mitochondrial Calcium Uniporter Regulator 1 | GC06M013788 |
| RBM3 | RNA Binding Motif Protein 3 | GC0XP048574 |
| CMKLR1 | Chemerin Chemokine-Like Receptor 1 | GC12M108288 |
| GBA | Glucosylceramidase Beta | GC01M155234 |
| PRKCH | Protein Kinase C Eta | GC14P061187 |
| UBE2S | Ubiquitin Conjugating Enzyme E2 S | GC19M055399 |
| PPBP | Pro-Platelet Basic Protein | GC04M073986 |
| PAPPA | Pappalysin 1 | GC09P116171 |
| GSTM4 | Glutathione S-Transferase Mu 4 | GC01P109657 |
| CLEC12A | C-Type Lectin Domain Family 12 Member A | GC12P009951 |
| CACUL1 | CDK2 Associated Cullin Domain 1 | GC10M118674 |
| SUMO2 | Small Ubiquitin Like Modifier 2 | GC17M075165 |
| H4C9 | H4 Clustered Histone 9 | GC06P028391 |
| H4C5 | H4 Clustered Histone 5 | GC06P028389 |
| H4C15 | H4 Clustered Histone 15 | GC01M149918 |
| SYNCRIP | Synaptotagmin Binding Cytoplasmic RNA Interacting Protein | GC06M085607 |
| INPP5D | Inositol Polyphosphate-5-Phosphatase D | GC02P233059 |
| SENP1 | SUMO Specific Peptidase 1 | GC12M048042 |
| BLMH | Bleomycin Hydrolase | GC17M030248 |
| NUCKS1 | Nuclear Casein Kinase And Cyclin Dependent Kinase Substrate 1 | GC01M205681 |
| IRAK3 | Interleukin 1 Receptor Associated Kinase 3 | GC12P066188 |
| DAD1 | Defender Against Cell Death 1 | GC14M022565 |
| GPA33 | Glycoprotein A33 | GC01M167052 |
| GPANK1 | G-Patch Domain And Ankyrin Repeats 1 | GC06M032372 |
| PDCD6IP | Programmed Cell Death 6 Interacting Protein | GC03P033798 |
| SRRD | SRR1 Domain Containing | GC22P026483 |
| RAP2B | RAP2B, Member Of RAS Oncogene Family | GC03P153162 |
| XCL1 | X-C Motif Chemokine Ligand 1 | GC01P168576 |
| EIF2AK4 | Eukaryotic Translation Initiation Factor 2 Alpha Kinase 4 | GC15P039934 |
| GDI2 | GDP Dissociation Inhibitor 2 | GC10M005765 |
| H2BS1 | H2B.S Histone 1 | GC21P043567 |
| HGS | Hepatocyte Growth Factor-Regulated Tyrosine Kinase Substrate | GC17P081683 |
| DGCR8 | DGCR8 Microprocessor Complex Subunit | GC22P020080 |
| SCARA3 | Scavenger Receptor Class A Member 3 | GC08P027633 |
| MIR577 | MicroRNA 577 | GC04P114656 |
| EPHA10 | EPH Receptor A10 | GC01M037713 |
| TMOD1 | Tropomodulin 1 | GC09P097501 |
| SETD1B | SET Domain Containing 1B, Histone Lysine Methyltransferase | GC12P122640 |
| MIR1207 | MicroRNA 1207 | GC08P128049 |
| SHMT2 | Serine Hydroxymethyltransferase 2 | GC12P057229 |
| CARD9 | Caspase Recruitment Domain Family Member 9 | GC09M136361 |
| THOP1 | Thimet Oligopeptidase 1 | GC19P002785 |
| PROM2 | Prominin 2 | GC02P095274 |
| LOC109113863 | FGF2 Promoter Region | GC04U902745 |
| TRIP12 | Thyroid Hormone Receptor Interactor 12 | GC02M229763 |
| BUB3 | BUB3 Mitotic Checkpoint Protein | GC10P123154 |
| CFD | Complement Factor D | GC19P000859 |
| BPI | Bactericidal Permeability Increasing Protein | GC20P038304 |
| FIP1L1 | Factor Interacting With PAPOLA And CPSF1 | GC04P053383 |
| DENND1B | DENN Domain Containing 1B | GC01M197473 |
| TYRO3 | TYRO3 Protein Tyrosine Kinase | GC15P041557 |
| PKMYT1 | Protein Kinase, Membrane Associated Tyrosine/Threonine 1 | GC16M002969 |
| GTSE1 | G2 And S-Phase Expressed 1 | GC22P046296 |
| CCNY | Cyclin Y | GC10P035254 |
| CD2BP2 | CD2 Cytoplasmic Tail Binding Protein 2 | GC16M030350 |
| PIK3IP1 | Phosphoinositide-3-Kinase Interacting Protein 1 | GC22M031281 |
| SYNJ2BP | Synaptojanin 2 Binding Protein | GC14M070366 |
| PCNT | Pericentrin | GC21P046324 |
| LETMD1 | LETM1 Domain Containing 1 | GC12P051047 |
| IDE | Insulin Degrading Enzyme | GC10M092451 |
| TUBB1 | Tubulin Beta 1 Class VI | GC20P059020 |
| PRKCSH | Protein Kinase C Substrate 80K-H | GC19P011435 |
| CSNK1E | Casein Kinase 1 Epsilon | GC22M044617 |
| COL11A2 | Collagen Type XI Alpha 2 Chain | GC06M033162 |
| GHSR | Growth Hormone Secretagogue Receptor | GC03M172443 |
| LIMD1 | LIM Domains Containing 1 | GC03P045555 |
| PDE4D | Phosphodiesterase 4D | GC05M058969 |
| UGT2B15 | UDP Glucuronosyltransferase Family 2 Member B15 | GC04M068646 |
| NASP | Nuclear Autoantigenic Sperm Protein | GC01P045583 |
| MAPKAP1 | MAPK Associated Protein 1 | GC09M125437 |
| SORBS3 | Sorbin And SH3 Domain Containing 3 | GC08P022544 |
| SEPTIN7 | Septin 7 | GC07P035828 |
| RAB7A | RAB7A, Member RAS Oncogene Family | GC03P128737 |
| DKK2 | Dickkopf WNT Signaling Pathway Inhibitor 2 | GC04M106921 |
| LOXL1 | Lysyl Oxidase Like 1 | GC15P073925 |
| NOP53 | NOP53 Ribosome Biogenesis Factor | GC19P047746 |
| LTB | Lymphotoxin Beta | GC06M032353 |
| MAS1 | MAS1 Proto-Oncogene, G Protein-Coupled Receptor | GC06P159906 |
| CNTF | Ciliary Neurotrophic Factor | GC11P058622 |
| CFI | Complement Factor I | GC04M109740 |
| PSPH | Phosphoserine Phosphatase | GC07M056010 |
| CITED2 | Cbp/P300 Interacting Transactivator With Glu/Asp Rich Carboxy-Terminal Domain 2 | GC06M139371 |
| CUL4B | Cullin 4B | GC0XM120524 |
| TNS2 | Tensin 2 | GC12P053046 |
| ERAP2 | Endoplasmic Reticulum Aminopeptidase 2 | GC05P096875 |
| ULBP1 | UL16 Binding Protein 1 | GC06P149963 |
| WLS | Wnt Ligand Secretion Mediator | GC01M068098 |
| CD22 | CD22 Molecule | GC19P035319 |
| SYNE2 | Spectrin Repeat Containing Nuclear Envelope Protein 2 | GC14P063852 |
| EVI5 | Ecotropic Viral Integration Site 5 | GC01M092508 |
| CPT1A | Carnitine Palmitoyltransferase 1A | GC11M068754 |
| DLG2 | Discs Large MAGUK Scaffold Protein 2 | GC11M083455 |
| ANP32B | Acidic Nuclear Phosphoprotein 32 Family Member B | GC09P097983 |
| DHX33 | DEAH-Box Helicase 33 | GC17M005440 |
| NRSN2 | Neurensin 2 | GC20P000346 |
| MIR873 | MicroRNA 873 | GC09M028880 |
| TEC | Tec Protein Tyrosine Kinase | GC04M048137 |
| AMY1A | Amylase Alpha 1A | GC01P103651 |
| HLA-DRB5 | Major Histocompatibility Complex, Class II, DR Beta 5 | GC06M032530 |
| MTCO3P1 | MT-CO3 Pseudogene 1 | GC06M032706 |
| ZNHIT3 | Zinc Finger HIT-Type Containing 3 | GC17P036486 |
| MIR301B | MicroRNA 301b | GC22P023731 |
| SLC26A5 | Solute Carrier Family 26 Member 5 | GC07M103352 |
| UQCRH | Ubiquinol-Cytochrome C Reductase Hinge Protein | GC01P046303 |
| PDZD7 | PDZ Domain Containing 7 | GC10M101007 |
| LXN | Latexin | GC03M158645 |
| EIF2S2 | Eukaryotic Translation Initiation Factor 2 Subunit Beta | GC20M034088 |
| PEX1 | Peroxisomal Biogenesis Factor 1 | GC07M092487 |
| FOXG1 | Forkhead Box G1 | GC14P028766 |
| SNAPIN | SNAP Associated Protein | GC01P153660 |
| ACE2 | Angiotensin I Converting Enzyme 2 | GC0XM015494 |
| MASP1 | Mannan Binding Lectin Serine Peptidase 1 | GC03M187216 |
| SLC22A2 | Solute Carrier Family 22 Member 2 | GC06M160173 |
| PAXIP1 | PAX Interacting Protein 1 | GC07M154943 |
| SLCO2B1 | Solute Carrier Organic Anion Transporter Family Member 2B1 | GC11P075718 |
| EIF3I | Eukaryotic Translation Initiation Factor 3 Subunit I | GC01P032221 |
| LAMP3 | Lysosomal Associated Membrane Protein 3 | GC03M183122 |
| CENPB | Centromere Protein B | GC20M003783 |
| SNW1 | SNW Domain Containing 1 | GC14M077717 |
| PIDD1 | P53-Induced Death Domain Protein 1 | GC11M000800 |
| NOD1 | Nucleotide Binding Oligomerization Domain Containing 1 | GC07M030424 |
| SULT2B1 | Sulfotransferase Family 2B Member 1 | GC19P048552 |
| CDC16 | Cell Division Cycle 16 | GC13P114234 |
| AHSP | Alpha Hemoglobin Stabilizing Protein | GC16P031527 |
| CDK20 | Cyclin Dependent Kinase 20 | GC09M087966 |
| FBP2 | Fructose-Bisphosphatase 2 | GC09M094558 |
| CAPN1 | Calpain 1 | GC11P065198 |
| WNT8A | Wnt Family Member 8A | GC05P138095 |
| ACKR1 | Atypical Chemokine Receptor 1 (Duffy Blood Group) | GC01P159203 |
| MBD1 | Methyl-CpG Binding Domain Protein 1 | GC18M050266 |
| FHL1 | Four And A Half LIM Domains 1 | GC0XP136146 |
| LOC110467515 | CYP1A1 5' Regulatory Region | GC15U902415 |
| MPZL1 | Myelin Protein Zero Like 1 | GC01P167721 |
| NEK6 | NIMA Related Kinase 6 | GC09P124259 |
| UQCC1 | Ubiquinol-Cytochrome C Reductase Complex Assembly Factor 1 | GC20M035302 |
| MIR520A | MicroRNA 520a | GC19P053690 |
| TUBA4A | Tubulin Alpha 4a | GC02M219249 |
| PPP5C | Protein Phosphatase 5 Catalytic Subunit | GC19P046346 |
| NPR1 | Natriuretic Peptide Receptor 1 | GC01P153691 |
| LINC00882 | Long Intergenic Non-Protein Coding RNA 882 | GC03M106449 |
| AHI1 | Abelson Helper Integration Site 1 | GC06M135283 |
| AQP4 | Aquaporin 4 | GC18M026852 |
| NFYA | Nuclear Transcription Factor Y Subunit Alpha | GC06P046092 |
| VAPB | VAMP Associated Protein B And C | GC20P058389 |
| CHIT1 | Chitinase 1 | GC01M203181 |
| FADS1 | Fatty Acid Desaturase 1 | GC11M061799 |
| GANAB | Glucosidase II Alpha Subunit | GC11M063073 |
| FBXW8 | F-Box And WD Repeat Domain Containing 8 | GC12P116910 |
| TRIM52 | Tripartite Motif Containing 52 | GC05M181254 |
| SELENOM | Selenoprotein M | GC22M031105 |
| MIR298 | MicroRNA 298 | GC20M058818 |
| MIR23C | MicroRNA 23c | GC0XM020037 |
| DENND2D | DENN Domain Containing 2D | GC01M111185 |
| RPS18 | Ribosomal Protein S18 | GC06P033521 |
| MAZ | MYC Associated Zinc Finger Protein | GC16P029806 |
| GTF2I | General Transcription Factor IIi | GC07P074658 |
| CYP2R1 | Cytochrome P450 Family 2 Subfamily R Member 1 | GC11M014856 |
| PPAT | Phosphoribosyl Pyrophosphate Amidotransferase | GC04M056393 |
| APOBEC3H | Apolipoprotein B MRNA Editing Enzyme Catalytic Subunit 3H | GC22P039097 |
| SLC38A1 | Solute Carrier Family 38 Member 1 | GC12M046183 |
| DHPS | Deoxyhypusine Synthase | GC19M012676 |
| PLAC8 | Placenta Associated 8 | GC04M083090 |
| BRF1 | BRF1 RNA Polymerase III Transcription Initiation Factor Subunit | GC14M105212 |
| MT-ND2 | Mitochondrially Encoded NADH:Ubiquinone Oxidoreductase Core Subunit 2 | GCMTP004472 |
| VDAC3 | Voltage Dependent Anion Channel 3 | GC08P042368 |
| ADH5 | Alcohol Dehydrogenase 5 (Class III), Chi Polypeptide | GC04M099070 |
| EIF3L | Eukaryotic Translation Initiation Factor 3 Subunit L | GC22P037848 |
| FBXO4 | F-Box Protein 4 | GC05P041925 |
| TSPAN4 | Tetraspanin 4 | GC11P000882 |
| CMTR2 | Cap Methyltransferase 2 | GC16M071315 |
| HNRNPM | Heterogeneous Nuclear Ribonucleoprotein M | GC19P008444 |
| KRT75 | Keratin 75 | GC12M052425 |
| RAC2 | Rac Family Small GTPase 2 | GC22M037227 |
| HAT1 | Histone Acetyltransferase 1 | GC02P171922 |
| TNFRSF4 | TNF Receptor Superfamily Member 4 | GC01M001211 |
| SELPLG | Selectin P Ligand | GC12M108621 |
| SPANXC | SPANX Family Member C | GC0XM141241 |
| AKR1B1 | Aldo-Keto Reductase Family 1 Member B | GC07M134442 |
| TUBA8 | Tubulin Alpha 8 | GC22P018110 |
| NPAS2 | Neuronal PAS Domain Protein 2 | GC02P100803 |
| WSB1 | WD Repeat And SOCS Box Containing 1 | GC17P027294 |
| PHF6 | PHD Finger Protein 6 | GC0XP134373 |
| PPM1F | Protein Phosphatase, Mg2+/Mn2+ Dependent 1F | GC22M021919 |
| RAB17 | RAB17, Member RAS Oncogene Family | GC02M237574 |
| ACTR1B | Actin Related Protein 1B | GC02M097638 |
| SH2D4A | SH2 Domain Containing 4A | GC08P019313 |
| BASP1 | Brain Abundant Membrane Attached Signal Protein 1 | GC05P017065 |
| ARHGAP18 | Rho GTPase Activating Protein 18 | GC06M129576 |
| HRC | Histidine Rich Calcium Binding Protein | GC19M049151 |
| RPL36A | Ribosomal Protein L36a | GC0XP101392 |
| VASN | Vasorin | GC16P004421 |
| RITA1 | RBPJ Interacting And Tubulin Associated 1 | GC12P113185 |
| MCM3AP-AS1 | MCM3AP Antisense RNA 1 | GC21P046229 |
| MIR1180 | MicroRNA 1180 | GC17M019344 |
| C1QTNF1-AS1 | C1QTNF1 Antisense RNA 1 | GC17M079160 |
| MIR487A | MicroRNA 487a | GC14P104468 |
| MIR105-1 | MicroRNA 105-1 | GC0XM152392 |
| MIR5692A2 | MicroRNA 5692a-2 | GC08P012719 |
| CALML3 | Calmodulin Like 3 | GC10P005556 |
| LMO4 | LIM Domain Only 4 | GC01P087329 |
| SLC7A11 | Solute Carrier Family 7 Member 11 | GC04M138164 |
| ARRB2 | Arrestin Beta 2 | GC17P004711 |
| HMGCS1 | 3-Hydroxy-3-Methylglutaryl-CoA Synthase 1 | GC05M043288 |
| HOXA4 | Homeobox A4 | GC07M027128 |
| HBE1 | Hemoglobin Subunit Epsilon 1 | GC11M005268 |
| ATPAF1 | ATP Synthase Mitochondrial F1 Complex Assembly Factor 1 | GC01M046632 |
| SPCS1 | Signal Peptidase Complex Subunit 1 | GC03P052807 |
| FBXL2 | F-Box And Leucine Rich Repeat Protein 2 | GC03P033293 |
| EGID-105180392 | Beta-Globin 3' Hypersensitive Site 1 | GC11U903001 |
| ADORA1 | Adenosine A1 Receptor | GC01P203090 |
| USP28 | Ubiquitin Specific Peptidase 28 | GC11M113797 |
| CIAPIN1 | Cytokine Induced Apoptosis Inhibitor 1 | GC16M057428 |
| CD3D | CD3d Molecule | GC11M118338 |
| DDX46 | DEAD-Box Helicase 46 | GC05P134758 |
| ZFYVE9 | Zinc Finger FYVE-Type Containing 9 | GC01P052142 |
| YWHAH | Tyrosine 3-Monooxygenase/Tryptophan 5-Monooxygenase Activation Protein Eta | GC22P031944 |
| C19orf48 | Chromosome 19 Open Reading Frame 48 | GC19M050797 |
| HLA-DQA2 | Major Histocompatibility Complex, Class II, DQ Alpha 2 | GC06P032741 |
| HLA-DRB6 | Major Histocompatibility Complex, Class II, DR Beta 6 (Pseudogene) | GC06M032555 |
| CYP21A1P | Cytochrome P450 Family 21 Subfamily A Member 1, Pseudogene | GC06P032005 |
| IPO7 | Importin 7 | GC11P009384 |
| PAQR3 | Progestin And AdipoQ Receptor Family Member 3 | GC04M078887 |
| CREM | CAMP Responsive Element Modulator | GC10P035126 |
| EIF3E | Eukaryotic Translation Initiation Factor 3 Subunit E | GC08M108201 |
| NR2E3 | Nuclear Receptor Subfamily 2 Group E Member 3 | GC15P071792 |
| WDR46 | WD Repeat Domain 46 | GC06M033279 |
| INA | Internexin Neuronal Intermediate Filament Protein Alpha | GC10P103277 |
| ADAM15 | ADAM Metallopeptidase Domain 15 | GC01P155023 |
| LUC7L2 | LUC7 Like 2, Pre-MRNA Splicing Factor | GC07P139344 |
| BDKRB2 | Bradykinin Receptor B2 | GC14P096205 |
| OPRD1 | Opioid Receptor Delta 1 | GC01P028812 |
| PPIB | Peptidylprolyl Isomerase B | GC15M064155 |
| MIR1301 | MicroRNA 1301 | GC02M025328 |
| UNC5B | Unc-5 Netrin Receptor B | GC10P071212 |
| USP39 | Ubiquitin Specific Peptidase 39 | GC02P085635 |
| SSX1 | SSX Family Member 1 | GC0XP048266 |
| HERC4 | HECT And RLD Domain Containing E3 Ubiquitin Protein Ligase 4 | GC10M067921 |
| MIR18B | MicroRNA 18b | GC0XM134246 |
| H1-2 | H1.2 Linker Histone, Cluster Member | GC06M026056 |
| CD2AP | CD2 Associated Protein | GC06P047445 |
| SMC4 | Structural Maintenance Of Chromosomes 4 | GC03P160399 |
| F11R | F11 Receptor | GC01M160995 |
| SLC25A6 | Solute Carrier Family 25 Member 6 | GC0XM001386 |
| SCAMP3 | Secretory Carrier Membrane Protein 3 | GC01M155255 |
| PHETA1 | PH Domain Containing Endocytic Trafficking Adaptor 1 | GC12M111361 |
| KHDRBS3 | KH RNA Binding Domain Containing, Signal Transduction Associated 3 | GC08P135457 |
| FOXK2 | Forkhead Box K2 | GC17P082519 |
| TRPV2 | Transient Receptor Potential Cation Channel Subfamily V Member 2 | GC17P016415 |
| MIR634 | MicroRNA 634 | GC17P066787 |
| MYDGF | Myeloid Derived Growth Factor | GC19M004641 |
| SNHG18 | Small Nucleolar RNA Host Gene 18 | GC05P009563 |
| RAB38 | RAB38, Member RAS Oncogene Family | GC11M088113 |
| PARVA | Parvin Alpha | GC11P012398 |
| LINC00328 | Long Intergenic Non-Protein Coding RNA 328 | GC21U900445 |
| TLCD3A | TLC Domain Containing 3A | GC17P000733 |
| POTEF | POTE Ankyrin Domain Family Member F | GC02M130073 |
| BCAP31 | B Cell Receptor Associated Protein 31 | GC0XM153701 |
| GALNT10 | Polypeptide N-Acetylgalactosaminyltransferase 10 | GC05P154165 |
| RORC | RAR Related Orphan Receptor C | GC01M151806 |
| H2BC4 | H2B Clustered Histone 4 | GC06M027056 |
| PPIL3 | Peptidylprolyl Isomerase Like 3 | GC02M200870 |
| KMT5A | Lysine Methyltransferase 5A | GC12P123385 |
| SAGE1 | Sarcoma Antigen 1 | GC0XP135889 |
| INTS6P1 | Integrator Complex Subunit 6 Pseudogene 1 | GC05M039719 |
| MT4 | Metallothionein 4 | GC16P056565 |
| BTK | Bruton Tyrosine Kinase | GC0XM101349 |
| RPL39L | Ribosomal Protein L39 Like | GC03M187121 |
| MIR1236 | MicroRNA 1236 | GC06M032394 |
| SEC16B | SEC16 Homolog B, Endoplasmic Reticulum Export Factor | GC01M177923 |
| MIR520D | MicroRNA 520d | GC19P053720 |
| FOXJ1 | Forkhead Box J1 | GC17M076136 |
| TMEM98 | Transmembrane Protein 98 | GC17P032927 |
| PMM2 | Phosphomannomutase 2 | GC16P008788 |
| KLF1 | Kruppel Like Factor 1 | GC19M012884 |
| PANK2 | Pantothenate Kinase 2 | GC20P003887 |
| ALS2 | Alsin Rho Guanine Nucleotide Exchange Factor ALS2 | GC02M201701 |
| FIG4 | FIG4 Phosphoinositide 5-Phosphatase | GC06P109691 |
| PANK1 | Pantothenate Kinase 1 | GC10M089579 |
| EIF2B3 | Eukaryotic Translation Initiation Factor 2B Subunit Gamma | GC01M044850 |
| HBG1 | Hemoglobin Subunit Gamma 1 | GC11M005340 |
| DNAL1 | Dynein Axonemal Light Chain 1 | GC14P073644 |
| UNC13A | Unc-13 Homolog A | GC19M017602 |
| HBD | Hemoglobin Subunit Delta | GC11M005232 |
| ACY3 | Aminoacylase 3 | GC11M067642 |
| ATP8B3 | ATPase Phospholipid Transporting 8B3 | GC19M001794 |
| TBC1D20 | TBC1 Domain Family Member 20 | GC20M000435 |
| GTPBP1 | GTP Binding Protein 1 | GC22P038705 |
| NPRL3 | NPR3 Like, GATOR1 Complex Subunit | GC16M000084 |
| SLC51A | Solute Carrier Family 51 Subunit Alpha | GC03P196211 |
| ATP10B | ATPase Phospholipid Transporting 10B (Putative) | GC05M160499 |
| FGGY | FGGY Carbohydrate Kinase Domain Containing | GC01P059296 |
| SLC37A1 | Solute Carrier Family 37 Member 1 | GC21P042511 |
| NDUFAF5 | NADH:Ubiquinone Oxidoreductase Complex Assembly Factor 5 | GC20P013786 |
| SLC37A3 | Solute Carrier Family 37 Member 3 | GC07M140293 |
| PATL1 | PAT1 Homolog 1, Processing Body MRNA Decay Factor | GC11M059636 |
| INSL6 | Insulin Like 6 | GC09M004991 |
| RILPL2 | Rab Interacting Lysosomal Protein Like 2 | GC12M123410 |
| DDX60L | DExD/H-Box 60 Like | GC04M168356 |
| PLSCR5 | Phospholipid Scramblase Family Member 5 | GC03M146576 |
| TERB2 | Telomere Repeat Binding Bouquet Formation Protein 2 | GC15P044957 |
| MESTIT1 | MEST Intronic Transcript 1, Antisense RNA | GC07M130487 |
| MIR1537 | MicroRNA 1537 | GC01M235853 |
| LOC108783645 | HFE Antisense RNA | GC06U903556 |
| LOC109951029 | Delta-Globin 5' Regulatory Region | GC11U903008 |
| DDIAS | DNA Damage Induced Apoptosis Suppressor | GC11P082899 |
| MIR718 | MicroRNA 718 | GC0XM154019 |
| UHRF2 | Ubiquitin Like With PHD And Ring Finger Domains 2 | GC09P006405 |
| TEX10 | Testis Expressed 10 | GC09M100302 |
| CD3G | CD3g Molecule | GC11P118344 |
| VASP | Vasodilator Stimulated Phosphoprotein | GC19P045507 |
| CBLL1 | Cbl Proto-Oncogene Like 1 | GC07P107743 |
| DDX6 | DEAD-Box Helicase 6 | GC11M118748 |
| ZMYM2 | Zinc Finger MYM-Type Containing 2 | GC13P019958 |
| HOOK1 | Hook Microtubule Tethering Protein 1 | GC01P059814 |
| PPL | Periplakin | GC16M004872 |
| DDX17 | DEAD-Box Helicase 17 | GC22M038483 |
| POTEE | POTE Ankyrin Domain Family Member E | GC02P132458 |
| FBL | Fibrillarin | GC19M039834 |
| MORF4L1 | Mortality Factor 4 Like 1 | GC15P078810 |
| GLP1R | Glucagon Like Peptide 1 Receptor | GC06P039048 |
| C1QBP | Complement C1q Binding Protein | GC17M005432 |
| TMOD3 | Tropomodulin 3 | GC15P051829 |
| LPCAT1 | Lysophosphatidylcholine Acyltransferase 1 | GC05M001456 |
| DHRS4 | Dehydrogenase/Reductase 4 | GC14P023953 |
| SARDH | Sarcosine Dehydrogenase | GC09M133663 |
| RNF181 | Ring Finger Protein 181 | GC02P085636 |
| ABCD3 | ATP Binding Cassette Subfamily D Member 3 | GC01P094418 |
| NECTIN2 | Nectin Cell Adhesion Molecule 2 | GC19P044849 |
| MIR765 | MicroRNA 765 | GC01M156905 |
| CYB5R3 | Cytochrome B5 Reductase 3 | GC22M042617 |
| PCLO | Piccolo Presynaptic Cytomatrix Protein | GC07M082754 |
| SLC28A1 | Solute Carrier Family 28 Member 1 | GC15P084884 |
| MBP | Myelin Basic Protein | GC18M076978 |
| FUCA2 | Alpha-L-Fucosidase 2 | GC06M143494 |
| FGFRL1 | Fibroblast Growth Factor Receptor Like 1 | GC04P001011 |
| EEF1B2 | Eukaryotic Translation Elongation Factor 1 Beta 2 | GC02P206159 |
| PSME1 | Proteasome Activator Subunit 1 | GC14P024136 |
| SLC35A3 | Solute Carrier Family 35 Member A3 | GC01P099968 |
| TRIM59 | Tripartite Motif Containing 59 | GC03M160432 |
| RPS25 | Ribosomal Protein S25 | GC11M119015 |
| LAMB2 | Laminin Subunit Beta 2 | GC03M049121 |
| UNG | Uracil DNA Glycosylase | GC12P109097 |
| IFNGR1 | Interferon Gamma Receptor 1 | GC06M137197 |
| CDT1 | Chromatin Licensing And DNA Replication Factor 1 | GC16P088803 |
| MT-ATP6 | Mitochondrially Encoded ATP Synthase Membrane Subunit 6 | GCMTP008531 |
| HNRNPR | Heterogeneous Nuclear Ribonucleoprotein R | GC01M023303 |
| PIK3R5 | Phosphoinositide-3-Kinase Regulatory Subunit 5 | GC17M008878 |
| SSBP2 | Single Stranded DNA Binding Protein 2 | GC05M081413 |
| PDIA4 | Protein Disulfide Isomerase Family A Member 4 | GC07M149003 |
| PFAS | Phosphoribosylformylglycinamidine Synthase | GC17P008247 |
| TRO | Trophinin | GC0XP054920 |
| TRIM14 | Tripartite Motif Containing 14 | GC09M098035 |
| HLA-DMB | Major Histocompatibility Complex, Class II, DM Beta | GC06M032934 |
| HLA-DOA | Major Histocompatibility Complex, Class II, DO Alpha | GC06M033004 |
| HLA-DOB | Major Histocompatibility Complex, Class II, DO Beta | GC06M032814 |
| HCG23 | HLA Complex Group 23 | GC06P033440 |
| HLA-DQB1-AS1 | HLA-DQB1 Antisense RNA 1 | GC06P032659 |
| ARHGEF2 | Rho/Rac Guanine Nucleotide Exchange Factor 2 | GC01M155946 |
| TRIM39 | Tripartite Motif Containing 39 | GC06P033230 |
| EGFL8 | EGF Like Domain Multiple 8 | GC06P033437 |
| MIR942 | MicroRNA 942 | GC01P117094 |
| HOXD9 | Homeobox D9 | GC02P176122 |
| IL9 | Interleukin 9 | GC05M135891 |
| CYYR1 | Cysteine And Tyrosine Rich 1 | GC21M026466 |
| UCP1 | Uncoupling Protein 1 | GC04M140559 |
| UGT1A10 | UDP Glucuronosyltransferase Family 1 Member A10 | GC02P233636 |
| LPIN1 | Lipin 1 | GC02P011649 |
| FOXP4 | Forkhead Box P4 | GC06P046098 |
| NOLC1 | Nucleolar And Coiled-Body Phosphoprotein 1 | GC10P102152 |
| CHD1 | Chromodomain Helicase DNA Binding Protein 1 | GC05M098853 |
| RPL35A | Ribosomal Protein L35a | GC03P197949 |
| PDK2 | Pyruvate Dehydrogenase Kinase 2 | GC17P050095 |
| RPL24 | Ribosomal Protein L24 | GC03M101681 |
| ZNF207 | Zinc Finger Protein 207 | GC17P032358 |
| FBXO8 | F-Box Protein 8 | GC04M174236 |
| GCNT3 | Glucosaminyl (N-Acetyl) Transferase 3, Mucin Type | GC15P059594 |
| CD209 | CD209 Molecule | GC19M007739 |
| ATF6B | Activating Transcription Factor 6 Beta | GC06M032115 |
| PIK3C3 | Phosphatidylinositol 3-Kinase Catalytic Subunit Type 3 | GC18P041955 |
| SRSF5 | Serine And Arginine Rich Splicing Factor 5 | GC14P069727 |
| ANAPC11 | Anaphase Promoting Complex Subunit 11 | GC17P081890 |
| AGA | Aspartylglucosaminidase | GC04M177430 |
| M6PR | Mannose-6-Phosphate Receptor, Cation Dependent | GC12M008951 |
| NRF1 | Nuclear Respiratory Factor 1 | GC07P129611 |
| H3C14 | H3 Clustered Histone 14 | GC01M149917 |
| SHC2 | SHC Adaptor Protein 2 | GC19M000420 |
| NKD2 | NKD Inhibitor Of WNT Signaling Pathway 2 | GC05P000993 |
| HES5 | Hes Family BHLH Transcription Factor 5 | GC01M002528 |
| CAPRIN1 | Cell Cycle Associated Protein 1 | GC11P034051 |
| FKBP5 | FKBP Prolyl Isomerase 5 | GC06M041289 |
| MSH5 | MutS Homolog 5 | GC06P033420 |
| USP21 | Ubiquitin Specific Peptidase 21 | GC01P161159 |
| RASAL2 | RAS Protein Activator Like 2 | GC01P178093 |
| TRIM3 | Tripartite Motif Containing 3 | GC11M006450 |
| P2RY11 | Purinergic Receptor P2Y11 | GC19P010146 |
| GALNT2 | Polypeptide N-Acetylgalactosaminyltransferase 2 | GC01P230057 |
| GSC | Goosecoid Homeobox | GC14M094768 |
| EGR3 | Early Growth Response 3 | GC08M022687 |
| PTTG1IP | PTTG1 Interacting Protein | GC21M044849 |
| SPINK6 | Serine Peptidase Inhibitor Kazal Type 6 | GC05P148202 |
| LNPEP | Leucyl And Cystinyl Aminopeptidase | GC05P096935 |
| SDCBP | Syndecan Binding Protein | GC08P058539 |
| CCNDBP1 | Cyclin D1 Binding Protein 1 | GC15P043185 |
| BIK | BCL2 Interacting Killer | GC22P043110 |
| ASPM | Abnormal Spindle Microtubule Assembly | GC01M197084 |
| RNF14 | Ring Finger Protein 14 | GC05P141958 |
| CAP2 | Cyclase Associated Actin Cytoskeleton Regulatory Protein 2 | GC06P017393 |
| MALT1 | MALT1 Paracaspase | GC18P058671 |
| USB1 | U6 SnRNA Biogenesis Phosphodiesterase 1 | GC16P057999 |
| PEPD | Peptidase D | GC19M033386 |
| LHX1 | LIM Homeobox 1 | GC17P036937 |
| BVES | Blood Vessel Epicardial Substance | GC06M105096 |
| RRAD | RRAD, Ras Related Glycolysis Inhibitor And Calcium Channel Regulator | GC16M066925 |
| GPX7 | Glutathione Peroxidase 7 | GC01P052602 |
| PCDH19 | Protocadherin 19 | GC0XM100291 |
| NCS1 | Neuronal Calcium Sensor 1 | GC09P130172 |
| CHST11 | Carbohydrate Sulfotransferase 11 | GC12P104455 |
| KRT28 | Keratin 28 | GC17M040792 |
| KRT73 | Keratin 73 | GC12M052607 |
| H2BC14 | H2B Clustered Histone 14 | GC06P028120 |
| DSCR8 | Down Syndrome Critical Region 8 | GC21P038121 |
| TRIM37 | Tripartite Motif Containing 37 | GC17M058982 |
| PRKAB2 | Protein Kinase AMP-Activated Non-Catalytic Subunit Beta 2 | GC01M147155 |
| SMARCD1 | SWI/SNF Related, Matrix Associated, Actin Dependent Regulator Of Chromatin, Subfamily D, Member 1 | GC12P050085 |
| MIR188 | MicroRNA 188 | GC0XP050003 |
| NR2F1 | Nuclear Receptor Subfamily 2 Group F Member 1 | GC05P093583 |
| CIDEC | Cell Death Inducing DFFA Like Effector C | GC03M009866 |
| MATN2 | Matrilin 2 | GC08P097868 |
| CDSN | Corneodesmosin | GC06M031115 |
| OTUB1 | OTU Deubiquitinase, Ubiquitin Aldehyde Binding 1 | GC11P063985 |
| IL10RB | Interleukin 10 Receptor Subunit Beta | GC21P033266 |
| EIF4A2 | Eukaryotic Translation Initiation Factor 4A2 | GC03P186783 |
| H3C15 | H3 Clustered Histone 15 | GC01P149907 |
| PSMD1 | Proteasome 26S Subunit, Non-ATPase 1 | GC02P231056 |
| TMEM88 | Transmembrane Protein 88 | GC17P007854 |
| CYP27A1 | Cytochrome P450 Family 27 Subfamily A Member 1 | GC02P218781 |
| TNFSF12 | TNF Superfamily Member 12 | GC17P007935 |
| SEC62-AS1 | SEC62 Antisense RNA 1 | GC03M169979 |
| PDZK1 | PDZ Domain Containing 1 | GC01M145670 |
| DUT | Deoxyuridine Triphosphatase | GC15P048331 |
| CSAD | Cysteine Sulfinic Acid Decarboxylase | GC12M053160 |
| EOMES | Eomesodermin | GC03M027715 |
| CFAP20 | Cilia And Flagella Associated Protein 20 | GC16M058113 |
| NXF1 | Nuclear RNA Export Factor 1 | GC11M063082 |
| PGAM5 | PGAM Family Member 5, Mitochondrial Serine/Threonine Protein Phosphatase | GC12P132710 |
| KIF23 | Kinesin Family Member 23 | GC15P069414 |
| MIR519A1 | MicroRNA 519a-1 | GC19P053752 |
| PGLYRP2 | Peptidoglycan Recognition Protein 2 | GC19M015468 |
| SHROOM3 | Shroom Family Member 3 | GC04P076435 |
| LY9 | Lymphocyte Antigen 9 | GC01P160796 |
| MIA3 | MIA SH3 Domain ER Export Factor 3 | GC01P222618 |
| GSTT2B | Glutathione S-Transferase Theta 2B (Gene/Pseudogene) | GC22M023957 |
| B3GALT4 | Beta-1,3-Galactosyltransferase 4 | GC06P033277 |
| ATG16L1 | Autophagy Related 16 Like 1 | GC02P233215 |
| GRAP2 | GRB2 Related Adaptor Protein 2 | GC22P039901 |
| CDHR2 | Cadherin Related Family Member 2 | GC05P176542 |
| ANKHD1 | Ankyrin Repeat And KH Domain Containing 1 | GC05P142823 |
| LSM2 | LSM2 Homolog, U6 Small Nuclear RNA And MRNA Degradation Associated | GC06M032380 |
| ABCF1 | ATP Binding Cassette Subfamily F Member 1 | GC06P030571 |
| GNA13 | G Protein Subunit Alpha 13 | GC17M065009 |
| HMGB2 | High Mobility Group Box 2 | GC04M173331 |
| AFAP1L2 | Actin Filament Associated Protein 1 Like 2 | GC10M114281 |
| TUBB4A | Tubulin Beta 4A Class IVa | GC19M006496 |
| PHF2 | PHD Finger Protein 2 | GC09P093576 |
| VPS4B | Vacuolar Protein Sorting 4 Homolog B | GC18M063389 |
| NCAPH | Non-SMC Condensin I Complex Subunit H | GC02P096365 |
| KARS1 | Lysyl-TRNA Synthetase 1 | GC16M075628 |
| SMARCC2 | SWI/SNF Related, Matrix Associated, Actin Dependent Regulator Of Chromatin Subfamily C Member 2 | GC12M056161 |
| CALU | Calumenin | GC07P128739 |
| SORBS2 | Sorbin And SH3 Domain Containing 2 | GC04M185585 |
| PRKCG | Protein Kinase C Gamma | GC19P053879 |
| PROC | Protein C, Inactivator Of Coagulation Factors Va And VIIIa | GC02P127418 |
| TM4SF5 | Transmembrane 4 L Six Family Member 5 | GC17P004771 |
| TBXAS1 | Thromboxane A Synthase 1 | GC07P139777 |
| RPL3 | Ribosomal Protein L3 | GC22M044553 |
| C1D | C1D Nuclear Receptor Corepressor | GC02M068041 |
| HMGCS2 | 3-Hydroxy-3-Methylglutaryl-CoA Synthase 2 | GC01M119747 |
| IL10RA | Interleukin 10 Receptor Subunit Alpha | GC11P117987 |
| EPB41 | Erythrocyte Membrane Protein Band 4.1 | GC01P028887 |
| MYH14 | Myosin Heavy Chain 14 | GC19P050192 |
| HSPA12A | Heat Shock Protein Family A (Hsp70) Member 12A | GC10M116671 |
| CFB | Complement Factor B | GC06P031945 |
| DHX16 | DEAH-Box Helicase 16 | GC06M030653 |
| LY6G5B | Lymphocyte Antigen 6 Family Member G5B | GC06P033407 |
| ENSG00000272221 |  | GC06M031476 |
| TRIP6 | Thyroid Hormone Receptor Interactor 6 | GC07P100867 |
| WNT9B | Wnt Family Member 9B | GC17P046833 |
| SSX4 | SSX Family Member 4 | GC0XP048383 |
| HRH3 | Histamine Receptor H3 | GC20M062214 |
| HSD11B1 | Hydroxysteroid 11-Beta Dehydrogenase 1 | GC01P209686 |
| EEF2K | Eukaryotic Elongation Factor 2 Kinase | GC16P022217 |
| IPO4 | Importin 4 | GC14M024181 |
| IPO9 | Importin 9 | GC01P201829 |
| TNFSF14 | TNF Superfamily Member 14 | GC19M006663 |
| CA3 | Carbonic Anhydrase 3 | GC08P085373 |
| SLC22A7 | Solute Carrier Family 22 Member 7 | GC06P046118 |
| RFC3 | Replication Factor C Subunit 3 | GC13P033818 |
| FPR2 | Formyl Peptide Receptor 2 | GC19P051752 |
| IGHG1 | Immunoglobulin Heavy Constant Gamma 1 (G1m Marker) | GC14M105736 |
| UTP4 | UTP4 Small Subunit Processome Component | GC16P069132 |
| PPM1B | Protein Phosphatase, Mg2+/Mn2+ Dependent 1B | GC02P044167 |
| ANOS1 | Anosmin 1 | GC0XM008528 |
| PSMD14 | Proteasome 26S Subunit, Non-ATPase 14 | GC02P161308 |
| COPA | COPI Coat Complex Subunit Alpha | GC01M160288 |
| MYL2 | Myosin Light Chain 2 | GC12M110910 |
| GORASP1 | Golgi Reassembly Stacking Protein 1 | GC03M039096 |
| CUL5 | Cullin 5 | GC11P108008 |
| GLDC | Glycine Decarboxylase | GC09M006522 |
| DHRS2 | Dehydrogenase/Reductase 2 | GC14P025017 |
| TFPT | TCF3 Fusion Partner | GC19M054107 |
| GCSH | Glycine Cleavage System Protein H | GC16M081081 |
| MAP4K2 | Mitogen-Activated Protein Kinase Kinase Kinase Kinase 2 | GC11M064786 |
| PSMC2 | Proteasome 26S Subunit, ATPase 2 | GC07P103344 |
| HLA-S | Major Histocompatibility Complex, Class I, S (Pseudogene) | GC06M031381 |
| S1PR2 | Sphingosine-1-Phosphate Receptor 2 | GC19M010223 |
| CALM2 | Calmodulin 2 | GC02M047124 |
| GFPT1 | Glutamine--Fructose-6-Phosphate Transaminase 1 | GC02M069283 |
| PKD2 | Polycystin 2, Transient Receptor Potential Cation Channel | GC04P088007 |
| ACVRL1 | Activin A Receptor Like Type 1 | GC12P051906 |
| RPGRIP1L | RPGRIP1 Like | GC16M053597 |
| MNT | MAX Network Transcriptional Repressor | GC17M002384 |
| MIR645 | MicroRNA 645 | GC20P050585 |
| FCN2 | Ficolin 2 | GC09P134864 |
| KIF3B | Kinesin Family Member 3B | GC20P032277 |
| SHC4 | SHC Adaptor Protein 4 | GC15M048823 |
| GPC5 | Glypican 5 | GC13P091398 |
| RBBP5 | RB Binding Protein 5, Histone Lysine Methyltransferase Complex Subunit | GC01M205055 |
| IL1RL1 | Interleukin 1 Receptor Like 1 | GC02P102294 |
| CLVS1 | Clavesin 1 | GC08P060966 |
| SLC1A4 | Solute Carrier Family 1 Member 4 | GC02P064988 |
| POLR2C | RNA Polymerase II Subunit C | GC16P057462 |
| S100A16 | S100 Calcium Binding Protein A16 | GC01M153606 |
| CNPY2 | Canopy FGF Signaling Regulator 2 | GC12M056309 |
| CAVIN2 | Caveolae Associated Protein 2 | GC02M191835 |
| MIR758 | MicroRNA 758 | GC14P104484 |
| PHKG2 | Phosphorylase Kinase Catalytic Subunit Gamma 2 | GC16P030749 |
| ACVR2B | Activin A Receptor Type 2B | GC03P038453 |
| LOC112679202 | ABO Promoter Region | GC09U903068 |
| MERTK | MER Proto-Oncogene, Tyrosine Kinase | GC02P111898 |
| RUBCN | Rubicon Autophagy Regulator | GC03M197673 |
| CABIN1 | Calcineurin Binding Protein 1 | GC22P024011 |
| KDM8 | Lysine Demethylase 8 | GC16P027214 |
| UGT1A4 | UDP Glucuronosyltransferase Family 1 Member A4 | GC02P233718 |
| TNFAIP8L1 | TNF Alpha Induced Protein 8 Like 1 | GC19P004639 |
| CAMKK2 | Calcium/Calmodulin Dependent Protein Kinase Kinase 2 | GC12M121276 |
| ULK2 | Unc-51 Like Autophagy Activating Kinase 2 | GC17M019841 |
| UNC5C | Unc-5 Netrin Receptor C | GC04M095162 |
| ICOS | Inducible T Cell Costimulator | GC02P203937 |
| CAVIN1 | Caveolae Associated Protein 1 | GC17M042404 |
| CARM1 | Coactivator Associated Arginine Methyltransferase 1 | GC19P010871 |
| CCL8 | C-C Motif Chemokine Ligand 8 | GC17P034319 |
| THOC5 | THO Complex 5 | GC22M029505 |
| OIP5 | Opa Interacting Protein 5 | GC15M041309 |
| SERPINE2 | Serpin Family E Member 2 | GC02M223975 |
| MIR509-3 | MicroRNA 509-3 | GC0XM147259 |
| WWP2 | WW Domain Containing E3 Ubiquitin Protein Ligase 2 | GC16P069796 |
| MOGS | Mannosyl-Oligosaccharide Glucosidase | GC02M074461 |
| EIF4G2 | Eukaryotic Translation Initiation Factor 4 Gamma 2 | GC11M010856 |
| OPRK1 | Opioid Receptor Kappa 1 | GC08M053227 |
| RPN1 | Ribophorin I | GC03M128619 |
| IL17RB | Interleukin 17 Receptor B | GC03P053855 |
| MIR520F | MicroRNA 520f | GC19P053682 |
| SMARCD3 | SWI/SNF Related, Matrix Associated, Actin Dependent Regulator Of Chromatin, Subfamily D, Member 3 | GC07M151238 |
| TSN | Translin | GC02P121737 |
| RNASE4 | Ribonuclease A Family Member 4 | GC14P020812 |
| SNRPG | Small Nuclear Ribonucleoprotein Polypeptide G | GC02M070281 |
| SLC2A4RG | SLC2A4 Regulator | GC20P063739 |
| ARHGAP42 | Rho GTPase Activating Protein 42 | GC11P100687 |
| TMEM99 | Transmembrane Protein 99 (Putative) | GC17P040819 |
| PROX1-AS1 | PROX1 Antisense RNA 1 | GC01M213817 |
| LINC02484 | Long Intergenic Non-Protein Coding RNA 2484 | GC04M034121 |
| DGKQ | Diacylglycerol Kinase Theta | GC04M000942 |
| ARPP19 | CAMP Regulated Phosphoprotein 19 | GC15M052547 |
| MIR1275 | MicroRNA 1275 | GC06M041264 |
| MIR329-1 | MicroRNA 329-1 | GC14P104444 |
| SERPINA11 | Serpin Family A Member 11 | GC14M094442 |
| TRIM16 | Tripartite Motif Containing 16 | GC17M015627 |
| TSPYL2 | TSPY Like 2 | GC0XP053082 |
| MIR153-1 | MicroRNA 153-1 | GC02M219294 |
| CFH | Complement Factor H | GC01P196621 |
| ACACB | Acetyl-CoA Carboxylase Beta | GC12P109116 |
| CKM | Creatine Kinase, M-Type | GC19M045306 |
| RAD18 | RAD18 E3 Ubiquitin Protein Ligase | GC03M008850 |
| H2BC3 | H2B Clustered Histone 3 | GC06M026044 |
| TRIM22 | Tripartite Motif Containing 22 | GC11P005689 |
| SKIV2L | Ski2 Like RNA Helicase | GC06P031958 |
| FKBPL | FKBP Prolyl Isomerase Like | GC06M032401 |
| MPP3 | Membrane Palmitoylated Protein 3 | GC17M043800 |
| USP16 | Ubiquitin Specific Peptidase 16 | GC21P029024 |
| NAV3 | Neuron Navigator 3 | GC12P077326 |
| KCNN3 | Potassium Calcium-Activated Channel Subfamily N Member 3 | GC01M154697 |
| SMAD5 | SMAD Family Member 5 | GC05P136132 |
| IL34 | Interleukin 34 | GC16P070613 |
| FAM160B2 | Family With Sequence Similarity 160 Member B2 | GC08P022089 |
| PLAA | Phospholipase A2 Activating Protein | GC09M026903 |
| PMPCA | Peptidase, Mitochondrial Processing Subunit Alpha | GC09P136410 |
| HSD17B8 | Hydroxysteroid 17-Beta Dehydrogenase 8 | GC06P033497 |
| NPY1R | Neuropeptide Y Receptor Y1 | GC04M163323 |
| CDK11B | Cyclin Dependent Kinase 11B | GC01M001705 |
| RAPGEF1 | Rap Guanine Nucleotide Exchange Factor 1 | GC09M131576 |
| KMT2A | Lysine Methyltransferase 2A | GC11P118436 |
| CHAF1B | Chromatin Assembly Factor 1 Subunit B | GC21P036385 |
| PPT2 | Palmitoyl-Protein Thioesterase 2 | GC06P032153 |
| NRM | Nurim | GC06M030790 |
| PSORS1C2 | Psoriasis Susceptibility 1 Candidate 2 | GC06M031137 |
| C6orf47 | Chromosome 6 Open Reading Frame 47 | GC06M032371 |
| HCG22 | HLA Complex Group 22 (Gene/Pseudogene) | GC06P031053 |
| RPL3P2 | Ribosomal Protein L3 Pseudogene 2 | GC06P031280 |
| ENSG00000271581 |  | GC06P033383 |
| TXN2 | Thioredoxin 2 | GC22M036467 |
| TRIO | Trio Rho Guanine Nucleotide Exchange Factor | GC05P014143 |
| GRAP | GRB2 Related Adaptor Protein | GC17M019481 |
| DNAJA1 | DnaJ Heat Shock Protein Family (Hsp40) Member A1 | GC09P033025 |
| NLRX1 | NLR Family Member X1 | GC11P119166 |
| CDC14B | Cell Division Cycle 14B | GC09M096490 |
| TLN2 | Talin 2 | GC15P062390 |
| DLG5 | Discs Large MAGUK Scaffold Protein 5 | GC10M077790 |
| MACF1 | Microtubule Actin Crosslinking Factor 1 | GC01P039082 |
| ZMYND8 | Zinc Finger MYND-Type Containing 8 | GC20M047209 |
| MYCBP2 | MYC Binding Protein 2 | GC13M077044 |
| SVEP1 | Sushi, Von Willebrand Factor Type A, EGF And Pentraxin Domain Containing 1 | GC09M110365 |
| DBI | Diazepam Binding Inhibitor, Acyl-CoA Binding Protein | GC02P119366 |
| UBA52 | Ubiquitin A-52 Residue Ribosomal Protein Fusion Product 1 | GC19P018563 |
| TFDP2 | Transcription Factor Dp-2 | GC03M141944 |
| KNL1 | Kinetochore Scaffold 1 | GC15P040595 |
| MXD1 | MAX Dimerization Protein 1 | GC02P069897 |
| YRDC | YrdC N6-Threonylcarbamoyltransferase Domain Containing | GC01M037802 |
| EIF1 | Eukaryotic Translation Initiation Factor 1 | GC17P041688 |
| MAD2L1BP | MAD2L1 Binding Protein | GC06P043629 |
| PSMC6 | Proteasome 26S Subunit, ATPase 6 | GC14P052707 |
| ATXN7 | Ataxin 7 | GC03P063864 |
| H2BC5 | H2B Clustered Histone 5 | GC06P028382 |
| H2BC15 | H2B Clustered Histone 15 | GC06P028140 |
| DIAPH3 | Diaphanous Related Formin 3 | GC13M059665 |
| TXLNA | Taxilin Alpha | GC01P032179 |
| MT-ND3 | Mitochondrially Encoded NADH:Ubiquinone Oxidoreductase Core Subunit 3 | GCMTP010061 |
| RPS3 | Ribosomal Protein S3 | GC11P075719 |
| FBXO7 | F-Box Protein 7 | GC22P032474 |
| HNRNPH2 | Heterogeneous Nuclear Ribonucleoprotein H2 | GC0XP101408 |
| IKZF1 | IKAROS Family Zinc Finger 1 | GC07P050343 |
| UBE2D2 | Ubiquitin Conjugating Enzyme E2 D2 | GC05P139526 |
| LUM | Lumican | GC12M091102 |
| ITPR1 | Inositol 1,4,5-Trisphosphate Receptor Type 1 | GC03P004486 |
| HSD17B10 | Hydroxysteroid 17-Beta Dehydrogenase 10 | GC0XM053431 |
| MATR3 | Matrin 3 | GC05P139274 |
| SRPX2 | Sushi Repeat Containing Protein X-Linked 2 | GC0XP100648 |
| GALNT4 | Polypeptide N-Acetylgalactosaminyltransferase 4 | GC12M089520 |
| CKAP2 | Cytoskeleton Associated Protein 2 | GC13P052455 |
| ASCL2 | Achaete-Scute Family BHLH Transcription Factor 2 | GC11M002289 |
| ZBTB17 | Zinc Finger And BTB Domain Containing 17 | GC01M015943 |
| ST3GAL5 | ST3 Beta-Galactoside Alpha-2,3-Sialyltransferase 5 | GC02M085839 |
| C7 | Complement C7 | GC05P040909 |
| NEIL3 | Nei Like DNA Glycosylase 3 | GC04P177309 |
| ZHX1 | Zinc Fingers And Homeoboxes 1 | GC08M123248 |
| A1CF | APOBEC1 Complementation Factor | GC10M050799 |
| AGPS | Alkylglycerone Phosphate Synthase | GC02P177392 |
| TENT5C | Terminal Nucleotidyltransferase 5C | GC01P117606 |
| SNTA1 | Syntrophin Alpha 1 | GC20M033407 |
| MSRA | Methionine Sulfoxide Reductase A | GC08P010054 |
| RFC4 | Replication Factor C Subunit 4 | GC03M186789 |
| NAA11 | N-Alpha-Acetyltransferase 11, NatA Catalytic Subunit | GC04M079225 |
| H2BC13 | H2B Clustered Histone 13 | GC06M027810 |
| MEFV | MEFV Innate Immuity Regulator, Pyrin | GC16M003259 |
| PSMD5 | Proteasome 26S Subunit, Non-ATPase 5 | GC09M120815 |
| NLRC3 | NLR Family CARD Domain Containing 3 | GC16M003539 |
| SPDYA | Speedy/RINGO Cell Cycle Regulator Family Member A | GC02P028782 |
| ZNF32 | Zinc Finger Protein 32 | GC10M043643 |
| H6PD | Hexose-6-Phosphate Dehydrogenase/Glucose 1-Dehydrogenase | GC01P009234 |
| AGXT | Alanine--Glyoxylate And Serine--Pyruvate Aminotransferase | GC02P240868 |
| TARDBP | TAR DNA Binding Protein | GC01P011013 |
| PAFAH1B1 | Platelet Activating Factor Acetylhydrolase 1b Regulatory Subunit 1 | GC17P002593 |
| NAE1 | NEDD8 Activating Enzyme E1 Subunit 1 | GC16M066803 |
| RND1 | Rho Family GTPase 1 | GC12M048857 |
| MIR33B | MicroRNA 33b | GC17M017813 |
| MIR208A | MicroRNA 208a | GC14M023388 |
| MIR604 | MicroRNA 604 | GC10M029545 |
| CHD3 | Chromodomain Helicase DNA Binding Protein 3 | GC17P007966 |
| RAB21 | RAB21, Member RAS Oncogene Family | GC12P071754 |
| FSTL3 | Follistatin Like 3 | GC19P000676 |
| KCNH2 | Potassium Voltage-Gated Channel Subfamily H Member 2 | GC07M150944 |
| INHBB | Inhibin Subunit Beta B | GC02P120347 |
| EPS15 | Epidermal Growth Factor Receptor Pathway Substrate 15 | GC01M051354 |
| KIR2DS2 | Killer Cell Immunoglobulin Like Receptor, Two Ig Domains And Short Cytoplasmic Tail 2 | GC19MR00122 |
| GFI1 | Growth Factor Independent 1 Transcriptional Repressor | GC01M092474 |
| NINJ1 | Ninjurin 1 | GC09M093121 |
| TBX21 | T-Box Transcription Factor 21 | GC17P047733 |
| SLC25A3 | Solute Carrier Family 25 Member 3 | GC12P098593 |
| DGKA | Diacylglycerol Kinase Alpha | GC12P055927 |
| PSMA1 | Proteasome 20S Subunit Alpha 1 | GC11M014505 |
| ITPKA | Inositol-Trisphosphate 3-Kinase A | GC15P041493 |
| RCN2 | Reticulocalbin 2 | GC15P076931 |
| GMPR2 | Guanosine Monophosphate Reductase 2 | GC14P024232 |
| TOLLIP | Toll Interacting Protein | GC11M001274 |
| SPARCL1 | SPARC Like 1 | GC04M087473 |
| NEDD4L | NEDD4 Like E3 Ubiquitin Protein Ligase | GC18P058044 |
| IFN1@ | Interferon, Type 1, Cluster | GC09U990039 |
| PDE4A | Phosphodiesterase 4A | GC19P010416 |
| OXCT1 | 3-Oxoacid CoA-Transferase 1 | GC05M041732 |
| PRPF3 | Pre-MRNA Processing Factor 3 | GC01P150321 |
| DPF3 | Double PHD Fingers 3 | GC14M072616 |
| UBTF | Upstream Binding Transcription Factor | GC17M044205 |
| TRIM39-RPP21 | TRIM39-RPP21 Readthrough | GC06P033233 |
| BLVRA | Biliverdin Reductase A | GC07P043758 |
| PDK4 | Pyruvate Dehydrogenase Kinase 4 | GC07M095583 |
| PCBD1 | Pterin-4 Alpha-Carbinolamine Dehydratase 1 | GC10M070882 |
| ZNF667 | Zinc Finger Protein 667 | GC19M056439 |
| CABYR | Calcium Binding Tyrosine Phosphorylation Regulated | GC18P024138 |
| PRKRA | Protein Activator Of Interferon Induced Protein Kinase EIF2AK2 | GC02M178431 |
| TARBP1 | TAR (HIV-1) RNA Binding Protein 1 | GC01M234391 |
| JTB | Jumping Translocation Breakpoint | GC01M153974 |
| SEPTIN11 | Septin 11 | GC04P076950 |
| IMMT | Inner Membrane Mitochondrial Protein | GC02M086144 |
| LBP | Lipopolysaccharide Binding Protein | GC20P038346 |
| HOXB-AS3 | HOXB Cluster Antisense RNA 3 | GC17P048549 |
| RBPMS | RNA Binding Protein, MRNA Processing Factor | GC08P030361 |
| CD244 | CD244 Molecule | GC01M160830 |
| IFIT1 | Interferon Induced Protein With Tetratricopeptide Repeats 1 | GC10P089396 |
| ISG20 | Interferon Stimulated Exonuclease Gene 20 | GC15P088635 |
| PRICKLE4 | Prickle Planar Cell Polarity Protein 4 | GC06P041780 |
| CCN5 | Cellular Communication Network Factor 5 | GC20P044715 |
| SLC7A2 | Solute Carrier Family 7 Member 2 | GC08P017497 |
| JARID2 | Jumonji And AT-Rich Interaction Domain Containing 2 | GC06P015246 |
| POU3F1 | POU Class 3 Homeobox 1 | GC01M038044 |
| CCL23 | C-C Motif Chemokine Ligand 23 | GC17M036013 |
| RAB34 | RAB34, Member RAS Oncogene Family | GC17M028714 |
| LOC110599567 | D7S22 Minisatellite Repeat Instability Region | GC07U904755 |
| ARNTL | Aryl Hydrocarbon Receptor Nuclear Translocator Like | GC11P013276 |
| ZNF23 | Zinc Finger Protein 23 | GC16M071468 |
| SLC1A2 | Solute Carrier Family 1 Member 2 | GC11M035272 |
| MIR1247 | MicroRNA 1247 | GC14M101560 |
| CIAO2A | Cytosolic Iron-Sulfur Assembly Component 2A | GC15M064546 |
| MAPK13 | Mitogen-Activated Protein Kinase 13 | GC06P046048 |
| ATP1A3 | ATPase Na+/K+ Transporting Subunit Alpha 3 | GC19M041966 |
| DHCR7 | 7-Dehydrocholesterol Reductase | GC11M071428 |
| RPS10 | Ribosomal Protein S10 | GC06M041274 |
| PAPSS1 | 3'-Phosphoadenosine 5'-Phosphosulfate Synthase 1 | GC04M107590 |
| DYNC1I1 | Dynein Cytoplasmic 1 Intermediate Chain 1 | GC07P095772 |
| BTLA | B And T Lymphocyte Associated | GC03M112463 |
| CKMT1B | Creatine Kinase, Mitochondrial 1B | GC15P043593 |
| ATAD3A | ATPase Family AAA Domain Containing 3A | GC01P001514 |
| EPB41L5 | Erythrocyte Membrane Protein Band 4.1 Like 5 | GC02P120013 |
| MIR646 | MicroRNA 646 | GC20P060308 |
| GLRX3 | Glutaredoxin 3 | GC10P130136 |
| CCDC137 | Coiled-Coil Domain Containing 137 | GC17P081666 |
| LILRB2 | Leukocyte Immunoglobulin Like Receptor B2 | GC19M054393 |
| KPNA1 | Karyopherin Subunit Alpha 1 | GC03M122421 |
| PSMD3 | Proteasome 26S Subunit, Non-ATPase 3 | GC17P039980 |
| TIAM2 | TIAM Rac1 Associated GEF 2 | GC06P154832 |
| DRAM1 | DNA Damage Regulated Autophagy Modulator 1 | GC12P101877 |
| H2BC12 | H2B Clustered Histone 12 | GC06M027150 |
| SGK2 | Serum/Glucocorticoid Regulated Kinase 2 | GC20P043558 |
| FABP2 | Fatty Acid Binding Protein 2 | GC04M119317 |
| TOMM34 | Translocase Of Outer Mitochondrial Membrane 34 | GC20M044942 |
| CTSZ | Cathepsin Z | GC20M058995 |
| RNF20 | Ring Finger Protein 20 | GC09P101533 |
| TNR | Tenascin R | GC01M175291 |
| GDF2 | Growth Differentiation Factor 2 | GC10P047322 |
| USP5 | Ubiquitin Specific Peptidase 5 | GC12P007850 |
| MIR761 | MicroRNA 761 | GC01M051836 |
| ACTL6B | Actin Like 6B | GC07M100643 |
| PPP1R10 | Protein Phosphatase 1 Regulatory Subunit 10 | GC06M030600 |
| TP53AIP1 | Tumor Protein P53 Regulated Apoptosis Inducing Protein 1 | GC11M128934 |
| MIR325 | MicroRNA 325 | GC0XM077005 |
| SLU7 | SLU7 Homolog, Splicing Factor | GC05M160401 |
| SERPINB9 | Serpin Family B Member 9 | GC06M002887 |
| HDAC7 | Histone Deacetylase 7 | GC12M047782 |
| TPP1 | Tripeptidyl Peptidase 1 | GC11M006614 |
| NMI | N-Myc And STAT Interactor | GC02M151270 |
| MIR466 | MicroRNA 466 | GC03M031161 |
| SLCO1A2 | Solute Carrier Organic Anion Transporter Family Member 1A2 | GC12M021264 |
| HSD17B2 | Hydroxysteroid 17-Beta Dehydrogenase 2 | GC16P082068 |
| CDYL | Chromodomain Y Like | GC06P004706 |
| GPATCH2 | G-Patch Domain Containing 2 | GC01M217426 |
| NUP62 | Nucleoporin 62 | GC19M049906 |
| DGAT2 | Diacylglycerol O-Acyltransferase 2 | GC11P075759 |
| PPP1R8 | Protein Phosphatase 1 Regulatory Subunit 8 | GC01P027830 |
| OSBPL1A | Oxysterol Binding Protein Like 1A | GC18M024162 |
| LOC111216273 | ADH1B Promoter | GC04U902751 |
| NUP88 | Nucleoporin 88 | GC17M005366 |
| DCAF13 | DDB1 And CUL4 Associated Factor 13 | GC08P103414 |
| SSX4B | SSX Family Member 4B | GC0XM048402 |
| SSX7 | SSX Family Member 7 | GC0XM052645 |
| TRIM32 | Tripartite Motif Containing 32 | GC09P116687 |
| CDC42BPA | CDC42 Binding Protein Kinase Alpha | GC01M226989 |
| SYF2 | SYF2 Pre-MRNA Splicing Factor | GC01M025222 |
| FUT5 | Fucosyltransferase 5 | GC19M005865 |
| PRDM1 | PR/SET Domain 1 | GC06P105993 |
| UGT2B4 | UDP Glucuronosyltransferase Family 2 Member B4 | GC04M069484 |
| ITGAM | Integrin Subunit Alpha M | GC16P031420 |
| DNAH2 | Dynein Axonemal Heavy Chain 2 | GC17P007717 |
| LINC02055 | Long Intergenic Non-Protein Coding RNA 2055 | GC08P135859 |
| ISG20L2 | Interferon Stimulated Exonuclease Gene 20 Like 2 | GC01M156723 |
| RPL13AP20 | Ribosomal Protein L13a Pseudogene 20 | GC12P012875 |
| PRRT1 | Proline Rich Transmembrane Protein 1 | GC06M032402 |
| SAPCD1 | Suppressor APC Domain Containing 1 | GC06P033423 |
| SNHG32 | Small Nucleolar RNA Host Gene 32 | GC06P046006 |
| PSORS1C3 | Psoriasis Susceptibility 1 Candidate 3 | GC06M031175 |
| HCG4 | HLA Complex Group 4 | GC06M030636 |
| HLA-K | Major Histocompatibility Complex, Class I, K (Pseudogene) | GC06P033209 |
| ENSG00000199332 |  | GC06M031482 |
| NONHSAG045982.2 |  | GC06M032630 |
| HSALNG0049430 |  | GC06M032641 |
| PBX3 | PBX Homeobox 3 | GC09P125747 |
| DCX | Doublecortin | GC0XM111293 |
| PRPF31 | Pre-MRNA Processing Factor 31 | GC19P054165 |
| GPAA1 | Glycosylphosphatidylinositol Anchor Attachment 1 | GC08P144082 |
| MYH1 | Myosin Heavy Chain 1 | GC17M010492 |
| FARSA | Phenylalanyl-TRNA Synthetase Subunit Alpha | GC19M012922 |
| CMBL | Carboxymethylenebutenolidase Homolog | GC05M010275 |
| RPP30 | Ribonuclease P/MRP Subunit P30 | GC10P090871 |
| IGDCC4 | Immunoglobulin Superfamily DCC Subclass Member 4 | GC15M065381 |
| DARS1 | Aspartyl-TRNA Synthetase 1 | GC02M135905 |
| ONECUT2 | One Cut Homeobox 2 | GC18P057436 |
| COP1 | COP1 E3 Ubiquitin Ligase | GC01M175944 |
| PASD1 | PAS Domain Containing Repressor 1 | GC0XP151563 |
| ODR4 | Odr-4 GPCR Localization Factor Homolog | GC01P186376 |
| SPINDOC | Spindlin Interactor And Repressor Of Chromatin Binding | GC11P063814 |
| MIR3928 | MicroRNA 3928 | GC22M031160 |
| DUSP14 | Dual Specificity Phosphatase 14 | GC17P037489 |
| H2BC17 | H2B Clustered Histone 17 | GC06P028351 |
| MIR770 | MicroRNA 770 | GC14P104485 |
| VSIG4 | V-Set And Immunoglobulin Domain Containing 4 | GC0XM066021 |
| LITAF | Lipopolysaccharide Induced TNF Factor | GC16M011547 |
| UTRN | Utrophin | GC06P144285 |
| MIR3619 | MicroRNA 3619 | GC22P046091 |
| AMER1 | APC Membrane Recruitment Protein 1 | GC0XM064185 |
| EFNB3 | Ephrin B3 | GC17P007953 |
| GCLM | Glutamate-Cysteine Ligase Modifier Subunit | GC01M093885 |
| SHCBP1 | SHC Binding And Spindle Associated 1 | GC16M046578 |
| UTP14A | UTP14A Small Subunit Processome Component | GC0XP129906 |
| IL31 | Interleukin 31 | GC12M122173 |
| GINS3 | GINS Complex Subunit 3 | GC16P058328 |
| MIR671 | MicroRNA 671 | GC07P151238 |
| IDO2 | Indoleamine 2,3-Dioxygenase 2 | GC08P039935 |
| DLX2 | Distal-Less Homeobox 2 | GC02M172099 |
| CYTH3 | Cytohesin 3 | GC07M006161 |
| EEF1D | Eukaryotic Translation Elongation Factor 1 Delta | GC08M143579 |
| SMARCD2 | SWI/SNF Related, Matrix Associated, Actin Dependent Regulator Of Chromatin, Subfamily D, Member 2 | GC17M063832 |
| GSTT2 | Glutathione S-Transferase Theta 2 (Gene/Pseudogene) | GC22P023980 |
| GORAB | Golgin, RAB6 Interacting | GC01P170501 |
| LOC111255642 | TNFRSF10B 5' Regulatory Region | GC08U902753 |
| PSIP1 | PC4 And SFRS1 Interacting Protein 1 | GC09M015456 |
| LSS | Lanosterol Synthase | GC21M047163 |
| PARP14 | Poly(ADP-Ribose) Polymerase Family Member 14 | GC03P122680 |
| CHD6 | Chromodomain Helicase DNA Binding Protein 6 | GC20M041402 |
| FBXW5 | F-Box And WD Repeat Domain Containing 5 | GC09M136941 |
| PBXIP1 | PBX Homeobox Interacting Protein 1 | GC01M154944 |
| SERPIND1 | Serpin Family D Member 1 | GC22P020810 |
| DUSP13 | Dual Specificity Phosphatase 13 | GC10M075094 |
| PLOD1 | Procollagen-Lysine,2-Oxoglutarate 5-Dioxygenase 1 | GC01P011934 |
| LIN7A | Lin-7 Homolog A, Crumbs Cell Polarity Complex Component | GC12M080792 |
| CASC3 | CASC3 Exon Junction Complex Subunit | GC17P040140 |
| HRG | Histidine Rich Glycoprotein | GC03P186660 |
| CHCHD2 | Coiled-Coil-Helix-Coiled-Coil-Helix Domain Containing 2 | GC07M056101 |
| AMH | Anti-Mullerian Hormone | GC19P002251 |
| SPC24 | SPC24 Component Of NDC80 Kinetochore Complex | GC19M011131 |
| ARHGAP9 | Rho GTPase Activating Protein 9 | GC12M057472 |
| XPO4 | Exportin 4 | GC13M020777 |
| GUCD1 | Guanylyl Cyclase Domain Containing 1 | GC22M027482 |
| CBR1 | Carbonyl Reductase 1 | GC21P036069 |
| POLE2 | DNA Polymerase Epsilon 2, Accessory Subunit | GC14M049643 |
| MAOB | Monoamine Oxidase B | GC0XM043766 |
| LOC111365141 | NOS2 5' Regulatory Region | GC17U902748 |
| AP2B1 | Adaptor Related Protein Complex 2 Subunit Beta 1 | GC17P035578 |
| ADCYAP1R1 | ADCYAP Receptor Type I | GC07P031058 |
| TUBB2B | Tubulin Beta 2B Class IIb | GC06M003224 |
| TUBB6 | Tubulin Beta 6 Class V | GC18P012307 |
| IQGAP3 | IQ Motif Containing GTPase Activating Protein 3 | GC01M156525 |
| UBE4A | Ubiquitination Factor E4A | GC11P118359 |
| CORO6 | Coronin 6 | GC17M029614 |
| SIVA1 | SIVA1 Apoptosis Inducing Factor | GC14P104753 |
| KDM4B | Lysine Demethylase 4B | GC19P004969 |
| HAPLN1 | Hyaluronan And Proteoglycan Link Protein 1 | GC05M083637 |
| ACTN3 | Actinin Alpha 3 (Gene/Pseudogene) | GC11P066546 |
| PARP12 | Poly(ADP-Ribose) Polymerase Family Member 12 | GC07M140023 |
| HIVEP2 | HIVEP Zinc Finger 2 | GC06M142751 |
| DDX47 | DEAD-Box Helicase 47 | GC12P012814 |
| HAO2 | Hydroxyacid Oxidase 2 | GC01P119368 |
| PUSL1 | Pseudouridine Synthase Like 1 | GC01P001308 |
| TNKS | Tankyrase | GC08P009555 |
| ITLN1 | Intelectin 1 | GC01M160876 |
| MRPS34 | Mitochondrial Ribosomal Protein S34 | GC16M001771 |
| FGG | Fibrinogen Gamma Chain | GC04M154604 |
| SERPING1 | Serpin Family G Member 1 | GC11P057597 |
| UBA1 | Ubiquitin Like Modifier Activating Enzyme 1 | GC0XP047190 |
| SNAP23 | Synaptosome Associated Protein 23 | GC15P042491 |
| MAP1S | Microtubule Associated Protein 1S | GC19P022236 |
| PTPN2 | Protein Tyrosine Phosphatase Non-Receptor Type 2 | GC18M016781 |
| PRPF4B | Pre-MRNA Processing Factor 4B | GC06P004021 |
| SYTL1 | Synaptotagmin Like 1 | GC01P027352 |
| ATG14 | Autophagy Related 14 | GC14M055366 |
| BCHE | Butyrylcholinesterase | GC03M165772 |
| INPP4B | Inositol Polyphosphate-4-Phosphatase Type II B | GC04M142023 |
| MEOX2 | Mesenchyme Homeobox 2 | GC07M015617 |
| H2BC1 | H2B Clustered Histone 1 | GC06P025845 |
| SSBP3 | Single Stranded DNA Binding Protein 3 | GC01M054225 |
| CEP57 | Centrosomal Protein 57 | GC11P095789 |
| TAB2 | TGF-Beta Activated Kinase 1 (MAP3K7) Binding Protein 2 | GC06P149218 |
| TMX3 | Thioredoxin Related Transmembrane Protein 3 | GC18M068673 |
| DSEL | Dermatan Sulfate Epimerase Like | GC18M067506 |
| RSU1 | Ras Suppressor Protein 1 | GC10M016672 |
| PRMT6 | Protein Arginine Methyltransferase 6 | GC01P107056 |
| PCBP4 | Poly(RC) Binding Protein 4 | GC03M051957 |
| PSMD11 | Proteasome 26S Subunit, Non-ATPase 11 | GC17P032444 |
| DUXAP10 | Double Homeobox A Pseudogene 10 | GC14M019293 |
| DDX20 | DEAD-Box Helicase 20 | GC01P111755 |
| IRF8 | Interferon Regulatory Factor 8 | GC16P085898 |
| INHBC | Inhibin Subunit Beta C | GC12P057434 |
| MIR548L | MicroRNA 548l | GC11M094466 |
| SLC39A7 | Solute Carrier Family 39 Member 7 | GC06P033200 |
| ADK | Adenosine Kinase | GC10P074152 |
| CDK5R2 | Cyclin Dependent Kinase 5 Regulatory Subunit 2 | GC02P218959 |
| ITGB7 | Integrin Subunit Beta 7 | GC12M053191 |
| BAG6 | BAG Cochaperone 6 | GC06M031639 |
| ANXA8L1 | Annexin A8 Like 1 | GC10P046375 |
| CIRBP | Cold Inducible RNA Binding Protein | GC19P001269 |
| HOXD8 | Homeobox D8 | GC02P176129 |
| IL25 | Interleukin 25 | GC14P025011 |
| DIXDC1 | DIX Domain Containing 1 | GC11P111927 |
| MIR760 | MicroRNA 760 | GC01P093846 |
| ADRB1 | Adrenoceptor Beta 1 | GC10P114044 |
| DYNC1H1 | Dynein Cytoplasmic 1 Heavy Chain 1 | GC14P104308 |
| GINS1 | GINS Complex Subunit 1 | GC20P025408 |
| LAPTM5 | Lysosomal Protein Transmembrane 5 | GC01M030732 |
| SUMF2 | Sulfatase Modifying Factor 2 | GC07P056140 |
| NAXE | NAD(P)HX Epimerase | GC01P156591 |
| IL26 | Interleukin 26 | GC12M068201 |
| GNAI3 | G Protein Subunit Alpha I3 | GC01P109548 |
| ROBO3 | Roundabout Guidance Receptor 3 | GC11P124865 |
| PSMB2 | Proteasome 20S Subunit Beta 2 | GC01M035599 |
| KRT80 | Keratin 80 | GC12M052168 |
| MAGEH1 | MAGE Family Member H1 | GC0XP055452 |
| B4GALNT1 | Beta-1,4-N-Acetyl-Galactosaminyltransferase 1 | GC12M057623 |
| LEFTY2 | Left-Right Determination Factor 2 | GC01M225937 |
| MGP | Matrix Gla Protein | GC12M014881 |
| GNPDA1 | Glucosamine-6-Phosphate Deaminase 1 | GC05M141991 |
| DNAH5 | Dynein Axonemal Heavy Chain 5 | GC05M013745 |
| MAFF | MAF BZIP Transcription Factor F | GC22P038200 |
| FMN1 | Formin 1 | GC15M032765 |
| SGSM3 | Small G Protein Signaling Modulator 3 | GC22P040370 |
| GIPC2 | GIPC PDZ Domain Containing Family Member 2 | GC01P077982 |
| TOB2 | Transducer Of ERBB2, 2 | GC22M041433 |
| UCKL1 | Uridine-Cytidine Kinase 1 Like 1 | GC20M063939 |
| TRPC4AP | Transient Receptor Potential Cation Channel Subfamily C Member 4 Associated Protein | GC20M035002 |
| CBX6 | Chromobox 6 | GC22M038861 |
| SAFB2 | Scaffold Attachment Factor B2 | GC19M005587 |
| BTG4 | BTG Anti-Proliferation Factor 4 | GC11M111385 |
| KCNRG | Potassium Channel Regulator | GC13P050015 |
| IER2 | Immediate Early Response 2 | GC19P013150 |
| HSF2BP | Heat Shock Transcription Factor 2 Binding Protein | GC21M043529 |
| CEP131 | Centrosomal Protein 131 | GC17M081189 |
| RTKN2 | Rhotekin 2 | GC10M062183 |
| CDYL2 | Chromodomain Y Like 2 | GC16M080598 |
| UBAP2 | Ubiquitin Associated Protein 2 | GC09M033921 |
| SPATA9 | Spermatogenesis Associated 9 | GC05M095652 |
| ZNF233 | Zinc Finger Protein 233 | GC19P044259 |
| ZNF26 | Zinc Finger Protein 26 | GC12P132986 |
| C1orf61 | Chromosome 1 Open Reading Frame 61 | GC01M156404 |
| MAP11 | Microtubule Associated Protein 11 | GC07M100257 |
| NXPE3 | Neurexophilin And PC-Esterase Domain Family Member 3 | GC03P101779 |
| CCDC178 | Coiled-Coil Domain Containing 178 | GC18M032937 |
| H2BC18 | H2B Clustered Histone 18 | GC01M149943 |
| LMCD1-AS1 | LMCD1 Antisense RNA 1 | GC03M007996 |
| TMEM92-AS1 | TMEM92 Antisense RNA 1 | GC17M050281 |
| PET117 | PET117 Cytochrome C Oxidase Chaperone | GC20P018119 |
| FTO | FTO Alpha-Ketoglutarate Dependent Dioxygenase | GC16P053737 |
| GPR37 | G Protein-Coupled Receptor 37 | GC07M124745 |
| CRY2 | Cryptochrome Circadian Regulator 2 | GC11P045960 |
| SYVN1 | Synoviolin 1 | GC11M065122 |
| TPTEP1 | TPTE Pseudogene 1 | GC22P016601 |
| NPPB | Natriuretic Peptide B | GC01M011858 |
| STRN | Striatin | GC02M036815 |
| MT1E | Metallothionein 1E | GC16P056625 |
| APOA2 | Apolipoprotein A2 | GC01M161222 |
| NECTIN1 | Nectin Cell Adhesion Molecule 1 | GC11M119624 |
| ARF6 | ADP Ribosylation Factor 6 | GC14P049895 |
| CSNK1A1L | Casein Kinase 1 Alpha 1 Like | GC13M037103 |
| TAF11 | TATA-Box Binding Protein Associated Factor 11 | GC06M041281 |
| GALNT1 | Polypeptide N-Acetylgalactosaminyltransferase 1 | GC18P035581 |
| HRNR | Hornerin | GC01M152184 |
| CTAG1A | Cancer/Testis Antigen 1A | GC0XP154585 |
| DYRK2 | Dual Specificity Tyrosine Phosphorylation Regulated Kinase 2 | GC12P067558 |
| OAS3 | 2'-5'-Oligoadenylate Synthetase 3 | GC12P112938 |
| UBR4 | Ubiquitin Protein Ligase E3 Component N-Recognin 4 | GC01M019074 |
| RPS19BP1 | Ribosomal Protein S19 Binding Protein 1 | GC22M044559 |
| MARVELD1 | MARVEL Domain Containing 1 | GC10P097713 |
| SYNGAP1 | Synaptic Ras GTPase Activating Protein 1 | GC06P033419 |
| HLA-J | Major Histocompatibility Complex, Class I, J (Pseudogene) | GC06P033213 |
| HCG25 | HLA Complex Group 25 | GC06P033523 |
| LINC02569 | Long Intergenic Non-Protein Coding RNA 2569 | GC06P033517 |
| RPL32P1 | Ribosomal Protein L32 Pseudogene 1 | GC06P033448 |
| BDH1 | 3-Hydroxybutyrate Dehydrogenase 1 | GC03M197521 |
| RPS26 | Ribosomal Protein S26 | GC12P056043 |
| KDELR2 | KDEL Endoplasmic Reticulum Protein Retention Receptor 2 | GC07M006447 |
| ACSM5 | Acyl-CoA Synthetase Medium Chain Family Member 5 | GC16P020420 |
| ANAPC13 | Anaphase Promoting Complex Subunit 13 | GC03M134477 |
| RAB24 | RAB24, Member RAS Oncogene Family | GC05M177301 |
| MS4A7 | Membrane Spanning 4-Domains A7 | GC11P060387 |
| GOLGA7 | Golgin A7 | GC08P041467 |
| ABHD3 | Abhydrolase Domain Containing 3 | GC18M021650 |
| UBL5 | Ubiquitin Like 5 | GC19P009827 |
| CDNF | Cerebral Dopamine Neurotrophic Factor | GC10M014771 |
| MED8 | Mediator Complex Subunit 8 | GC01M043383 |
| SLC22A25 | Solute Carrier Family 22 Member 25 | GC11M063163 |
| APOC4 | Apolipoprotein C4 | GC19P044943 |
| CDADC1 | Cytidine And DCMP Deaminase Domain Containing 1 | GC13P049247 |
| C12orf66 | Chromosome 12 Open Reading Frame 66 | GC12M064186 |
| CCDC158 | Coiled-Coil Domain Containing 158 | GC04M076312 |
| C2orf72 | Chromosome 2 Open Reading Frame 72 | GC02P231037 |
| CFAP221 | Cilia And Flagella Associated Protein 221 | GC02P119544 |
| C3P1 | Complement Component 3 Precursor Pseudogene | GC19P010037 |
| LRCOL1 | Leucine Rich Colipase Like 1 | GC12M132603 |
| REELD1 | Reeler Domain Containing 1 | GC04P146214 |
| APOC1P1 | Apolipoprotein C1 Pseudogene 1 | GC19P044926 |
| LOC100288798 | Uncharacterized LOC100288798 | GC12P046384 |
| TCEAL3-AS1 | TCEAL3 Antisense RNA 1 | GC0XM103626 |
| LOC101927269 | Uncharacterized LOC101927269 | GC07M080372 |
| PCCA-DT | PCCA Divergent Transcript | GC13M100053 |
| LINC01348 | Long Intergenic Non-Protein Coding RNA 1348 | GC01M235065 |
| LINC01595 | Long Intergenic Non-Protein Coding RNA 1595 | GC19M047865 |
| LOC101928858 | Uncharacterized LOC101928858 | GC05P067793 |
| ADRM1 | Adhesion Regulating Molecule 1 | GC20P062302 |
| ZGPAT | Zinc Finger CCCH-Type And G-Patch Domain Containing | GC20P063707 |
| RASA2 | RAS P21 Protein Activator 2 | GC03P141487 |
| NT5C2 | 5'-Nucleotidase, Cytosolic II | GC10M103088 |
| JMJD6 | Jumonji Domain Containing 6, Arginine Demethylase And Lysine Hydroxylase | GC17M076718 |
| FOXL1 | Forkhead Box L1 | GC16P086576 |
| MIR3662 | MicroRNA 3662 | GC06M134979 |
| MGST3 | Microsomal Glutathione S-Transferase 3 | GC01P165600 |
| FRAT2 | FRAT Regulator Of WNT Signaling Pathway 2 | GC10M097332 |
| ATP1B1 | ATPase Na+/K+ Transporting Subunit Beta 1 | GC01P169105 |
| UBE2J2 | Ubiquitin Conjugating Enzyme E2 J2 | GC01M001254 |
| CEP164 | Centrosomal Protein 164 | GC11P117314 |
| ATP5ME | ATP Synthase Membrane Subunit E | GC04M000673 |
| ENO3 | Enolase 3 | GC17P004948 |
| AQP7 | Aquaporin 7 | GC09M033384 |
| USP11 | Ubiquitin Specific Peptidase 11 | GC0XP047232 |
| FBXW2 | F-Box And WD Repeat Domain Containing 2 | GC09M120751 |
| AFM | Afamin | GC04P073481 |
| CTNNBL1 | Catenin Beta Like 1 | GC20P037693 |
| MUC17 | Mucin 17, Cell Surface Associated | GC07P101020 |
| ANGPTL3 | Angiopoietin Like 3 | GC01P062597 |
| HERC1 | HECT And RLD Domain Containing E3 Ubiquitin Protein Ligase Family Member 1 | GC15M063608 |
| SPRED1 | Sprouty Related EVH1 Domain Containing 1 | GC15P038252 |
| C6 | Complement C6 | GC05M041142 |
| ACOT8 | Acyl-CoA Thioesterase 8 | GC20M045841 |
| EIF3C | Eukaryotic Translation Initiation Factor 3 Subunit C | GC16P028689 |
| IRGM | Immunity Related GTPase M | GC05P150846 |
| OSBPL11 | Oxysterol Binding Protein Like 11 | GC03M125529 |
| NABP2 | Nucleic Acid Binding Protein 2 | GC12P056236 |
| LEPROT | Leptin Receptor Overlapping Transcript | GC01P065420 |
| LARP4 | La Ribonucleoprotein 4 | GC12P050392 |
| ACRBP | Acrosin Binding Protein | GC12M006638 |
| SLC25A48 | Solute Carrier Family 25 Member 48 | GC05P135579 |
| LOC111216272 | ADH1A Promoter | GC04U902758 |
| FMR1 | FMRP Translational Regulator 1 | GC0XP147912 |
| DSN1 | DSN1 Component Of MIS12 Kinetochore Complex | GC20M036751 |
| CSNK2A3 | Casein Kinase 2 Alpha 3 | GC11M011351 |
| PTPN18 | Protein Tyrosine Phosphatase Non-Receptor Type 18 | GC02P130356 |
| TASP1 | Taspase 1 | GC20M013389 |
| BOP1 | BOP1 Ribosomal Biogenesis Factor | GC08M144262 |
| FLII | FLII Actin Remodeling Protein | GC17M018244 |
| MARK4 | Microtubule Affinity Regulating Kinase 4 | GC19P045079 |
| SYTL2 | Synaptotagmin Like 2 | GC11M085694 |
| SH3KBP1 | SH3 Domain Containing Kinase Binding Protein 1 | GC0XM019552 |
| MLN | Motilin | GC06M033794 |
| MGA | MAX Dimerization Protein MGA | GC15P041621 |
| MAP3K2 | Mitogen-Activated Protein Kinase Kinase Kinase 2 | GC02M127298 |
| CDC34 | Cell Division Cycle 34 | GC19P000532 |
| ENPP7P13 | Ectonucleotide Pyrophosphatase/Phosphodiesterase 7 Pseudogene 13 | GC16P033769 |
| ZNF267 | Zinc Finger Protein 267 | GC16P031885 |
| KIR2DL3 | Killer Cell Immunoglobulin Like Receptor, Two Ig Domains And Long Cytoplasmic Tail 3 | GC19P055318 |
| CLN3 | CLN3 Lysosomal/Endosomal Transmembrane Protein, Battenin | GC16M028466 |
| DNAJB11 | DnaJ Heat Shock Protein Family (Hsp40) Member B11 | GC03P186567 |
| GLCE | Glucuronic Acid Epimerase | GC15P071840 |
| B3GNT2 | UDP-GlcNAc:BetaGal Beta-1,3-N-Acetylglucosaminyltransferase 2 | GC02P062196 |
| KANK2 | KN Motif And Ankyrin Repeat Domains 2 | GC19M011165 |
| SUPT6H | SPT6 Homolog, Histone Chaperone And Transcription Elongation Factor | GC17P028662 |
| ERGIC3 | ERGIC And Golgi 3 | GC20P035542 |
| KANK3 | KN Motif And Ankyrin Repeat Domains 3 | GC19M008322 |
| PATJ | PATJ Crumbs Cell Polarity Complex Component | GC01P061743 |
| MIR1260B | MicroRNA 1260b | GC11P096341 |
| BRD1 | Bromodomain Containing 1 | GC22M049773 |
| APOC1 | Apolipoprotein C1 | GC19P044914 |
| HSD17B12 | Hydroxysteroid 17-Beta Dehydrogenase 12 | GC11P043702 |
| ARL5A | ADP Ribosylation Factor Like GTPase 5A | GC02M151789 |
| REL | REL Proto-Oncogene, NF-KB Subunit | GC02P060881 |
| TKTL2 | Transketolase Like 2 | GC04M163471 |
| BMPR1B | Bone Morphogenetic Protein Receptor Type 1B | GC04P094757 |
| CLIP2 | CAP-Gly Domain Containing Linker Protein 2 | GC07P074289 |
| DPYSL3 | Dihydropyrimidinase Like 3 | GC05M147390 |
| AGBL2 | ATP/GTP Binding Protein Like 2 | GC11M059715 |
| TRIM66 | Tripartite Motif Containing 66 | GC11M008612 |
| MZB1 | Marginal Zone B And B1 Cell Specific Protein | GC05M139387 |
| FGF17 | Fibroblast Growth Factor 17 | GC08P022042 |
| SMPD2 | Sphingomyelin Phosphodiesterase 2 | GC06P109440 |
| FMNL3 | Formin Like 3 | GC12M049636 |
| DERL1 | Derlin 1 | GC08M123013 |
| GSTM5 | Glutathione S-Transferase Mu 5 | GC01P109711 |
| MGST2 | Microsomal Glutathione S-Transferase 2 | GC04P139665 |
| DPF1 | Double PHD Fingers 1 | GC19M038211 |
| GSTA5 | Glutathione S-Transferase Alpha 5 | GC06M052831 |
| ACTBL2 | Actin Beta Like 2 | GC05M057480 |
| LSINCT5 | Long Stress-Induced Non-Coding Transcript 5 | GC05P002712 |
| ADH1A | Alcohol Dehydrogenase 1A (Class I), Alpha Polypeptide | GC04M099276 |
| LDB1 | LIM Domain Binding 1 | GC10M102106 |
| ITGA7 | Integrin Subunit Alpha 7 | GC12M055684 |
| AK3 | Adenylate Kinase 3 | GC09M004703 |
| EGFL6 | EGF Like Domain Multiple 6 | GC0XP013569 |
| POLA1 | DNA Polymerase Alpha 1, Catalytic Subunit | GC0XP024693 |
| GABRB3 | Gamma-Aminobutyric Acid Type A Receptor Subunit Beta3 | GC15M026543 |
| PRR5 | Proline Rich 5 | GC22P044668 |
| MAT2B | Methionine Adenosyltransferase 2B | GC05P163523 |
| GNB1 | G Protein Subunit Beta 1 | GC01M001785 |
| SGPL1 | Sphingosine-1-Phosphate Lyase 1 | GC10P070815 |
| PROK2 | Prokineticin 2 | GC03M071771 |
| CSRP1 | Cysteine And Glycine Rich Protein 1 | GC01M201484 |
| MED23 | Mediator Complex Subunit 23 | GC06M131573 |
| SLIT1 | Slit Guidance Ligand 1 | GC10M096998 |
| PITPNM3 | PITPNM Family Member 3 | GC17M006451 |
| DDX24 | DEAD-Box Helicase 24 | GC14M094048 |
| DLX6 | Distal-Less Homeobox 6 | GC07P097005 |
| TNPO1 | Transportin 1 | GC05P072816 |
| LMO3 | LIM Domain Only 3 | GC12M016548 |
| PLXDC1 | Plexin Domain Containing 1 | GC17M039063 |
| SENP7 | SUMO Specific Peptidase 7 | GC03M101324 |
| SETD3 | SET Domain Containing 3, Actin Histidine Methyltransferase | GC14M099397 |
| POF1B | POF1B Actin Binding Protein | GC0XM085277 |
| RAD51AP1 | RAD51 Associated Protein 1 | GC12P006581 |
| MLF2 | Myeloid Leukemia Factor 2 | GC12M006750 |
| LUZP1 | Leucine Zipper Protein 1 | GC01M023085 |
| VPS13D | Vacuolar Protein Sorting 13 Homolog D | GC01P012231 |
| ZG16 | Zymogen Granule Protein 16 | GC16P029760 |
| FOXR2 | Forkhead Box R2 | GC0XP055623 |
| H2BC11 | H2B Clustered Histone 11 | GC06M027134 |
| MIR642A | MicroRNA 642a | GC19P045674 |
| ZNF350 | Zinc Finger Protein 350 | GC19M051965 |
| RNF41 | Ring Finger Protein 41 | GC12M056202 |
| MARCO | Macrophage Receptor With Collagenous Structure | GC02P118942 |
| MIR552 | MicroRNA 552 | GC01M034669 |
| RNASE3 | Ribonuclease A Family Member 3 | GC14P020891 |
| MAP3K21 | Mitogen-Activated Protein Kinase Kinase Kinase 21 | GC01P233329 |
| NFE2L1 | Nuclear Factor, Erythroid 2 Like 1 | GC17P048049 |
| TGIF2 | TGFB Induced Factor Homeobox 2 | GC20P036573 |
| METTL21A | Methyltransferase Like 21A | GC02M207580 |
| VPS54 | VPS54 Subunit Of GARP Complex | GC02M063892 |
| EEF1AKMT3 | EEF1A Lysine Methyltransferase 3 | GC12P057772 |
| LINC01451 | Long Intergenic Non-Protein Coding RNA 1451 | GC09M136616 |
| ENSG00000216663 |  | GC06M112326 |
| RASAL1 | RAS Protein Activator Like 1 | GC12M113098 |
| SPTAN1 | Spectrin Alpha, Non-Erythrocytic 1 | GC09P128552 |
| FBN2 | Fibrillin 2 | GC05M128257 |
| MRPL28 | Mitochondrial Ribosomal Protein L28 | GC16M000357 |
| SLC25A17 | Solute Carrier Family 25 Member 17 | GC22M044563 |
| PTPN22 | Protein Tyrosine Phosphatase Non-Receptor Type 22 | GC01M113813 |
| CDC123 | Cell Division Cycle 123 | GC10P012195 |
| CREB5 | CAMP Responsive Element Binding Protein 5 | GC07P028305 |
| RPS6KA5 | Ribosomal Protein S6 Kinase A5 | GC14M090847 |
| ANXA11 | Annexin A11 | GC10M080150 |
| TNIK | TRAF2 And NCK Interacting Kinase | GC03M171061 |
| NELFB | Negative Elongation Factor Complex Member B | GC09P137255 |
| SRI | Sorcin | GC07M088205 |
| SSR3 | Signal Sequence Receptor Subunit 3 | GC03M156540 |
| CCL28 | C-C Motif Chemokine Ligand 28 | GC05M043356 |
| SCLY | Selenocysteine Lyase | GC02P238061 |
| USP18 | Ubiquitin Specific Peptidase 18 | GC22P018149 |
| GEM | GTP Binding Protein Overexpressed In Skeletal Muscle | GC08M094249 |
| TMPRSS13 | Transmembrane Serine Protease 13 | GC11M117900 |
| GLIPR2 | GLI Pathogenesis Related 2 | GC09P036127 |
| CCDC40 | Coiled-Coil Domain Containing 40 | GC17P080037 |
| MBNL3 | Muscleblind Like Splicing Regulator 3 | GC0XM132369 |
| CROCC | Ciliary Rootlet Coiled-Coil, Rootletin | GC01P016765 |
| TXNDC9 | Thioredoxin Domain Containing 9 | GC02M099301 |
| GLYATL2 | Glycine-N-Acyltransferase Like 2 | GC11M059802 |
| C1orf43 | Chromosome 1 Open Reading Frame 43 | GC01M154206 |
| GPR78 | G Protein-Coupled Receptor 78 | GC04P008560 |
| FAM9C | Family With Sequence Similarity 9 Member C | GC0XM013053 |
| MIR610 | MicroRNA 610 | GC11P028056 |
| LINC01287 | Long Intergenic Non-Protein Coding RNA 1287 | GC07M153399 |
| MIR4458 | MicroRNA 4458 | GC05P008460 |
| PHACTR2-AS1 | PHACTR2 Antisense RNA 1 | GC06M143555 |
| LINC01374 | Long Intergenic Non-Protein Coding RNA 1374 | GC10P090039 |
| MIR892A | MicroRNA 892a | GC0XM146022 |
| UC.134 | Long Non-Coding RNA Uc.134 | GC03U903184 |
| MICD | MHC Class I Polypeptide-Related Sequence D (Pseudogene) | GC06M030649 |
| TRIM31-AS1 | TRIM31 Antisense RNA 1 | GC06P033225 |
| HLA-W | Major Histocompatibility Complex, Class I, W (Pseudogene) | GC06P033193 |
| RPL23AP1 | Ribosomal Protein L23a Pseudogene 1 | GC06M030628 |
| HLA-DPA3 | Major Histocompatibility Complex, Class II, DP Alpha 3 (Pseudogene) | GC06M033131 |
| RNY4P10 | RNY4 Pseudogene 10 | GC06P033199 |
| ENSG00000259053 |  | GC14M093526 |
| SLC6A3 | Solute Carrier Family 6 Member 3 | GC05M001392 |
| EIF2S3 | Eukaryotic Translation Initiation Factor 2 Subunit Gamma | GC0XP024054 |
| GABRA3 | Gamma-Aminobutyric Acid Type A Receptor Subunit Alpha3 | GC0XM152166 |
| GABARAPL1 | GABA Type A Receptor Associated Protein Like 1 | GC12P010212 |
| SLC38A2 | Solute Carrier Family 38 Member 2 | GC12M046358 |
| RAB35 | RAB35, Member RAS Oncogene Family | GC12M120096 |
| ARHGEF19 | Rho Guanine Nucleotide Exchange Factor 19 | GC01M016197 |
| NOSTRIN | Nitric Oxide Synthase Trafficking | GC02P168786 |
| CCDC8 | Coiled-Coil Domain Containing 8 | GC19M046410 |
| GINS4 | GINS Complex Subunit 4 | GC08P041529 |
| FSIP1 | Fibrous Sheath Interacting Protein 1 | GC15M039594 |
| CCNJ | Cyclin J | GC10P096043 |
| H2BC9 | H2B Clustered Histone 9 | GC06P028386 |
| PLPPR5 | Phospholipid Phosphatase Related 5 | GC01M098891 |
| MIR769 | MicroRNA 769 | GC19P046018 |
| MIR877 | MicroRNA 877 | GC06P030584 |
| MIR520G | MicroRNA 520g | GC19P053722 |
| MIR920 | MicroRNA 920 | GC12P024212 |
| MIR922 | MicroRNA 922 | GC03M197674 |
| MIR575 | MicroRNA 575 | GC04M082753 |
| RAD51-AS1 | RAD51 Antisense RNA 1 | GC15M040686 |
| MIR329-2 | MicroRNA 329-2 | GC14P104445 |
| MIR924 | MicroRNA 924 | GC18M039622 |
| MIR4782 | MicroRNA 4782 | GC02M113721 |
| MIR4443 | MicroRNA 4443 | GC03P048198 |
| MIR194-2HG | MIR194-2 Host Gene | GC11M064890 |
| LOC106014249 | PAX6 Upstream Regulatory Region | GC11U902918 |
| SEC14L2 | SEC14 Like Lipid Binding 2 | GC22P030396 |
| TNFSF13B | TNF Superfamily Member 13b | GC13P108251 |
| UQCRC1 | Ubiquinol-Cytochrome C Reductase Core Protein 1 | GC03M048598 |
| VLDLR | Very Low Density Lipoprotein Receptor | GC09P002611 |
| IL2RG | Interleukin 2 Receptor Subunit Gamma | GC0XM071108 |
| PCSK6 | Proprotein Convertase Subtilisin/Kexin Type 6 | GC15M101297 |
| ITIH5 | Inter-Alpha-Trypsin Inhibitor Heavy Chain 5 | GC10M007559 |
| PCSK7 | Proprotein Convertase Subtilisin/Kexin Type 7 | GC11M117199 |
| HEXA | Hexosaminidase Subunit Alpha | GC15M072340 |
| AASS | Aminoadipate-Semialdehyde Synthase | GC07M122073 |
| ART3 | ADP-Ribosyltransferase 3 | GC04P076011 |
| TNIP2 | TNFAIP3 Interacting Protein 2 | GC04M002741 |
| LIN9 | Lin-9 DREAM MuvB Core Complex Component | GC01M226231 |
| GINS2 | GINS Complex Subunit 2 | GC16M085676 |
| SERPINB12 | Serpin Family B Member 12 | GC18P063556 |
| GRASP | General Receptor For Phosphoinositides 1 Associated Scaffold Protein | GC12P052006 |
| RASSF7 | Ras Association Domain Family Member 7 | GC11P000560 |
| RTP3 | Receptor Transporter Protein 3 | GC03P046538 |
| ACVR1C | Activin A Receptor Type 1C | GC02M157526 |
| ABCG1 | ATP Binding Cassette Subfamily G Member 1 | GC21P042199 |
| HTR2B | 5-Hydroxytryptamine Receptor 2B | GC02M231108 |
| UBE2V2 | Ubiquitin Conjugating Enzyme E2 V2 | GC08P047998 |
| CHN1 | Chimerin 1 | GC02M174799 |
| U2AF2 | U2 Small Nuclear RNA Auxiliary Factor 2 | GC19P055654 |
| PLCD4 | Phospholipase C Delta 4 | GC02P218608 |
| DUSP28 | Dual Specificity Phosphatase 28 | GC02P240560 |
| FLAD1 | Flavin Adenine Dinucleotide Synthetase 1 | GC01P154983 |
| BCCIP | BRCA2 And CDKN1A Interacting Protein | GC10P125823 |
| CATSPERZ | Catsper Channel Auxiliary Subunit Zeta | GC11P064304 |
| DIAPH1 | Diaphanous Related Formin 1 | GC05M141477 |
| AMPD2 | Adenosine Monophosphate Deaminase 2 | GC01P109616 |
| GRHPR | Glyoxylate And Hydroxypyruvate Reductase | GC09P037412 |
| OLR1 | Oxidized Low Density Lipoprotein Receptor 1 | GC12M013371 |
| MAPKAPK5 | MAPK Activated Protein Kinase 5 | GC12P111842 |
| DDAH1 | Dimethylarginine Dimethylaminohydrolase 1 | GC01M085318 |
| OPA1 | OPA1 Mitochondrial Dynamin Like GTPase | GC03P193594 |
| IL21R | Interleukin 21 Receptor | GC16P027413 |
| CYB5B | Cytochrome B5 Type B | GC16P069424 |
| RBM8A | RNA Binding Motif Protein 8A | GC01M145921 |
| GSTK1 | Glutathione S-Transferase Kappa 1 | GC07P144742 |
| HLX | H2.0 Like Homeobox | GC01P220879 |
| P3H1 | Prolyl 3-Hydroxylase 1 | GC01M042746 |
| STX2 | Syntaxin 2 | GC12M130789 |
| TXNDC12 | Thioredoxin Domain Containing 12 | GC01M052020 |
| STK26 | Serine/Threonine Kinase 26 | GC0XP132023 |
| PROKR1 | Prokineticin Receptor 1 | GC02P068643 |
| TMX1 | Thioredoxin Related Transmembrane Protein 1 | GC14P051240 |
| BYSL | Bystin Like | GC06P046104 |
| SYPL1 | Synaptophysin Like 1 | GC07M106090 |
| MYO18A | Myosin XVIIIA | GC17M029707 |
| DUOXA1 | Dual Oxidase Maturation Factor 1 | GC15M045119 |
| SAMSN1 | SAM Domain, SH3 Domain And Nuclear Localization Signals 1 | GC21M014485 |
| ABHD10 | Abhydrolase Domain Containing 10 | GC03P111978 |
| BZW2 | Basic Leucine Zipper And W2 Domains 2 | GC07P016646 |
| ZC3HAV1 | Zinc Finger CCCH-Type Containing, Antiviral 1 | GC07M139044 |
| IL9R | Interleukin 9 Receptor | GC0XP155997 |
| MICALL2 | MICAL Like 2 | GC07M001441 |
| FBXO6 | F-Box Protein 6 | GC01P011664 |
| ARHGAP44 | Rho GTPase Activating Protein 44 | GC17P012789 |
| KIF27 | Kinesin Family Member 27 | GC09M083856 |
| COA7 | Cytochrome C Oxidase Assembly Factor 7 (Putative) | GC01M052684 |
| SGTB | Small Glutamine Rich Tetratricopeptide Repeat Containing Beta | GC05M065665 |
| SPIDR | Scaffold Protein Involved In DNA Repair | GC08P047260 |
| MIR519C | MicroRNA 519c | GC19P053686 |
| MIR601 | MicroRNA 601 | GC09M123402 |
| KTN1-AS1 | KTN1 Antisense RNA 1 | GC14M055499 |
| MIR767 | MicroRNA 767 | GC0XM152393 |
| MIR522 | MicroRNA 522 | GC19P053751 |
| MIR591 | MicroRNA 591 | GC07M096219 |
| MSRB3 | Methionine Sulfoxide Reductase B3 | GC12P065279 |
| ABT1 | Activator Of Basal Transcription 1 | GC06P028049 |
| LIMA1 | LIM Domain And Actin Binding 1 | GC12M050175 |
| RANBP1 | RAN Binding Protein 1 | GC22P020115 |
| RSL1D1 | Ribosomal L1 Domain Containing 1 | GC16M011833 |
| MARVELD2 | MARVEL Domain Containing 2 | GC05P069415 |
| PPT1 | Palmitoyl-Protein Thioesterase 1 | GC01M040072 |
| TMBIM6 | Transmembrane BAX Inhibitor Motif Containing 6 | GC12P049707 |
| RAET1E | Retinoic Acid Early Transcript 1E | GC06M149883 |
| LOC111162621 | DeltaNp63 Promoter Of Tumor Protein P63 | GC03U903197 |
| CYC1 | Cytochrome C1 | GC08P144095 |
| IWS1 | Interacts With SUPT6H, CTD Assembly Factor 1 | GC02M127436 |
| ARAP1 | ArfGAP With RhoGAP Domain, Ankyrin Repeat And PH Domain 1 | GC11M072686 |
| GGA3 | Golgi Associated, Gamma Adaptin Ear Containing, ARF Binding Protein 3 | GC17M075225 |
| CYP2C8 | Cytochrome P450 Family 2 Subfamily C Member 8 | GC10M095038 |
| ULBP2 | UL16 Binding Protein 2 | GC06P149941 |
| CIT | Citron Rho-Interacting Serine/Threonine Kinase | GC12M119650 |
| AMPD1 | Adenosine Monophosphate Deaminase 1 | GC01M114673 |
| SPTA1 | Spectrin Alpha, Erythrocytic 1 | GC01M158610 |
| CNDP1 | Carnosine Dipeptidase 1 | GC18P074534 |
| CRY1 | Cryptochrome Circadian Regulator 1 | GC12M106991 |
| DGKG | Diacylglycerol Kinase Gamma | GC03M186105 |
| GMDS | GDP-Mannose 4,6-Dehydratase | GC06M001624 |
| BAAT | Bile Acid-CoA:Amino Acid N-Acyltransferase | GC09M101360 |
| KMO | Kynurenine 3-Monooxygenase | GC01P241532 |
| RAPGEF4 | Rap Guanine Nucleotide Exchange Factor 4 | GC02P172735 |
| CLIC5 | Chloride Intracellular Channel 5 | GC06M045848 |
| CLDN14 | Claudin 14 | GC21M036460 |
| NR2E1 | Nuclear Receptor Subfamily 2 Group E Member 1 | GC06P108166 |
| PSMD12 | Proteasome 26S Subunit, Non-ATPase 12 | GC17M067337 |
| PSMD7 | Proteasome 26S Subunit, Non-ATPase 7 | GC16P074296 |
| RFX5 | Regulatory Factor X5 | GC01M151313 |
| PSMB3 | Proteasome 20S Subunit Beta 3 | GC17P038752 |
| CHMP4B | Charged Multivesicular Body Protein 4B | GC20P033812 |
| ARHGAP4 | Rho GTPase Activating Protein 4 | GC0XM153907 |
| SRSF7 | Serine And Arginine Rich Splicing Factor 7 | GC02M038709 |
| PEMT | Phosphatidylethanolamine N-Methyltransferase | GC17M017506 |
| RAB31 | RAB31, Member RAS Oncogene Family | GC18P009701 |
| SLAMF6 | SLAM Family Member 6 | GC01M160454 |
| STXBP3 | Syntaxin Binding Protein 3 | GC01P108746 |
| CYTH2 | Cytohesin 2 | GC19P048470 |
| HPCAL1 | Hippocalcin Like 1 | GC02P010302 |
| SCD5 | Stearoyl-CoA Desaturase 5 | GC04M082629 |
| RPL36 | Ribosomal Protein L36 | GC19P005674 |
| RPL30 | Ribosomal Protein L30 | GC08M098024 |
| B3GALNT2 | Beta-1,3-N-Acetylgalactosaminyltransferase 2 | GC01M235440 |
| TPPP | Tubulin Polymerization Promoting Protein | GC05M000659 |
| IGSF3 | Immunoglobulin Superfamily Member 3 | GC01M116574 |
| HNRNPH3 | Heterogeneous Nuclear Ribonucleoprotein H3 | GC10P068331 |
| EDARADD | EDAR Associated Death Domain | GC01P236348 |
| CHST12 | Carbohydrate Sulfotransferase 12 | GC07P002403 |
| CFHR3 | Complement Factor H Related 3 | GC01P196774 |
| MGAT4B | Alpha-1,3-Mannosyl-Glycoprotein 4-Beta-N-Acetylglucosaminyltransferase B | GC05M179797 |
| DNER | Delta/Notch Like EGF Repeat Containing | GC02M229357 |
| OPN3 | Opsin 3 | GC01M241590 |
| CDC42EP3 | CDC42 Effector Protein 3 | GC02M037610 |
| ART4 | ADP-Ribosyltransferase 4 (Dombrock Blood Group) | GC12M014825 |
| CD48 | CD48 Molecule | GC01M160648 |
| ZNF24 | Zinc Finger Protein 24 | GC18M035332 |
| TUFT1 | Tuftelin 1 | GC01P151513 |
| SLC35C1 | Solute Carrier Family 35 Member C1 | GC11P045958 |
| LMCD1 | LIM And Cysteine Rich Domains 1 | GC03P008518 |
| PEF1 | Penta-EF-Hand Domain Containing 1 | GC01M031630 |
| PPP1R12B | Protein Phosphatase 1 Regulatory Subunit 12B | GC01P202348 |
| PBLD | Phenazine Biosynthesis Like Protein Domain Containing | GC10M068282 |
| OXGR1 | Oxoglutarate Receptor 1 | GC13M096985 |
| SH3RF1 | SH3 Domain Containing Ring Finger 1 | GC04M169094 |
| SIGLEC10 | Sialic Acid Binding Ig Like Lectin 10 | GC19M051410 |
| STAB2 | Stabilin 2 | GC12P103587 |
| GSPT2 | G1 To S Phase Transition 2 | GC0XP051743 |
| GUF1 | GUF1 Homolog, GTPase | GC04P044680 |
| HMCN1 | Hemicentin 1 | GC01P185734 |
| NCAPG2 | Non-SMC Condensin II Complex Subunit G2 | GC07M158631 |
| FXYD6 | FXYD Domain Containing Ion Transport Regulator 6 | GC11M117836 |
| BMS1 | BMS1 Ribosome Biogenesis Factor | GC10P042782 |
| BUD31 | BUD31 Homolog | GC07P099408 |
| CAB39 | Calcium Binding Protein 39 | GC02P230712 |
| APOBEC3F | Apolipoprotein B MRNA Editing Enzyme Catalytic Subunit 3F | GC22P039040 |
| FAM83H | Family With Sequence Similarity 83 Member H | GC08M143723 |
| TOR1B | Torsin Family 1 Member B | GC09P129803 |
| UNC5A | Unc-5 Netrin Receptor A | GC05P176813 |
| VAT1 | Vesicle Amine Transport 1 | GC17M043014 |
| KHDRBS2 | KH RNA Binding Domain Containing, Signal Transduction Associated 2 | GC06M061631 |
| TMEM9 | Transmembrane Protein 9 | GC01M201134 |
| NFE2L3 | Nuclear Factor, Erythroid 2 Like 3 | GC07P026152 |
| EHD3 | EH Domain Containing 3 | GC02P031234 |
| COG6 | Component Of Oligomeric Golgi Complex 6 | GC13P039655 |
| SCPEP1 | Serine Carboxypeptidase 1 | GC17P056978 |
| SF3A3 | Splicing Factor 3a Subunit 3 | GC01M037956 |
| GABRQ | Gamma-Aminobutyric Acid Type A Receptor Subunit Theta | GC0XP152638 |
| AKR7A3 | Aldo-Keto Reductase Family 7 Member A3 | GC01M019282 |
| TPTE | Transmembrane Phosphatase With Tensin Homology | GC21P010521 |
| TRIM55 | Tripartite Motif Containing 55 | GC08P066184 |
| PTPMT1 | Protein Tyrosine Phosphatase Mitochondrial 1 | GC11P047568 |
| IGSF11 | Immunoglobulin Superfamily Member 11 | GC03M118900 |
| PRDM8 | PR/SET Domain 8 | GC04P080183 |
| LRRC1 | Leucine Rich Repeat Containing 1 | GC06P053798 |
| CMTM6 | CKLF Like MARVEL Transmembrane Domain Containing 6 | GC03M032499 |
| CHST13 | Carbohydrate Sulfotransferase 13 | GC03P126524 |
| MANEA | Mannosidase Endo-Alpha | GC06P095577 |
| TONSL | Tonsoku Like, DNA Repair Protein | GC08M144428 |
| SPRYD4 | SPRY Domain Containing 4 | GC12P056468 |
| PCDH11X | Protocadherin 11 X-Linked | GC0XP091779 |
| KIZ | Kizuna Centrosomal Protein | GC20P021125 |
| DBX2 | Developing Brain Homeobox 2 | GC12M045014 |
| DMWD | DM1 Locus, WD Repeat Containing | GC19M045782 |
| DNAH10 | Dynein Axonemal Heavy Chain 10 | GC12P123762 |
| SARS1 | Seryl-TRNA Synthetase 1 | GC01P109214 |
| AKAP3 | A-Kinase Anchoring Protein 3 | GC12M004614 |
| ADAM21 | ADAM Metallopeptidase Domain 21 | GC14P070452 |
| FBXL5 | F-Box And Leucine Rich Repeat Protein 5 | GC04M015606 |
| FKBP11 | FKBP Prolyl Isomerase 11 | GC12M048934 |
| WWC2 | WW And C2 Domain Containing 2 | GC04P183099 |
| TRIM7 | Tripartite Motif Containing 7 | GC05M181193 |
| SPAG7 | Sperm Associated Antigen 7 | GC17M004959 |
| SYAP1 | Synapse Associated Protein 1 | GC0XP016719 |
| TMED3 | Transmembrane P24 Trafficking Protein 3 | GC15P079311 |
| DENND5B | DENN Domain Containing 5B | GC12M031392 |
| GOLGA3 | Golgin A3 | GC12M132768 |
| GPR155 | G Protein-Coupled Receptor 155 | GC02M174431 |
| RRS1 | Ribosome Biogenesis Regulator 1 Homolog | GC08P066429 |
| CDKN2AIP | CDKN2A Interacting Protein | GC04P183444 |
| CCDC47 | Coiled-Coil Domain Containing 47 | GC17M063745 |
| ANP32E | Acidic Nuclear Phosphoprotein 32 Family Member E | GC01M150190 |
| EPPK1 | Epiplakin 1 | GC08M143857 |
| KRT26 | Keratin 26 | GC17M040766 |
| KLHL21 | Kelch Like Family Member 21 | GC01M006591 |
| TMEM100 | Transmembrane Protein 100 | GC17M055719 |
| UBQLN3 | Ubiquilin 3 | GC11M005669 |
| TRMT6 | TRNA Methyltransferase 6 | GC20M005937 |
| SLC44A5 | Solute Carrier Family 44 Member 5 | GC01M075202 |
| IFI44L | Interferon Induced Protein 44 Like | GC01P078619 |
| SVOP | SV2 Related Protein | GC12M108907 |
| CEP85 | Centrosomal Protein 85 | GC01P026234 |
| DNAJC14 | DnaJ Heat Shock Protein Family (Hsp40) Member C14 | GC12M055820 |
| F8A1 | Coagulation Factor VIII Associated 1 | GC0XP154886 |
| EFCAB2 | EF-Hand Calcium Binding Domain 2 | GC01P244969 |
| ICE2 | Interactor Of Little Elongation Complex ELL Subunit 2 | GC15M060419 |
| CMYA5 | Cardiomyopathy Associated 5 | GC05P079689 |
| RSBN1L | Round Spermatid Basic Protein 1 Like | GC07P077696 |
| CCDC174 | Coiled-Coil Domain Containing 174 | GC03P014655 |
| FBXO36 | F-Box Protein 36 | GC02P229923 |
| KRI1 | KRI1 Homolog | GC19M010553 |
| VNN3 | Vanin 3 | GC06M132722 |
| LCTL | Lactase Like | GC15M066547 |
| PRMT9 | Protein Arginine Methyltransferase 9 | GC04M147637 |
| SH3D21 | SH3 Domain Containing 21 | GC01P036306 |
| SOGA1 | Suppressor Of Glucose, Autophagy Associated 1 | GC20M036777 |
| CLDN17 | Claudin 17 | GC21M030165 |
| RNF187 | Ring Finger Protein 187 | GC01P228487 |
| RMDN1 | Regulator Of Microtubule Dynamics 1 | GC08M086467 |
| DUSP21 | Dual Specificity Phosphatase 21 | GC0XP044844 |
| SAC3D1 | SAC3 Domain Containing 1 | GC11P065040 |
| FBXO34 | F-Box Protein 34 | GC14P055271 |
| ZC3H12B | Zinc Finger CCCH-Type Containing 12B | GC0XP065034 |
| L3MBTL4 | L3MBTL Histone Methyl-Lysine Binding Protein 4 | GC18M005954 |
| TRIM60 | Tripartite Motif Containing 60 | GC04P165027 |
| ZNF717 | Zinc Finger Protein 717 | GC03M075709 |
| DTHD1 | Death Domain Containing 1 | GC04P036283 |
| FAM43B | Family With Sequence Similarity 43 Member B | GC01P020553 |
| ZNRD2 | Zinc Ribbon Domain Containing 2 | GC11P065626 |
| PCDH11Y | Protocadherin 11 Y-Linked | GC0YP005000 |
| SVBP | Small Vasohibin Binding Protein | GC01M042808 |
| MFSD4A | Major Facilitator Superfamily Domain Containing 4A | GC01P205568 |
| OR2T29 | Olfactory Receptor Family 2 Subfamily T Member 29 | GC01M248558 |
| GRAMD2A | GRAM Domain Containing 2A | GC15M072160 |
| RMC1 | Regulator Of MON1-CCZ1 | GC18P023504 |
| OR2T5 | Olfactory Receptor Family 2 Subfamily T Member 5 | GC01P248488 |
| BBOF1 | Basal Body Orientation Factor 1 | GC14P074021 |
| INSYN2B | Inhibitory Synaptic Factor Family Member 2B | GC05M169861 |
| LINC00461 | Long Intergenic Non-Protein Coding RNA 461 | GC05M088507 |
| PTTG3P | Pituitary Tumor-Transforming 3, Pseudogene | GC08M066767 |
| MIR1224 | MicroRNA 1224 | GC03P184241 |
| MIR1306 | MicroRNA 1306 | GC22P020086 |
| LINC00161 | Long Intergenic Non-Protein Coding RNA 161 | GC21P028539 |
| MIR636 | MicroRNA 636 | GC17M076736 |
| EIF2S3B | Eukaryotic Translation Initiation Factor 2 Subunit Gamma B | GC12P010505 |
| MIR663B | MicroRNA 663b | GC02M132435 |
| MIR1296 | MicroRNA 1296 | GC10M063372 |
| LINCMD1 | Long Intergenic Non-Protein Coding RNA, Muscle Differentiation 1 | GC06M052146 |
| EEF1A1P5 | Eukaryotic Translation Elongation Factor 1 Alpha 1 Pseudogene 5 | GC09P133019 |
| FAM99B | Family With Sequence Similarity 99 Member B | GC11M001684 |
| FLVCR1-DT | FLVCR1 Divergent Transcript | GC01M212856 |
| LINC00462 | Long Intergenic Non-Protein Coding RNA 462 | GC13M048576 |
| MIR3117 | MicroRNA 3117 | GC01P066628 |
| MIR3178 | MicroRNA 3178 | GC16M002531 |
| DUX4L1 | Double Homeobox 4 Like 1 (Pseudogene) | GC04P190084 |
| MIR512-2 | MicroRNA 512-2 | GC19P054093 |
| MIR513C | MicroRNA 513c | GC0XM147189 |
| MIR602 | MicroRNA 602 | GC09P137838 |
| MIR1231 | MicroRNA 1231 | GC01P201777 |
| LINC01080 | Long Intergenic Non-Protein Coding RNA 1080 | GC13P079873 |
| MIR3126 | MicroRNA 3126 | GC02P069103 |
| MIR4516 | MicroRNA 4516 | GC16P002392 |
| MIR376A2 | MicroRNA 376a-2 | GC14P104449 |
| CARD8-AS1 | CARD8 Antisense RNA 1 | GC19P048255 |
| LINC00682 | Long Intergenic Non-Protein Coding RNA 682 | GC04M041878 |
| MIR1268A | MicroRNA 1268a | GC15M022225 |
| SNORD113-1 | Small Nucleolar RNA, C/D Box 113-1 | GC14P104262 |
| SNORD76 | Small Nucleolar RNA, C/D Box 76 | GC01M174127 |
| RB1-DT | RB1 Divergent Transcript | GC13M048297 |
| MIR3131 | MicroRNA 3131 | GC02M219058 |
| MIR1199 | MicroRNA 1199 | GC19P014073 |
| MIR3910-1 | MicroRNA 3910-1 | GC09P091636 |
| MIR764 | MicroRNA 764 | GC0XP114639 |
| MIR4262 | MicroRNA 4262 | GC02M011836 |
| MIR4641 | MicroRNA 4641 | GC06P041598 |
| MIR3910-2 | MicroRNA 3910-2 | GC09M091636 |
| MIR103B2 | MicroRNA 103b-2 | GC20M003899 |
| LINC01703 | Long Intergenic Non-Protein Coding RNA 1703 | GC01P226085 |
| MIR6875 | MicroRNA 6875 | GC07P100868 |
| MIR103B1 | MicroRNA 103b-1 | GC05P168560 |
| KRT8P3 | Keratin 8 Pseudogene 3 | GC08P061578 |
| MIR5692A1 | MicroRNA 5692a-1 | GC07P097963 |
| UBE2CP3 | Ubiquitin Conjugating Enzyme E2 C Pseudogene 3 | GC04P057073 |
| HVBS8 | Hepatitis B Virus Integration Site 8 | GC17U990042 |
| MIR1843 | MicroRNA 1843 | GC01U906052 |
| MIR9985 | MicroRNA 9985 | GC0YU900784 |
| MIR9986 | MicroRNA 9986 | GC02U904688 |
| MIR548BC | MicroRNA 548BC | GC17U902874 |
| EGILA | EGFR Interacting LncRNA | GC00U936868 |
| LOC110599568 | MS43a Minisatellite Repeat Instability Region | GC12U902739 |
| LGMN | Legumain | GC14M092703 |
| MIR935 | MicroRNA 935 | GC19P054123 |
| SMOX | Spermine Oxidase | GC20P004120 |
| H2AZ1 | H2A.Z Variant Histone 1 | GC04M099949 |
| MOB2 | MOB Kinase Activator 2 | GC11M001470 |
| IL3RA | Interleukin 3 Receptor Subunit Alpha | GC0XP001336 |
| C1QA | Complement C1q A Chain | GC01P022636 |
| GALNS | Galactosamine (N-Acetyl)-6-Sulfatase | GC16M088813 |
| PPP1CB | Protein Phosphatase 1 Catalytic Subunit Beta | GC02P028752 |
| VAV1 | Vav Guanine Nucleotide Exchange Factor 1 | GC19P006772 |
| C4A | Complement C4A (Rodgers Blood Group) | GC06P033433 |
| KANSL1 | KAT8 Regulatory NSL Complex Subunit 1 | GC17M046031 |
| NUAK2 | NUAK Family Kinase 2 | GC01M205302 |
| UBE2N | Ubiquitin Conjugating Enzyme E2 N | GC12M093406 |
| RAET1G | Retinoic Acid Early Transcript 1G | GC06M149916 |
| CCNF | Cyclin F | GC16P002429 |
| DDX41 | DEAD-Box Helicase 41 | GC05M177511 |
| CXCL6 | C-X-C Motif Chemokine Ligand 6 | GC04P073837 |
| MYBBP1A | MYB Binding Protein 1a | GC17M004538 |
| OSBPL8 | Oxysterol Binding Protein Like 8 | GC12M076354 |
| BNIPL | BCL2 Interacting Protein Like | GC01P151036 |
| KIR2DL1 | Killer Cell Immunoglobulin Like Receptor, Two Ig Domains And Long Cytoplasmic Tail 1 | GC19P055289 |
| CHAT | Choline O-Acetyltransferase | GC10P049609 |
| GATD3A | Glutamine Amidotransferase Like Class 1 Domain Containing 3A | GC21P044134 |
| PTGDS | Prostaglandin D2 Synthase | GC09P136977 |
| PDE3B | Phosphodiesterase 3B | GC11P014643 |
| NOM1 | Nucleolar Protein With MIF4G Domain 1 | GC07P156949 |
| REPIN1 | Replication Initiator 1 | GC07P150368 |
| LOC102725019 | Uncharacterized LOC102725019 | GC06P032718 |
| ENSG00000237669 |  | GC06M030684 |
| NEIL2 | Nei Like DNA Glycosylase 2 | GC08P011769 |
| LIPA | Lipase A, Lysosomal Acid Type | GC10M089213 |
| HBP1 | HMG-Box Transcription Factor 1 | GC07P107168 |
| CXCL3 | C-X-C Motif Chemokine Ligand 3 | GC04M074036 |
| NPC2 | NPC Intracellular Cholesterol Transporter 2 | GC14M074476 |
| XAB2 | XPA Binding Protein 2 | GC19M007619 |
| TK2 | Thymidine Kinase 2 | GC16M066508 |
| CCNK | Cyclin K | GC14P099483 |
| KIR2DS5 | Killer Cell Immunoglobulin Like Receptor, Two Ig Domains And Short Cytoplasmic Tail 5 | GC19MR00030 |
| LOC110366354 | CYP3A4 5' Regulatory Region | GC07U904757 |
| RPS6KA2 | Ribosomal Protein S6 Kinase A2 | GC06M166409 |
| NDUFV1 | NADH:Ubiquinone Oxidoreductase Core Subunit V1 | GC11P067623 |
| COA3 | Cytochrome C Oxidase Assembly Factor 3 | GC17M042795 |
| ASAP1 | ArfGAP With SH3 Domain, Ankyrin Repeat And PH Domain 1 | GC08M130052 |
| MIR664A | MicroRNA 664a | GC01M220200 |
| FGR | FGR Proto-Oncogene, Src Family Tyrosine Kinase | GC01M027622 |
| WFDC21P | WAP Four-Disulfide Core Domain 21, Pseudogene | GC17M060085 |
| NOMO3 | NODAL Modulator 3 | GC16P016232 |
| PSME2 | Proteasome Activator Subunit 2 | GC14M024143 |
| COL16A1 | Collagen Type XVI Alpha 1 Chain | GC01M031653 |
| LZTS2 | Leucine Zipper Tumor Suppressor 2 | GC10P100996 |
| A2M | Alpha-2-Macroglobulin | GC12M009067 |
| ZNF274 | Zinc Finger Protein 274 | GC19P058182 |
| AS3MT | Arsenite Methyltransferase | GC10P102869 |
| TP53I11 | Tumor Protein P53 Inducible Protein 11 | GC11M044881 |
| BBS9 | Bardet-Biedl Syndrome 9 | GC07P033112 |
| TANK | TRAF Family Member Associated NFKB Activator | GC02P161136 |
| PRPF40A | Pre-MRNA Processing Factor 40 Homolog A | GC02M152651 |
| SERPINF2 | Serpin Family F Member 2 | GC17P001742 |
| IFNGR2 | Interferon Gamma Receptor 2 | GC21P033402 |
| KLRG1 | Killer Cell Lectin Like Receptor G1 | GC12P008950 |
| ITGAE | Integrin Subunit Alpha E | GC17M003722 |
| CANT1 | Calcium Activated Nucleotidase 1 | GC17M078992 |
| DPT | Dermatopontin | GC01M168664 |
| PABPC4 | Poly(A) Binding Protein Cytoplasmic 4 | GC01M039560 |
| ELF2 | E74 Like ETS Transcription Factor 2 | GC04M139028 |
| LOC111255645 | TNFRSF10A 5' Regulatory Region | GC08U902766 |
| RPS2 | Ribosomal Protein S2 | GC16M002160 |
| UBE2L6 | Ubiquitin Conjugating Enzyme E2 L6 | GC11M059789 |
| COL15A1 | Collagen Type XV Alpha 1 Chain | GC09P098943 |
| ABHD5 | Abhydrolase Domain Containing 5 | GC03P043707 |
| TMEM165 | Transmembrane Protein 165 | GC04P055395 |
| AKR1C4 | Aldo-Keto Reductase Family 1 Member C4 | GC10P005195 |
| MAVS | Mitochondrial Antiviral Signaling Protein | GC20P003827 |
| SCARB2 | Scavenger Receptor Class B Member 2 | GC04M076158 |
| HADHA | Hydroxyacyl-CoA Dehydrogenase Trifunctional Multienzyme Complex Subunit Alpha | GC02M026190 |
| PRSS23 | Serine Protease 23 | GC11P086791 |
| BTBD3 | BTB Domain Containing 3 | GC20P011890 |
| PRIM1 | DNA Primase Subunit 1 | GC12M056731 |
| NCKAP1 | NCK Associated Protein 1 | GC02M182909 |
| CTF1 | Cardiotrophin 1 | GC16P030910 |
| JADE3 | Jade Family PHD Finger 3 | GC0XP046912 |
| MAST4 | Microtubule Associated Serine/Threonine Kinase Family Member 4 | GC05P066596 |
| DLX6-AS1 | DLX6 Antisense RNA 1 | GC07M096955 |
| FGF23 | Fibroblast Growth Factor 23 | GC12M004368 |
| SENP3 | SUMO Specific Peptidase 3 | GC17P007937 |
| KIR2DS1 | Killer Cell Immunoglobulin Like Receptor, Two Ig Domains And Short Cytoplasmic Tail 1 | GC19MR00013 |
| NAA10 | N-Alpha-Acetyltransferase 10, NatA Catalytic Subunit | GC0XM153929 |
| LRP2 | LDL Receptor Related Protein 2 | GC02M169127 |
| LOC108281177 | SOX2 5' Regulatory Region | GC03U903121 |
| RPS14 | Ribosomal Protein S14 | GC05M150443 |
| NEK8 | NIMA Related Kinase 8 | GC17P028725 |
| PUM1 | Pumilio RNA Binding Family Member 1 | GC01M030931 |
| AIMP1 | Aminoacyl TRNA Synthetase Complex Interacting Multifunctional Protein 1 | GC04P106315 |
| TRIB1 | Tribbles Pseudokinase 1 | GC08P125430 |
| INTS4 | Integrator Complex Subunit 4 | GC11M077878 |
| PF4 | Platelet Factor 4 | GC04M073980 |
| LRP10 | LDL Receptor Related Protein 10 | GC14P022871 |
| DYSF | Dysferlin | GC02P071453 |
| SNIP1 | Smad Nuclear Interacting Protein 1 | GC01M037534 |
| IL1RAP | Interleukin 1 Receptor Accessory Protein | GC03P190514 |
| SOX13 | SRY-Box Transcription Factor 13 | GC01P204074 |
| EIF3F | Eukaryotic Translation Initiation Factor 3 Subunit F | GC11P007966 |
| MRPS11 | Mitochondrial Ribosomal Protein S11 | GC15P088467 |
| TRAK1 | Trafficking Kinesin Protein 1 | GC03P042016 |
| CHST4 | Carbohydrate Sulfotransferase 4 | GC16P071560 |
| SF3B3 | Splicing Factor 3b Subunit 3 | GC16P070523 |
| EXOC7 | Exocyst Complex Component 7 | GC17M076080 |
| TM9SF2 | Transmembrane 9 Superfamily Member 2 | GC13P099446 |
| LOC106560211 | APOB 5' Regulatory Region | GC02U904360 |
| CETN2 | Centrin 2 | GC0XM152827 |
| COA5 | Cytochrome C Oxidase Assembly Factor 5 | GC02M098599 |
| FMNL1 | Formin Like 1 | GC17P045222 |
| ST6GALNAC2 | ST6 N-Acetylgalactosaminide Alpha-2,6-Sialyltransferase 2 | GC17M076565 |
| ATF7IP | Activating Transcription Factor 7 Interacting Protein | GC12P014365 |
| ACSL1 | Acyl-CoA Synthetase Long Chain Family Member 1 | GC04M184755 |
| ATPAF2 | ATP Synthase Mitochondrial F1 Complex Assembly Factor 2 | GC17M017977 |
| ADRA2C | Adrenoceptor Alpha 2C | GC04P003766 |
| MT-TS1 | Mitochondrially Encoded TRNA-Ser (UCN) 1 | GCMTM007447 |
| DARS2 | Aspartyl-TRNA Synthetase 2, Mitochondrial | GC01P173824 |
| MAP3K12 | Mitogen-Activated Protein Kinase Kinase Kinase 12 | GC12M053479 |
| SSX2IP | SSX Family Member 2 Interacting Protein | GC01M084643 |
| ELP4 | Elongator Acetyltransferase Complex Subunit 4 | GC11P031509 |
| MBNL2 | Muscleblind Like Splicing Regulator 2 | GC13P097141 |
| SH3BP1 | SH3 Domain Binding Protein 1 | GC22P037634 |
| PTBP3 | Polypyrimidine Tract Binding Protein 3 | GC09M112217 |
| NXF3 | Nuclear RNA Export Factor 3 | GC0XM103075 |
| SGF29 | SAGA Complex Associated Factor 29 | GC16P028554 |
| MIR517A | MicroRNA 517a | GC19P053712 |
| MIR517C | MicroRNA 517c | GC19P054118 |
| MIR1299 | MicroRNA 1299 | GC09M040929 |
| A2MP1 | Alpha-2-Macroglobulin Pseudogene 1 | GC12M009228 |
| MIR6508 | MicroRNA 6508 | GC21P039447 |
| LHX6 | LIM Homeobox 6 | GC09M122202 |
| GAD1 | Glutamate Decarboxylase 1 | GC02P170813 |
| ELK3 | ETS Transcription Factor ELK3 | GC12P096194 |
| RAMP3 | Receptor Activity Modifying Protein 3 | GC07P045163 |
| SCRIB | Scribble Planar Cell Polarity Protein | GC08M143811 |
| PDHB | Pyruvate Dehydrogenase E1 Subunit Beta | GC03M058428 |
| SEMA3C | Semaphorin 3C | GC07M080742 |
| CNTN1 | Contactin 1 | GC12P040692 |
| F11-AS1 | F11 Antisense RNA 1 | GC04M186286 |
| RBM14 | RNA Binding Motif Protein 14 | GC11P066632 |
| NAB1 | NGFI-A Binding Protein 1 | GC02P190646 |
| SAMM50 | SAMM50 Sorting And Assembly Machinery Component | GC22P043955 |
| PPP1R11 | Protein Phosphatase 1 Regulatory Inhibitor Subunit 11 | GC06P033224 |
| VEPH1 | Ventricular Zone Expressed PH Domain Containing 1 | GC03M157259 |
| RNF39 | Ring Finger Protein 39 | GC06M030651 |
| VWA7 | Von Willebrand Factor A Domain Containing 7 | GC06M032377 |
| HCG17 | HLA Complex Group 17 | GC06M030656 |
| OSMR-AS1 | OSMR Antisense RNA 1 (Head To Head) | GC05M038693 |
| MIR3166 | MicroRNA 3166 | GC11P088176 |
| LINC01265 | Long Intergenic Non-Protein Coding RNA 1265 | GC05M038713 |
| HLA-DPA2 | Major Histocompatibility Complex, Class II, DP Alpha 2 (Pseudogene) | GC06M033091 |
| RNU6-828P | RNA, U6 Small Nuclear 828, Pseudogene | GC01P010163 |
| RNU6-959P | RNA, U6 Small Nuclear 959, Pseudogene | GC02P191121 |
| RN7SL731P | RNA, 7SL, Cytoplasmic 731, Pseudogene | GC01P010368 |
| RNU6-37P | RNA, U6 Small Nuclear 37, Pseudogene | GC01P010299 |
| RPS20P10 | Ribosomal Protein S20 Pseudogene 10 | GC02M071984 |
| LOC105376725 | Uncharacterized LOC105376725 | GC01M010358 |
| HNRNPUL1 | Heterogeneous Nuclear Ribonucleoprotein U Like 1 | GC19P041262 |
| RNASE1 | Ribonuclease A Family Member 1, Pancreatic | GC14M020801 |
| PLCD3 | Phospholipase C Delta 3 | GC17M045108 |
| CNBP | CCHC-Type Zinc Finger Nucleic Acid Binding Protein | GC03M129167 |
| IFIT2 | Interferon Induced Protein With Tetratricopeptide Repeats 2 | GC10P089284 |
| AP1G1 | Adaptor Related Protein Complex 1 Subunit Gamma 1 | GC16M071729 |
| IGHMBP2 | Immunoglobulin Mu DNA Binding Protein 2 | GC11P068921 |
| ST8SIA1 | ST8 Alpha-N-Acetyl-Neuraminide Alpha-2,8-Sialyltransferase 1 | GC12M022063 |
| RBP2 | Retinol Binding Protein 2 | GC03M139452 |
| TEAD2 | TEA Domain Transcription Factor 2 | GC19M049341 |
| TSGA10 | Testis Specific 10 | GC02M098997 |
| NENF | Neudesin Neurotrophic Factor | GC01P212432 |
| C11orf65 | Chromosome 11 Open Reading Frame 65 | GC11M108308 |
| BHLHA15 | Basic Helix-Loop-Helix Family Member A15 | GC07P098211 |
| TEFM | Transcription Elongation Factor, Mitochondrial | GC17M030897 |
| MYO1B | Myosin IB | GC02P191246 |
| SPRR1A | Small Proline Rich Protein 1A | GC01P152984 |
| UGT1A8 | UDP Glucuronosyltransferase Family 1 Member A8 | GC02P233618 |
| PDE3A | Phosphodiesterase 3A | GC12P020294 |
| LSM4 | LSM4 Homolog, U6 Small Nuclear RNA And MRNA Degradation Associated | GC19M018306 |
| DHX30 | DExH-Box Helicase 30 | GC03P047802 |
| BZW1 | Basic Leucine Zipper And W2 Domains 1 | GC02P200810 |
| TINAGL1 | Tubulointerstitial Nephritis Antigen Like 1 | GC01P031576 |
| RNF123 | Ring Finger Protein 123 | GC03P049689 |
| PLA2G7 | Phospholipase A2 Group VII | GC06M046704 |
| IL17D | Interleukin 17D | GC13P020702 |
| LOC111162620 | TAp63 Promoter Of Tumor Protein P63 | GC03U903196 |
| FFAR4 | Free Fatty Acid Receptor 4 | GC10P093566 |
| MACIR | Macrophage Immunometabolism Regulator | GC05P103259 |
| LRRC53 | Leucine Rich Repeat Containing 53 | GC01M074469 |
| NDUFA10 | NADH:Ubiquinone Oxidoreductase Subunit A10 | GC02M239893 |
| TCN2 | Transcobalamin 2 | GC22P030606 |
| HECTD1 | HECT Domain E3 Ubiquitin Protein Ligase 1 | GC14M031100 |
| KIR3DL3 | Killer Cell Immunoglobulin Like Receptor, Three Ig Domains And Long Cytoplasmic Tail 3 | GC19P055319 |
| SIN3A | SIN3 Transcription Regulator Family Member A | GC15M075369 |
| NDUFS1 | NADH:Ubiquinone Oxidoreductase Core Subunit S1 | GC02M206114 |
| FAM172A | Family With Sequence Similarity 172 Member A | GC05M093617 |
| UBQLN2 | Ubiquilin 2 | GC0XP056563 |
| GIMAP6 | GTPase, IMAP Family Member 6 | GC07M150625 |
| ORM1 | Orosomucoid 1 | GC09P114323 |
| POTEI | POTE Ankyrin Domain Family Member I | GC02M130459 |
| POTEJ | POTE Ankyrin Domain Family Member J | GC02P130611 |
| ABCF2 | ATP Binding Cassette Subfamily F Member 2 | GC07M151207 |
| ETFB | Electron Transfer Flavoprotein Subunit Beta | GC19M051345 |
| NELFCD | Negative Elongation Factor Complex Member C/D | GC20P058981 |
| GCFC2 | GC-Rich Sequence DNA-Binding Factor 2 | GC02M075652 |
| DYNC1I2 | Dynein Cytoplasmic 1 Intermediate Chain 2 | GC02P171687 |
| CUBN | Cubilin | GC10M016824 |
| ATP5F1E | ATP Synthase F1 Subunit Epsilon | GC20M059026 |
| UQCRC2 | Ubiquinol-Cytochrome C Reductase Core Protein 2 | GC16P021963 |
| MIR520B | MicroRNA 520b | GC19P054099 |
| HIPK3 | Homeodomain Interacting Protein Kinase 3 | GC11P033278 |
| PLA2G4B | Phospholipase A2 Group IVB | GC15P041837 |
| GPM6B | Glycoprotein M6B | GC0XM013789 |
| EPG5 | Ectopic P-Granules Autophagy Protein 5 Homolog | GC18M045800 |
| SIGMAR1 | Sigma Non-Opioid Intracellular Receptor 1 | GC09M034634 |
| SCG3 | Secretogranin III | GC15P051681 |
| NFIC | Nuclear Factor I C | GC19P003314 |
| TTC28 | Tetratricopeptide Repeat Domain 28 | GC22M027978 |
| CYP3A43 | Cytochrome P450 Family 3 Subfamily A Member 43 | GC07P099829 |
| POLL | DNA Polymerase Lambda | GC10M101578 |
| GDF11 | Growth Differentiation Factor 11 | GC12P055743 |
| POTEM | POTE Ankyrin Domain Family Member M | GC14P018967 |
| SEMA3B-AS1 | SEMA3B Antisense RNA 1 (Head To Head) | GC03M050266 |
| SUB1 | SUB1 Regulator Of Transcription | GC05P032533 |
| PLA2G1B | Phospholipase A2 Group IB | GC12M120322 |
| CDKAL1 | CDK5 Regulatory Subunit Associated Protein 1 Like 1 | GC06P020534 |
| KPNA4 | Karyopherin Subunit Alpha 4 | GC03M160494 |
| NDUFS3 | NADH:Ubiquinone Oxidoreductase Core Subunit S3 | GC11P047567 |
| FOXH1 | Forkhead Box H1 | GC08M144473 |
| MRPS22 | Mitochondrial Ribosomal Protein S22 | GC03P139005 |
| NEUROG3 | Neurogenin 3 | GC10M069571 |
| COQ8A | Coenzyme Q8A | GC01P226899 |
| ENTPD5 | Ectonucleoside Triphosphate Diphosphohydrolase 5 (Inactive) | GC14M073958 |
| CNTNAP2 | Contactin Associated Protein 2 | GC07P146116 |
| LOC106096416 | KRT18 Locus Control Region | GC12U902688 |
| GIPR | Gastric Inhibitory Polypeptide Receptor | GC19P045668 |
| ADH7 | Alcohol Dehydrogenase 7 (Class IV), Mu Or Sigma Polypeptide | GC04M099412 |
| USP25 | Ubiquitin Specific Peptidase 25 | GC21P015730 |
| RNPEP | Arginyl Aminopeptidase | GC01P201982 |
| SRCAP | Snf2 Related CREBBP Activator Protein | GC16P030700 |
| CAD | Carbamoyl-Phosphate Synthetase 2, Aspartate Transcarbamylase, And Dihydroorotase | GC02P027217 |
| NRARP | NOTCH Regulated Ankyrin Repeat Protein | GC09M137299 |
| PARP4 | Poly(ADP-Ribose) Polymerase Family Member 4 | GC13M024420 |
| SUPT20H | SPT20 Homolog, SAGA Complex Component | GC13M037009 |
| ESRP2 | Epithelial Splicing Regulatory Protein 2 | GC16M068229 |
| USP27X | Ubiquitin Specific Peptidase 27 X-Linked | GC0XP049879 |
| KCNJ3 | Potassium Inwardly Rectifying Channel Subfamily J Member 3 | GC02P154698 |
| POTEG | POTE Ankyrin Domain Family Member G | GC14M019402 |
| PDPK2P | 3-Phosphoinositide Dependent Protein Kinase 2, Pseudogene | GC16M002637 |
| ACTG1P1 | Actin Gamma 1 Pseudogene 1 | GC03P139493 |
| ACTG1P14 | Actin Gamma 1 Pseudogene 14 | GC09P006844 |
| ACTG1P19 | Actin Gamma 1 Pseudogene 19 | GC09P100731 |
| LOC402221 | Actin Beta Pseudogene | GC05P098338 |
| ZWINT | ZW10 Interacting Kinetochore Protein | GC10M056357 |
| LOC110596866 | CYP7A1 5' Regulatory Region | GC08U902747 |
| PLSCR1 | Phospholipid Scramblase 1 | GC03M146515 |
| CPXM2 | Carboxypeptidase X, M14 Family Member 2 | GC10M123710 |
| CILK1 | Ciliogenesis Associated Kinase 1 | GC06M053002 |
| EXOSC8 | Exosome Component 8 | GC13P036998 |
| UMOD | Uromodulin | GC16M020344 |
| C4BPA | Complement Component 4 Binding Protein Alpha | GC01P207105 |
| NETO2 | Neuropilin And Tolloid Like 2 | GC16M047077 |
| MIR543 | MicroRNA 543 | GC14P104302 |
| P2RY6 | Pyrimidinergic Receptor P2Y6 | GC11P073264 |
| ABCA2 | ATP Binding Cassette Subfamily A Member 2 | GC09M137007 |
| MAF | MAF BZIP Transcription Factor | GC16M079212 |
| MIR486-2 | MicroRNA 486-2 | GC08P041679 |
| SAMHD1 | SAM And HD Domain Containing Deoxynucleoside Triphosphate Triphosphohydrolase 1 | GC20M036890 |
| TALDO1 | Transaldolase 1 | GC11P000749 |
| PSMA3 | Proteasome 20S Subunit Alpha 3 | GC14P058244 |
| GATAD1 | GATA Zinc Finger Domain Containing 1 | GC07P092447 |
| KIR3DL2 | Killer Cell Immunoglobulin Like Receptor, Three Ig Domains And Long Cytoplasmic Tail 2 | GC19P055295 |
| MIR616 | MicroRNA 616 | GC12M057519 |
| GTF2H2 | General Transcription Factor IIH Subunit 2 | GC05M071034 |
| SLC13A5 | Solute Carrier Family 13 Member 5 | GC17M006684 |
| TACR2 | Tachykinin Receptor 2 | GC10M069403 |
| CAPN10 | Calpain 10 | GC02P240586 |
| CDK11A | Cyclin Dependent Kinase 11A | GC01M001702 |
| SDF4 | Stromal Cell Derived Factor 4 | GC01M001216 |
| VDAC2 | Voltage Dependent Anion Channel 2 | GC10P075210 |
| MGAM | Maltase-Glucoamylase | GC07P144806 |
| APOL1 | Apolipoprotein L1 | GC22P036253 |
| LGALS2 | Galectin 2 | GC22M037570 |
| H3-3B | H3.3 Histone B | GC17M075780 |
| TTPA | Alpha Tocopherol Transfer Protein | GC08M063048 |
| SOCS5 | Suppressor Of Cytokine Signaling 5 | GC02P046698 |
| GRHL3 | Grainyhead Like Transcription Factor 3 | GC01P024319 |
| LOC110806306 | Telomerase RNA Component (TERC) Promoter | GC03U903185 |
| SHKBP1 | SH3KBP1 Binding Protein 1 | GC19P040576 |
| PLOD3 | Procollagen-Lysine,2-Oxoglutarate 5-Dioxygenase 3 | GC07M101205 |
| RASA4 | RAS P21 Protein Activator 4 | GC07M102577 |
| DERL2 | Derlin 2 | GC17M005471 |
| NPTX2 | Neuronal Pentraxin 2 | GC07P098620 |
| EXOSC4 | Exosome Component 4 | GC08P144079 |
| NRCAM | Neuronal Cell Adhesion Molecule | GC07M108147 |
| TSTD2 | Thiosulfate Sulfurtransferase Like Domain Containing 2 | GC09M097600 |
| TRDMT1 | TRNA Aspartic Acid Methyltransferase 1 | GC10M017098 |
| MYL9 | Myosin Light Chain 9 | GC20P036541 |
| RGS3 | Regulator Of G Protein Signaling 3 | GC09P114642 |
| AFDN | Afadin, Adherens Junction Formation Factor | GC06P167827 |
| GIT2 | GIT ArfGAP 2 | GC12M109929 |
| TNFRSF17 | TNF Receptor Superfamily Member 17 | GC16P011965 |
| LOC112081413 | Sharpr-MPRA Regulatory Region 5992 | GC11U903044 |
| LOC112529895 | Sharpr-MPRA Regulatory Region 5903 | GC17U902821 |
| NDUFS8 | NADH:Ubiquinone Oxidoreductase Core Subunit S8 | GC11P068030 |
| PTCD3 | Pentatricopeptide Repeat Domain 3 | GC02P086106 |
| SLC44A3 | Solute Carrier Family 44 Member 3 | GC01P094820 |
| CSRNP1 | Cysteine And Serine Rich Nuclear Protein 1 | GC03M039159 |
| DUSP11 | Dual Specificity Phosphatase 11 | GC02M073762 |
| HMOX2 | Heme Oxygenase 2 | GC16P004474 |
| USP13 | Ubiquitin Specific Peptidase 13 | GC03P179652 |
| CMTM7 | CKLF Like MARVEL Transmembrane Domain Containing 7 | GC03P032409 |
| RNF111 | Ring Finger Protein 111 | GC15P058866 |
| RAP1GDS1 | Rap1 GTPase-GDP Dissociation Stimulator 1 | GC04P098261 |
| CERS6 | Ceramide Synthase 6 | GC02P168455 |
| OS9 | OS9 Endoplasmic Reticulum Lectin | GC12P057693 |
| LARGE2 | LARGE Xylosyl- And Glucuronyltransferase 2 | GC11P045920 |
| ACADS | Acyl-CoA Dehydrogenase Short Chain | GC12P120822 |
| PEX6 | Peroxisomal Biogenesis Factor 6 | GC06M042963 |
| G6PC2 | Glucose-6-Phosphatase Catalytic Subunit 2 | GC02P168901 |
| DDRGK1 | DDRGK Domain Containing 1 | GC20M003191 |
| THADA | THADA Armadillo Repeat Containing | GC02M043193 |
| GIMAP5 | GTPase, IMAP Family Member 5 | GC07P150722 |
| MNX1 | Motor Neuron And Pancreas Homeobox 1 | GC07M156994 |
| GDF9 | Growth Differentiation Factor 9 | GC05M132861 |
| MELTF | Melanotransferrin | GC03M196987 |
| BMP8A | Bone Morphogenetic Protein 8a | GC01P039492 |
| ZRANB1 | Zinc Finger RANBP2-Type Containing 1 | GC10P124918 |
| CR1L | Complement C3b/C4b Receptor 1 Like | GC01P207646 |
| SPINK13 | Serine Peptidase Inhibitor Kazal Type 13 | GC05P148268 |
| LINC01551 | Long Intergenic Non-Protein Coding RNA 1551 | GC14P028772 |
| DLGAP1-AS1 | DLGAP1 Antisense RNA 1 | GC18P003597 |
| SOX9-AS1 | SOX9 Antisense RNA 1 | GC17M072034 |
| NCBP2AS2 | NCBP2 Antisense 2 (Head To Head) | GC03P196944 |
| LINC00364 | Long Intergenic Non-Protein Coding RNA 364 | GC13P067372 |
| LINC01391 | Long Intergenic Non-Protein Coding RNA 1391 | GC03M138935 |
| LINC01224 | Long Intergenic Non-Protein Coding RNA 1224 | GC19M023737 |
| MIR888 | MicroRNA 888 | GC0XM145996 |
| AURKAP1 | Aurora Kinase A Pseudogene 1 | GC01M220267 |
| LINC02027 | Long Intergenic Non-Protein Coding RNA 2027 | GC03P080993 |
| LOC100507377 | Uncharacterized LOC100507377 | GC12M074133 |
| ZKSCAN2-DT | ZKSCAN2 Divergent Transcript | GC16P026373 |
| LOC111216291 | ADH6 Promoter | GC04U902750 |
| ADRA1B | Adrenoceptor Alpha 1B | GC05P159867 |
| INTS2 | Integrator Complex Subunit 2 | GC17M061865 |
| GPC4 | Glypican 4 | GC0XM133300 |
| RBBP9 | RB Binding Protein 9, Serine Hydrolase | GC20M018486 |
| POLR2K | RNA Polymerase II Subunit K | GC08P100150 |
| SLC30A8 | Solute Carrier Family 30 Member 8 | GC08P116950 |
| RFX6 | Regulatory Factor X6 | GC06P116877 |
| MRPL13 | Mitochondrial Ribosomal Protein L13 | GC08M120377 |
| RPA4 | Replication Protein A4 | GC0XP096883 |
| TIPRL | TOR Signaling Pathway Regulator | GC01P168148 |
| TM4SF4 | Transmembrane 4 L Six Family Member 4 | GC03P149473 |
| CMPK1 | Cytidine/Uridine Monophosphate Kinase 1 | GC01P047333 |
| SLC27A4 | Solute Carrier Family 27 Member 4 | GC09P128340 |
| KLF15 | Kruppel Like Factor 15 | GC03M126342 |
| CHMP3 | Charged Multivesicular Body Protein 3 | GC02M086505 |
| PLEKHO2 | Pleckstrin Homology Domain Containing O2 | GC15P072823 |
| FOXN2 | Forkhead Box N2 | GC02P048314 |
| MAP3K11 | Mitogen-Activated Protein Kinase Kinase Kinase 11 | GC11M065598 |
| KDM2A | Lysine Demethylase 2A | GC11P067119 |
| NDUFA8 | NADH:Ubiquinone Oxidoreductase Subunit A8 | GC09M122132 |
| RAB22A | RAB22A, Member RAS Oncogene Family | GC20P058309 |
| CASP12 | Caspase 12 (Gene/Pseudogene) | GC11M104885 |
| PACRG | Parkin Coregulated | GC06P162727 |
| LAS1L | LAS1 Like Ribosome Biogenesis Factor | GC0XM065512 |
| PEX5 | Peroxisomal Biogenesis Factor 5 | GC12P007889 |
| LCT | Lactase | GC02M135787 |
| CPB1 | Carboxypeptidase B1 | GC03P148791 |
| UCP3 | Uncoupling Protein 3 | GC11M074000 |
| SNX1 | Sorting Nexin 1 | GC15P064094 |
| AP2A1 | Adaptor Related Protein Complex 2 Subunit Alpha 1 | GC19P049766 |
| BMP3 | Bone Morphogenetic Protein 3 | GC04P081030 |
| C12orf43 | Chromosome 12 Open Reading Frame 43 | GC12M121000 |
| SRCIN1 | SRC Kinase Signaling Inhibitor 1 | GC17M038530 |
| KDM3B | Lysine Demethylase 3B | GC05P138352 |
| ABCD1 | ATP Binding Cassette Subfamily D Member 1 | GC0XP153724 |
| MAP3K10 | Mitogen-Activated Protein Kinase Kinase Kinase 10 | GC19P040191 |
| TAX1BP1 | Tax1 Binding Protein 1 | GC07P027739 |
| ERLIN2 | ER Lipid Raft Associated 2 | GC08P037736 |
| SAP130 | Sin3A Associated Protein 130 | GC02M127967 |
| ZNF362 | Zinc Finger Protein 362 | GC01P033256 |
| ENPP1 | Ectonucleotide Pyrophosphatase/Phosphodiesterase 1 | GC06P131808 |
| ADARB1 | Adenosine Deaminase RNA Specific B1 | GC21P045073 |
| NANS | N-Acetylneuraminate Synthase | GC09P098056 |
| ETNPPL | Ethanolamine-Phosphate Phospho-Lyase | GC04M108742 |
| ADSL | Adenylosuccinate Lyase | GC22P040346 |
| HIRA | Histone Cell Cycle Regulator | GC22M019318 |
| DR1 | Down-Regulator Of Transcription 1 | GC01P093345 |
| YIPF5 | Yip1 Domain Family Member 5 | GC05M144158 |
| PRDM4 | PR/SET Domain 4 | GC12M107732 |
| NOMO1 | NODAL Modulator 1 | GC16P015037 |
| NDUFA1 | NADH:Ubiquinone Oxidoreductase Subunit A1 | GC0XP119871 |
| CEP78 | Centrosomal Protein 78 | GC09P078236 |
| CLTRN | Collectrin, Amino Acid Transport Regulator | GC0XM015629 |
| IFNL1 | Interferon Lambda 1 | GC19P039296 |
| NPTXR | Neuronal Pentraxin Receptor | GC22M038818 |
| NDUFAF4 | NADH:Ubiquinone Oxidoreductase Complex Assembly Factor 4 | GC06M096889 |
| EEF1AKNMT | EEF1A Lysine And N-Terminal Methyltransferase | GC01P171782 |
| FAM3B | FAM3 Metabolism Regulating Signaling Molecule B | GC21P041304 |
| STIM2 | Stromal Interaction Molecule 2 | GC04P026859 |
| SLC35B3 | Solute Carrier Family 35 Member B3 | GC06M008413 |
| PLTP | Phospholipid Transfer Protein | GC20M045898 |
| TIMMDC1 | Translocase Of Inner Mitochondrial Membrane Domain Containing 1 | GC03P119498 |
| EFNA4 | Ephrin A4 | GC01P155063 |
| HCFC1 | Host Cell Factor C1 | GC0XM153947 |
| LMO2 | LIM Domain Only 2 | GC11M033858 |
| ETFDH | Electron Transfer Flavoprotein Dehydrogenase | GC04P158672 |
| C12orf65 | Chromosome 12 Open Reading Frame 65 | GC12P123232 |
| INTS11 | Integrator Complex Subunit 11 | GC01M001332 |
| HSP90AA2P | Heat Shock Protein 90 Alpha Family Class A Member 2, Pseudogene | GC11M027888 |
| MYH10 | Myosin Heavy Chain 10 | GC17M008474 |
| STAG1 | Stromal Antigen 1 | GC03M136336 |
| STK38L | Serine/Threonine Kinase 38 Like | GC12P027243 |
| PIGK | Phosphatidylinositol Glycan Anchor Biosynthesis Class K | GC01M077088 |
| RGMA | Repulsive Guidance Molecule BMP Co-Receptor A | GC15M093035 |
| NFIL3 | Nuclear Factor, Interleukin 3 Regulated | GC09M091409 |
| CD8B | CD8b Molecule | GC02M086815 |
| PDE4DIP | Phosphodiesterase 4D Interacting Protein | GC01P148808 |
| SNRNP70 | Small Nuclear Ribonucleoprotein U1 Subunit 70 | GC19P049085 |
| CHPF | Chondroitin Polymerizing Factor | GC02M219538 |
| DNAJC7 | DnaJ Heat Shock Protein Family (Hsp40) Member C7 | GC17M041977 |
| ZNF510 | Zinc Finger Protein 510 | GC09M096755 |
| MAP7D1 | MAP7 Domain Containing 1 | GC01P036155 |
| ZNF395 | Zinc Finger Protein 395 | GC08M028345 |
| MIR431 | MicroRNA 431 | GC14P104251 |
| PIAS2 | Protein Inhibitor Of Activated STAT 2 | GC18M046808 |
| MAFIP | MAFF Interacting Protein (Pseudogene) | GC14M9U0053 |
| ASH1L | ASH1 Like Histone Lysine Methyltransferase | GC01M155335 |
| CLIC4 | Chloride Intracellular Channel 4 | GC01P024745 |
| DPAGT1 | Dolichyl-Phosphate N-Acetylglucosaminephosphotransferase 1 | GC11M119096 |
| SERAC1 | Serine Active Site Containing 1 | GC06M158109 |
| ABITRAM | Actin Binding Transcription Modulator | GC09P108935 |
| F8 | Coagulation Factor VIII | GC0XM154835 |
| GNE | Glucosamine (UDP-N-Acetyl)-2-Epimerase/N-Acetylmannosamine Kinase | GC09M036214 |
| GNPTG | N-Acetylglucosamine-1-Phosphate Transferase Subunit Gamma | GC16P001351 |
| ERVV-1 | Endogenous Retrovirus Group V Member 1, Envelope | GC19P053013 |
| NAGLU | N-Acetyl-Alpha-Glucosaminidase | GC17P042535 |
| RIPK2 | Receptor Interacting Serine/Threonine Kinase 2 | GC08P089758 |
| ARNT2 | Aryl Hydrocarbon Receptor Nuclear Translocator 2 | GC15P080404 |
| GALE | UDP-Galactose-4-Epimerase | GC01M023795 |
| TOM1L2 | Target Of Myb1 Like 2 Membrane Trafficking Protein | GC17M017843 |
| DELE1 | DAP3 Binding Cell Death Enhancer 1 | GC05P142885 |
| ABR | ABR Activator Of RhoGEF And GTPase | GC17M001003 |
| JKAMP | JNK1/MAPK8 Associated Membrane Protein | GC14P059484 |
| PHACTR3 | Phosphatase And Actin Regulator 3 | GC20P059577 |
| ROMO1 | Reactive Oxygen Species Modulator 1 | GC20P035699 |
| CYGB | Cytoglobin | GC17M076527 |
| MPV17 | Mitochondrial Inner Membrane Protein MPV17 | GC02M027309 |
| C1S | Complement C1s | GC12P007884 |
| PPARGC1B | PPARG Coactivator 1 Beta | GC05P149730 |
| MARCKSL1 | MARCKS Like 1 | GC01M032334 |
| CBFA2T3 | CBFA2/RUNX1 Partner Transcriptional Co-Repressor 3 | GC16M088874 |
| PPP3CA | Protein Phosphatase 3 Catalytic Subunit Alpha | GC04M101024 |
| PPP1R1C | Protein Phosphatase 1 Regulatory Inhibitor Subunit 1C | GC02P181954 |
| EVC2 | EvC Ciliary Complex Subunit 2 | GC04M005534 |
| KLRB1 | Killer Cell Lectin Like Receptor B1 | GC12M013361 |
| MIR939 | MicroRNA 939 | GC08M144394 |
| MIR1285-2 | MicroRNA 1285-2 | GC02M070252 |
| NFE2 | Nuclear Factor, Erythroid 2 | GC12M054292 |
| BRD8 | Bromodomain Containing 8 | GC05M138150 |
| HADHB | Hydroxyacyl-CoA Dehydrogenase Trifunctional Multienzyme Complex Subunit Beta | GC02P026243 |
| TBC1D24 | TBC1 Domain Family Member 24 | GC16P002475 |
| RNMT | RNA Guanine-7 Methyltransferase | GC18P013716 |
| PHF5A | PHD Finger Protein 5A | GC22M041459 |
| USP42 | Ubiquitin Specific Peptidase 42 | GC07P006078 |
| TENT5A | Terminal Nucleotidyltransferase 5A | GC06M081494 |
| EED | Embryonic Ectoderm Development | GC11P086244 |
| LOC110599585 | CYP2E1 5' Regulatory Region | GC10U902740 |
| ACSL3 | Acyl-CoA Synthetase Long Chain Family Member 3 | GC02P222860 |
| METAP2 | Methionyl Aminopeptidase 2 | GC12P095473 |
| NDUFA4 | NDUFA4 Mitochondrial Complex Associated | GC07M010938 |
| NDUFB3 | NADH:Ubiquinone Oxidoreductase Subunit B3 | GC02P201071 |
| COQ9 | Coenzyme Q9 | GC16P057447 |
| PTCD1 | Pentatricopeptide Repeat Domain 1 | GC07M099419 |
| NUBPL | Nucleotide Binding Protein Like | GC14P031489 |
| PIAS4 | Protein Inhibitor Of Activated STAT 4 | GC19P004007 |
| RBM24 | RNA Binding Motif Protein 24 | GC06P017281 |
| ZFAND4 | Zinc Finger AN1-Type Containing 4 | GC10M045615 |
| MIR889 | MicroRNA 889 | GC14P104486 |
| LOC109286563 | TBX21 Promoter Region | GC17U902722 |
| GAD2 | Glutamate Decarboxylase 2 | GC10P026216 |
| IL20 | Interleukin 20 | GC01P206866 |
| RGS2 | Regulator Of G Protein Signaling 2 | GC01P192809 |
| KYAT1 | Kynurenine Aminotransferase 1 | GC09M128833 |
| APOA5 | Apolipoprotein A5 | GC11M116789 |
| AOX1 | Aldehyde Oxidase 1 | GC02P200585 |
| VPS4A | Vacuolar Protein Sorting 4 Homolog A | GC16P069311 |
| UGT1A3 | UDP Glucuronosyltransferase Family 1 Member A3 | GC02P233729 |
| DBP | D-Box Binding PAR BZIP Transcription Factor | GC19M048630 |
| UBE2B | Ubiquitin Conjugating Enzyme E2 B | GC05P134371 |
| LIPE | Lipase E, Hormone Sensitive Type | GC19M042401 |
| MAP3K13 | Mitogen-Activated Protein Kinase Kinase Kinase 13 | GC03P185282 |
| CYP4F12 | Cytochrome P450 Family 4 Subfamily F Member 12 | GC19P015672 |
| LPGAT1 | Lysophosphatidylglycerol Acyltransferase 1 | GC01M211743 |
| PAMR1 | Peptidase Domain Containing Associated With Muscle Regeneration 1 | GC11M035453 |
| TST | Thiosulfate Sulfurtransferase | GC22M037010 |
| GLYCTK | Glycerate Kinase | GC03P052288 |
| OSBPL5 | Oxysterol Binding Protein Like 5 | GC11M003088 |
| EXPH5 | Exophilin 5 | GC11M108505 |
| RNF103 | Ring Finger Protein 103 | GC02M086603 |
| APOBEC2 | Apolipoprotein B MRNA Editing Enzyme Catalytic Subunit 2 | GC06P041053 |
| RANBP9 | RAN Binding Protein 9 | GC06M013621 |
| ZBTB10 | Zinc Finger And BTB Domain Containing 10 | GC08P080485 |
| TRIB3 | Tribbles Pseudokinase 3 | GC20P000361 |
| IGHA1 | Immunoglobulin Heavy Constant Alpha 1 | GC14M105830 |
| SLC22A4 | Solute Carrier Family 22 Member 4 | GC05P132294 |
| RPL32 | Ribosomal Protein L32 | GC03M012834 |
| ANKRD13A | Ankyrin Repeat Domain 13A | GC12P109999 |
| GRIP1 | Glutamate Receptor Interacting Protein 1 | GC12M066347 |
| PROZ | Protein Z, Vitamin K Dependent Plasma Glycoprotein | GC13P113158 |
| PEX2 | Peroxisomal Biogenesis Factor 2 | GC08M076980 |
| CNN2 | Calponin 2 | GC19P001026 |
| PYCR2 | Pyrroline-5-Carboxylate Reductase 2 | GC01M225919 |
| GPRC6A | G Protein-Coupled Receptor Class C Group 6 Member A | GC06M116793 |
| MKKS | McKusick-Kaufman Syndrome | GC20M010403 |
| TMEM67 | Transmembrane Protein 67 | GC08P093754 |
| CARD11 | Caspase Recruitment Domain Family Member 11 | GC07M002912 |
| CCR10 | C-C Motif Chemokine Receptor 10 | GC17M042678 |
| ITIH3 | Inter-Alpha-Trypsin Inhibitor Heavy Chain 3 | GC03P052794 |
| ACAA2 | Acetyl-CoA Acyltransferase 2 | GC18M049782 |
| ZC3H15 | Zinc Finger CCCH-Type Containing 15 | GC02P186486 |
| PDSS1 | Decaprenyl Diphosphate Synthase Subunit 1 | GC10P026697 |
| COQ2 | Coenzyme Q2, Polyprenyltransferase | GC04M083138 |
| DCAF8 | DDB1 And CUL4 Associated Factor 8 | GC01M160215 |
| WDR83 | WD Repeat Domain 83 | GC19P012666 |
| FAM167A | Family With Sequence Similarity 167 Member A | GC08M011421 |
| INSIG1 | Insulin Induced Gene 1 | GC07P155297 |
| HSD17B4 | Hydroxysteroid 17-Beta Dehydrogenase 4 | GC05P119452 |
| SNX16 | Sorting Nexin 16 | GC08M081799 |
| THOC1 | THO Complex 1 | GC18M000204 |
| COX4I1 | Cytochrome C Oxidase Subunit 4I1 | GC16P085798 |
| SLC25A15 | Solute Carrier Family 25 Member 15 | GC13P040789 |
| AFMID | Arylformamidase | GC17P078185 |
| RACGAP1P | Rac GTPase Activating Protein 1 Pseudogene | GC12M045062 |
| LINC02835 | Long Intergenic Non-Protein Coding RNA 2835 | GC04M065154 |
| NFATC3 | Nuclear Factor Of Activated T Cells 3 | GC16P068119 |
| PCTP | Phosphatidylcholine Transfer Protein | GC17P055750 |
| SNRPD1 | Small Nuclear Ribonucleoprotein D1 Polypeptide | GC18P021612 |
| LOC110013312 | BGLAP Promoter Region | GC01U905701 |
| EPN3 | Epsin 3 | GC17P050532 |
| SORD | Sorbitol Dehydrogenase | GC15P045023 |
| GNPAT | Glyceronephosphate O-Acyltransferase | GC01P231241 |
| EDEM1 | ER Degradation Enhancing Alpha-Mannosidase Like Protein 1 | GC03P005187 |
| DPY30 | Dpy-30 Histone Methyltransferase Complex Regulatory Subunit | GC02M031867 |
| SLC7A1 | Solute Carrier Family 7 Member 1 | GC13M029509 |
| AFF1 | AF4/FMR2 Family Member 1 | GC04P086934 |
| WDR76 | WD Repeat Domain 76 | GC15P043826 |
